# Structure-Based Discovery of Hsp90/HDAC6 Dual Inhibitors Targeting Aggressive Prostate Cancer

**DOI:** 10.1021/acs.jmedchem.5c00717

**Published:** 2025-07-23

**Authors:** Andrea Citarella, Silvia Belluti, Davide Bonanni, Davide Moi, Isabella Piccinini, Arianna Rinaldi, Chiara Papulino, Rosaria Benedetti, Laura Cuoghi, Stefano Di Ciolo, Alessandra Silvani, Lucia Altucci, Luca Pinzi, Silvia Franchini, Daniele Passarella, Claudia Sorbi, Clelia Giannini, Carol Imbriano, Giulio Rastelli

**Affiliations:** † Department of Life Sciences, 9306 University of Modena and Reggio Emilia, Via Campi 103, Modena 41125, Italy; ‡ Department of Chemistry, 9304 University of Milan, Via Golgi 19, Milano 20133, Italy; § Department of Precision Medicine, 18994 University of Campania “Luigi Vanvitelli”, Naples 80138, Italy; ∥ Program of Medical Epigenetics, Vanvitelli Hospital, Naples 80138, Italy; ⊥ Biogem Institute of Molecular and Genetic Biology, Ariano 83031, Irpino, Italy

## Abstract

HDAC6 and Heat Shock Protein 90 (Hsp90) are key regulators within the androgen response pathway, exhibiting a close interplay and mutual interaction patterns that make their combined inhibition a promising strategy for treating aggressive prostate cancer (PC). Herein, we present the structure-based design of dual inhibitors of Hsp90 and HDAC6 that leveraged the crystal structure requirements of HDAC6 and two distinct Hsp90 binding pockets. The study led to the discovery of compound **17**, a potent, nearly balanced, and selective dual inhibitor of HDAC6 and Hsp90 endowed with favorable drug-like properties. The compound demonstrated excellent antiproliferative activity across PC cell lines. In 3D tumor spheroid models, it demonstrated marked anticancer activity and ability to target both established tumor masses and tumor-initiating cell populations. Furthermore, combination studies showed marked synergistic effects that outperformed the coadministration of single-target inhibitors. Overall, compound **17** stands as a promising candidate for further preclinical evaluation against aggressive forms of PC.

## Graphical abstract

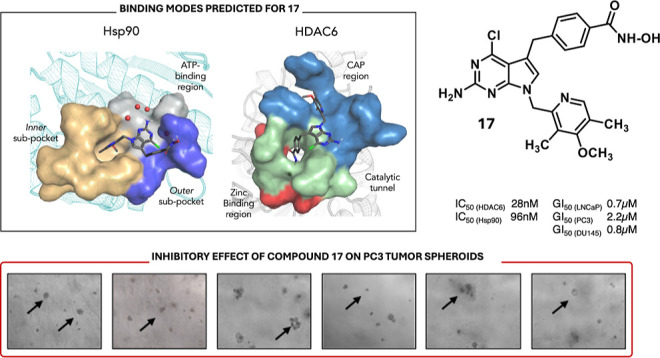

## Introduction

According to recent statistics, prostate cancer (PC) remains the second most common type of tumor in men.^1^ Treating PC patients with androgen deprivation therapy yields high 5 year survival rates. Unfortunately, the disease often progresses to a more aggressive castration-resistant form (CRPC), which has a significantly worse prognosis.^2,3^ Increasing evidence suggests that androgen receptor (AR) signaling persists in most patients with CRPC, who develop resistance to AR inhibitors as abiraterone acetate, enzalutamide, apalutamide, and darolutamide.^4–7^ Therefore, there remains a pressing need for novel and more effective treatments.

In recent years, the development of multitarget inhibitors has shown an upward trend in drug discovery.^8,9^ Multitarget drugs may provide improved efficacy and lower toxicity compared to existing drugs while also reducing the risk of drug resistance and drug–drug interactions.^8^ The success of this approach depends heavily on selecting ad hoc combinations of targets, which is often guided by clinical experience from single-target drugs, in search of synergistic activity and reduced side effects.^10,11^ However, designing molecules able to bind to specific combinations of targets remains a challenge, especially when the binding sites of these targets do not share significant similarities.^12^

Histone deacetylases (HDACs) are epigenetic regulators that modulate the expression and activity of several transcription factors, cellular mediators, and chaperones, many of which are often overactivated in cancer cells.^13,14^ In CRPC, for example, HDAC6 regulates the acetylation levels of Heat Shock Protein 90 (Hsp90), a molecular chaperone that plays a key role in the nuclear import of AR.^15^ Together, HDAC6 is a client protein of Hsp90, and its stability is regulated by this chaperone, resulting in an intriguing interplay between these two targets.^16^ The mutual involvement of HDAC6 and Hsp90 in the development and progression of several cancers suggests that the combined inhibition of both targets may demonstrate significant therapeutic value.^17,18^ In line with the polypharmacology concept, a dual inhibitor targeting both Hsp90 and HDAC6 could provide significant advantages in the treatment of complex diseases as CRPC.^19^

While the structural requirements of HDAC6 and Hsp90 inhibitors have been extensively elucidated and corroborated by a number of crystallographic studies, the design of dual inhibitors is hampered by the low structural similarity of their respective binding sites.^19^ In Hsp90, the vast majority of inhibitors target the ATP site of the N-terminal domain, establishing interactions with the key residue Asp93, nearby residues, and conserved water molecules.^20,21^ HDAC6 inhibitors need a Zinc Binding Group (ZBG) that coordinates the catalytic zinc ion, along with a linker, and sterically hindered cap groups extending toward the outer surface of the enzyme.^22,23^ Encouragingly, recent studies have reported the development of dual inhibitors of Hsp90 and HDAC with promising antiproliferative activity.^19,24–28^ These studies highlight that molecular scaffolds originally developed to inhibit one enzyme can be combined and engineered to modulate the activity of both targets.

We have previously reported the discovery of potent pyrrolo-pyrimidine and purine-based HDAC inhibitors that demonstrated potent antiproliferative activity against aggressive PC cell lines, along with marked antimigration properties.^29^ Interestingly, the 2-amino-pyrrolo­[2,3-*d*]­pyrimidine scaffold is also found in potent Hsp90 inhibitors like BIIB021, which has reached clinical phase 2 for the treatment of breast cancer and gastrointestinal stromal tumors.^30^ Therefore, this scaffold appears to be well-suited for the obtainment of the desired dual inhibitors. Building on these evidence, in this study, we present the results of a structure-based design, synthesis, and biological evaluation of two new series of N^7^-substituted and C^5^,N^7^-disubstituted derivatives of this scaffold in search of potent dual inhibitors. In addition, we investigated the effect of replacing the pyrrolo-pyrimidine with a purine moiety by preparing the corresponding N^9^-substituted derivatives. Notably, the structure-based design was based on the exploration of two distinct Hsp90 binding pockets: an “inner”, predominantly hydrophobic subpocket lined by residues Phe138, Tyr139, Gly108, and Phe22, and an “outer”, more solvent-exposed pocket located at the entrance of the Hsp90 binding cleft, surrounded by residues Lys58, Met98, Ile96, Asp102, and Leu107. In both pockets, we successfully obtained potent and nearly balanced dual inhibitors exhibiting high selectivity for HDAC6 and significant antiproliferative effects against aggressive PC cells. Most of the synthesized compounds showed potency in the low nanomolar range against HDAC6. Five of the synthesized compounds also inhibited Hsp90 with IC_50_ values below 10 μM, and two of them showed potent and nearly balanced Hsp90/HDAC6 dual inhibitory activity with IC_50_ values in the nanomolar range. Several compounds displayed antiproliferative effects against PC cell lines with different aggressiveness (i.e., LNCaP, PC3 and DU145). Remarkably, experiments performed on 3D spheroids demonstrated that the best dual inhibitor identified from in vitro assays (compound **17**) exhibited marked anticancer activity, highlighting its potential as an effective treatment option for targeting HDAC6 and Hsp90 pathways in CRPC.

## Results and Discussion

### Design of Hsp90 and HDAC6 Dual Inhibitors

The rational design of this new class of dual inhibitors started from the previously identified potent and selective HDAC6 inhibitor **1** (referred to as compound **37** in our previous publication), which features a 2-amino-pyrrolo­[2,3-*d*]­pyrimidine cap group, a substituted phenyl ring linker, and a hydroxamate ZBG.^29^ Despite the presence of the 2-amino-pyrrolo­[2,3-*d*]­pyrimidine, which is a known scaffold present in Hsp90 inhibitors, the compound itself was inactive when tested against Hsp90 (Table 1). Although the binding mode of this compound showed excellent agreement with the crystallographic structure of BIIB021 in complex with Hsp90 (Figure 1),^31^ the lack of Hsp90 inhibitory activity is likely due to the insertion of the polar hydroxamate group into a narrow hydrophobic pocket lined by Phe138, Leu107, Tyr139, and Trp162.

**Table 1:** In Vitro Inhibition of the Synthesized Molecules **2**–**17** against Recombinant HDAC6 and Hsp90 Enzymes, Expressed as IC_50_ (μM) Values^a^

Compound	HDAC6 IC_50_ (μM)	Hsp90 IC_50_ (μM)
1 ^32^	0.005	n.a.
2	0.035	n.a.
3	0.002	38.8
4	0.031	48.5
5	0.039	80.4
6	0.004	50.3
7	0.084	0.88
8	0.015	n.a.
9	0.137	n.a.
10	9.2	n.a.
11	1.2	n.a.
12	0.97	n.a.
13	0.173	n.a.
14	0.11	26
15	0.032	3.2
16	0.027	2.9
17	0.028	0.096
trichostatin A	0.001	n.t.
geldanamycin	n.t.	0.008

a Dose–response curves of the compounds are reported in Figures S1 (for HDAC6) and S2 (for Hsp90). Note: n.a.—not active up to 100 μM concentration. n.t.—not tested.

**Figure 1:**
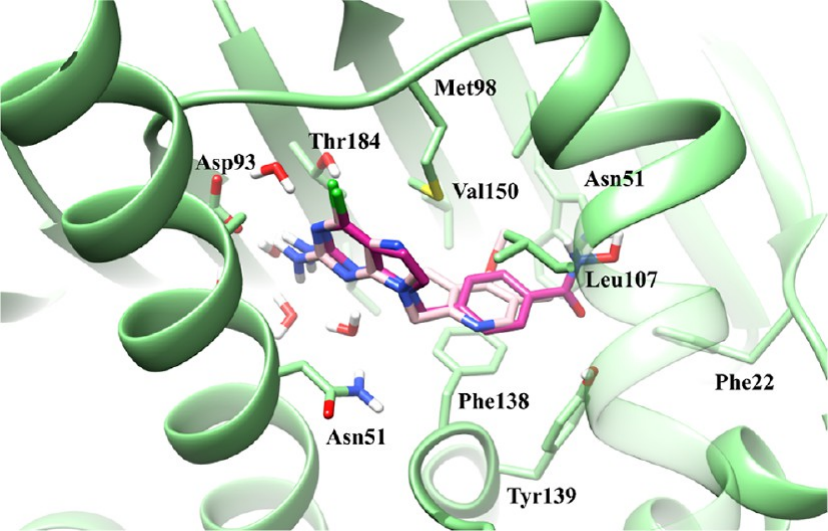
Superimposition of the predicted binding mode of **1** (purple) with the ligand BIIB021 (white) cocrystallized with Hsp90 (PDB ID: 3QDD).^31^

Therefore, further elaboration was required to make these compounds suitable for dual inhibition. To reach this goal, we explored two Hsp90 subpockets: an “inner” subpocket extending into the inner hydrophobic cleft mentioned above and another, more solvent-exposed “outer” pocket projecting outward from the ATP-binding site (Figure 2).

**Figure 2:**
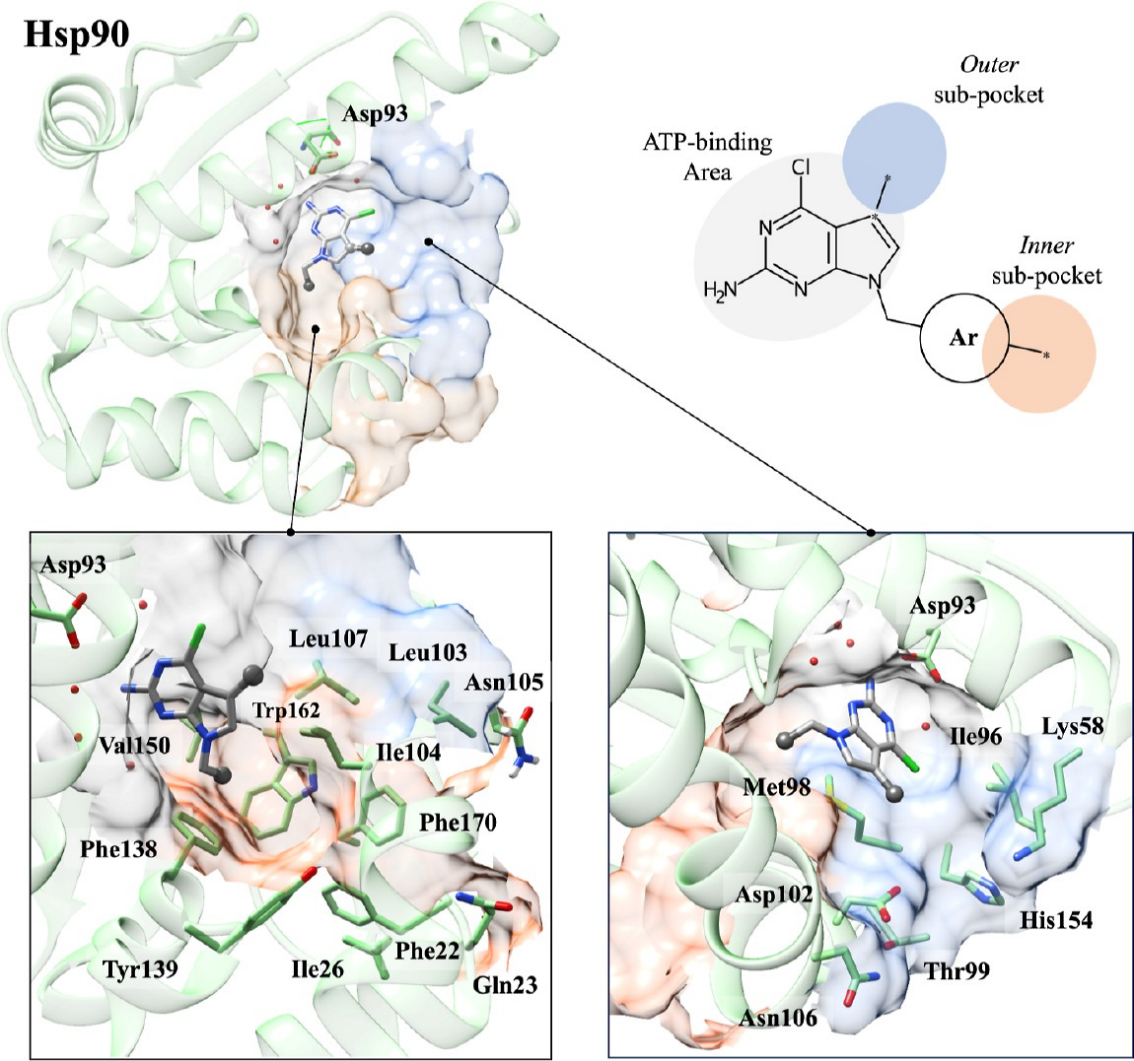
Overview of the binding of 2-amino-pyrrolo-pyrimidine derivatives to the “outer” and “inner” subpockets of Hsp90 (PDB ID: 3QDD). The anchoring points for introducing the aryl-hydroxamate group are indicated by orange and blue spheres connected to the 2-amino-pyrrolo-pyrimidine scaffold. The surfaces of the “outer” and “inner” subpockets are represented by blue and orange surfaces, respectively. Amino acid residues are colored in green, and hydrogen bonds are depicted by black dotted lines.

The inner hydrophobic subpocket, defined by residues Phe138, Tyr139, Gly108, and Phe22, has been previously explored with different aromatic or heteroaromatic rings (Figure 3, panel A).^32^ The outer subpocket is placed at the entrance of the Hsp90 binding cleft and is defined by residues Lys58, Met98, Ile96, Asp102, and Leu107. Notably, this pocket was investigated using 2-amino-pyrrolo-pyrimidine derivatives with a benzyl group at position 7, resulting in potent Hsp90 inhibitors (Figure 3, panel B).^33^

**Figure 3:**
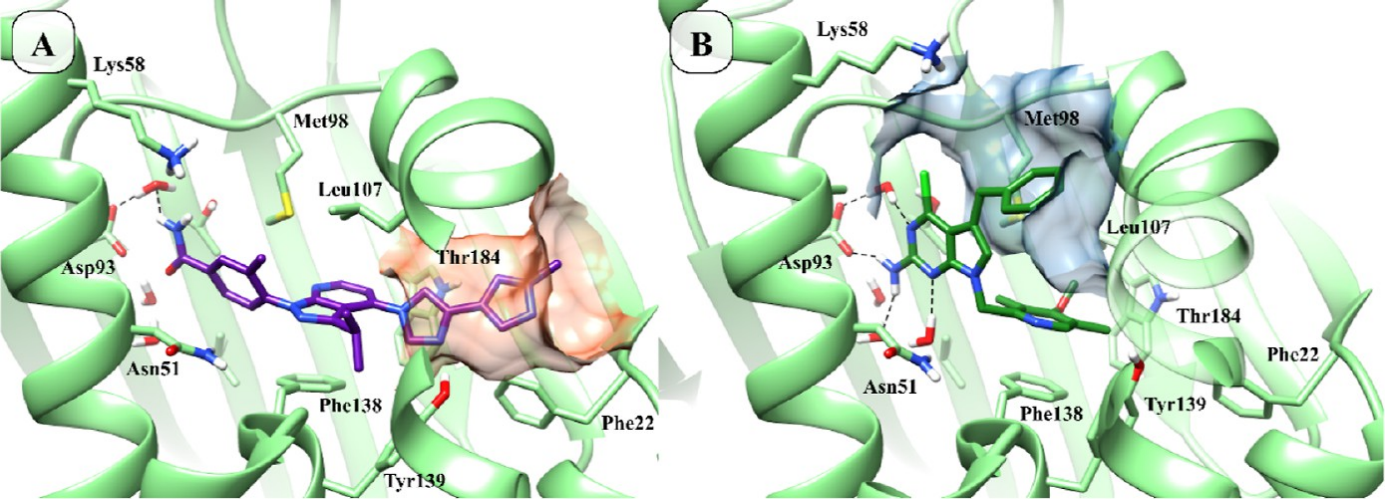
ATP binding pocket of the N-terminal domain of Hsp90, showing the surface of the inner subpocket in orange (Panel A, PDB ID: 5ZR3, crystallographic ligand ID: 9J0, compound **16d** in the original article)^32^ and the outer subpocket in blue (Panel B, PDB ID: 5H22, crystallographic ligand ID:7FT, compound **6a** in the original article).^33^

To access the inner subpocket of Hsp90, the hydroxamic acid (HA) group required for HDAC6 inhibition was linked to the 2-amino-pyrrolo-pyrimidine or purine scaffolds via various (hetero)­aryl spacers (Figure 4, panel A). In particular, the methylene bridge increases the flexibility of the molecule and helps direct the HA group toward the inner pocket (Figure 4, upper side). The (hetero)­aryl groups included 6-, 5-membered and condensed rings, in line with previous structure–activity relationship (SAR) studies reported on HDAC6 inhibitors.^22,34,35^ Specifically, we incorporated benzene, thiophene, thiazole, isoxazole, and indole rings, each bearing a hydroxamic acid group positioned appropriately to satisfy the requirements of both HDAC6 and Hsp90.

**Figure 4:**
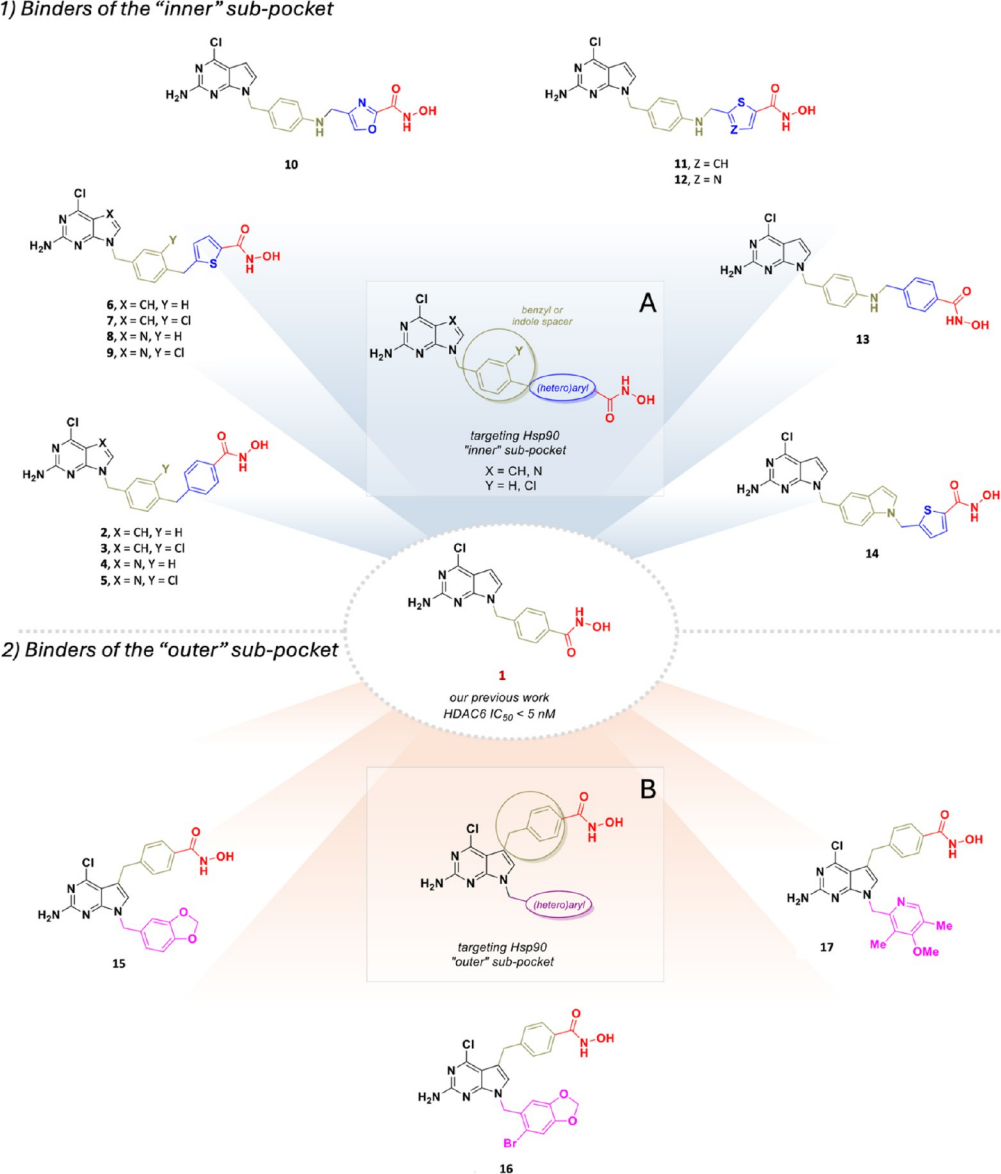
Chemical structures of the HDAC6/Hsp90 dual inhibitors **2–17** exploring the “inner” (panel A) and “outer” (panel B) Hsp90 subpockets.

To target the outer subpocket, we substituted position 5 of the 2-amino-4-chloro-pyrrolo-pyrimidine scaffold with a benzyl-hydroxamate group (Figure 4, panel B). In this case, the Hsp90 hydrophobic pocket was targeted by linking the pyrrole nitrogen to either a piperonyl or a methoxy-dimethylpyridine groups, both of which are recurring scaffolds in previously reported potent Hsp90 inhibitors.^33,36,37^ The design of the dual compounds was supported by extensive docking studies conducted in both the Hsp90 and HDAC6 binding sites to ensure that the binding requirements for both enzymes were met. The chemical structures of the designed HDAC6/Hsp90 dual inhibitors are shown in Figure 4.

### Chemistry

Four synthetic pathways were employed to obtain the final compounds **2–17**.

#### Synthesis of Hsp90 “Inner” Subpocket Binders

The synthesis of the compounds **2–9**, bearing a methylene unit between the phenyl spacer and the (hetero)­aryl-hydroxamate group, is illustrated in Scheme 1. Compounds **18**–**21** were synthesized from 4-chloro-7*H*-pyrrolo­[2,3-*d*]­pyrimidin-2-amine or 2-amino-6-chloropurine through N^7^ or N^9^ alkylation with the corresponding benzyl pinacol boronic esters. The alkylation of the purine ring was not regiospecific, resulting in the formation of both N^7^ and N^9^ isomers; the predominant N^9^ isomer was isolated, its connectivity confirmed, and used in the subsequent step. A Suzuki–Miyaura cross-coupling between **18–21** and the corresponding benzyl esters afforded **22–25** and **26–29**, which were then reacted with aqueous NH_2_OH to yield the corresponding hydroxamic acid final products (**2–9**).

**Scheme 1:**
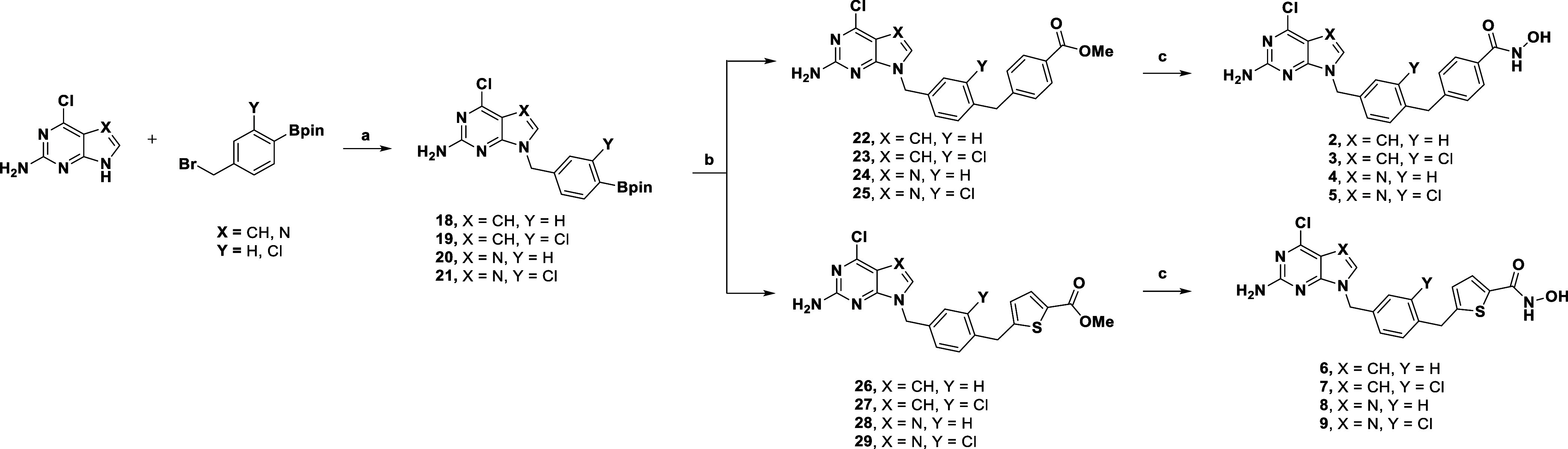
Synthesis of **2–9** ^a^

a Reagents and conditions: (a) K_2_CO_3_ or NaH, dry DMF, rt, overnight, 47–60%; (b) appropriate halide, Pd­(PPh_3_)_4_, K_2_CO_3_ or Na_2_CO_3_, EtOH/toluene 1:3 (v/v), 90 °C, 4–6 h, 13–59%; and (c) NH_2_OH 50% W, NaOH 1M, THF/MeOH 1:1 (v/v), rt, 2 h, 12–48%.

The synthesis of the compounds **10–13**, bearing a NH–CH_2_ unit between the phenyl spacer and the (hetero)­aryl-hydroxamate group, is depicted in Scheme 2. The commercially available 4-chloro-7*H*-pyrrolo­[2,3-*d*]­pyrimidin-2-amine was first alkylated with 4-nitrobenzyl chloride to afford **30**, which was then converted to **31** through reduction of the nitro group. Compound **31** was then reacted with the respective commercially available halide to yield the carboxylic esters **32–35**. In this case, to avoid the formation of undesired side products, a different approach was taken for the synthesis of the final hydroxamic acid products. Carboxylic esters **32**, **33**, **34**, and **35** were conveniently hydrolyzed under mild conditions using LiOH in a 1,4-dioxane/water 1:1 solution. The completion of this process was confirmed by LC–MS; however, the product was not isolated, and the crude material was used without further purification in the next step. *O*-Trimethylsilylhydroxylamine (NH_2_OTMS) was employed to perform the final functional group interconversion, resulting in the formation of the final hydroxamic acid derivatives via amide coupling mediated by HOBt/EDCI. Compounds **10–13** were obtained in good yields through a single step that included both coupling and deprotection of the hydroxyl group, as previously described.^38^

**Scheme 2:**
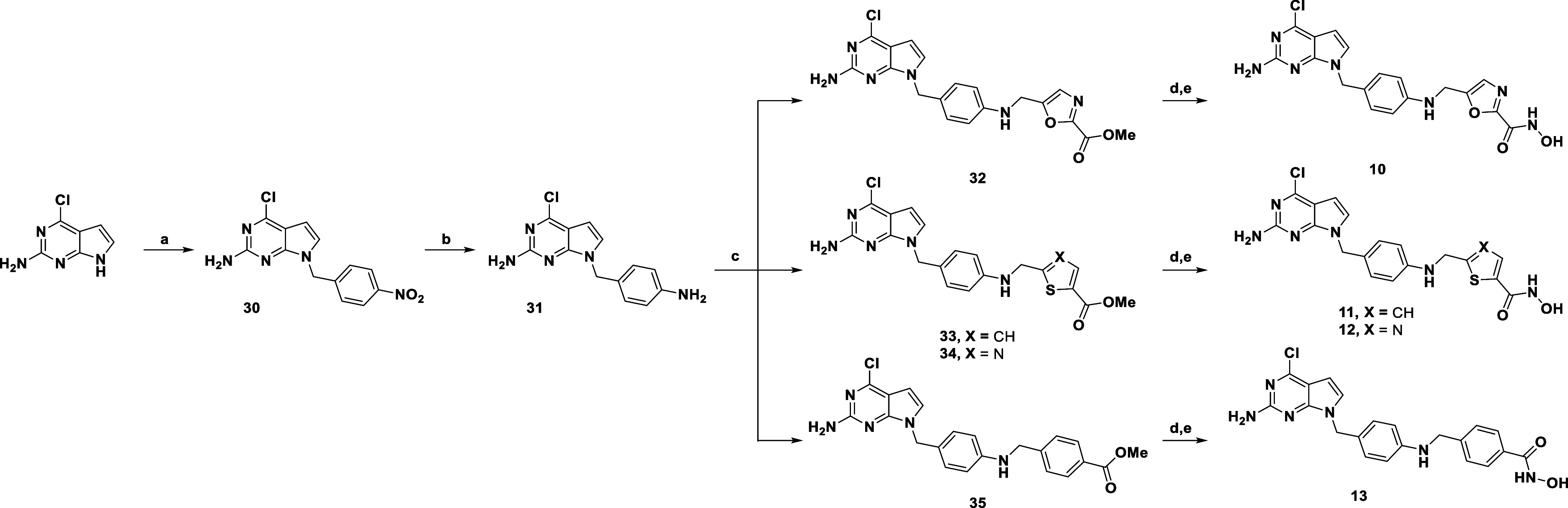
Synthesis of **10–13** ^a^

a Reagents and conditions: (a) 1-(bromomethyl)-4-nitrobenzene, K_2_CO_3_, dry DMF, rt, overnight, 70%; (b) Fe, NH_4_Cl, water/EtOH 1:1 (v/v), 80 °C, 2 h, 84%; (c) appropriate halide, K_2_CO_3_ or TEA, dry DMF, rt, overnight, 42–51%; (d) LiOH, water/1,4-dioxane 1:1 (v/v), at room temperature (rt), overnight;^1^ and (e) NH_2_OTMS, HOBt, EDCI, dry DMF, rt, overnight, 25–47%. Note: ^1^completion of the reaction was confirmed by LC–MS, the product was not isolated, and the crude was used without further purification.

The indole derivative **14** was synthesized according to Scheme 3. 5-Methylindole was protected with Boc_2_O in the presence of catalytic amounts of DMAP, affording **36** in excellent yield. Radical bromination of **36** with NBS in CCl_4_ provided **37** with moderate yields. It is important to note that attempts to substitute CCl_4_ with environmentally friendly solvents, such as EtOAc and ACN, did not yield the desired product. Instead, multiple side products were formed, with bromination occurring at the aromatic ring. Halide **37** was then reacted with the pyrrolo-pyrimidine scaffold in acetone achieving product **38** in good yield. After deprotecting the Boc group using TFE to form intermediate **39**,^39^ another alkylation step was conducted to obtain ester **40**. Subsequently, ester **40** was transformed into the final hydroxamic acid product **14** using the previously described procedure.

**Scheme 3:**
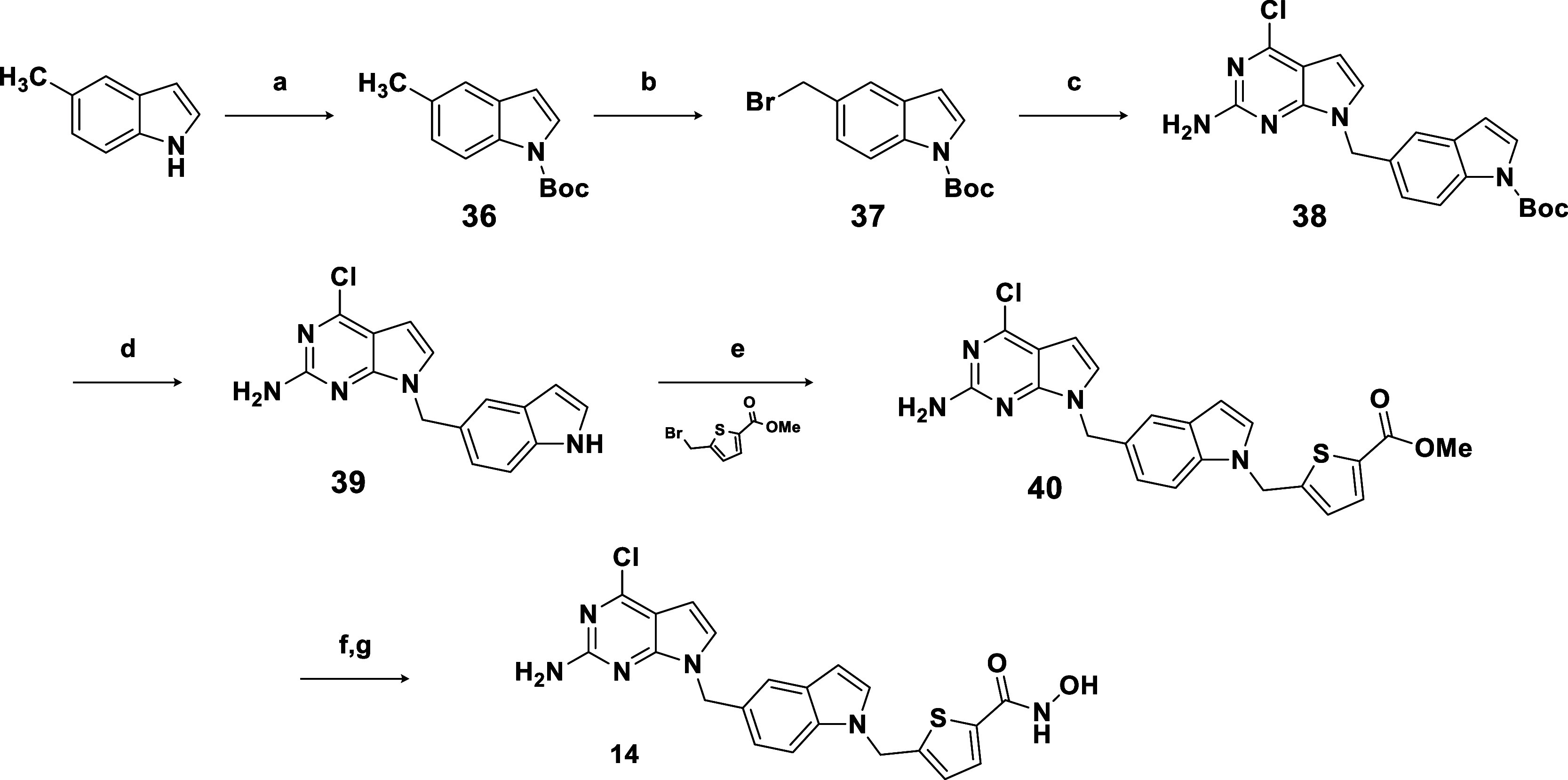
Synthesis of **14** ^a^

a Reagents and conditions: (a) DMAP, Boc_2_O, dry ACN, rt, overnight, 96%; (b) NBS, AIBN, CCl_4_, 78 °C, 1 h, 46%; (c) 4-chloro-7*H*-pyrrolo­[2,3-*d*]­pyrimidin-2-amine, K_2_CO_3_, acetone, 40 °C, overnight, 58%; (d) TFE, 80 °C, 24 h, 52%; (e) Cs_2_CO_3_, dry ACN, 75 °C, 5 h, 20%; (f) LiOH, 1,4-dioxane/water 1:1 (v/v), rt, overnight;^1^ and (g) NH_2_OTMS, HOBt, EDCI, dry DMF, rt, overnight, 63%. Note: ^1^completion of the reaction was confirmed by LC–MS, the product was not isolated, and the crude was used without any further purification.

#### Synthesis of Hsp90 “Outer” Subpocket Binders

The synthesis of compounds **15–17** was achieved through a 7-step synthetic pathway, as illustrated in Scheme 4.^33^ Aldehyde **41** was obtained by reacting ethyl 4-iodobenzoate with allyl alcohol in a Pd-catalyzed Heck-type reaction. After converting **41** to **42** through introduction of iodine in α-position in the presence of NIS, the latter was cyclized with the commercially available 2,4-diamino-6-hydroxypyrimidine to obtain **43** in moderate yield. Insertion of chlorine at position 4 was achieved by reacting SD1 with POCl_3_ to produce **44**, unfortunately with low yields. With the central scaffold in hand, a similar pathway was followed to obtain the final products **15–17** via a first alkylation step with the appropriate benzyl fragments (**45–47**) and a final DMAP/EDCI-mediated amide coupling.

**Scheme 4:**
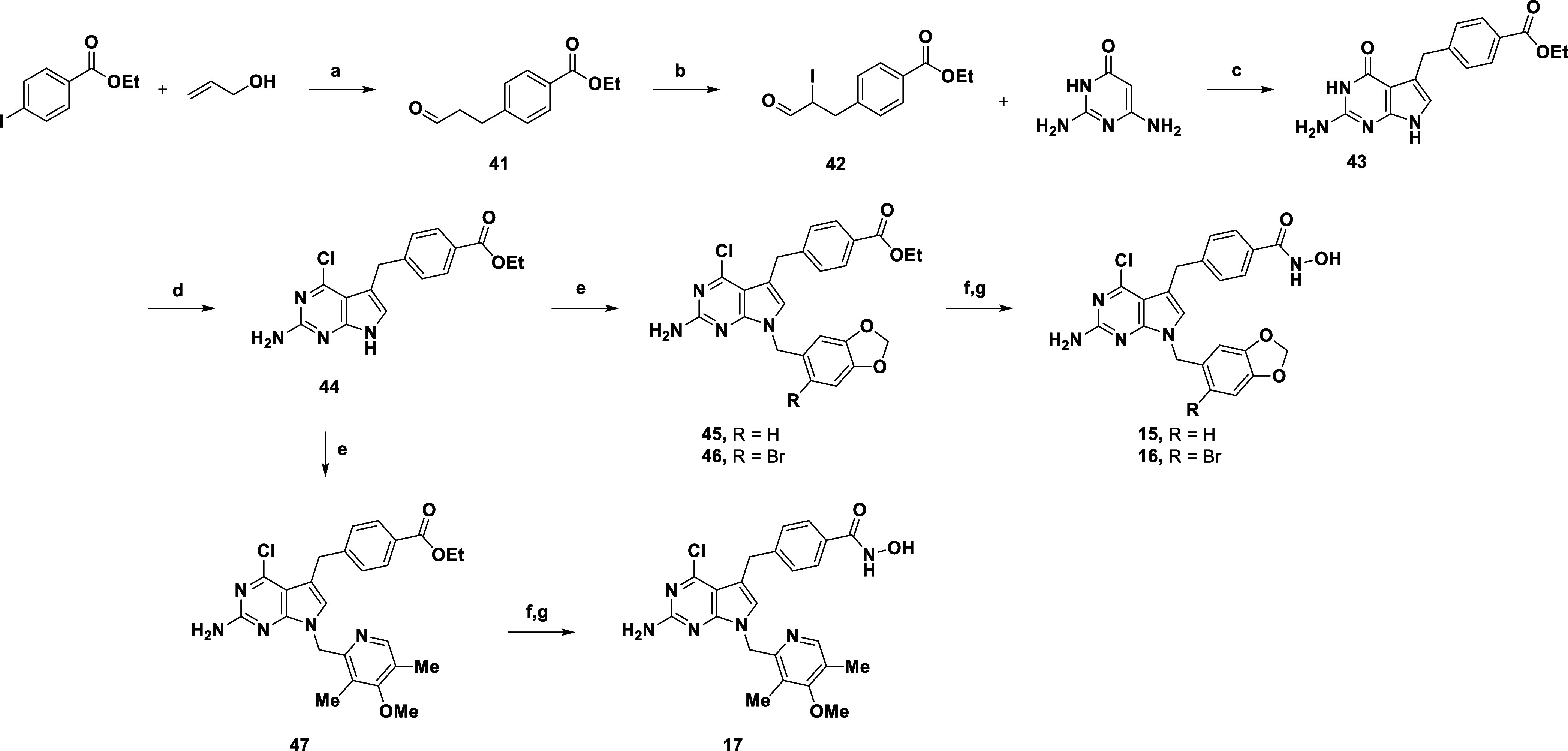
Synthesis of **15–17** ^a^

a Reagents and conditions: (a) Pd­(OAc)_2_, NaHCO_3_, TBAB, dry DMF, 3 h, 80 °C, 67%; (b) NIS, l-proline, dry DCM, rt, 10 min, 88%; (c) AcONa, water/acetonitrile 1:1 (v/v), rt, overnight, 60%; (d) POCl_3_, dry acetonitrile, 100 °C, 4 h, 23%; (e) appropriate halide, K_2_CO_3_, dry DMF, rt, overnight, 52–87%; (f) LiOH, 1,4-dioxane/water 1:1 (v/v), rt, overnight;^1^ and (g) NH_2_OTMS, DMAP, EDCI, dry DMF, rt, overnight, 25–51%. Note: ^1^completion of the reaction was confirmed by LC–MS, the product was not isolated, and the crude was used without any further purification.

### Structure–Activity Relationships

We evaluated the in vitro inhibitory activity of compounds **2**–**17** against both human Hsp90 and HDAC6 recombinant enzymes (Table 1). Among the binders of the inner pocket, the phenyl-hydroxamates **2**–**5** (Figure 5, panel A) and the thiophene-hydroxamates **6–9** (Figure 5, panel B) were investigated, considering the replacement of the purine N-7 with a CH unit and the introduction of a chlorine substituent at position 2 of the phenyl ring. Compounds **2**, **8**, and **9** were found to be inactive against Hsp90. However, compounds **3–6** (Figure 5, panel A) showed modest inhibitory activity, with their IC_50_ values ranging from 30 to 80 μM. The thiophene-bearing compound **7** emerged as the most active of this series, with an IC_50_ of 0.88 μM. The activity of **7** is favored by the presence of the 2-chloro substituent, which maximizes hydrophobic interactions with Thr104 and Val150 (Figure 5, panel B). In contrast, the halogen-free derivative **6** is more than 50-fold less potent compared to **7**. Furthermore, the activities of compounds **7** and **9** of the thiophene series suggest that replacing N-7 with a carbon atom improves Hsp90 inhibitory activity.

**Figure 5:**
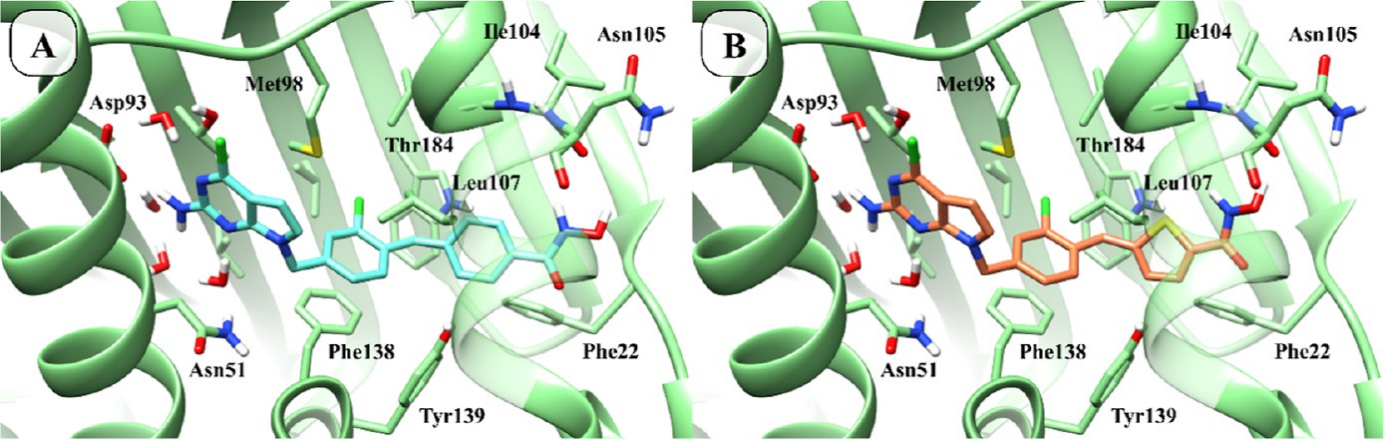
Mode of binding predicted for the compounds **3** (panel A) and **7** (panel B) within the Hsp90 binding pocket (PDB ID: 5H22).^33^

In the phenyl-hydroxamates series, introducing a chlorine substituent resulted in a less pronounced effect. Consistent with this observation, derivative **4** showed a slightly lower IC_50_ compared to that of the chlorinated compound **5**. However, compound **3**, which is also substituted with a chlorine atom, proved to be the most active among the four phenyl-hydroxamates here explored. These findings are consistent with previously reported SARs on 2-amino-pyrrolo­[2,3-*d*]­pyrimidine derivatives.^37,40^

In vitro assays on HDAC6 resulted in IC_50_ values in the nanomolar range for compounds **2–9**. Interestingly, the phenyl- and thiophene-substituted hydroxamates showed similar HDAC6 inhibitory activities, despite having different coordination geometries of the catalytic zinc ion. According to docking results, phenyl hydroxamate derivatives are predicted to preferentially bind the Zn^2+^ ion of HDAC6 through monodentate coordination (Figure 6, panel A), in agreement with literature data.^41^ Conversely, five-membered heterocyclic compounds are predicted to preferentially coordinate the Zn^2+^ ion in a bidentate fashion (Figure 6, panel B), comparable to various crystallographic structures of ligand complexes with similar linkers.^42^ Despite the different Zn^2+^ coordination patterns of the phenyl- and five-membered heterocyclic hydroxamates, the predicted binding modes of **3** and **7** were similar. The aromatic linker is sandwiched between Phe620 and Phe680 of HDAC6, while the rest of the molecule accommodates in a pocket on the external protein surface lined by residues Asp497, Trp496, His500, Ser563, and Ser564. Moreover, the 2-amino substituent establishes hydrogen bonds with the side chain of Asp497.

**Figure 6:**
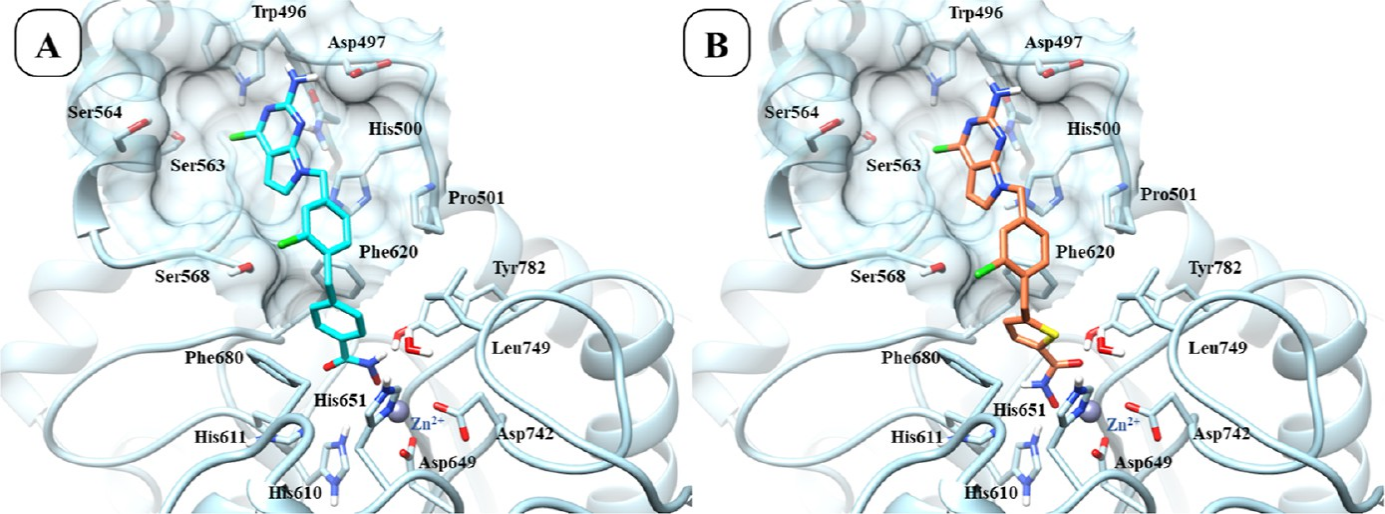
Mode of binding predicted for the compounds **3** (panel A) and **7** (panel B) within the HDAC6 binding pocket (PDB ID: 5EDU).^41^

According to the in vitro assay results, pyrrolo-pyrimidine derivatives **6** and **7** exhibited higher HDAC6 inhibitory activity compared to the corresponding purines **8** and **9** (Table 1). We then investigated the effect of substituting the methyl spacer between the phenyl and aryl-hydroxamate groups with a –NHCH_2_ group (compounds **10–13** in Table 1). This structural modification led to compounds with no Hsp90 inhibitory activity and reduced activity against HDAC6. Unfortunately, attempts to prepare derivatives bearing the desired –CH_2_NH spacer, which could have provided higher complementarity with the Tyr139 residue of Hsp90, failed. Consistent with the literature data, results from in vitro assays highlighted a different complementarity of the explored linkers to the catalytic tunnel of HDAC6. In the –NHCH_2_ derivatives, the phenyl-hydroxamate **13** (Figure 7, panel A) proved to be the most active of the series, with an IC_50_ of 173 nM. The oxazole (**10**), thiophene (**11**), and thiazole (**12**) derivatives showed HDAC6 IC_50_ values of 9.2, 1.2, and 0.97 μM, respectively.

**Figure 7:**
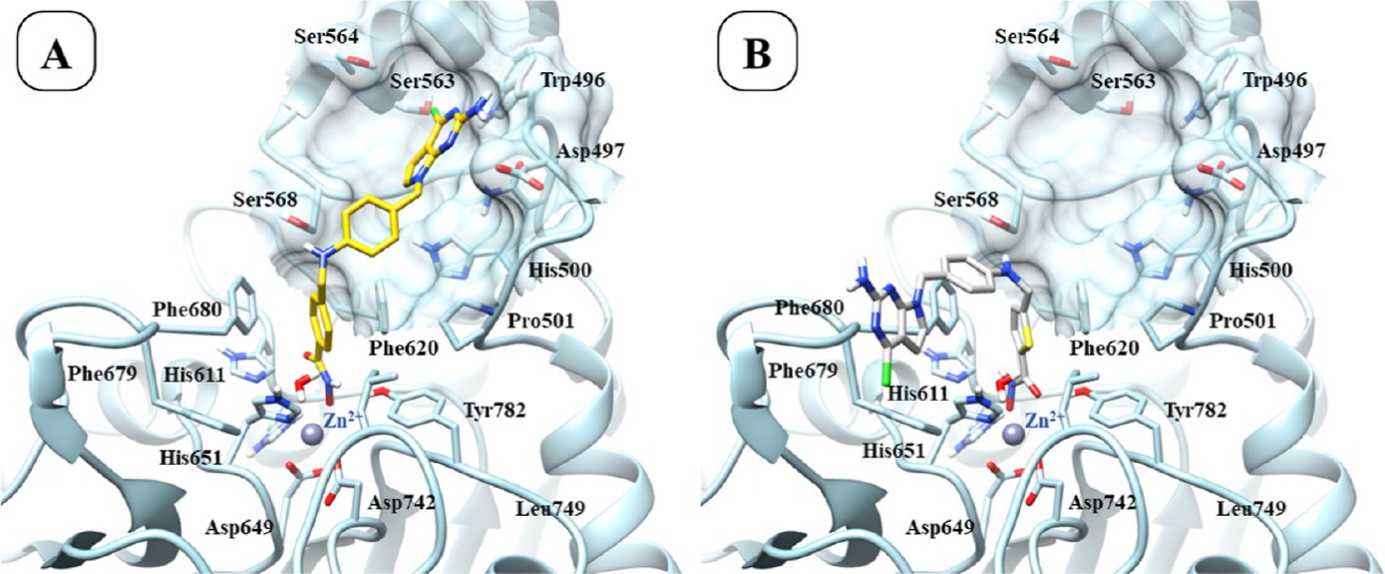
Predicted binding mode of compounds **13** (panel A) and **11** (panel B) within the HDAC6 binding pocket (PDB ID: 5EDU).^41^

According to docking results, compound **11** binds HDAC6 with a slightly different binding mode if compared with the other inhibitors identified in this study (i.e., **3**, **7**, and **13**). Of note, this difference is reflected in their experimentally observed activity (Table 1). The docking results suggest that the lower activity of **11**, along with similar compounds **10** and **12**, may be due to the lack of interactions of the 2-amino-pyrrolo­[2,3-*d*]­pyrimidine in the cavity surrounded by Asp497, Trp496, and Ser563 (Figure 7, panel B).

As for the second series of compounds designed to target the outer Hsp90 pocket, we synthesized and tested three additional 5,7-disubstituted derivatives. Remarkably, compound **17** showed potent Hsp90 inhibitory activity (IC_50_ = 96 nM), comparable to that of the Hsp90 inhibitor 7FT (PDB ID: 5H22, IC_50_ = 36 nM).^33^ Docking results suggest that the phenyl hydroxamate ring of **17** binds to the outer surface pocket of Hsp90, and the hydroxamate group hydrogen bonds with the backbone of Asp54 (Figure 8, panel A). Compound **17** was also very potent on HDAC6, with an IC_50_ of 28 nM.

**Figure 8:**
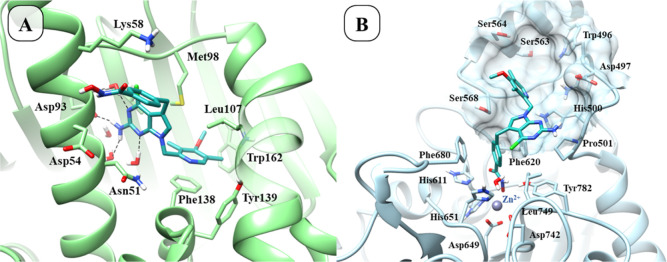
Predicted binding mode of compounds **17** in the Hsp90 (panel A, PDB ID: 5H22)^33^ and HDAC6 (panel B, PDB ID: 5EDU)^41^ binding sites.

Replacing the 4-methoxy-3,5-dimethylpyridine of **17** with a benzodioxolane group (**15**) led to an approximately 30-fold decrease in activity (Table 1). Moreover, introducing a bromine substituent on the benzodioxolane resulted in a compound (**16**) showing similar activity. As for HDAC6, compounds **15**–**17** potently inhibited this enzyme, with IC_50_ values in the nanomolar range. According to the predicted docking pose, **17** coordinates the Zn^2+^ ion in a monodentate fashion, positioning the 2-amino-7*H*-pyrrolo­[2,3-*d*]­pyrimidine between Phe620 and Pro501 (Figure 8, panel B), while the substituted pyridine moiety extends toward Ile569 and hydrogen bonds to His500.

In conclusion, the design strategies employed in this work enabled the obtainment of dual inhibitors of HDAC6 and Hsp90 by targeting both the inner and the outer subpockets of Hsp90. The crystal structure requirements were finely exploited to create dual inhibitors with the lowest possible molecular weight using an integrated pharmacophoric approach supported by docking. This approach contrasts with others, which produced chimeric compounds by connecting HDAC and Hsp90 inhibitors with flexible linkers. Notably, compounds **7** from the 7-substituted series (targeting the inner subpocket) and **17** from the 5,7-disubstituted series (targeting the outer subpocket) stand as the more active and promising dual inhibitors identified in this study. Therefore, both compounds were tested against all HDAC isoforms to assess their selectivity profiles.

### Selectivity Profiling of Compounds **7** and **17**

Compounds **7** and **17** were selected for evaluation of their selectivity on the different HDACs, as described in the Experimental Methods section. The results of this profiling are reported in Table 2. Compound **7** is 3- to 230-fold more potent against HDAC6 compared to class I HDACs and 16- to 100-fold more potent compared to class IIa HDACs. It shows similar activity against class IV HDAC11 and is 5 times more potent on class IIb HDAC10 than to HDAC6. Compound **17** is 107- to 430-fold more potent on HDAC6 compared to class I HDACs and 18- to 96-fold more potent compared to class IIa HDACs. The compound is also 4 times more active on HDAC6 than HDAC11 and 29 times more active than HDAC10. Overall, the selectivity profile of **7** is clearly in favor of HDAC6, with the exception of HDAC10 and HDAC11, whereas **17** is completely selective for HDAC6.

**Table 2:** In Vitro Inhibitory Activity (IC_50_, μM) of the More Interesting Dual Inhibitors **7** and **17** against All Class I, IIa, IIb, and IV HDACs^a^

class I				
Compound	HDAC1	HDAC2	HDAC3	HDAC8
7	0.472	1.39	0.787	34
17	3.0	5.5	3.6	12
trichostatin A	0.001	0.005	0.002	1.0

class IIa				
Compound	HDAC4	HDAC5	HDAC7	HDAC9
7	3.0	2.4	7.1	15
17	2.3	2.7	0.5	1.3
TMP269	0.122	0.135	0.057	0.013

class IIb		
Compound	HDAC6	HDAC10
7	0.084	0.029
17	0.028	0.8
trichostatin A	0.001	
quisinostat		0.005

class IV	
Compound	HDAC11
7	0.103
17	0.122
trichostatin A	2.0

a IC_50_ values of the control compounds trichostatin A, TMP269, and quisinostat are also reported. Dose–response curves of compounds **7** and **17** on the different histone deacetylases are reported in Figure S3.

### Antiproliferative Activity on PC Cells

To evaluate the potential antitumor activity of the synthesized compounds, we performed dose–response experiments on androgen-unresponsive, highly aggressive (PC3), and moderately metastatic (DU145) cells and on androgen-responsive, low metastatic LNCaP PC cells after 72 h of treatment. The HDAC6 inhibitor tubastatin A and the Hsp90 inhibitor geldanamycin were used as reference compounds.

Cancer cell viability results (Table 3) indicate that compounds **9**, **10**, and **11** failed to reduce the proliferation of PC cells, in line with the low anti-HDAC6 and absent anti-Hsp90 activities observed in vitro on recombinant proteins. **4**, **5**, and **8** were active at high concentrations (GI_50_ > 150 μM) in PC3 and DU145 cells, while **12** and **14** were only moderately active in PC3 and LNCaP. Notably, seven compounds (marked in bold in Table 3) demonstrated cell growth inhibitory activities across all tested PC cell lines, with GI_50_ values below 100 μM. These included two phenyl-hydroxamates (**2** and **3**), two thiophene-hydroxamates (**6** and **7**), and the three 5,7-disubstituted derivatives (**15**, **16**, and **17**). In line with the in vitro enzyme inhibition data, compounds **7** and **17** clearly emerged as the most potent also in reducing PC cell viability, with **17** being considerably more active than our lead compound **1**, achieving low micromolar GI_50_ values across the three cell lines tested.

**Table 3:** Antiproliferative Activities (GI_50_ ± SEM, μM) of the Synthesized Compounds in LNCaP, PC3, and DU145 Cancer Cells Treated for 72 h^a^

Compound	GI_50_ LNCaP (μM)	GI_50_ PC3 (μM)	GI_50_ DU145 (μM)
1	5.8 ± 1.4	14.1 ± 3.7	12.8 ± 2.6
**2**	**41.0 ± 7.9**	**19.1 ± 0.8**	**31.0 ± 5.1**
**3**	**30.2 ± 8.2**	**11.1 ± 1.4**	**28.1 ± 4.0**
4	n.a.	166.1 ± 3.3	170.7 ± 7.2
5	n.a.	150.9 ± 5.7	168.7 ± 8.0
**6**	**55.6 ± 8.3**	**31.7 ± 6.5**	**71.5 ± 11.7**
**7**	**8.1 ± 0.4**	**7.1 ± 3.5**	**42.6 ± 8.1**
8	n.a.	150.7 ± 8.9	157.2 ± 8.7
9	n.a.	n.a.	n.a.
10	n.a.	n.a.	n.a.
11	n.a.	n.a.	n.a.
12	59.9 ± 17.5	160.6 ± 12.2	n.a.
13	n.t.	n.t.	n.t.
14	22.6 ± 5.8	55.4 ± 3.5	n.a.
**15**	**42.6 ± 10.3**	**27.0 ± 5.3**	**41.3 ± 4.5**
**16**	**39.5 ± 11.6**	**25.4 ± 2.7**	**24.4 ± 4.3**
**17**	0.7 ± 0.1	2.2 ± 0.6	0.8 ± 0.2
tubastatin A	5.9 ± 1.5	11.1 ± 1.6	6.7 ± 2.4
geldanamycin	0.05 ± 0.02	0.03 ± 0.02	0.03 ± 0.01

a Tubastatin A and geldanamycin were used as positive controls. Dose–response curves are reported in Figures S4 (LNCaP), S5 (PC3), and S6 (DU145). Note: n.a: not active, compounds not characterized by dose–response cellular effects or by GI_50_ ≥ 200 μM. n.t.: not tested.

The seven compounds with GI_50_ values ≤100 μM in all three cell lines (marked in bold in Table 3) were selected for further investigation, as described below.

### Inhibition of HDAC6 and Hsp90 in PC Cells

The most promising compounds were initially analyzed by Western blotting (WB) extracts from PC3 cells after 24 h treatment at GI_50_ doses (Figure 9) to assess whether their antiproliferative activity observed at 72 h could be a consequence of the specific inhibition of HDAC6 and Hsp90. The HDAC6 inhibitor tubastatin A, the Hsp90 inhibitor geldanamycin, and the lead compound **1** (which exclusively targets HDAC6) were used as reference compounds.

**Figure 9:**
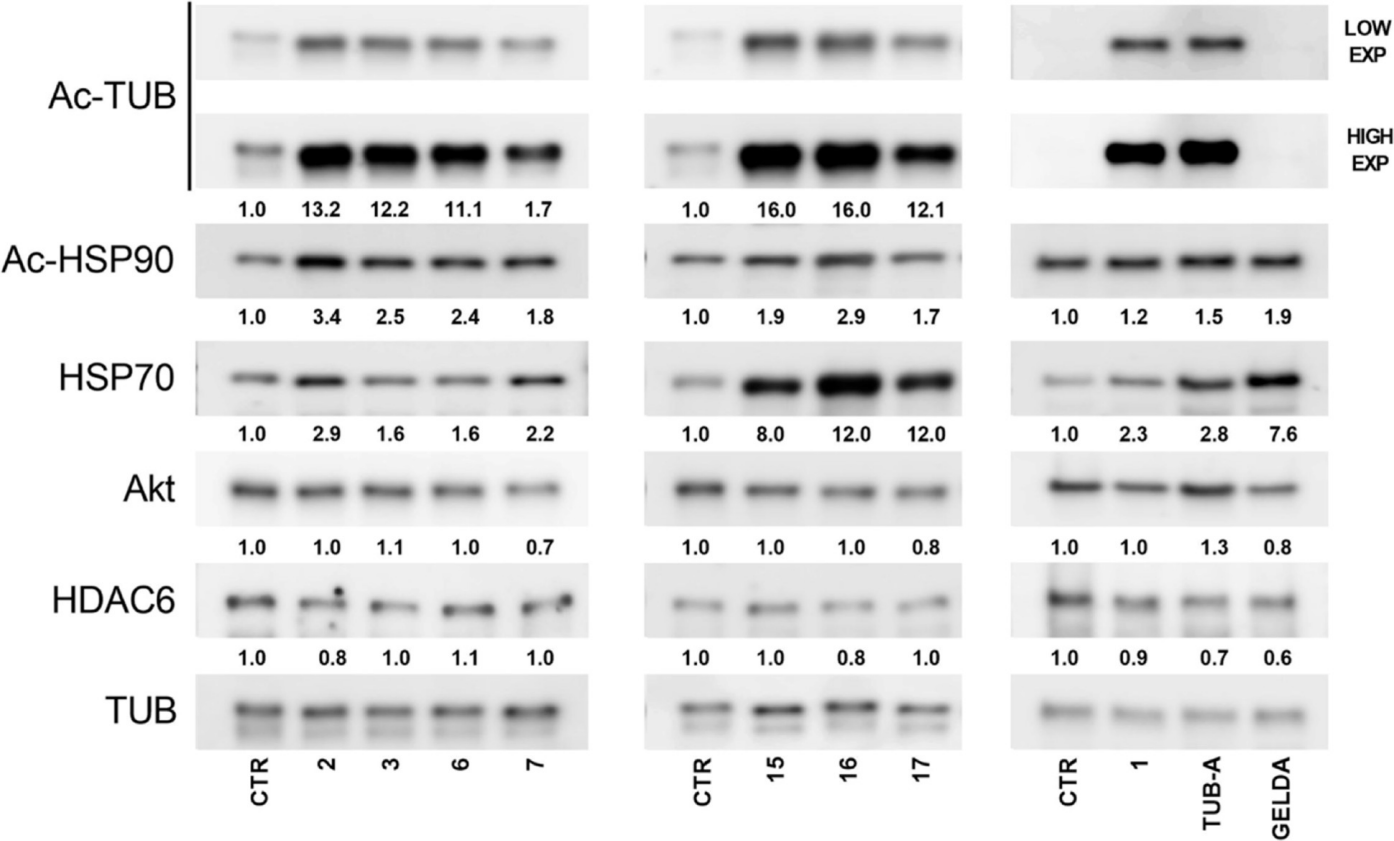
Levels of expression of acetylated-Tubulin (Ac-TUB), acetylated-Hsp90 (Ac-HSP90), Hsp70, Akt, and HDAC6 in PC3 cells treated for 24 h at the GI_50_ doses, as evaluated by WB analysis. Tubastatin A and geldanamycin were used as HDAC6- and Hsp90-specific positive controls, respectively. Tubulin (TUB) was used as loading controls. Quantification is reported in terms of fold change in treated cells vs DMSO (CTR), which was set to 1. Fold change represents the mean of three independent experiments.

Ac-TUB expression levels were substantially increased by all tested compounds, although to different extents, except for geldanamycin. Tubulin was used as loading control in these assays. Given that HDAC6 is a known substrate of Hsp90 and that Hsp90 inhibition leads to degradation of HDAC6,^16^ we next assessed the total HDAC6 protein level. Western blot showed that only **2** and **16** caused a slight reduction in HDAC6, suggesting that the observed effects on Ac-TUB are likely due to enzyme inhibition rather than reduced HDAC6 expression. Since HDAC6 inhibition can lead to Hsp90 acetylation, with disruption of its chaperone functions, we evaluated the levels of acetylated-Hsp90, which increased following treatment with all compounds, and mainly by compound **2**.

Because the inhibition of Hsp90 causes a pronounced induction of its cochaperone Hsp70, measuring Hsp70 levels can help determine the potency of Hsp90 inhibitors. Therefore, we examined the Hsp70 protein levels after treatment with the selected compounds. Western blots in Figure 9 highlight a remarkable induction of Hsp70 after incubation with the three 5,7-disubstituted derivatives (**15, 16, and 17**) and geldanamycin, while moderate activation is observed with **2** and **7**, to levels comparable to those of **1** and tubastatin A. Additionally, only cell exposure to **7**, **17**, and geldanamycin downregulated the protein levels of the Hsp90 client protein Akt.

Given the complex regulatory interplay between HDAC6 and Hsp90, we performed Western blot analyses 24 h after treating PC3 cells with compound concentrations of 1 μM and 10 μM to gain insights into their potency on cellular targets. Notably, 1 μM is below the GI_50_ value for all compounds except geldanamycin, while 10 μM is below or near the GI_50_ for all compounds except compound **17** and geldanamycin. Unfortunately, 10 μM geldanamycin could not be included in the Western blot analysis due to excessive cell mortality. Western blot analysis in Figure 10 (panel A) shows that compounds **2**, **3**, **6**, and **17** maintain strong HDAC6-inhibitory activity after treatment at 1 μM, while **15** and **16** also exhibit inhibitory effect, though to a lesser extent. Only compound **17** induced an increase of Hsp70 to levels comparable to those of geldanamycin, with minor changes in Ac-Hsp90 expression levels, demonstrating concomitant inhibitory activity on both HDAC6 and Hsp90. When the concentration was increased to 10 μM (Figure 10, panel B), a dose-dependent effect on Ac-TUB levels was observed for all synthesized compounds, with HDAC6 inhibition also evident for compound **7**. Additionally, Hsp70 was further upregulated by **17** and induced by **2**, **3**, **7**, **15**, and **16**. To evaluate potential off-target effects on nuclear HDACs, we performed Western blot analysis using an antiacetyl histone H3 antibody, a well-established marker of nuclear HDACs inhibition (Figures S7 panels A and B). Cells were treated for 24 h with each of the tested compounds at both 1 μM and 10 μM concentrations, following the same protocol of Figure 10. Only the positive control SAHA resulted in a clear increase in histone H3 acetylation levels, while none of the synthesized compounds produced detectable acetylation of histone H3 at both concentrations. Taken together, Western blot analyses performed at GI_50_ (Figure 9), 1 μM (Figure 10, panel A), and 10 μM (Figure 10, panel B) concentrations indicate that all the tested compounds can modulate HDAC6 activity. In agreement with the in vitro enzyme inhibition results, the three 5,7-disubstituted derivatives targeting the outer subpocket of Hsp90 (**15**, **16**, and **17**) showed dual activity on both HDAC6 and Hsp90 targets, along with the thiophene-hydroxamate **7** of the inner subpocket, although to a lesser extent. Compound **17** stands out as the best compound based on both in vitro and cellular assays. As a further positive control of the molecular efficacy of our dual targeting approach, we coadministered geldanamycin and tubastatin A and performed Western blot analysis. The combination at their respective GI_50_ concentrations resulted in high toxicity, cell viability being reduced to approximately 20% (Figure S7 panel C). Therefore, geldanamycin and tubastatin A were simultaneously administered at either 1/4 or 1/10 of their respective GI_50_ concentrations, which led to 36% and 75% cell viability, respectively. Western blot analyses using anti-Hsp70 and anti-Ac-TUB antibodies (Figure S7 panel D) confirmed effective dual target engagement in a dose-dependent manner and the higher efficacy of compound **17**.

**Figure 10:**
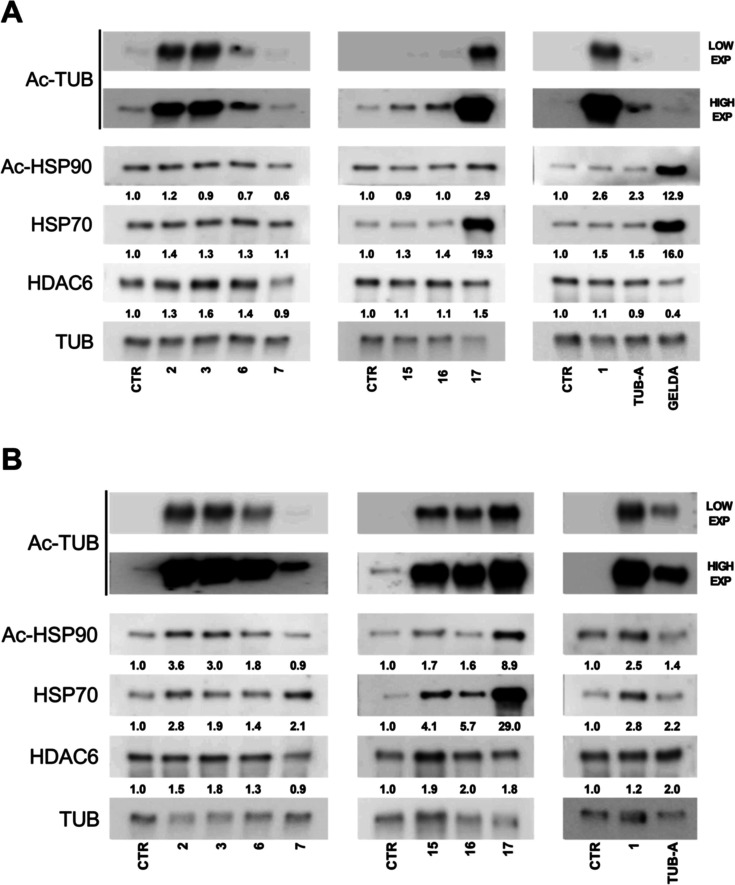
Expression levels of acetylated-Tubulin, acetylated-Hsp90, Hsp70, and HDAC6 in PC3 cells treated for 24 h at 1 μM dose (panel A) and 10 μM dose (panel B), as evaluated by WB analyses. TUB-A and GELDA were used as HDAC6- and Hsp90-specific positive controls, respectively. Tubulin was used as a loading control. Quantification is reported in terms of fold change in treated cells vs DMSO (CTR), which was set to 1. Fold change represents the mean of three independent experiments.

### Effect on Cell Cycle Distribution after Treatment of PC Cells

The four compounds identified as the most promising dual inhibitors in previous analyses (compounds **7** and **15–17**) were further investigated. We evaluated the cell cycle distribution by flow cytometry, through Propidium Iodide (PI) staining of DNA content, following treatment with the compounds at GI_50_ doses for 72 h in PC3 cells. As shown in Figure 11, administration of compound **17** led to a notable increase in the G2/M population, rising from 19.3% in control cells (DMSO) to 35.3% in treated cells, with a concomitant reduction of the S phase from 14% in control cells to 5.6% in treated cells.

**Figure 11:**
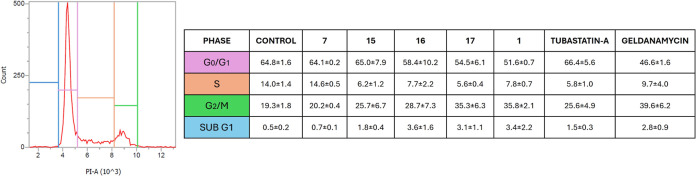
Effects of 72 h treatment with compounds **7** and **15**–**17** at their GI_50_ concentration on cell cycle progression in PC3 cells. Compound **1**, tubastatin A, and geldanamycin were used as reference controls. The table shows the mean ± SEM of three independent experiments of the distribution (%) of PC3 cells across different cell cycle phases. Cells were incubated for 72 h at the GI_50_ concentration of each compound. PC3 cells treated with DMSO alone were used as the control. A representative flow cytometry histogram of DNA content in the different phases of the cell cycle in cultures of PC3 cells is shown in the left panel.

Compound **17** also induced a modest increase in SubG1 events, indicative of cell death, from 0.5% in control cells to 3.1% in treated cells. Similarly, **1** and geldanamycin induced an accumulation of cells in the G2/M phase, with a reduction of replicating S-phase cells and a mild increase in SubG1 events to approximately 3%. Compound **16** also caused an increase in SubG1 events to 3.6%; however, its effects on cell cycle arrest were less pronounced, with G2/M rising from 19.3% in control cells to 28.7%. Treatment with compound **15** or tubastatin A had a milder effect on the cell cycle, reducing the S-phase population by half and increasing SubG1 events from 0.5% in control cells to 1.8% and 1.5%, respectively. Unexpectedly, cells exposed to the GI_50_ concentration of **7** did not exhibit significant changes in the distribution of cell cycle phases. This suggests that the compound may generally slow down the growth of PC3 cells or that its effects are transient, allowing cells to resume normal proliferation after the inhibitory phase. In conclusion, cell cycle cytofluorimetric analysis highlights compound **17** as the most effective cytostatic and moderately cytotoxic compound.

### Inhibitory Effect of Compound **17** on PC3 Tumor Spheroids

The promising antiproliferative activity of compound **17** observed in PC3 cells was further validated in tumor spheroids generated from multicellular cell aggregates (Multicellular Tumor Spheroid, MTS) or from single cells (single cell-derived Tumor Spheroid, sc-TS). These spheroids differ in cellular arrangement, extracellular matrix (ECM) production, and drug response, making them valuable for modeling tumor dynamics.

MTSs generated through multiple cell aggregation in 0.25 mg/mL Matrigel^43^ were treated with 10 μM of compound **17** or DMSO (control), and their morphology was monitored daily using microscopy (Figure 12, panel A). The relative spheroid area, normalized to day 0, was tracked over 7 days by measuring the total diameter (schematically represented in red in Figure 12, panel A). From day 5, untreated spheroids exhibited a more than 3-fold increase in relative area, while treated spheroids remained stable in size, as a result of drug-induced antiproliferative effects (Figure 12, panel B). MTT assay on day 7 further confirmed reduced cell viability in treated spheroids (56% ± 10) compared to untreated controls (100%) (Figure 12, panel C).

**Figure 12:**
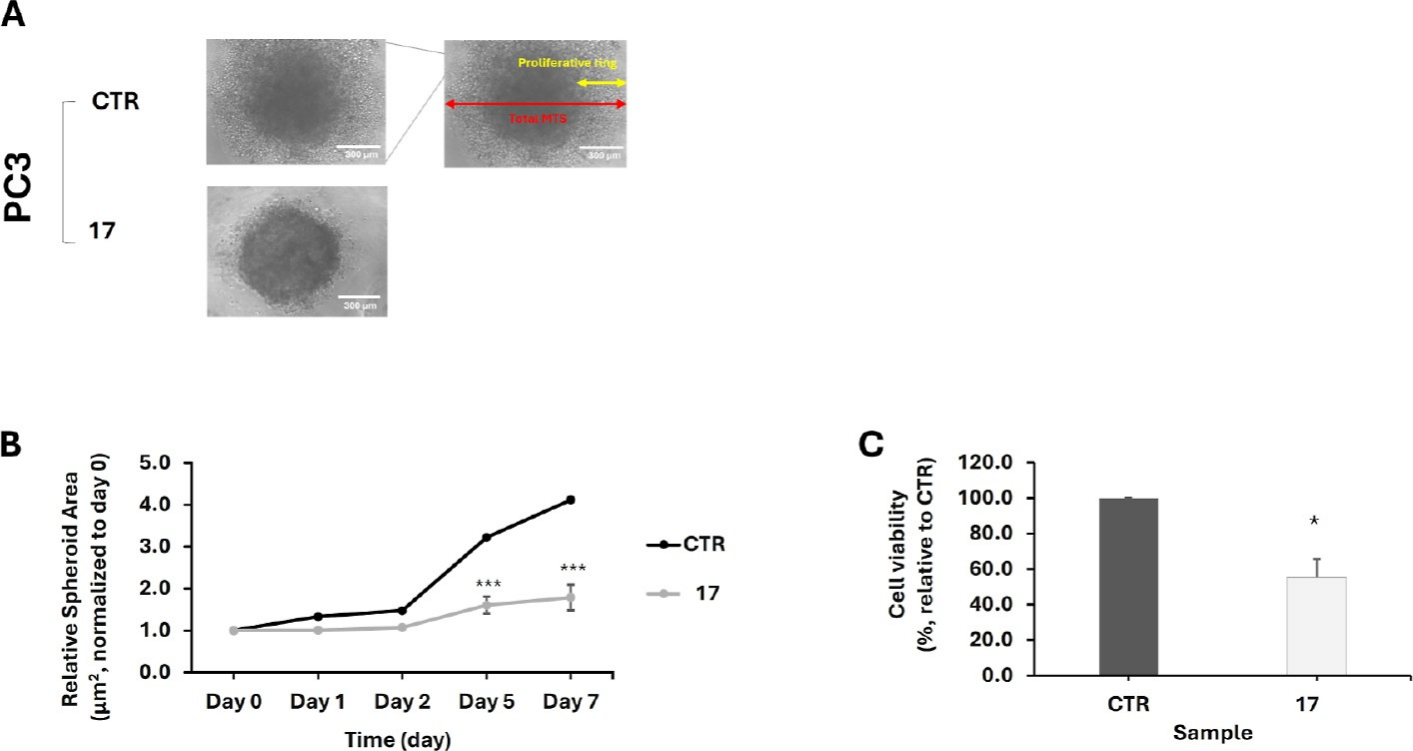
Effect of treating PC3 multicellular tumor spheroids (MTSs) with compound **17**. MTSs were treated with the compound at 10 μM dose and monitored daily from day 0 (immediately post-treatment) to day 7. The control (CTR) refers to MTSs treated only with DMSO. (A) Representative phase contrast microscopy images of PC3MTSs at day 7, either untreated (CTR) or treated with compound **17**. Scale bar = 300 μm. (B) Relative area (normalized to day 0) of both treated and untreated MTSs, determined daily from day 0 to day 7 by measuring the total diameter (schematically represented in A, in red). (C) Effect of compound **17** on MTS cell viability, determined by MTT assay at day 7. Data represents mean ± St.Dev (one sample *t*-test for the treated group vs CTR group, *n* = 3). Asterisks indicate statistical significance between the treated and CTR groups: (*p < 0.05 and ***p < 0.001).

Given its marked antiproliferative activity in MTSs, compound **17** was further tested on PC3-derived sc-TSs, which originate from single-cell suspensions, to examine its impact on the ability of single cells to survive, proliferate, and form spheroids over time. For this purpose, a 10 μM concentration was selected for both **17** and its precursor **1** for comparison, and sc-TSs were monitored for over 6 days. Additionally, tubastatin A (10 μM) and geldanamycin (10 μM or 1 μM) were used as reference inhibitors of HDAC6 and Hsp90, respectively. To mimic the dual inhibitory effect on Hsp90 and HDAC6, geldanamycin and tubastatin A were also coadministrated in combinations of 1 + 10 μM and 10 + 10 μM, respectively. The combined use of these two inhibitors provided insights into the dual inhibition mechanism, demonstrating varying degrees of effectiveness depending on the concentration used, with geldanamycin showing the highest antiproliferative effect. Phase-contrast images (Figure 13) and histograms obtained from MTT data (Figure 14A–E, panel A) indicate that compound **17** had remarkable effects on PC3 sc-TSs viability over time compared to compound **1**, further supporting the advantage of administering a dual inhibitor instead of our single-target starting compound. The antiproliferative effects of compound **17** were comparable to those obtained with the combination of geldanamycin and tubastatin A, two of the most effective single-target inhibitors developed so far.

**Figure 13:**
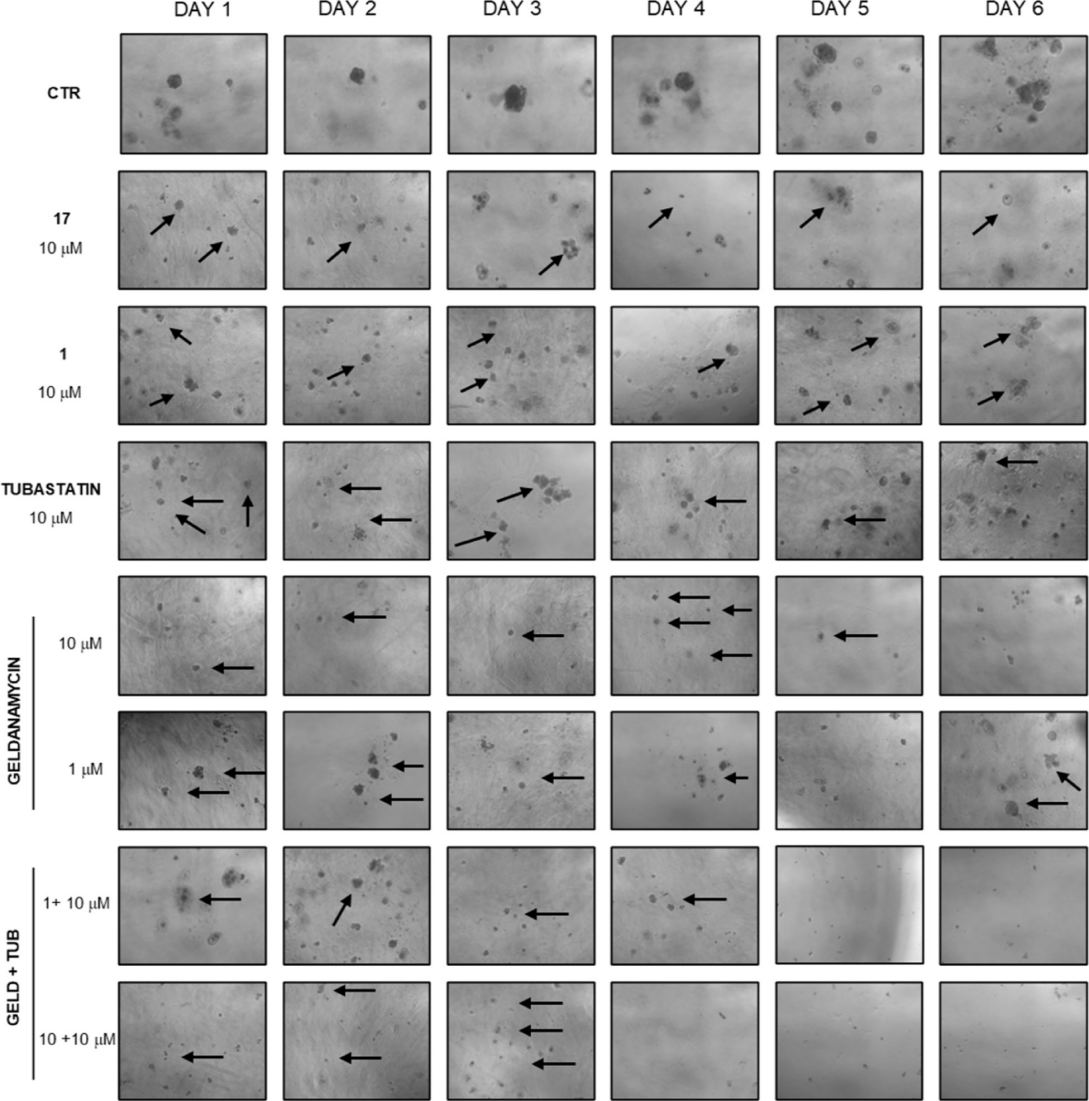
Representative images of PC3 spheroids generated from single cells (sc-TSs) captured by light microscopy (magnification 10×) over 6 days of treatment; compounds **17**, **1**, and tubastatin A were used at 10 μM; geldanamycin was used at 10 or 1 μM; geldanamycin + tubastatin A were coadministered at (1 + 10) μM and (10 + 10) μM concentrations, respectively.

**Figure 14:**
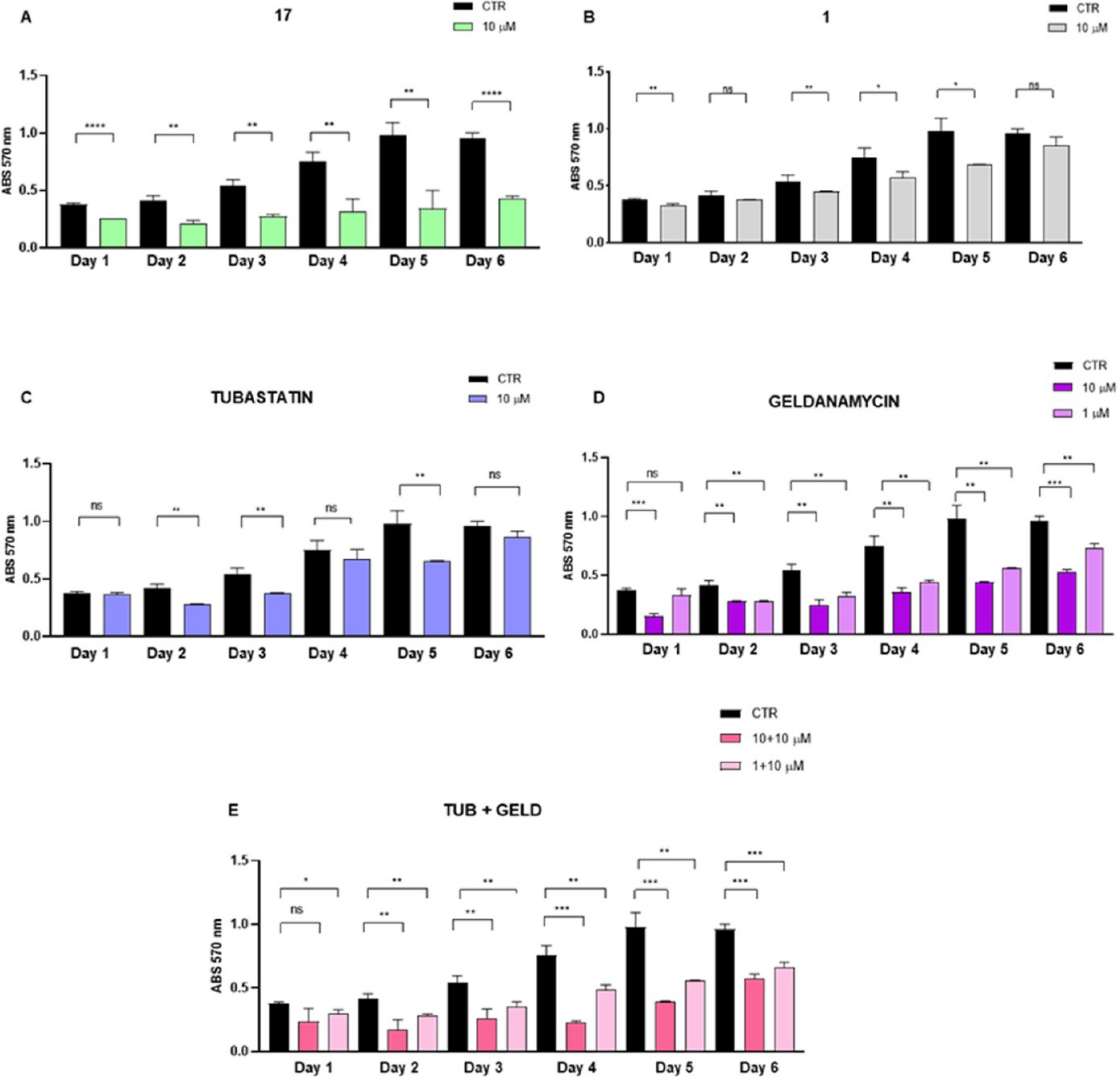
Viability assay results performed on PC3 spheroids generated by single cells (sc-TSs) over 6 days by using MTT. The compounds were used at a final concentration as follows: compounds **17** (panel A), **1** (panel B), and tubastatin A (panel C) were used at 10 μM; geldanamycin (panel D) was used at 10 or 1 μM; and geldanamycin + tubastatin A (panel E) were coadministered at (1 + 10) μM and (10 + 10) μM concentrations, respectively.

Altogether, these results demonstrate that compound **17** significantly decreases the viability of prostate tumor cell 3D cultures over time, leading to a marked reduction in size (MTSs) and number (sc-TSs) compared to the control.

### Combination Studies of Compound **17** with Tubastatin A, Geldanamycin, and Doxorubicin

The effectiveness of drug combinations depends on the biological targets of the respective drugs and the network of interactions between them. To further confirm the mechanism of action of **17** and assess whether simultaneous inhibition of HDAC6 and Hsp90 enhances cell death when combined with single-target anticancer agents, PC3 cells were cultured for 72 h with compound **17**, tubastatin A, geldanamycin, and doxorubicin in various combinations. Drugs were combined in a dose–response curve spanning concentrations above and below the GI_50_ values of the individual agents, at a constant ratio as detailed in the Methods section. The combination index (CI) was calculated using the Chou-Talalay method for drug combination, with Compusyn (version 1.0) software.^44,45^ CI values < 1, = 1, or >1 indicate synergism, additivity, or antagonism, respectively.

First, coadministration of geldanamycin (Hsp90 inhibitor) and tubastatin A (HDAC6 inhibitor) demonstrated synergistic effects in PC3 cells, thereby confirming the rationale for dual Hsp90 and HDAC6 targeting. Then, we combined compound **17** with tubastatin A or geldanamycin to further confirm the molecular mechanisms underlying the activity of our dual inhibitor. The combination index CI calculated at 50% cell viability (Figure 15A) revealed antagonistic effects of **17** with either tubastatin A or geldanamycin, in agreement with the dual inhibitory activity of our best candidate. Figure 15 (panel B) shows the CI values of the experimental data points alongside the computer simulation of dose–effect drug combinations. This antagonistic effect is consistent with the competition occurring between compound **17** and known ligands for the binding to HDAC6 and Hsp90 in PC cells.

**Figure 15:**
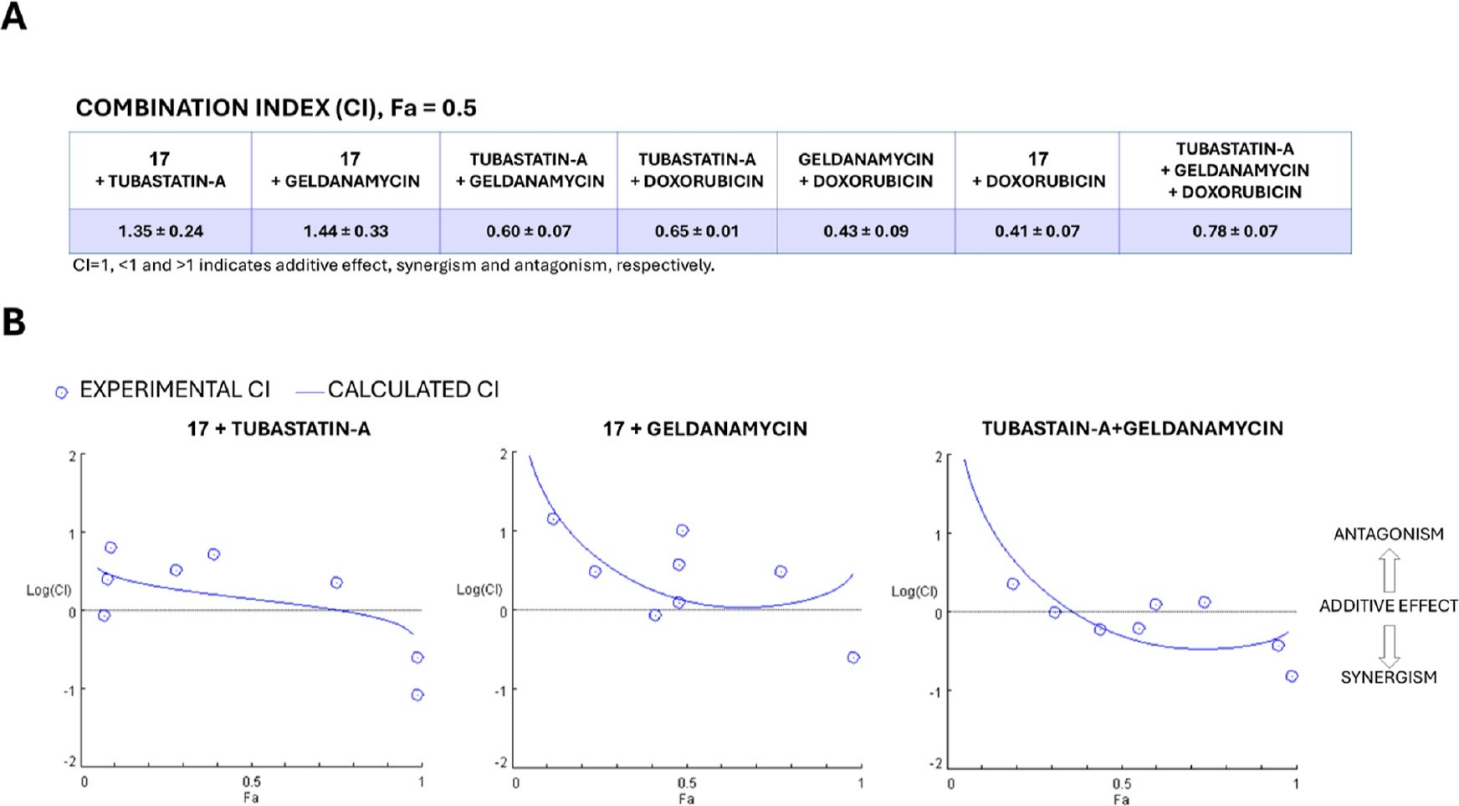
Comparison of CI values for different combinations of compounds **17**, tubastatin A, geldanamycin, and doxorubicin on PC3 cells treated for 72 h. Panel A: combination indices were calculated at Fa = 0.5 corresponding to 50% inhibition of cell viability. Drugs are classified as synergistic (CI < 1), additive (CI = 1), or antagonistic (CI > 1). Panel B: Effects of compound **17** in combination with tubastatin A and geldanamycin on PC3 cells. Cells were incubated for 72 h with the indicated compound combinations, starting at a dose of 50 μM, followed by 3-fold serial dilutions at a constant ratio of 1:1, except for geldanamycin which was maintained at a constant ratio of 1:50 (geldanamycin: compound). Fa-log­(CI) plots were generated with CompuSyn (version 1.0) software. The baseline at log­(CI) = 0 indicates an additive effect, while log­(CI) values below or above 0 indicate synergy or antagonism, respectively.

Since doxorubicin is often used in combination with other targeted anticancer drugs for treating solid tumors,^46,47^ including castration-resistant PC,^48,49^ we combined compound **17** with doxorubicin. The CI calculated from dose–response experiments indicated a marked synergistic effect (Figures 15 panel A and S8). As an additional control, doxorubicin was also combined with tubastatin A, geldanamycin, or both. Notably, the combination of **17** with doxorubicin outperformed the coadministration of tubastatin A, geldanamycin, and doxorubicin (see the CI values calculated for Fa = 0.5 in Figures 15, panel A, and S8). These findings further support the advantages and potential offered by our dual inhibitor **17**, even in combination therapies.

### In Vitro Evaluation of the Drug-like Properties of Compound **17**

Compound **17**, which exhibits the best activity on both isolated enzymes and in cells, was evaluated for its drug-like properties, which included water solubility, cell permeability, and metabolic stability.^50–52^ The results are reported in Table 4.

**Table 4:** In Vitro Drug-like Properties of Compound **17** ^a^

Compound	solubility (μM) pH 7.4	solubility (μM) pH 4.5	*P*_app_ × 10^–6^ (cm/s)^b^	intrinsic clearance^c^ (μL/min/mg protein) mouse	intrinsic clearance^d^ (μL/min/mg protein) human
**17**	12.4 ± 0.7	23.7 ± 1.1	1.3 ± 0.1	49.8 ± 1.8	32.4 ± 4.9

a The Table reports water solubility at pH 7.4 and 4.5, cellular permeability (*P*_app_), and metabolic stability (intrinsic clearance) in mouse and human liver microsomes.

b Note. *P*_app_ values: low (≤1 × 10^–6^ cm/s); medium (1–10 × 10^–6^ cm/s); and high (>10 × 10^–6^ cm/s).

c Clearance in mouse microsomes: low (≤2.5), medium (2.5–66), and high (>66).

d Clearance in human microsomes: low (≤1.8), medium (1.8–48), and high (>48).

The kinetic solubility of compound **17** at pH 7.4 and 4.5 was 12.4 and 23.7 μM, respectively. The cellular permeability was evaluated using Caco-2 cells, as a well-established model for assessing intestinal absorption. Compound **17** exhibited medium cellular permeability, with a *P*_app_ of 1.3 × 10^–6^ cm/s. The metabolic stability was determined after incubation with human and mouse liver microsomes. In human liver microsomes, compound **17** exhibited a medium intrinsic clearance of 32.4 μL/min/mg protein and a slightly lower stability in mouse (49.8 μL/min/mg protein). In conclusion, compound **17** shows favorable drug-like properties, including solubility and permeability profiles. Although its phase I metabolic stability appears suboptimal, its excellent antiproliferative activity in cells and 3D spheroids make it a promising candidate for more advanced preclinical evaluations.

## Conclusions

According to recent findings, Hsp90 and HDAC6 emerged as valuable targets for developing multitarget inhibitors, their activity being mutually involved in the regulation of a number of biological processes related to PC development and progression.^17–19^ In a previous study, we reported a series of 2-amino-pyrrolo­[2,3-*d*]­pyrimidine compounds that demonstrated potent HDAC inhibitory activity and antiproliferative effects against aggressive PC cells.^29^ The same scaffold is present in known Hsp90 inhibitors.^30^ Building on this background and using our HDAC6 inhibitor **1** as the starting point, in this article, we have structure-based designed and synthesized two new series of dual activity compounds possessing ad hoc features required for HDAC6 inhibition while targeting either an inner or an outer pocket of Hsp90. This led to the synthesis of two series of compounds, which were then submitted to biological assays. Of note, most of the tested compounds exhibited nanomolar inhibition of HDAC6, and some of them also showed marked Hsp90 inhibitory activity (Tables 1 and 2). Compounds **7** and **15–17** showed the desired Hsp90/HDAC6 dual inhibitory activity (Table 1). The compounds were tested for their antiproliferative activity on LNCaP, DU145, and PC3 PC cells, with several of them demonstrating activity across all three cell lines (Table 3). Remarkably, one compound (**17**) exhibited single-digit micromolar cellular activity against PC3 and submicromolar cellular activity against LNCaP and DU145. Notably, this compound is the most potent dual inhibitor of the series, which displayed nearly balanced nanomolar inhibition of both targets.

To further characterize the cellular effects of the compounds, we also performed Western blot analyses on PC3 extracts at their GI_50_, 1 μM, and 10 μM concentrations. These analyses confirmed that the compounds target HDAC6 and inhibit the enzyme activity in cells, triggering subsequent cellular responses as a result of altered molecular cascades caused by HDAC6 inhibition. Among them, compounds **15**, **16**, **17**, and to a lesser extent compound **7** demonstrated dual cellular activity, affecting both HDAC6 and Hsp90. Of the tested compounds, **17** emerged as the most potent inhibitor in both enzyme and cellular in vitro assays, also showing potent cytostatic and moderate cytotoxic activity in cell cycle evaluations.

Compound **17** also demonstrated marked antiproliferative properties in three-dimensional models derived from PC3 cells, closely mimicking the structural organization of tumors. These findings highlight its efficacy in targeting both fully developed, heterogeneous tumor masses (MTSs) and tumor formations enriched in tumor-initiating cells (sc-TSs), which are associated with increased metastatic potential. Furthermore, comparison between compound **17** (dual HDAC6/Hsp90 inhibitor) with its precursor **1** (HDAC6 inhibitor), tubastatin-A (HDAC6 inhibitor), and geldanamycin (Hsp90 inhibitor), both as single or combined agents, shows that compound **17** is more effective in reducing the size and number of sc-TSs. Finally, the combination of compound **17** with doxorubicin outperformed other combinations with single-target drugs.

Overall, compound **17** combines potent on-target dual HDAC6/Hsp90 activity with excellent antiproliferative activity in cells and 3D spheroids, making it a promising candidate for more advanced preclinical evaluations against aggressive forms of advanced PC.

## Experimental Methods

### Molecular Modeling

#### Protein Preparation

The docking studies on HDAC6 and Hsp90 were conducted on the crystal structures with PDB codes 5EDU^41^ and 5H22,^33^ respectively. The crystal structure of the proteins under investigation was prepared by means of the Protein Preparation Wizard tool (Schrödinger Suite 2021-1),^53^ as previously described.^29,54,55^

#### Molecular Docking

The XP protocol of Glide (Schrödinger Suite 2021-1)^56^ was used to perform molecular docking of the compounds into the prepared structures. The structure-based calculations were carried out with modalities similar to those previously described,^29,54,55^ applying a series of constraints to ensure coordination between the ligand and the catalytic Zn^2+^ ion of HDAC6. In silico models were validated prior to docking calculations by redocking the cocrystallized ligands TSN and 7FT into 5EDU and 5H22, respectively, with satisfactory results. The docked poses of the ligands designed in this study were selected after visual inspection of their complexes with Hsp90 and HDAC6.

### Chemistry

All chemical reagents and solvents necessary for the synthesis of the compounds were purchased, and used without further purification, from BLDPharm (Hamburg, Germany), Fluorochem (Hadfield, United Kingdom), Merck (Milan, Italy), TCI (Zwijndrecht, Belgium), unless otherwise stated. All reactions were monitored by means of (i) thin layer chromatography (TLC) performed on silica gel (Merck precoated 60 F_254_ plates), with UV detection at 254 nm wavelength or by permanganate for the detection of compounds, or (ii) appropriate high-performance liquid chromatography (HPLC) performed on an Agilent 1100 Series System, equipped with a 150 × 3 LC Column Gemini 5 μM C18 110 Å and with a gradient of H_2_O/ACN (+0.1% HCOOH) that ranged from 5% ACN up to 100% ACN over 40 min (flux of 1.0 mL/min and sample injection of 5 μL), 220 or 254 nm wavelengths being used for compound detection. The synthesized products were purified through flash column chromatography with a stationary phase of silica gel Merck 60 (230–400 mesh) or by reverse phase chromatography using a prepacked C18 column on an automated flash system (Selekt, Biotage). All compounds are >95% pure by HPLC analysis. ^1^H NMR and ^13^C NMR spectra were recorded at 298 K on a Brüker Avance Spectrometer (400 or 600 MHz for ^1^H NMR), using deuterated solvents (Chloroform-*d*, MeOD, DMSO-*d*_6_). Chemical shifts, which are compared to TMS as an Internal Standard (IS), are reported in parts per million (δ ppm). Coupling constants (*J*) are given in hertz (Hz) and are quoted to the nearest 0.5 Hz. Peak multiplicities are noted as s, singlet; bs, broad singlet; d, doublet; m, multiplet; and br, broad. High-resolution mass spectra (HRMS) were recorded using the Q-ToF Synapt G2-Si HDMS Acquity UPLC I-Class photodiode detector array (Waters) or a UHPLC-MS Q Exactive (Thermo Scientific).

### 4-(4-((2-Amino-4-chloro-7*H*-pyrrolo­[2,3-*d*]­pyrimidin-7-yl)­methyl)­benzyl)-*N*-hydroxybenzamide (**2**)

To a solution of **22** (0.06 g, 0.15 mmol, 1.0 equiv) in THF/MeOH 1:1, v/v (3 mL) were added NH_2_OH 50% W (2.0 mL, 30 equiv) and NaOH 1N (1.0 mL, 7.5 mmol, 30 equiv), and the reaction mixture was stirred at 25 °C for 2 h. The solvent was evaporated under vacuum, and the pH was adjusted to 4–5 with 1 N HCl. The resulting precipitate was recrystallized from MeOH/Et_2_O, after filtration and washing with water, leading to **2** (0.04 g, 33% yield) in high purity, as an amorphous white solid. ^1^H NMR (400 MHz, DMSO-*d*_6_, δ): 11.10 (s, 1H, -NH), 8.96 (br s, 1H, -O*H*), 7.64 (d, *J* = 7.67 Hz, 2H, Ar H), 7.26 (d, *J* = 7.75 Hz, 2H, Ar H), 7.18 (d, *J* = 7.65 Hz, 2H, Ar H), 7.07 (d, *J* = 7.37 Hz, 2H, Ar H), 6.70 (d, *J* = 2.46 Hz, 1H, Ar H), 6.22 (m, 3H, Ar H and –NH_2_), 5.09 (s, 2H, –C*H*_2_), 3.93 (s, 2H, –C*H*_2_). ^13^C NMR (100 MHz, DMSO-*d*_6_ δ): 158.6, 152.5, 150.3, 147.6, 139.9, 136.2, 130.6, 129.5, 128.8 (2C), 128.6 (2C), 127.2 (2C), 127.1 (2C), 120.2, 101.5, 99.8, 46.7, 40.5. MS (ESI) (*m*/*z*): [M + H]^+^ 408.11. HRMS (ESI) (*m*/*z*): [M + H]^+^ calcd for C_21_H_19_ClN_5_O_2_^+^, 408.1222; found, 408.1227.

### 4-(4-((2-Amino-4-chloro-7*H*-pyrrolo­[2,3-*d*]­pyrimidin-7-yl)­methyl)-2-chlorobenzyl)-*N*-hydroxybenzamide (**3**)

To a solution of **23** (0.13 g, 0.3 mmol, 1.0 equiv) in THF/MeOH 1:1, v/v (8 mL) were added NH_2_OH 50% W (3.0 mL, 30 equiv) and NaOH 1N (2.0 mL, 7.5 mmol, 30 equiv), and the reaction mixture was stirred at 25 °C for 2 h. The solvent was evaporated under vacuum, and the pH was adjusted to 4–5 with 1 N HCl. The resulting precipitate was recrystallized from MeOH/Et_2_O, after filtration and washing with water, leading to **3** (0.02 g, 28% yield) in high purity, as an amorphous white solid. ^1^H NMR (400 MHz, DMSO-*d*_6_ δ): 11.12 (s, 1H, -N*H*); 8.97 (br s, 1H, -O*H*); 7.65 (d, *J* = 8.12 Hz, 2H, Ar H), 7.32 (d, *J* = 8.42 Hz, 1H, Ar H), 7.23 (d, *J* = 8.33 Hz, 2H, Ar H), 7.20 (m, 1H, Ar H), 7.08 (d, *J* = 8.12 Hz, 1H, Ar H), 6.76 (d, *J* = 3.36 Hz, 1H, Ar H), 6.25 (m, 3H, Ar H and –NH_2_), 5.12 (s, 2H, −C*H*_2_), 4.06 (s, 2H, −C*H*_2_). ^13^C NMR (100 MHz, DMSO-*d*_6_ δ): 164.1, 158.7, 152.6, 150.3, 142.7, 139.0, 136.8, 133.1, 130.8, 129.5, 128.7, 128.5 (2C), 127.7, 127.1, 126.0, 120.2, 101.7, 99.9, 46.1, 37.9. MS (ESI) (*m*/*z*): [M + H]^+^ 442.07. HRMS (ESI) (*m*/*z*): [M + H]^+^ calcd for C_21_H_18_Cl_2_N_5_O_2_^+^, 442.0832; found, 442.0833.

### 4-(4-((2-Amino-6-chloro-9*H*-purin-9-yl)­methyl)­benzyl)-*N*-hydroxybenzamide (**4**)

To a solution of **24** (0.1 g, 0.25 mmol, 1.0 equiv) in THF/MeOH 1:1, v/v (4 mL) were added NH_2_OH 50% W (0.7 mL, 30 equiv) and NaOH 1N (1.0 mL, 7.5 mmol, 30 equiv), and the reaction mixture was stirred at 25 °C for 2 h. The solvent was evaporated under vacuum, and the pH was adjusted to 4–5 with 1 N HCl. The resulting precipitate was recrystallized from MeOH/Et_2_O, after filtration and washing with water, leading to **4** (0.07 g, 48% yield) in high purity, as an amorphous white solid. ^1^H NMR (400 MHz, DMSO-*d*_6_ δ): 11.17 (s, 1H, -N*H*), 9.03 (br s, 1H, -O*H*), 7.89 (s, 1H, Ar H), 7.71 (d, *J* = 7.11 Hz, 2H, Ar H), 7.33 (d, *J* = 8.84 Hz, 2H, Ar H), 7.27 (d, *J* = 8.46 Hz, 2H, Ar H), 7.21 (d, *J* = 7.11 Hz, 2H, Ar H), 6.55 (s, 2H, –NH_2_), 5.20 (s, 2H, −C*H*_2_), 4.00 (s, 2H, −C*H*_2_). ^13^C NMR (100 MHz, DMSO-*d*_6_ δ): 164.1, 156.6, 153.8, 151.1, 144.5, 140.2, 137.4, 134.9, 130.6, 129.0 (2C), 128.6 (2C), 127.3 (2C), 127.0 (2C), 116.0, 45.6, 40.4. MS (ESI) (*m*/*z*): [M + H]^+^ 409.11. HRMS (ESI) (*m*/*z*): [M + H]^+^ calcd for C_20_H_18_ClN_6_O_2_^+^, 409.1175; found, 409.1180.

### 4-(4-((2-Amino-6-chloro-9*H*-purin-9-yl)­methyl)-2-chlorobenzyl)-*N*-hydroxybenzamide (**5**)

To a solution of **25** (0.05 g, 0.11 mmol, 1.0 equiv) in THF/MeOH 1:1, v/v (4 mL) were added NH_2_OH 50% W (0.36 mL, 30 equiv) and NaOH 1N (0.25 mL, 30 equiv), and the reaction mixture was stirred at 25 °C for 2 h. The solvent was evaporated under vacuum, and the pH was adjusted to 4–5 with 1 N HCl. The resulting precipitate was recrystallized from MeOH/Et_2_O, after filtration and washing with water, leading to **5** (0.01 g, 12% yield) in high purity, as an amorphous white solid. ^1^H NMR (400 MHz, Methanol-*d*_*4*_ δ): 9.78 (br s, 1H, O*H*); 7.91 (m, 1H, Ar H), 7.67 (d, *J* = 8.05 Hz, 2H, Ar H), 7.59 (s, 1H, Ar H), 7.40 (d, *J* = 7.03 Hz, 1H, Ar H), 7.34 (d, *J* = 7.61 Hz, 2H, Ar H), 7.28 (d, *J* = 7.61 Hz, 2H, Ar H), 5.44 (s, 2H, −C*H*_2_), 4.16 (s, 2H, −C*H*_2_). ^13^C NMR (100 MHz, Methanol-*d*_*4*_ δ): 157.4, 155.1, 151.7, 140.1, 138.1, 136.0, 135.7, 133.12 (2C), 130.7 (2C), 130.1, 130.0, 128.4 (2C), 128.3, 109.1, 39.6, 21.2, 15.9. MS (ESI) (*m*/*z*): [M + H]^+^ 443.07. HRMS (ESI) (*m*/*z*): [M + H]^+^ calcd for C_20_H_17_Cl_2_N_6_O_2_^+^, 443.0785; found, 443.0789.

### 5-(4-((2-Amino-4-chloro-7*H*-pyrrolo­[2,3-*d*]­pyrimidin-7-yl)­methyl)­benzyl)-*N*-hydroxythiophene-2-carboxamide (**6**)

To a solution of **26** (0.08 g, 0.2 mmol, 1.0 equiv) in THF/MeOH 1:1, v/v (8 mL) were added NH_2_OH 50% W (1.0 mL, 30 equiv) and NaOH 1N (0.6 mL, 7.5 mmol, 30 equiv), and the reaction mixture was stirred at 25 °C for 2 h. The solvent was evaporated under vacuum, and the pH was adjusted to 4–5 with 1 N HCl. The resulting precipitate was recrystallized from MeOH/Et_2_O, after filtration and washing with water, leading to **6** (0.04 g, 47% yield), in high purity, as an amorphous white solid. ^1^H NMR (400 MHz, DMSO-*d*_6_ δ): 11.07 (s, 1H, -N*H*), 9.03 (br s, 1H, -O*H*), 7.42 (m, 1H, Ar H), 7.22 (d, *J* = 8.35 Hz, 2H, Ar H), 7.09 (d, *J* = 7.92 Hz, 2H, Ar H), 6.87 (d, *J* = 3.40 Hz, 1H, Ar H), 6.72 (d, *J* = 3.32 Hz, 1H, Ar H), 6.24 (d, *J* = 3.33 Hz, 1H, Ar H), 6.21 (s, 2H, –NH_2_), 5.11 (s, 2H, −C*H*_2_), 4.10 (s, 2H, −C*H*_2_). ^13^C NMR (100 MHz, DMSO-*d*_6_ δ): 158.7, 152.5, 150.3, 139.0, 136.7, 128.7 (4C), 127.2 (4C), 12.2 (2C), 101.5, 99.9, 46.7, 35.0. MS (ESI) (*m*/*z*): [M + H]^+^ 414.07. HRMS (ESI) (*m*/*z*): [M + H]^+^ calcd for C_19_H_17_ClN_5_O_2_S^+^, 414.0786; found, 414.0785.

### 5-(4-((2-Amino-4-chloro-7*H*-pyrrolo­[2,3-*d*]­pyrimidin-7-yl)­methyl)-2-chlorobenzyl)-*N*-hydroxythiophene-2-carboxamide (**7**)

To a solution of **27** (0.08 g, 0.2 mmol, 1.0 equiv) in THF/MeOH 1:1, v/v (8 mL) were added NH_2_OH 50% W (1.0 mL, 30 equiv) and NaOH 1N (0.6 mL, 7.5 mmol, 30 equiv), and the reaction mixture was stirred at 25 °C for 2 h. The solvent was evaporated under vacuum, and the pH was adjusted to 4–5 with 1 N HCl. The resulting precipitate was recrystallized from MeOH/Et_2_O, after filtration and washing with water, leading to **7** (0.04 g, 47% yield) in high purity, as an amorphous white solid. ^1^H NMR (400 MHz, DMSO-*d*_6_, δ): 11.09 (s, 1H, -N*H*), 9.05 (br s, 1H, -O*H*), 7.38 (d, *J* = 8.22 Hz, 2H, Ar H), 7.28 (s, 1H, Ar H), 7.23 (d, *J* = 8.03 Hz, 2H, Ar H), 7.11 (d, *J* = 3.38 Hz, 1H, Ar H), 6.70 (s, 2H, –NH_2_), 6.34 (d, *J* = 3.22 Hz, 1H, Ar H), 5.25 (s, 2H, −C*H*_2_), 4.21 (s, 2H, −C*H*_2_). ^13^C NMR (100 MHz, DMSO-*d*_6_, δ): 159.5, 153.5, 151.3, 138.7, 136.5, 132.9, 131.4 (2C), 127.8 (2C), 126.4 (2C), 126.2, 108.5, 99.1, 67.3, 46.1, 32.8, 22.8. MS (ESI) (*m*/*z*): [M + H]^+^ 448.03. HRMS (ESI) (*m*/*z*): [M + H]^+^ calcd for C_19_H_16_Cl_2_N_5_O_2_S^+^, 448.0397; found, 448.0396.

### 5-(4-((2-Amino-6-chloro-9*H*-purin-9-yl)­methyl)­benzyl)-*N*-hydroxythiophene-2-carboxamide (**8**)

To a solution of **28** (0.094 g, 0.23 mmol, 1.0 equiv) in THF/MeOH 1:1, v/v (3 mL) were added NH_2_OH 50% W (0.7 mL, 30 equiv) and NaOH 1N (1.0 mL, 7.5 mmol, 30 equiv), and the reaction mixture was stirred at 25 °C for 2 h. The solvent was evaporated under vacuum, and the pH was adjusted to 4–5 with 1 N HCl. The resulting precipitate was recrystallized from MeOH/Et_2_O, after filtration and washing with water, leading to **8** (0.04 g, 40% yield) in high purity, as an amorphous white solid. ^1^H NMR (400 MHz, DMSO-*d*_6_ δ): 11.07 (s, 1H, -N*H*), 9.05 (br s, 1H, -O*H*), 7.74 (s, 1H, Ar H), 7.42 (m, 1H, Ar H), 7.24 (d, *J* = 8.06 Hz, 2H, Ar H), 7.16 (d, *J* = 8.12 Hz, 2H, Ar H), 6.87 (d, *J* = 3.71 Hz, 1H, Ar H), 6.46 (s, 2H, –NH_2_), 5.15 (s, 2H, −C*H*_2_), 4.11 (s, 2H, −C*H*_2_). ^13^C NMR (100 MHz, DMSO-*d*_6_, δ): 156.8, 153.7, 151.2, 139.4, 137.4 (2C), 135.5, 128.8 (4C), 127.3 (4C), 116.5, 45.5, 34.9. MS (ESI) (*m*/*z*): [M + H]^+^ 414.86. HRMS (ESI) (*m*/*z*): [M + H]^+^ calcd for C_18_H_16_ClN_6_O_2_S^+^, 415.0738; found, 415.0741.

### 5-(4-((2-Amino-6-chloro-9H-purin-9-yl)­methyl)-2-chlorobenzyl)-*N*-hydroxythiophene-2-carboxamide (**9**)

To a solution of **29** (0.054 g, 0.12 mmol, 1.0 equiv) in THF/MeOH 1:1, v/v (3 mL) were added NH_2_OH 50% W (0.7 mL, 30 equiv) and NaOH 1N (1.0 mL, 7.5 mmol, 30 equiv), and the reaction mixture was stirred at 25 °C for 2 h. The solvent was evaporated under vacuum, and the pH was adjusted to 4–5 with 1 N HCl. The resulting precipitate was recrystallized from MeOH/Et_2_O, after filtration and washing with water, leading to **9** (0.022 g, 41% yield) in high purity, as an amorphous white solid. ^1^H NMR (400 MHz, DMSO-*d*_6_, δ): 11.07 (s, 1H, -N*H*), 9.04 (br s, 1H, -O*H*), 7.74 (s, 1H, Ar H), 7.42 (d, *J* = 3.6 Hz, 1H, Ar H), 7.24 (d, *J* = 1.7 Hz, 2H, Ar H), 7.16 (d, *J* = 1.8 Hz, 1H, Ar H), 6.87 (d, *J* = 1.8 Hz, 1H, Ar H), 6.46 (s, 2H, -N*H*_2_), 5.15 (s, 2H, −C*H*_2_), 4.11 (s, 2H, −C*H*_2_). ^13^C NMR (100 MHz, DMSO-*d*_6_, δ): 159.4, 153.9, 151.8, 149.6, 142.0, 137.6, 136.0, 134.8, 133.8, 132.1, 131.5, 129.0, 126.7, 126.5, 108.5, 99.1, 67.3, 46.1. MS (ESI) (*m*/*z*): [M + H]^+^ 449.8. HRMS (ESI) (*m*/*z*): [M + H]^+^ calcd for C_18_H_15_Cl_2_N_6_O_2_S^+^, 449.0349; found, 449.0351.

### 2-(((4-((2-Amino-4-chloro-7*H*-pyrrolo­[2,3-*d*]­pyrimidin-7-yl)­methyl)­phenyl)­amino)­methyl)-*N*-hydroxyoxazole-4-carboxamide (**10**)

To a solution of **32** (160 mg, 0.39 mmol, 1.0 equiv) in 1,4-dioxane/water (1:1, v/v, 6 mL) was added LiOH (93 mg, 3.9 mmol, 10 equiv), and the mixture was left stirring at r.t., overnight. The mixture resulting from the reaction was concentrated under vacuum, and the residue dissolved in water. The pH of the solution was then adjusted to 6 using a solution of NaHSO_4_ 1 M. The formed precipitate was filtered, washed with water, and dried under vacuum. LCMS analysis confirmed the presence of the carboxylic acid, and the crude was used for the next reaction step without any further purification. Carboxylic acid, HOBt (74 mg, 0.55 mmol, 1.4 equiv), and EDCI (105 mg, 0.55 mmol, 1.4 equiv) were dissolved in dry DMF (4 mL), and the mixture was left stirring at room temperature for 30 min. NH_2_OTMS (0.066 mL, 0.55 mmol, 1.4 equiv) was added, and the mixture was left stirring at r.t., overnight. The reaction mixture was then diluted with ethyl acetate (10 mL) and was washed with saturated aq. solution of NH_4_Cl (3 × 8 mL), saturated aq. solution of NaHCO_3_ (3 × 8 mL), and brine (3 × 8 mL). The organic layer was dried with anhydrous Na_2_SO_4_, filtered, and concentrated under reduced pressure. The crude was purified via automatic flash column chromatography (reverse phase, water/acetonitrile gradient from 5% to 100%) to afford **10** as a white amorphous solid (25% yield, 59 mg). ^1^H NMR (500 MHz, MeOD, δ): 8.34 (s, 1H, Isoxazole H), 7.12 (d, ^3^ *J*_H,H_ = 8.2 Hz, 2H, Ph H), 7.03 (d, ^3^ *J*_H,H_ = 3.6 Hz, 1H, Ar–H), 6.72 (d, ^3^ *J*_H,H_ = 8.2 Hz, 2H, Ph H), 6.41 (d, ^3^ *J*_H,H_ = 3.6 Hz, 1H, Ar–H), 5.18 (s, 2H, ArCH_2_), 4.51 (s, 2H, ArCH_2_). ^13^C NMR (126 MHz, MeOD, δ): 164.8, 160.7, 160.6, 154.8, 153.0, 148.8, 142.9, 135.4, 129.9, 127.6 (2C), 127.5 (2C), 114.1, 111.3, 100.4, 48.4, 41.7. HRMS (ESI) (*m*/*z*): [M + H]^+^ calcd for C_18_H_17_ClN_7_O_3_^+^, 414.1076; found, 414.1086.

### 5-(((4-((2-Amino-4-chloro-7H-pyrrolo­[2,3-*d*]­pyrimidin-7-yl)­methyl)­phenyl)­amino)­methyl)-*N*-hydroxythiophene-2-carboxamide (**11**)

To a solution of **33** (180 mg, 0.142 mmol, 1.0 equiv) in 1,4-dioxane/water (1:1, v/v, 6 mL) was added LiOH (100 mg, 4.2 mmol, 10 equiv), and the mixture was left stirring at r.t., overnight. The mixture resulting from the reaction was concentrated under vacuum, and the residue dissolved in water. The pH of the solution was then adjusted to 6 using a solution of NaHSO_4_ 1 M. The formed precipitate was filtered, washed with water, and dried under vacuum. LCMS analysis confirmed the presence of the carboxylic acid, and the crude was used for the next reaction step without any further purification. Carboxylic acid, HOBt (80 mg, 0.59 mmol, 1.4 equiv), and EDCI (113 mg, 0.59 mmol, 1.4 equiv) were dissolved in dry DMF (4 mL), and the mixture was left stirring at room temperature for 30 min. NH_2_OTMS (0.071 mL, 0.59 mmol, 1.4 equiv) was added, and the mixture was left stirring at r.t., overnight. The reaction mixture was then diluted with ethyl acetate (10 mL) and was washed with saturated aq. solution of NH_4_Cl (3 × 8 mL), saturated aq. solution of NaHCO_3_ (3 × 8 mL), and brine (3 × 8 mL). The organic layer was dried with anhydrous Na_2_SO_4_, filtered, and concentrated under reduced pressure. The crude was purified via automatic flash column chromatography (reverse phase, water/acetonitrile gradient from 5% to 100%) to afford **11** as a white amorphous solid (25% yield, 49 mg). ^1^H NMR (600 MHz, MeOD, δ): 7.49 (m, 1H, Thiophene H), 7.12 (d, ^3^ *J*_H,H_ = 8.0 Hz, 2H, Ph H), 7.06 (d, ^3^ *J*_H,H_ = 3.9 Hz, 1H, Thiophene) 7.03 (d, ^3^ *J*_H,H_ = 3.6 Hz, 1H, Ar–H), 6.73 (d, ^3^ *J*_H,H_ = 8.1 Hz, 2H, Ph H), 6.40 (d,^3^ *J*_H,H_ = 3.6 Hz, 1H, Ar–H), 5.18 (s, 2H, ArCH_2_), 4.58 (s, 2H, ArCH_2_). ^13^C NMR (126 MHz, MeOD, δ): 163.1, 160.4, 154.8, 152.9, 147.9, 135.5, 129.9, 129.5, 128.3, 127.7 (2C), 126.5 (2C), 115.2, 114.6, 111.3, 100.4, 44.5, 27.2. HRMS (ESI) (*m*/*z*): [M + H]^+^ calcd for C_19_H_18_ClN_6_O_2_S^+^, 429.0895; found, 429.0901.

### 2-(((4-((2-Amino-4-chloro-7*H*-pyrrolo­[2,3-*d*]­pyrimidin-7-yl)­methyl)­phenyl)­amino)­methyl)-*N*-hydroxythiazole-5-carboxamide (**12**)

To a solution of **34** (100 mg, 0.23 mmol, 1.0 equiv) in 1,4-dioxane/water (1:1, v/v, 6 mL) was added LiOH (55 mg, 2.3 mmol, 10 equiv), and the mixture was left stirring at r.t., overnight. The mixture resulting from the reaction was concentrated under vacuum, and the residue dissolved in water. The pH of the solution was then adjusted to 6 using a solution of NaHSO_4_ 1 M. The formed precipitate was filtered, washed with water, and dried under vacuum. LCMS analysis confirmed the presence of the carboxylic acid, and the crude was used for the next reaction step without any further purification. Carboxylic acid, HOBt (43 mg, 0.32 mmol, 1.4 equiv), and EDCI (61 mg, 0.32 mmol, 1.4 equiv) were dissolved in dry DMF (4 mL), and the mixture was left stirring at room temperature for 30 min. NH_2_OTMS (0.028 mL, 0.32 mmol, 1.4 equiv) was added, and the mixture was left stirring at r.t., overnight. The reaction mixture was then diluted with ethyl acetate (10 mL) and was washed with saturated aq. solution of NH_4_Cl (3 × 8 mL), saturated aq. solution of NaHCO_3_ (3 × 8 mL), and brine (3 × 8 mL). The organic layer was dried with anhydrous Na_2_SO_4_, filtered, and concentrated under reduced pressure. The crude was purified via automatic flash column chromatography (reverse phase, water/acetonitrile gradient from 5% to 100%) to afford **12** as a white amorphous solid (40% yield, 40 mg). ^1^H NMR (400 MHz, MeOD, δ): 8.05 (s, 1H, Thiazole H), 7.01 (d, ^3^ *J*_H,H_ = 8.2 Hz, 2H, Ph H), 6.95 (d, ^3^ *J*_H,H_ = 3.6 Hz, 1H, Ar–H), 6.54 (d, ^3^ *J*_H,H_ = 8.2 Hz, 2H, Ph H), 6.31 (d, ^3^ *J*_H,H_ = 3.6 Hz, 1H, Ar–H), 5.07 (s, 2H, ArCH_2_), 4.56 (s, 2H, ArCH_2_). ^13^C NMR (100 MHz, MeOD, δ): 154.7, 152.4, 151.4, 148.4, 144.2, 123.0, 128.9, 128.3, 128.0 (2C), 127.7 (2C), 114.2, 111.3, 107.7, 100.5, 48.4, 47.0. HRMS (ESI) (*m*/*z*): [M + H]^+^ calcd for C_18_H_17_ClN_7_O_2_S^+^, 430.0848; found, 430.0849.

### 4-(((4-((2-Amino-4-chloro-7*H*-pyrrolo­[2,3-*d*]­pyrimidin-7-yl)­methyl)­phenyl)­amino)­methyl)-*N*-hydroxybenzamide (**13**)

To a solution of **35** (150 mg, 0.35 mmol, 1.0 equiv) in 1,4-dioxane/water (1:1, v/v, 6 mL) was added LiOH (84 mg, 3.5 mmol, 10 equiv), and the mixture was left stirring at r.t., overnight. The mixture resulting from the reaction was concentrated under vacuum, and the residue dissolved in water. The pH of the solution was then adjusted to 6 using a solution of NaHSO_4_ 1 M. The formed precipitate was filtered, washed with water, and dried under vacuum. LCMS analysis confirmed the presence of the carboxylic acid, and the crude was used for the next reaction step without any further purification. Carboxylic acid, HOBt (66 mg, 0.49 mmol, 1.4 equiv), and EDCI (94 mg, 0.49 mmol, 1.4 equiv) were dissolved in dry DMF (4 mL), and the mixture was left stirring at room temperature for 30 min. NH_2_OTMS (0.059 mL, 0.49 mmol, 1.4 equiv) was added, and the mixture was left stirring at r.t., overnight. The reaction mixture was then diluted with ethyl acetate (10 mL) and was washed with saturated aq. solution of NH_4_Cl (3 × 8 mL), saturated aq. solution of NaHCO_3_ (3 × 8 mL), and brine (3 × 8 mL). The organic layer was dried with anhydrous Na_2_SO_4_, filtered, and concentrated under reduced pressure. The crude was purified via automatic flash column chromatography (reverse phase, water/acetonitrile gradient from 5% to 100%) to afford **13** as a white amorphous solid (47% yield, 69 mg). ^1^H NMR (500 MHz, MeOD, δ): 7.77 (d, 2H, ^3^ *J*_H,H_ = 8.6 Hz, 2H, Ph H), 7.51 (d, 2H, ^3^ *J*_H,H_ = 8.2 Hz, 2H, Ph H), 7.13 (d, 2H, ^3^ *J*_H,H_ = 8.2 Hz, 2H, Ph H), 7.04 (d, 1H, ^3^ *J*_H,H_ = 3.7 Hz, Ar H), 6.73 (d, 2H, ^3^ *J*_H,H_ = 8.2 Hz, 2H, Ph H), 6.42 (d, 1H, ^3^ *J*_H,H_ = 3.7 Hz, Ar H), 5.20 (s, 2H, ArCH_2_), 4.47 (s, 2H, ArCH_2_). ^13^C NMR (126 MHz, MeOD, δ): 168.0, 160.6, 154.8, 153.0, 147.9, 144.5, 132.4 (2C), 129.9 (2C), 128.8 (2C), 128.3 (2C), 127.6 (2C), 115.4, 111.2, 100.4, 50.6, 48.4. HRMS (ESI) (*m*/*z*): [M + H]^+^ calcd for C_21_H_20_ClN_6_O_2_^+^, 423.1331; found, 423.1322.

### 5-((5-((2-Amino-4-chloro-7*H*-pyrrolo­[2,3-*d*]­pyrimidin-7-yl)­methyl)-1*H*-indol-1-yl)­methyl)-*N*-hydroxythiophene-2-carboxamide (**14**)

To a solution of **40** (85 mg, 0.188 mmol, 1.0 equiv) in 1,4-dioxane/water (1:1, v/v, 6 mL) was added LiOH (45 mg, 1.88 mmol, 10 equiv), and the mixture was left stirring at r.t., overnight. The mixture resulting from the reaction was concentrated under vacuum, and the residue dissolved in water. The pH of the solution was then adjusted to 6 using a solution of NaHSO_4_ 1 M. The formed precipitate was filtered, washed with water, and dried under vacuum. LCMS analysis confirmed the presence of the carboxylic acid, and the crude was used for the next reaction step without any further purification. Carboxylic acid (70 mg, 0.16 mmol, 1.0 equiv) and DMAP (29 mg, 0.24 mmol, 1.5 equiv) were dissolved in dry DCM (4 mL); after 30 min, EDCI (92 mg, 0.48 mmol, 3.0 equiv) and NH_2_OTMS (23 μL, 0.192 mmol, 1.2 equiv) were added, and the mixture was left stirring at r.t., overnight. The reaction mixture was then diluted with ethyl acetate and washed with saturated aq. solution of NH_4_Cl (3 × 10 mL), saturated aq. solution of NaHCO_3_ (3 × 10 mL), and brine (3 × 10 mL). The organic layer was dried with anhydrous Na_2_SO_4_, filtered, and concentrated under reduced pressure. The crude was purified via automatic flash column chromatography (reverse phase, water/ACN gradient from 5% to 100%) to afford **14** as a white solid (54 mg, 63% yield); mp = > 262 °C with decomposition. ^1^H NMR (400 MHz, MeOD, δ): 7.45 (s, 1H, Ar–H), 7.37 (d, *J* = 8.4 Hz, 2H, Ar–H), 7.30 (d, *J* = 3.2 Hz, 1H, Ar–H), 7.08 (d, *J* = 8.5 Hz, 1H, Ar–H), 7.01 (d, *J* = 3.7 Hz, 1H, Ar–H), 6.94 (d, *J* = 3.9 Hz, 1H, Ar–H), 6.51–6.41 (m, 1H, Ar–H), 6.35 (d, *J* = 3.7 Hz, 1H, Ar–H), 5.56 (s, 2H, –CH_2_), 5.33 (s, 2H, –CH_2_). ^13^C NMR (100 MHz, DMSO-*d*_6_) 153.6, 148.7, 135.0, 130.2, 129.9, 127.2, 122.1, 120.2, 109.5, 102.4, 99.4, 84.6, 47.5, 27.9. HRMS (ESI) (*m*/*z*): [M + H]^+^ calcd for C_21_H_18_ClN_6_O_2_S^+^, 453.0895; found, 453.0886.

### 4-((2-Amino-7-(benzo­[*d*]­[1,3]­dioxol-5-ylmethyl)-4-chloro-7*H*-pyrrolo­[2,3-*d*]­pyrimidin-5-yl)­methyl)-*N*-hydroxybenzamide (**15**)

To a solution of **45** (90 mg, 0.2 mmol, 1.0 equiv) in 1,4-dioxane/water (3:1, v/v, 18 mL) was added LiOH (38 mg, 1.6 mmol, 8.0 equiv), and the mixture was left stirring at room temperature for 3 days. The reaction mixture was concentrated under reduced pressure; the residue was taken up in water and adjusted to pH 4 with 2 N HCl. The formed precipitate was filtered, washed with water, and dried under vacuum. LCMS analysis confirmed the presence of the carboxylic acid, and the crude was used for the next reaction step without any further purification. Carboxylic acid (52 mg, 0.12 mmol, 1.0 equiv) and DMAP (22 mg, 0.18 mmol, 1.5 equiv) were dissolved in dry DMF (4 mL); after 30 min, EDCI (69 mg, 0.36 mmol, 3.0 equiv) and NH_2_OTMS (15 μL, 0.13 mmol, 1.1 equiv) were added, and the mixture was left stirring at r.t., overnight. The reaction mixture was then diluted with ethyl acetate and washed with saturated aq. solution of NH_4_Cl (3 × 10 mL), saturated aq. solution of NaHCO_3_ (3 × 10 mL), and brine (3 × 10 mL). The organic layer was dried with anhydrous Na_2_SO_4_, filtered, and concentrated under reduced pressure. The crude was purified via automatic flash column chromatography (reverse phase, water/acetonitrile gradient from 5% to 100%) to afford **15** as a white solid (16 mg, 25% yield); mp = > 260 °C with decomposition. ^1^H NMR (400 MHz, DMSO-*d*_6_, δ): 11.12 (br s, 1H, –NH), 8.97 (br s, 1H, –OH), 7.65 (d, ^3^ *J*_H,H_ = 8.3 Hz, 2H, Ph H-2,6), 7.25 (d, ^3^ *J*_H,H_ = 8.3 Hz, 2H, Ph H-3,5), 6.92 (s, 1H, Pyr H-6), 6.85 (d, ^3^ *J*_H,H_ = 6.7 Hz, 1H, Ar H), 6.82 (s, 1H, Ar H) 6.68 (^3^ *J*_H,H_ = 6.7 Hz, 1H, Ar H), 6.67 (br s, 2H, -N*H*_2_), 5.98 (s, 2H, -OC*H*_2_O), 5.09 (s, 2H, −C*H*_2_N), 4.08 (s, 2H, −C*H*_2_Ar). ^13^C NMR (100 MHz, DMSO-*d*_6_, δ): 165.7, 159.3, 154.0, 151.1, 147.4, 146.6, 146.2, 131.5, 129.4 (2C), 128.5 (2C), 124.7, 120.8, 112.2, 108.3, 108.0, 107.3, 101.1, 46.6, 31.2. MS (ESI) (*m*/*z*): [M + H]^+^ 452.47. HRMS (ESI) (*m*/*z*): [M + H]^+^ calcd for C_22_H_19_ClN_5_O_4_^+^, 452.1121; found, 452.1124.

### 4-((2-Amino-7-((6-bromobenzo­[*d*]­[1,3]­dioxol-5-yl)­methyl)-4-chloro-7*H*-pyrrolo­[2,3-*d*]­pyrimidin-5-yl)­methyl)-*N*-hydroxybenzamide (**16**)

To a solution of **46** (80 mg, 0.15 mmol, 1.0 equiv) in 1,4-dioxane/water (3:1, v/v, 10 mL) was added LiOH (36 mg, 1.5 mmol, 10.0 equiv), and the mixture was left stirring at r.t., overnight. The reaction mixture was concentrated under reduced pressure; the residue was taken up in water and adjusted to pH 4 with 2 N HCl. The formed precipitate was filtered, washed with water, and dried under vacuum. LCMS analysis confirmed the presence of the carboxylic acid, and the crude was used for the next reaction step without any further purification. Carboxylic acid (50 mg, 0.1 mmol, 1.0 equiv) and DMAP (18 mg, 0.14 mmol, 1.5 equiv) were dissolved in dry DMF (2 mL); after 30 min, EDCI (56 mg, 0.19 mmol, 3.0 equiv) and NH_2_OTMS (14 μL, 0.12 mmol, 1.2 equiv) were added, and the mixture was left stirring at r.t., overnight. The reaction mixture was then diluted with ethyl acetate and washed with saturated aq. solution of NH_4_Cl (3 × 10 mL), saturated aq. solution of NaHCO_3_ (3 × 10 mL), and brine (3 × 10 mL). The organic layer was dried with anhydrous Na_2_SO_4_, filtered, and concentrated under reduced pressure. The crude was purified via automatic flash column chromatography (reverse phase, water/ACN gradient from 5% to 100%) to afford **16** as a white solid (27 mg, 51% yield); mp = > 265 °C with decomposition. ^1^H NMR (400 MHz, MeOD, δ): 7.65 (d, ^3^ *J*_H,H_ = 8.5 Hz, 2H, Ph H-2,6), 7.25 (d, ^3^ *J*_H,H_ = 8.5 Hz, 2H, Ph H-3,5), 7.05 (s, 1H, Pyr H-6), 6.72 (s, 1H, Ar H), 6.49 (s, 1H, Ar H), 5.94 (s, 2H, -OC*H*_2_O), 5.20 (s, 2H, −C*H*_2_N), 4.17 (s, 2H, −C*H*_2_Ar). ^13^C NMR (100 MHz, MeOD, δ): 168.2, 160.6, 155.8, 153.1, 149.7, 149.3, 146.3, 131.4, 130.6, 129.8 (2C), 128.2 (2C), 126.2, 115.2, 114.3, 113.7, 110.2, 109.6, 103.5, 49.0, 32.6. MS (ESI) (*m*/*z*): [M + H]^+^ HRMS (ESI) (*m*/*z*): [M + H]^+^ calcd for C_22_H_18_BrClN_5_O_4_^+^, 530.0226; found, 530.0224.

### 4-((2-Amino-4-chloro-7-((4-methoxy-3,5-dimethylpyridin-2-yl)­methyl)-7H-pyrrolo­[2,3-*d*]­pyrimidin-5-yl)­methyl)-*N*-hydroxybenzamide (**17**)

To a solution of **47** (112 mg, 0.23 mmol, 1.0 equiv) in 1,4-dioxane/water (3:1, v/v, 16 mL) was added LiOH (55 mg, 2.3 mmol, 10 equiv), and the mixture was left stirring at r.t., overnight. The reaction mixture was concentrated under reduced pressure; the residue was taken up in water and adjusted to pH 6 with 2 N HCl. The formed precipitate was filtered, washed with water, and dried under vacuum. LCMS analysis confirmed the presence of the carboxylic acid, and the crude was used for the next reaction step without any further purification. Carboxylic acid (97 mg, 0.21 mmol, 1.0 equiv) and DMAP (37 mg, 0.3 mmol, 1.5 equiv) were dissolved in dry DMF (4 mL); after 30 min, EDCI (115 mg, 0.6 mmol, 3.0 equiv) and NH_2_OTMS (29 μL, 0.24 mmol, 1.2 equiv) were added, and the mixture was left stirring at r.t., overnight. The reaction mixture was then diluted with ethyl acetate and washed with saturated aq. solution of NH_4_Cl (3 × 10 mL), saturated aq. solution of NaHCO_3_ (3 × 10 mL), and brine (3 × 10 mL). The organic layer was dried with anhydrous Na_2_SO_4_, filtered, and concentrated under reduced pressure. The crude was purified via automatic flash column chromatography (reverse phase, water/ACN gradient from 5% to 100%) to afford **17** as a white solid (37 mg, 40% yield); mp = > 260 °C with decomposition. ^1^H NMR (400 MHz, MeOD, δ): 8.21 (s, 1H, Pyridine), 7.65 (d, ^3^ *J*_H,H_ = 8.1 Hz, 2H, Ph H-2,6), 7.29 (d, ^3^ *J*_H,H_ = 8.1 Hz, 2H, Ph H-3,5), 6.71 (s, 1H, Pyrrole H-6), 5.41 (s, 2H, −C*H*_2_N), 4.16 (s, 2H, −C*H*_2_Ar), 3.92 (s, 3H, –OCH_3_), 2.33 (s, 3H, Pyridine–C*H*_3_), 2.29 (s, 3H, Pyridine–C*H*_3_). ^13^C NMR (100 MHz, MeOD, δ): 168.1, 160.8, 156.2, 153.5, 152.0, 146.0, 144.9, 131.4, 130.5, 129.9 (2C), 129.3, 128.2 (2C), 125.9, 116.0, 109.7, 61.7, 46.1, 32.7, 14.1, 11.4. MS (ESI) (*m*/*z*): [M + H]^+^ 467.49. HRMS (ESI) (*m*/*z*): [M + H]^+^ calcd for C_23_H_24_ClN_6_O_3_^+^, 467.1593; found, 467.1596.

### 4-Chloro-7-(4-(4,4,5,5-tetramethyl-1,3,2-dioxaborolan-2-yl)­benzyl)-7*H*-pyrrolo­[2,3-*d*]­pyrimidin-2-amine (**18**)

To a solution of 4-chloro-7*H*-pyrrolo­[2,3-*d*]­pyrimidin-2-amine (0.7 g, 4 mmol, 1.0 equiv) and 2-(4-(bromomethyl)­phenyl)-4,4,5,5-tetramethyl-1,3,2-dioxaborolane (1.31 g, 4.4 mmol, 1.1 equiv) in dry DMF (12 mL) was added NaH (0.08 g, 4.8 mmol, 1.2 equiv), and the reaction mixture was stirred at r.t., overnight. The reaction mixture was extracted with ethyl acetate (3 × 12 mL), after being poured into water. The resulting combined organic layers were washed with brine (3 × 10 mL), dried with anhydrous Na_2_SO_4_, and filtered, and the solvent was evaporated under vacuum. The crude mixture was then purified by silica gel column chromatography using *n*-hexane/ethyl acetate (7:3, v/v) as an eluent to afford the pure **18** (0.73 g, 48% yield) as an amorphous white solid. ^1^H NMR (400 MHz, CDCl_3_, δ): 7.76 (d, *J* = 8.3 Hz, 2H, Ar H), 7.18 (d, *J* = 7.7 Hz, 2H, Ar H), 6.80 (d, *J* = 3.8 Hz, 1H, Ar H), 6.41 (d, *J* = 3.8 Hz, 1H, Ar H), 5.26 (s, 2H, −C*H*_2_), 5.11 (br s, 2H, -N*H*_2_), 1.33 (s, 12H, −C*H*_3_). ^13^C NMR (100 MHz, CDCl_3_, δ): 158.3, 153.4, 152.6, 139.6, 135.5 (2C), 135.2, 127.0 (2C), 126.1, 110.8, 100.6, 84.0, 48.2 (2C), 25.0 (4C). MS (ESI) (*m*/*z*): [M + H]^+^ 385.15. HRMS (ESI) (*m*/*z*): [M + H]^+^ calcd for C_19_H_23_BClN_4_O_2_^+^, 385.1598; found, 385.1595.

### 4-Chloro-7-(3-chloro-4-(4,4,5,5-tetramethyl-1,3,2-dioxaborolan-2-yl)­benzyl)-7*H*-pyrrolo­[2,3-*d*]­pyrimidin-2-amine (**19**)

To a solution of 4-chloro-7*H*-pyrrolo­[2,3-*d*]­pyrimidin-2-amine (1.01 g, 6 mmol, 1.2 equiv) and 2-(4-(bromomethyl)-2-chlorophenyl)-4,4,5,5-tetramethyl-1,3,2-dioxaborolane (1.66 g, 5 mmol, 1.0 equiv) in dry DMF (12 mL) was added K_2_CO_3_ (1.4 g, 10 mmol, 2 equiv), and the reaction mixture was stirred at r.t., overnight. The reaction mixture was extracted with ethyl acetate (3 × 10 mL), after being poured into water. The combined organic layers were washed with brine (3 × 10 mL), dried with anhydrous Na_2_SO_4_, and filtered, and the solvent was evaporated under vacuum. The crude mixture was then purified by silica gel column chromatography using *n*-hexane/ethyl acetate (8:2, v/v) as an eluent to afford the pure **19** (0.98 g, 47% yield) as an amorphous white solid. ^1^H NMR (600 MHz, CDCl_3_, δ): 7.64 (d, *J* = 8.4 Hz, 1H, Ar H), 7.16 (m, 1H, Ar H), 7.03 (d, *J* = 7.4 Hz, 1H, Ar H), 6.78 (d, *J* = 3.4 Hz, 1H, Ar H), 6.42 (d, *J* = 3.6 Hz, 1H, Ar H), 5.21 (s, 2H, −C*H*_2_), 5.02 (br s, 2H, -N*H*_2_), 1.35 (s, 12H, −C*H*_3_). ^13^C NMR (150 MHz, CDCl_3_, δ): 158.6, 153.6, 152.8, 140.9, 140.3, 137.1 (2C), 128.4, 125.7, 125.0, 110.8, 100.8, 84.4, 47.4 (2C), 24.9 (4C). MS (ESI) (*m*/*z*): [M + H]^+^ 419.11. HRMS (ESI) (*m*/*z*): [M + H]^+^ calcd for C_19_H_22_BCl_2_N_4_O_2_^+^, 419.1208; found, 419.1208.

### 6-Chloro-9-(4-(4,4,5,5-tetramethyl-1,3,2-dioxaborolan-2-yl)­benzyl)-9*H*-purin-2-amine (**20**)

To a solution of 6-chloro-9*H*-purin-2-amine (0.17 g, 1 mmol, 1.0 equiv) and 2-(4-(bromomethyl)­phenyl)-4,4,5,5-tetramethyl-1,3,2-dioxaborolane (0.450 g, 1.5 mmol, 1.5 equiv) in dry DMF (5 mL) was added K_2_CO_3_ (0.55 g, 4 mmol, 4 equiv), and the reaction mixture was stirred at r.t., overnight. The reaction mixture was extracted with ethyl acetate (3 × 10 mL), after being poured into water. The combined organic layers were washed with brine (3 × 10 mL), dried with anhydrous Na_2_SO_4_, and filtered, and the solvent was evaporated under vacuum. The crude mixture was then purified by silica gel column chromatography using *n*-hexane/ethyl acetate (2:8, v/v) as an eluent to afford the pure **20** (0.7 g, 60% yield) as an amorphous white solid. ^1^H NMR (400 MHz, DMSO-*d*_6_, δ): 8.23 (s, 1H, Ar H), 7.64 (d, *J* = 8.1 Hz, 2H, Ar H), 7.24 (d, *J* = 8.0 Hz, 2H, Ar H), 6.93 (s, 2H, -N*H*_2_), 5.32 (s, 2H, −C*H*_2_), 1.28 (s, 12H, −C*H*_3_). ^13^C NMR (100 MHz, DMSO-*d*_6_, δ): 159.9, 154.1, 149.5, 143.2, 139.8, 134.8 (2C), 126.5 (2C), 123.2, 83.7 (2C), 46.1, 24.9, 24.6 (4C). MS (ESI) (*m*/*z*): [M + H]^+^ 386.14. HRMS (ESI) (*m*/*z*): [M + H]^+^ calcd for C_18_H_22_BClN_5_O_2_^+^, 386.1550; found, 386.1554.

### 6-Chloro-9-(3-chloro-4-(4,4,5,5-tetramethyl-1,3,2-dioxaborolan-2-yl)­benzyl)-9*H*-purin-2-amine (**21**)

To a solution of 6-chloro-9*H*-purin-2-amine (1.5 g, 9 mmol, 1.0 equiv) and 2-(4-(bromomethyl)-2-chlorophenyl)-4,4,5,5-tetramethyl-1,3,2-dioxaborolane (0.450 g, 13.5 mmol, 1.5 equiv) in dry DMF (5 mL) was added K_2_CO_3_ (0.55 g, 4 mmol, 4 equiv), and the reaction mixture was stirred at r.t., overnight. The reaction mixture was extracted with ethyl acetate (3 × 10 mL), after being poured into water. The combined organic layers were washed with brine (3 × 10 mL), dried with anhydrous Na_2_SO_4_, and filtered, and the solvent was evaporated under vacuum. The crude mixture was then purified by silica gel column chromatography using *n*-hexane/ethyl acetate (2:8, v/v) as an eluent to afford the pure **21** (0.7 g, 60% yield) as an amorphous white solid. ^1^H NMR (400 MHz, DMSO-*d*_6_, δ): 8.23 (s, 1H, Ar H), 7.64 (d, *J* = 8.1 Hz, 2H, Ar H), 7.24 (d, *J* = 8.0 Hz, 1H, Ar H), 6.93 (s, 2H, -N*H*_2_), 5.32 (s, 2H, −C*H*_2_), 1.28 (s, 12H, −C*H*_3_). ^13^C NMR (100 MHz, DMSO-*d*_6_, δ): 159.9, 154.1, 149.5, 143.2, 139.8, 134.8 (2C), 126.5 (2C), 123.2, 83.7 (2C), 46.1, 24.9, 24.6 (4C). MS (ESI) (*m*/*z*): [M + H]^+^ 420.14. HRMS (ESI) (*m*/*z*): [M + H]^+^ calcd for C_18_H_21_BCl_2_N_5_O_2_^+^, 420.1160; found, 420.1162.

### Methyl 4-(4-((2-Amino-4-chloro-7*H*-pyrrolo­[2,3-*d*]­pyrimidin-7-yl)­methyl)­benzyl)­benzoate (**22**)

To a solution of **18** (0.69 g, 1.8 mmol, 1.0 equiv) and methyl 4-(bromomethyl)­benzoate (0.5 g, 2.15 mmol, 1.2 equiv) in toluene/EtOH 1:3, v/v (15 mL) were added K_2_CO_3_ (0.5 g, 3.2 mmol, 2 equiv) and Pd­(PPh_3_)_4_ (0.1 g, 0.18 mmol, 0.1 equiv), and the reaction mixture was stirred at 90 °C for 6 h, under a N_2_ atmosphere. The residue was filtered off, and the solid was washed with ethyl acetate. The solution was poured into water and then extracted with ethyl acetate (3 × 10 mL). The combined organic layers were washed with brine (3 × 10 mL), dried with anhydrous Na_2_SO_4_, and filtered, and the solvent was evaporated under vacuum. The crude mixture was then purified by silica gel column chromatography using *n*-hexane/ethyl acetate (7:3, v/v) as an eluent to afford the pure **22** (0.06 g, 18% yield) as an amorphous white solid. ^1^H NMR (400 MHz, CDCl_3_, δ): 7.94 (d, *J* = 8.8 Hz, 2H, Ar H), 7.22 (d, *J* = 8.7 Hz, 2H, Ar H), 7.12 (m, 4H, Ar H), 6.81 (d, *J* = 3.3 Hz, 1H, Ar H), 6.40 (d, *J* = 3.6 Hz, 1H, Ar H), 5.22 (s, 2H, −C*H*_2_), 5.09 (br s, 2H, -N*H*_2_), 3.99 (s, 2H, −C*H*_2_), 3.89 (s, 3H, -OC*H*_3_). ^13^C NMR (100 MHz, CDCl_3_, δ): 167.1, 158.4, 153.4, 152.7, 146.2, 140.1, 134.9, 130.0 (2C), 129.5 (2C), 129.0 (2C), 128.4 (2C), 127.9, 126.0, 110.8, 100.4, 52.2, 47.8, 41.7. MS (ESI) (*m*/*z*): [M + H]^+^ 407.12. HRMS (ESI) (*m*/*z*): [M + H]^+^ calcd for C_22_H_20_ClN_4_O_2_^+^, 407.1270; found, 407.1274.

### Methyl 4-(4-((2-Amino-4-chloro-7*H*-pyrrolo­[2,3-*d*]­pyrimidin-7-yl)­methyl)­benzyl)­benzoate (**23**)

To a solution of **19** (0.9 g, 2.15 mmol, 1.0 equiv) and methyl 4-(bromomethyl)­benzoate (0.6 g, 2.56 mmol, 1.2 equiv) in toluene/EtOH 1:3, v/v (21 mL) were added K_2_CO_3_ (0.6 g, 4.3 mmol, 2 equiv) and Pd­(PPh_3_)_4_ (0.25 g, 0.22 mmol, 0.1 equiv), and the reaction mixture was stirred at 90 °C for 6 h, under a N_2_ atmosphere. The residue was filtered off, and the solid was washed with ethyl acetate. The solution was poured into water and then extracted with ethyl acetate (3 × 10 mL). The combined organic layers were washed with brine (3 × 10 mL), dried with anhydrous Na_2_SO_4_, and filtered, and the solvent was evaporated under vacuum. The crude mixture was then purified by silica gel column chromatography using *n*-hexane/ethyl acetate (7:3, v/v) as an eluent to afford the pure **23** (0.13 g, 14% yield) as an amorphous white solid. ^1^H NMR (400 MHz, CDCl_3_, δ): 7.94 (d, *J* = 9.1 Hz, 2H, Ar H) 7.22 (m, 3H, Ar H), 7.09 (d, *J* = 7.2 Hz, 1H, Ar H), 7.01 (d, *J* = 8.1 Hz, 1H, Ar H), 6.82 (d, *J* = 3.6 Hz, 1H, Ar H), 6.43 (d, *J* = 3.9 Hz, 1H, Ar H), 5.21 (s, 2H, −C*H*_2_), 5.04 (br s, 2H, -N*H*_2_), 4.11 (s, 2H, −C*H*_2_), 3.89 (s, 3H, -OC*H*_3_). ^13^C NMR (100 MHz, CDCl_3_, δ): 167.1, 158.5, 152.9, 144.7, 137.7, 137.1, 134.8, 131.6 (2C), 130.0 (2C), 129.0 (2C), 128.7, 128.4, 128.5, 126.2, 125.8, 110.8, 52.2, 47.2, 39.1. MS (ESI) (*m*/*z*): [M + H]^+^ 441.08. HRMS (ESI) (*m*/*z*): [M + H]^+^ calcd for C_22_H_19_Cl_2_N_4_O_2_^+^, 441.0880; found, 441.0884.

### Methyl 4-(4-((2-Amino-6-chloro-9*H*-purin-9-yl)­methyl)­benzyl)­benzoate (**24**)

To a solution of **20** (0.19 g, 0.5 mmol, 1.2 equiv) and methyl 4-(bromomethyl)­benzoate (0.09 g, 0.4 mmol, 1.0 equiv) in toluene/EtOH 1:3, v/v (8 mL) were added Na_2_CO_3_ (0.08 g, 0.8 mmol, 2 equiv) and Pd­(PPh_3_)_4_ (0.04 g, 0.04 mmol, 0.1 equiv), and the reaction mixture was stirred at 90 °C for 6 h, under a N_2_ atmosphere. The residue was filtered off, and the solid was washed with ethyl acetate. The solution was poured into water and then extracted with ethyl acetate (3 × 10 mL). The combined organic layers were washed with brine (3 × 10 mL), dried with anhydrous Na_2_SO_4_, and filtered, and the solvent was evaporated under vacuum. The crude mixture was then purified by silica gel column chromatography using *n*-hexane/ethyl acetate (1:1, v/v) as an eluent to afford the pure **24** (0.07 g, 48% yield) as an amorphous white solid. ^1^H NMR (400 MHz, CDCl_3_) 7.94 (d, *J* = 8.0 Hz, 2H, Ar H), 7.73 (s, 1H, Ar H), 7.24 (d, *J* = 8.0 Hz, 2H, Ar H), 7.19 (m, 6H, Ar H), 5.21 (s, 2H, −C*H*_2_), 4.00 (s, 2H, −C*H*_2_), 3.89 (s, 3H, -OC*H*_3_). ^13^C NMR (100 MHz, CDCl_3_, δ): 167.1, 159.3, 154.0, 151.5, 147.0, 142.3, 140.9, 133.4, 130.0 (2C), 129.8 (2C), 129.0 (2C), 128.5, 128.2 (2C), 125.3, 52.2, 47.0, 41.6. MS (ESI) (*m*/*z*): [M + H]^+^ 408.11. HRMS (ESI) (*m*/*z*): [M + H]^+^ calcd for C_21_H_19_ClN_5_O_2_^+^, 408.1222; found, 408.1223.

### Methyl 4-(4-((2-Amino-6-chloro-9*H*-purin-9-yl)­methyl)-2-chlorobenzyl)­benzoate (**25**)

To a solution of **21** (0.35 g, 0.8 mmol, 1.0 equiv) and methyl 4-(bromomethyl)­benzoate (0.3 g, 1.2 mmol, 1.0 equiv) in toluene/EtOH 1:3, v/v (16 mL) were added Na_2_CO_3_ (0.18 g, 1.7 mmol, 2 equiv) and Pd­(PPh_3_)_4_ (0.1 g, 0.08 mmol, 0.1 equiv), and the reaction mixture was stirred at 90 °C for 6 h, under a N_2_ atmosphere. The residue was filtered off, and the solid was washed with ethyl acetate. The solution was poured into water and then extracted with ethyl acetate (3 × 10 mL). The combined organic layers were washed with brine (3 × 10 mL), dried with anhydrous Na_2_SO_4_, and filtered, and the solvent was evaporated under vacuum. The crude mixture was then purified by silica gel column chromatography using *n*-hexane/ethyl acetate (1:1, v/v) as an eluent to afford the pure **25** (0.06 g, 13% yield) as an amorphous white solid. ^1^H NMR (400 MHz, CDCl_3_, δ): 8.24 (s, 1H, Ar H), 7.87 (d, *J* = 8.1 Hz, 2H, Ar H), 7.40 (s, 1H, Ar H), 7.36 (d, *J* = 8.2 Hz, 1H, Ar H), 7.31 (d, *J* = 7.8 Hz, 1H, Ar H), 7.19 (d, *J* = 7.6 Hz, 2H, Ar H), 6.95 (s, 2H, -N*H*_2_), 5.28 (s, 2H, −C*H*_2_), 4.12 (s, 2H, −C*H*_2_), 3.82 (s, 3H, -OC*H*_3_). ^13^C NMR (100 MHz, CDCl_3_, δ): 166.8, 159.7, 152.1, 151.3, 146.4, 145.3, 140.7, 135.9, 133.3, 131.5, 130.6 (2C), 129.2 (2C), 128.4 (2C), 127.3, 125.4, 52.5, 48.7, 36.3. MS (ESI) (*m*/*z*): [M + H]^+^ 442.07. HRMS (ESI) (*m*/*z*): [M + H]^+^ calcd for C_21_H_18_Cl_2_N_5_O_2_^+^, 442.0833; found, 442.0834.

### Methyl 5-(4-((2-Amino-4-chloro-7*H*-pyrrolo­[2,3-*d*]­pyrimidin-7-yl)­methyl)­benzyl)­thiophene-2-carboxylate (**26**)

To a solution of **18** (0.29 g, 74 mmol, 1.0 equiv) and methyl 5-(bromomethyl)­thiophene-2-carboxylate (0.21 g, 0.89 mmol, 1.2 equiv) in toluene/EtOH 1:3, v/v (15 mL) were added K_2_CO_3_ (0.2 g, 1.48 mmol, 2 equiv) and Pd­(PPh_3_)_4_ (0.085 g, 0.074 mmol, 0.1 equiv), and the reaction mixture was stirred at 90 °C for 6 h, under a N_2_ atmosphere. The residue was filtered off, and the solid was washed with ethyl acetate. The solution was poured into water and then extracted with ethyl acetate (3 × 10 mL). The combined organic layers were washed with brine (3 × 10 mL), dried with anhydrous Na_2_SO_4_, and filtered, and the solvent was evaporated under vacuum. The crude mixture was then purified by silica gel column chromatography using *n*-hexane/ethyl acetate (6:4, v/v) as an eluent to afford the pure **26** (0.18 g, 59% yield) as an amorphous white solid. ^1^H NMR (400 MHz, CDCl_3_, δ): 7.62 (d, *J* = 3.8 Hz, 1H, Ar H), 7.20 (d, *J* = 7.3 Hz, 2H, Ar H), 7.15 (d, *J* = 8.5 Hz, 2H, Ar H), 6.83 (d, *J* = 3.6 Hz, 1H, Ar H), 6.78 (d, *J* = 4.4 Hz, 1H, Ar H), 6.42 (d, *J* = 4.3 Hz, 1H, Ar H), 5.24 (s, 2H, −C*H*_2_), 5.13 (br s, 2H, -N*H*_2_), 4.12 (s, 2H, −C*H*_2_), 3.83 (s, 3H, -OC*H*_3_). ^13^C NMR (100 MHz, CDCl_3_, δ): 162.6, 158.4, 151.7, 139.1, 135.2, 133.7 (2C), 131.8 (2C), 129.2 (2C), 128.0 (2C), 126.1, 126.0, 110.7, 100.5, 52.0, 47.8, 36.2. MS (ESI) (*m*/*z*): [M + H]^+^ 413.08. HRMS (ESI) (*m*/*z*): [M + H]^+^ calcd for C_20_H_18_Cl_2_N_4_O_2_S^+^, 413.0834; found, 413.0827.

### Methyl 5-(4-((2-Amino-4-chloro-7*H*-pyrrolo­[2,3-*d*]­pyrimidin-7-yl)­methyl)-2-chlorobenzyl)­thiophene-2-carboxylate (**27**)

To a solution of **19** (0.42 g, 1 mmol, 1.0 equiv) and methyl 5-(bromomethyl)­thiophene-2-carboxylate (0.29 g, 1.2 mmol, 1.2 equiv) in toluene/EtOH 1:3, v/v (12 mL) were added K_2_CO_3_ (0.28 g, 2 mmol, 2 equiv) and Pd­(PPh_3_)_4_ (0.12 g, 0.1 mmol, 0.1 equiv), and the reaction mixture was stirred at 90 °C for 6 h, under a N_2_ atmosphere. The residue was filtered off, and the solid was washed with ethyl acetate. The solution was poured into water and then extracted with ethyl acetate (3 × 10 mL). The combined organic layers were washed with brine (3 × 10 mL), dried with anhydrous Na_2_SO_4_, and filtered, and the solvent was evaporated under vacuum. The crude mixture was then purified by silica gel column chromatography using *n*-hexane/ethyl acetate (7:3, v/v) as an eluent to afford the pure **27** (0.13 g, 30% yield) as an amorphous white solid. ^1^H NMR (400 MHz, CDCl_3_, δ): 7.61 (d, *J* = 4.5 Hz, 1H, Ar H), 7.21 (m, 2H, Ar H), 7.04 (d, *J* = 7.7 Hz, 1H, Ar H), 6.83 (d, *J* = 3.8 Hz, 1H, Ar H), 6.80 (d, *J* = 4.5 Hz, 1H, Ar H), 6.44 (d, *J* = 3.6, 1H, Ar H), 5.22 (s, 2H, −C*H*_2_), 5.15 (br s, 2H, -N*H*_2_), 4.23 (s, 2H, −C*H*_2_), 3.83 (s, 3H, -OC*H*_3_). ^13^C NMR (100 MHz, CDCl_3_, δ): 162.7, 158.3, 153.2, 152.8, 149.9, 137.5, 136.9, 134.5, 133.8, 132.0, 131.2, 128.8, 126.6, 126.4, 125.9, 110.8, 100.9, 52.2, 47.3, 33.9. MS (ESI) (*m*/*z*): [M + H]^+^ 447.04. HRMS (ESI) (*m*/*z*): [M + H]^+^ calcd for C_20_H_17_Cl_2_N_4_O_2_S^+^, 447.0444; found, 447.0442.

### Methyl 5-(4-((2-Amino-6-chloro-9*H*-purin-9-yl)­methyl)­benzyl)­thiophene-2-carboxylate (**28**)

To a solution of **20** (0.32 g, 0.83 mmol, 1.0 equiv) and methyl 5-(bromomethyl)­thiophene-2-carboxylate (0.24 g, 1.0 mmol, 1.2 equiv) in toluene/EtOH 1:3, v/v (12 mL) were added Na_2_CO_3_ (0.23 g, 1.66 mmol, 2 equiv) and Pd­(PPh_3_)_4_ (0.1 g, 0.08 mmol, 0.1 equiv), and the reaction mixture was stirred at 90 °C for 6 h, under a N_2_ atmosphere. The residue was filtered off, and the solid was washed with ethyl acetate. The solution was poured into water and then extracted with ethyl acetate (3 × 10 mL). The combined organic layers were washed with brine (3 × 10 mL), dried with anhydrous Na_2_SO_4_, and filtered, and the solvent was evaporated under vacuum. The crude mixture was then purified by silica gel column chromatography using *n*-hexane/ethyl acetate (2:8, v/v) as an eluent to afford the pure **28** (0.1 g, 27% yield) as an amorphous white solid. ^1^H NMR (400 MHz, CDCl_3_, δ): 7.66 (s, 1H, Ar H), 7.60 (m, 3H, Ar H), 7.55 (d, *J* = 3.7 Hz, 1H, Ar H), 7.47 (m, 1H, Ar H), 7.38 (m, 2H, Ar H), 6.71 (d, *J* = 3.8 Hz, 1H, Ar H), 5.15 (s, 2H, −C*H*_2_), 4.05 (s, 2H, −C*H*_2_), 3.76 (s, 3H, -OC*H*_3_).^13^C NMR (100 MHz, CDCl_3_, δ): 162.7, 159.4, 153.9, 151.5, 142.2, 139.8, 133.9, 133.8, 131.9, 129.5 (2C), 128.2 (2C), 126.3, 65.9, 52.1, 46.9, 36.2, 15.4. MS (ESI) (*m*/*z*): [M + H]^+^ 414.07. HRMS (ESI) (*m*/*z*): [M + H]^+^ calcd for C_19_H_17_ClN_5_O_2_S^+^, 414.0786; found, 414.0784.

### Methyl 5-(4-((2-Amino-6-chloro-9*H*-purin-9-yl)­methyl)-2-chlorobenzyl)­thiophene-2-carboxylate (**29**)

To a solution of **21** (0.39 g, 0.93 mmol, 1.0 equiv) and methyl 5-(bromomethyl)­thiophene-2-carboxylate (0.26 g, 1.12 mmol, 1.2 equiv) in toluene/EtOH 1:3, v/v (12 mL) were added Na_2_CO_3_ (0.4 g, 3.7 mmol, 4 equiv) and Pd­(PPh_3_)_4_ (0.11 g, 0.09 mmol, 0.1 equiv), and the reaction mixture was stirred at 90 °C for 6 h, under a N_2_ atmosphere. The residue was filtered off, and the solid was washed with ethyl acetate. The solution was poured into water and then extracted with ethyl acetate (3 × 10 mL). The combined organic layers were washed with brine (3 × 10 mL), dried with anhydrous Na_2_SO_4_, and filtered, and the solvent was evaporated under vacuum. The crude mixture was then purified by silica gel column chromatography using *n*-hexane/ethyl acetate (1:1, v/v) as an eluent to afford the pure **29** (0.24 g, 56% yield) as an amorphous white solid. ^1^H NMR (400 MHz, CDCl_3_, δ): 7.75 (s, 1H, Ar H). 7.62 (d, *J* = 3.8 Hz, 1H, Ar H), 7.30 (d, *J* = 1.7 Hz, 1H, Ar H), 7.24 (d, *J* = 7.8 Hz, 1H, Ar H), 7.11 (dd, *J* = 7.9, 1.7 Hz, 1H, Ar H), 6.81 (d, *J* = 3.6 Hz, 1H, Ar H), 5.21 (s, 2H, −C*H*_2_), 5.12 (s, 2H, -N*H*_2_), 4.25 (s, 2H, −C*H*_2_), 3.63 (s, 3H, -OC*H*_3_). ^13^C NMR (100 MHz, CDCl_3_, δ): 162.7, 159.4, 153.9, 151.8, 149.6, 142.0, 137.6, 136.0, 134.8, 133.8, 132.1, 131.5, 129.0, 126.7, 126.5, 125.4, 52.2, 46.3, 33.9. MS (ESI) (*m*/*z*): [M + H]^+^ 448.03. HRMS (ESI) (*m*/*z*): [M + H]^+^ calcd for C_19_H_16_Cl_2_N_5_O_2_S^+^, 448.0397; found, 448.0399.

### 4-Chloro-7-(4-nitrobenzyl)-7*H*-pyrrolo­[2,3-*d*]­pyrimidin-2-amine (**30**)

To a solution of 4-chloro-7*H*-pyrrolo­[2,3-*d*]­pyrimidin-2-amine (2.0 g, 12 mmol, 1.0 equiv) and 1-(bromomethyl)-4-nitrobenzene (3.8 g, 18 mmol, 1.5 equiv) in dry DMF (15 mL) were added K_2_CO_3_ (6.6 g, 48 mmol, 4 equiv), and the reaction mixture was stirred at r.t., overnight. The reaction mixture was extracted with ethyl acetate (3 × 10 mL), after being poured into water. The combined organic layers were washed with brine (3 × 10 mL), dried with anhydrous Na_2_SO_4_, and filtered, and the solvent was evaporated under vacuum. The crude mixture was then purified by silica gel column chromatography using *n*-hexane/ethyl acetate (6:4, v/v) as an eluent to afford the pure **30** (2.5 g, 70% yield) as an amorphous yellow solid. ^1^H NMR (400 MHz, DMSO-*d*_6_, δ): 8.19 (d, ^3^ *J*_H,H_ = 8.7 Hz, 2H, Ph H-3,5), 7.37 (d, ^3^ *J*_H,H_ = 8.7 Hz, 2H, Ph H-2,6), 7.25 (d, ^3^ *J*_H,H_ = 3.8 Hz, 1H, Pyr H-5), 6.69 (br s, 2H, –NH_2_), 6.40 (d, ^3^ *J*_H,H_ = 3.8 Hz, 1H, Pyr H-4), 5.42 (s, 2H, –CH_2_). ^13^C NMR (100 MHz, DMSO-*d*_6_, δ): 159.5, 153.6, 151.4, 146.8, 145.5, 127.9, 126.4, 123.8, 108.6, 99.3, 46.5, 40.1, 39.9, 39.7, 39.5, 39.3, 39.1, 38.9. HRMS (ESI) (*m*/*z*): [M + H]^+^ calcd for C_13_H_11_ClN_5_O_2_^+^, 304.0596; found, 304.0585.

### 7-(4-Aminobenzyl)-4-chloro-7*H*-pyrrolo­[2,3-*d*]­pyrimidin-2-amine (**31**)

A stirred suspension of **30** (550 mg, 1.81 mmol, 1.0 equiv), iron powder (606 mg, 10.9 mmol, 6 equiv), and NH_4_Cl (580 mg, 10.9 mmol, 6 equiv) in ethanol/water 1:1 v/v (20 mL) was refluxed for 2 h. The reaction mixture was filtered through a short pad of Celite, and the solvent was evaporated under vacuum. The resulting crude was then treated with a sat. solution of K_2_CO_3_, and the precipitate was filtered and dried to afford pure **31** as an orange amorphous solid. ^1^H NMR (400 MHz, DMSO-*d*_6_, δ): 7.10 (d, ^3^ *J*_H,H_ = 3.6 Hz, 1H, Pyr H-5), 6.93 (d, ^3^ *J*_H,H_ = 8.3 Hz, 2H, Ph H-2,6), 6.62 (s, 2H, Pyr NH_2_), 6.49 (d, ^3^ *J*_H,H_ = 8.7 Hz, 2H, Ph H-3,5), 6.27 (d, ^3^ *J*_H,H_ = 3.6 Hz, 1H, Pyr H-4), 5.03 (s, 2H, –CH_2_), 5.03 (s, 2H, Ph-N*H*_2_). ^13^C NMR (100 MHz, DMSO-*d*_6_, δ): 159.3, 153.3, 151.0, 148.2, 128.5 (2C), 126.1 (2C), 124.3, 113.7, 108.6, 98.5, 46.8. HRMS (ESI) (*m*/*z*): [M + H]^+^ calcd for C_13_H_13_ClN_5_^+^, 274.0854; found, 274.0860.

### Methyl 2-(((4-((2-Amino-4-chloro-7*H*-pyrrolo­[2,3-*d*]­pyrimidin-7-yl)­methyl)­phenyl)­amino)­methyl)­oxazole-4-carboxylate (**32**)

To a solution of **31** (0.27 g, 1.0 mmol, 1.0 equiv) and methyl 2-(bromomethyl)­oxazole-4-carboxylate (0.33 g, 1.5 mmol, 1.5 equiv) in dry DMF (7 mL) was added triethylamine (0.4 mL, 3.0 mmol, 3.0 equiv), and the reaction mixture was stirred at r.t., overnight. The reaction mixture was extracted with ethyl acetate (3 × 10 mL), after being poured into water. The combined organic layers were washed with brine (3 × 10 mL), dried with anhydrous Na_2_SO_4_, and filtered, and the solvent was evaporated under vacuum. The crude mixture was then purified by silica gel column chromatography using *n*-hexane/ethyl acetate (6:4, v/v) as an eluent to afford the pure **32** (200 mg, 49% yield) as an amorphous yellow solid. ^1^H NMR (400 MHz, MeOD, δ): 8.36 (s, 1H, Isoxazole H), 7.17 (d, ^3^ *J*_H,H_ = 8.2 Hz, 2H, Ph H), 7.09 (d, ^3^ *J*_H,H_ = 3.6 Hz, 1H, Ar–H), 6.70 (d, ^3^ *J*_H,H_ = 8.2 Hz, 2H, Ph H), 6.44 (d, ^3^ *J*_H,H_ = 3.6 Hz, 1H, Ar–H), 5.20 (s, 2H, ArCH_2_), 4.51 (s, 2H, ArCH_2_), 3.69 (s, 3H, OCH_3_). ^13^C NMR (100 MHz, MeOD, δ): 170.1, 162.6, 160.8, 155.5, 153.1, 148.9, 142.1, 135.0, 130.9, 127.6 (2C), 127.5 (2C), 113.4, 112.3, 100.1, 51.1, 48.1, 41.2. HRMS (ESI) (*m*/*z*): [M + H]^+^ calcd for C_19_H_18_ClN_6_O_3_^+^, 413.1124; found, 413.1121.

### Methyl 5-(((4-((2-Amino-4-chloro-7*H*-pyrrolo­[2,3-*d*]­pyrimidin-7-yl)­methyl)­phenyl)­amino)­methyl)­thiophene-2-carboxylate (**33**)

To a solution of **31** (0.29 g, 1.06 mmol, 1.0 equiv) and methyl 5-(bromomethyl)­thiophene-2-carboxylate (0.275 g, 1.16 mmol, 1.1 equiv) in dry DMF (7 mL) was added K_2_CO_3_ (0.366 g, 2.65 mmol, 2.5 equiv), and the reaction mixture was stirred at r.t., overnight. The reaction mixture was extracted with ethyl acetate (3 × 10 mL), after being poured into water. The combined organic layers were washed with brine (3 × 10 mL), dried with anhydrous Na_2_SO_4_, and filtered, and the solvent was evaporated under vacuum. The crude mixture was then purified by silica gel column chromatography using *n*-hexane/ethyl acetate (6:4, v/v) as an eluent to afford the pure **33** (190 mg, 42% yield) as an amorphous yellow solid. ^1^H NMR (400 MHz, CD_2_Cl_2_, δ): 7.68 (d, ^3^J_H,H_ = 3.8 Hz, 1H, Thiophene H), 7.10 (d, ^3^ *J*_H,H_ = 8.2 Hz, 2H, Ph H), 7.04 (d, ^3^J_H,H_ = 3.8 Hz, 1H, Thiophene H), 6.91 (d, ^3^ *J*_H,H_ = 3.6 Hz, 1H, Ar H), 6.64 (d, ^3^ *J*_H,H_ = 8.2 Hz, 2H, Ph H), 6.41 (d, ^3^ *J*_H,H_ = 3.6 Hz, 1H, Ar H), 5.16 (br s, 4H, ArCH_2_ and -N*H*_2_), 4.56 (s, 2H, ArCH_2_), 3.86 (s, 3H, OCH_3_). ^13^C NMR (100 MHz, DMSO-*d*_6_, δ): 163.5, 159.2, 155.1, 152.0, 145.6, 135.2, 129.6, 129.3, 128.3, 127.0 (2C), 126.1 (2C), 114.4, 114.6, 111.6, 101.6, 50.9, 45.7, 27.1. HRMS (ESI) (*m*/*z*): [M + H]^+^ calcd for C_20_H_19_ClN_5_O_2_S^+^, 428.0943; found, 428.0959.

### Methyl 2-(((4-((2-Amino-4-chloro-7*H*-pyrrolo­[2,3-*d*]­pyrimidin-7-yl)­methyl)­phenyl)­amino)­methyl)­thiazole-5-carboxylate (**34**)

To a solution of **31** (0.15 g, 0.55 mmol, 1.0 equiv) and methyl 5-(bromomethyl)­thiophene-2-carboxylate (0.144 g, 0.61 mmol, 1.1 equiv) in dry DMF (7 mL) was added K_2_CO_3_ (0.190 g, 1.37 mmol, 2.5 equiv), and the reaction mixture was stirred at r.t., overnight. The reaction mixture was extracted with ethyl acetate (3 × 10 mL), after being poured into water. The combined organic layers were washed with brine (3 × 10 mL), dried with anhydrous Na_2_SO_4_, and filtered, and the solvent was evaporated under vacuum. The crude mixture was then purified by silica gel column chromatography using *n*-hexane/ethyl acetate (6:4, v/v) as an eluent to afford the pure **34** (118 mg, 51% yield) as an amorphous yellow solid. ^1^H NMR (400 MHz, CDCl_3_, δ): 8.33 (s, 1H, Thiazole H), 7.05 (d, ^3^ *J*_H,H_ = 8.2 Hz, 2H, Ph H), 7.00 (d, ^3^ *J*_H,H_ = 3.6 Hz, 1H, Ar–H), 6.59 (d, ^3^ *J*_H,H_ = 8.2 Hz, 2H, Ph H), 6.37 (d, ^3^ *J*_H,H_ = 3.6 Hz, 1H, Ar–H), 5.07 (s, 2H, ArCH_2_), 5.01 (br s, 2H, –NH_2_), 4.55 (s, 2H, ArCH_2_), 3.78 (s, 3H, OCH_3_). ^13^C NMR (100 MHz, CDCl_3_, δ): 159.6, 153.6, 151.2, 148.0, 143.1, 123.7, 129.9, 128.5, 127.1 (2C), 126.7 (2C), 113.7, 111.7, 107.5, 100.1, 53.0, 48.1, 47.4. HRMS (ESI) (*m*/*z*): [M + H]^+^ calcd for C_19_H_18_ClN_6_O_2_S^+^, 429.0895; found, 429.0891.

### Methyl 4-(((4-((2-Amino-4-chloro-7*H*-pyrrolo­[2,3-*d*]­pyrimidin-7-yl)­methyl)­phenyl)­amino)­methyl)­benzoate (**35**)

To a solution of **31** (0.40 g, 1.46 mmol, 1.0 equiv) and methyl 4-(bromomethyl)­benzoate (0.50 g, 2.19 mmol, 1.5 equiv) in dry DMF (10 mL) was added triethylamine (0.6 mL, 4.38 mmol, 3.0 equiv), and the reaction mixture was stirred at r.t., overnight. The reaction mixture was extracted with ethyl acetate (3 × 10 mL), after being poured into water. The combined organic layers were washed with brine (3 × 10 mL), dried with anhydrous Na_2_SO_4_, and filtered, and the solvent was evaporated under vacuum. The crude mixture was then purified by silica gel column chromatography using *n*-hexane/ethyl acetate (7:3, v/v) as an eluent to afford the pure **35** (308 mg, 50% yield) as an amorphous yellow solid. ^1^H NMR (400 MHz, MeOD, δ): 7.80 (d, 2H, ^3^ *J*_H,H_ = 8.5 Hz, 2H, Ph H), 7.50 (d, 2H, ^3^ *J*_H,H_ = 8.2 Hz, 2H, Ph H), 7.19 (d, 2H, ^3^ *J*_H,H_ = 8.2 Hz, 2H, Ph H), 6.99 (d, 1H, ^3^ *J*_H,H_ = 3.7 Hz, Ar H), 6.61 (d, 2H, ^3^ *J*_H,H_ = 8.2 Hz, 2H, Ph H), 6.43 (d, 1H, ^3^ *J*_H,H_ = 3.7 Hz, Ar H), 5.21 (s, 2H, ArCH_2_), 4.47 (s, 2H, ArCH_2_), 3.87 (s, 3H, OCH_3_). ^13^C NMR (100 MHz, MeOD, δ): 170.1, 161.2, 155.4, 153.1, 148.7, 143.2, 132.4 (2C), 129.9 (2C), 127.9 (2C), 127.6 (2C), 127.1 (2C), 115.9, 111.0, 100.2, 51.8, 50.3, 48.1. HRMS (ESI) (*m*/*z*): [M + H]^+^ calcd for C_22_H_21_ClN_5_O_2_^+^, 422.1379; found, 422.1371.

### *Tert*-butyl 5-methyl-1*H*-indole-1-carboxylate (**36**)

To a solution of 5-methylindole (2.00 g, 15.2 mmol, 1.0 equiv) in dry acetonitrile (16 mL) were added DMAP (279 mg, 2.3 mmol, 0.15 equiv) and Boc_2_O (4.00 g, 18.3 mmol, 1.2 equiv), and the mixture was left stirring at r.t., overnight. The reaction mixture was concentrated under reduced pressure, and the residue was then diluted with ethyl acetate. The organic layer was washed with saturated aq. solution of NH_4_Cl (3 × 10 mL) and brine (3 × 10 mL), dried with anhydrous Na_2_SO_4_, filtered, and concentrated under reduced pressure to afford **36** as a yellow liquid (3.42 g, 96% yield). Spectral data match with the ones previously reported.^55^ ^1^H NMR (400 MHz, CDCl_3_, δ): 8.00 (d, *J* = 7.3 Hz, 1H, Ind H), 7.55 (d, *J* = 3.4 Hz, 1H, Ind H), 7.35 (t, *J* = 0.7 Hz, 1H, Ind H), 7.13 (dd, *J* = 8.5, 1.3 Hz, 1H, Ind H), 6.49 (d, *J* = 3.7 Hz, 1H, Ind H), 2.44 (s, 3H, Ar–CH_3_), 1.67 (s, 9H, –CH_3_ Boc).

### *Tert*-butyl 5-(bromomethyl)-1*H*-indole-1-carboxylate (**37**)

To a solution of **36** (1.60 g, 6.9 mmol, 1.0 equiv) in CCl_4_ (15 mL) were added NBS (1.19 g, 6.9 mmol, 1.0 equiv) and AIBN (0.70 g, 4.2 mmol, 0.6 equiv), and the mixture was left stirring at room temperature for 1 h. After completion of the reaction, the mixture was filtered and diluted with DCM. The organic layer was washed with brine (3 × 10 mL), dried with anhydrous Na_2_SO_4_, filtered, and concentrated under reduced pressure. The crude mixture was then purified by silica gel column chromatography using *n*-hexane/ethyl acetate (98:2, v/v) as an eluent to afford the pure **37** (996 mg, 46% yield) as a yellow liquid. Spectral data match with the ones previously reported.^55^ ^1^H NMR (400 MHz, CDCl_3_, δ): 8.11 (d, *J* = 8.4 Hz, 1H, Ind H), 7.61 (d, *J* = 3.6 Hz, 1H, Ind H), 7.59 (d, *J* = 1.5 Hz, 1H, Ind H), 7.35 (dd, *J* = 8.6, 1.8 Hz, 1H, Ind H), 6.55 (d, *J* = 3.7 Hz, 1H, Ind H), 4.64 (s, 2H, –CH_2_–Br), 1.67 (s, 9H, –CH_3_ Boc).

### *Tert*-butyl 5-((2-Amino-4-chloro-7*H*-pyrrolo­[2,3-*d*]­pyrimidin-7-yl)­methyl)-1*H*-indole-1-carboxylate (**38**)

To a solution of **37** (1.00 g, 3.22 mmol, 1.2 equiv) and 4-chloro-7*H*-pyrrolo­[2,3-*d*]­pyrimidin-2-amine (0.456 g, 2.7 mmol, 1.0 equiv) in dry DMF (7 mL) was added K_2_CO_3_ (3.10 g, 15.6 mmol, 8 equiv), and the reaction mixture was stirred at 40 °C for 24 h. The reaction mixture was extracted with ethyl acetate (3 × 10 mL), after being poured into water. The combined organic layers were washed with brine (3 × 10 mL), dried with anhydrous Na_2_SO_4_, and filtered, and the solvent was evaporated under vacuum. The crude mixture was then purified by silica gel column chromatography using *n*-hexane/ethyl acetate (7:3, v/v) as an eluent to afford the pure **38** (630 mg, 58% yield) as an amorphous white solid. Spectral data match with those previously reported.^57^ ^1^H NMR (400 MHz, CDCl_3_, δ): 8.08 (d, *J* = 8.3 Hz, 1H, Ind H), 7.59 (d, *J* = 3.5 Hz, 1H, Ind H), 7.37 (s, 1H, Ind H), 7.17 (d, *J* = 8.6 Hz, 1H, Ind H), 6.81 (d, *J* = 3.7 Hz, 1H, Ar H), 6.51 (d, *J* = 3.7 Hz, 1H, Ind H), 6.38 (d, *J* = 3.7 Hz, 1H, Ar H), 5.32 (s, 2H, Ar–CH_2_−), 5.00 (br s, 2H, –NH_2_), 1.65 (s, 9H, –CH_3_ Boc).

### 7-((1*H*-Indol-5-yl)­methyl)-4-chloro-7*H*-pyrrolo­[2,3-*d*]­pyrimidin-2-amine (**39**)

A solution of **38** (630 mg, 1.58 mmol) in TFE (15 mL) was refluxed for 24 h. The solvent was evaporated under vacuum, and the remaining crude was then purified by silica gel column chromatography using *n*-hexane/ethyl acetate (6:4, v/v) as an eluent to afford the pure **39** (244 mg, 52% yield) as an amorphous white solid. Spectral data match with those ones previously reported.^57^ ^1^H NMR (400 MHz, CDCl_3_, δ): 11.09 (s, 1H, Ind –NH), 7.39 (s, 1H, Ar–H), 7.33 (d, *J* = 8.7 Hz, 1H, Ar–H), 7.33 (d, *J* = 3.1 Hz, 1H, Ar–H), 7.17 (d, *J* = 3.6 Hz, 1H, Ar–H), 7.01 (dd, *J* = 8.4, 1.3 Hz, 1H, Ar–H), 6.68 (s, 2H, –NH_2_), 6.37 (s, 1H, Ar–H), 6.30 (d, *J* = 3.6 Hz, 1H, Ar–H), 5.28 (s, 2H, –CH_2_–Ar).

### Methyl 5-((5-((2-Amino-4-chloro-7*H*-pyrrolo­[2,3-*d*]­pyrimidin-7-yl)­methyl)-1*H*-indol-1-yl)­methyl)­thiophene-2-carboxylate (**40**)

To a solution of **39** (280 mg, 0.94 mmol, 1.0 equiv) and methyl 5-(bromomethyl)­thiophene-2-carboxylate (331 mg, 1.41 mmol, 1.5 equiv) in dry ACN (8 mL) was added Cs_2_CO_3_ (0.92 g, 2.82 mmol, 3 equiv), and the reaction mixture was stirred at 75 °C for 5 h. The reaction mixture was extracted with ethyl acetate (3 × 10 mL), after being poured into water. The combined organic layers were washed with brine (3 × 10 mL), dried with anhydrous Na_2_SO_4_, and filtered, and the solvent was evaporated under vacuum. The crude mixture was then purified by silica gel column chromatography using DCM/MeOH (98:2, v/v) as an eluent to afford the pure **40** (80 mg, 20% yield) as an amorphous white solid. ^1^H NMR (400 MHz, CDCl_3_, δ): 7.60 (d, *J* = 3.8 Hz, 1H, Ind H), 7.48 (s, 1H, Ind H), 7.26 (d, *J* = 8.5 Hz, 1H, Ind H), 7.15 (d, *J* = 3.2 Hz, 1H, Ar H), 7.08 (dd, *J* = 8.5, 1.5 Hz, 1H, Ind H), 6.84 (d, *J* = 3.8 Hz, 1H, Ind H), 6.83 (d, *J* = 3.7 Hz, 1H, Ar H), 6.51 (dd, *J* = 3.2, 0.6 Hz, 1H, Ar H), 6.37 (d, *J* = 3.7 Hz, 1H, Ar H), 5.43 (s, 2H, –CH_2_–Ar), 5.32 (s, 2H, –CH_2_–Ar), 5.04 (br s, 2H, –NH_2_), 3.82 (s, 3H, –CH_3_). ^13^C NMR (100 MHz, CDCl_3_) 162.5, 159.4, 151.4, 151.2, 147.4, 134.0, 133.5, 132.1, 130.9, 128.9, 128.4, 128.1, 122.8, 120.1, 118.1, 115.1, 108.0, 100.9, 99.3, 61.2, 58.7, 51.5. HRMS (ESI) (*m*/*z*): [M + H]^+^ calcd for C_22_H_19_ClN_5_O_2_S^+^, 452.0943; found, 452.0956.

### Ethyl 4-(3-Oxopropyl)­benzoate (**41**)

To a solution of ethyl 4-iodobenzoate (2.0 g, 7.2 mmol, 1.0 equiv) in dry DMF (15 mL) were added benzyltriethylammonium chloride (2.3 g, 7.2 mmol, 1.0 equiv), NaHCO_3_ (1.8 g, 21.6 mmol, 5.0 equiv), allyl alcohol (1.47 mL, 21.6 mmol, 5.0 equiv), and Pd­(OAc)_2_ (64 mg, 0.288 mmol, 0.04 equiv). The reaction mixture was stirred at 80 °C for 3 h. After cooling to room temperature, the mixture was poured into water and extracted with DCM (3 × 10 mL), and the combined organic layers were washed with brine (3 × 10 mL), dried with anhydrous Na_2_SO_4_, and filtered. The solvent was evaporated under vacuum, and the crude was purified by silica gel column chromatography using *n*-hexane/ethyl acetate (8:2, v/v) as an eluent to afford the pure **41** (1.0 g, 67% yield) as a yellow oil. ^1^H NMR (400 MHz, CDCl_3_, δ): 9.82 (t, ^3^ *J*_H,H_ = 1.2, 1H, −C*H*O), 7.97 (d, ^3^ *J*_H,H_ = 8.2 Hz, 2H, Ph H-2,6), 7.26 (d, ^3^ *J*_H,H_ = 8.2 Hz, 2H, Ph H-3,5), 4.36 (q, ^3^ *J*_H,H_ = 7.1 Hz, 2H, -OC*H*_2_), 3.01 (t, ^3^ *J*_H,H_ = 7.4 Hz, 2H, H-1), 2.81 (tt, ^3^ *J*_H,H_ = 7.4 Hz, ^3^J_H,H_ = 1.2 Hz 2H, H-2), 1.36 (t, ^3^ *J*_H,H_ = 7.1 Hz, 3H, CH_2_–C*H*_3_). ^13^C NMR (100 MHz, CDCl_3_, δ): 201.5, 140.5, 138.2, 129.2, 128.6, 127.1 (2C), 125.4 (2C), 45.3, 28.1, 21.4. MS (ESI) (*m*/*z*): [M + H]^+^ 207.11. HRMS (ESI) (*m*/*z*): [M + H]^+^ calcd for C_12_H_15_O_3_^+^, 207.1016; found, 207.1020.

### Ethyl 4-(2-Iodo-3-oxopropyl)­benzoate (**42**)

To a solution of **41** (0.7 g, 3.4 mmol, 1.0 equiv) in dry DCM (8 mL) were added l-proline (78 mg, 0.68 mmol, 0.2 equiv) and NIS (1.0 g, 5.1 mmol, 1.5 equiv), and the mixture was left stirring at room temperature for 10 min. After completion of the reaction by TLC analysis, ethyl acetate was added, and the mixture was washed with brine (3 × 10 mL), dried with anhydrous Na_2_SO_4_, and filtered. The solvent was evaporated under vacuum affording the pure compound **42** (1.0 g, 88% yield) as a yellow oil. ^1^H NMR (400 MHz, CDCl_3_, δ): 9.32 (d, ^3^ *J*_H,H_ = 1.4, 1H, −C*H*O), 8.01 (d, ^3^ *J*_H,H_ = 8.2 Hz, 2H, Ph H-2,6), 7.27 (d, ^3^ *J*_H,H_ = 8.2 Hz, 2H, Ph H-3,5), 4.72 (t, ^3^ *J*_H,H_ = 1.4, 1H, −C*H*–I), 4.37 (q, ^3^ *J*_H,H_ = 7.1 Hz, 2H, -OC*H*_2_), 3.55–3.24 (m, 2H, −C*H*_2_Ar), 1.39 (t, ^3^ *J*_H,H_ = 7.1 Hz, 3H, CH_2_–C*H*_3_). ^13^C NMR (100 MHz, CDCl_3_, δ): 190.8, 166.4, 143.2, 130.2, 129.7 (2C), 128.4 (2C), 61.2, 38.4, 35.2, 14.5. MS (ESI) (*m*/*z*): [M + H]^+^ 333.0. HRMS (ESI) (*m*/*z*): [M + H]^+^ calcd for C_12_H_14_IO_3_^+^, 332.9983; found, 332.9986.

### Ethyl 4-((2-Amino-4-oxo-4,7-dihydro-3*H*-pyrrolo­[2,3-*d*]­pyrimidin-5-yl)­methyl)­benzoate (**43**)

To a suspension of **42** (1.12 g, 3.37 mmol, 1.0 equiv) and sodium acetate (0.6 g, 6.74 mmol, 2.0 equiv) in acetonitrile/water (1:1, v/v 12 mL) was added 2,4-diamino-6-hydroxypyrimidine (0.47 g, 3.71 mmol, 1.1 equiv), and the reaction mixture was stirred at r.t., overnight. The precipitate was collected by filtration, washed with water/acetonitrile (1:1, v/v), and dried to afford **43** as a fluffy amorphous yellow solid (0.66 g, 60% yield). ^1^H NMR (400 MHz, DMSO-*d*_6_, δ): 10.73 (br s, 1H, -N^7^ *H*), 10.11 (br s, 1H, - N^3^ *H*), 7.83 (d, ^3^ *J*_H,H_ = 8.2 Hz, 2H, Ph H-2,6), 7.41 (d, ^3^ *J*_H,H_ = 8.2 Hz, 2H, Ph H-3,5), 6.35 (s, 1H, Pyr H-6), 5.99 (br s, 2H, -N*H*_2_), 4.28 (q, ^3^ *J*_H,H_ = 7.2 Hz, 2H, -OC*H*_2_), 4.00 (s, 2H, −C*H*_2_Ar), 1.30 (t, ^3^J_H,H_ = 7.2 Hz, 3H, CH_2_–C*H*_3_). ^13^C NMR (100 MHz, DMSO-*d*_6_, δ): 165.7, 159.2, 152.2, 151.2, 148.1, 128.9 (2C), 128.8 (2C), 127.2, 116.5, 114.2, 98.6, 60.4, 31.7, 14.2. MS (ESI) (*m*/*z*): [M + H]^+^ 313.24. HRMS (ESI) (*m*/*z*): [M + H]^+^ calcd for C_16_H_17_N_4_O_3_^+^, 313.1296; found, 313.1299.

### Ethyl 4-((2-Amino-4-chloro-7*H*-pyrrolo­[2,3-*d*]­pyrimidin-5-yl)­methyl)­benzoate (**44**)

To a suspension of **43** (0.63 g, 2.02 mmol, 1.0 equiv), benzyltriethylammonium chloride (0.92 g, 4.04 mmol, 2.0 equiv), and *N*,*N*-dimethylaniline (0.53 mL, 0.48 g, 4.04 mmol, 2.0 equiv) in dry acetonitrile (10 mL) was slowly added phosphorus­(V) oxychloride (0.9 mL, 1.55 g, 10.1 mmol, 5.0 equiv) under a nitrogen atmosphere. The reaction mixture was stirred at 100 °C for 3 h and then concentrated. The residue was poured into ice water and neutralized with a saturated solution of NaHCO_3_. The mixture was extracted with DCM (3 × 10 mL), and the organic layer was washed with brine (3 × 10 mL), dried with anhydrous Na_2_SO_4_, and evaporated under reduced pressure. The crude mixture was then purified by silica gel column chromatography using *n*-hexane/ethyl acetate (3:7, v/v) as an eluent to afford the pure **44** (0.15 g, 23% yield) as an amorphous gray solid. ^1^H NMR (400 MHz, DMSO-*d*_6_, δ): 11.31 (br s, 1H, -N^7^ *H*), 7.86 (d, ^3^ *J*_H,H_ = 8.2 Hz, 2H, Ph H-2,6), 7.32 (d, ^3^ *J*_H,H_ = 8.2 Hz, 2H, Ph H-3,5), 6.87 (s, 1H, Pyr H-6), 6.52 (br s, 2H, –NH_2_), 4.28 (q, ^3^ *J*_H,H_ = 7.2 Hz, 2H, -OC*H*_2_), 4.12 (s, 2H, −C*H*_2_Ar), 1.30 (t, ^3^ *J*_H,H_ = 7.2 Hz, 3H, CH_2_–C*H*_3_). ^13^C NMR (100 MHz, DMSO-*d*_6_, δ): 165.7, 159.2, 152.2, 151.2, 148.1, 128.9 (2C), 128.8 (2C), 127.2, 116.5, 114.2, 98.6, 60.4, 31.7, 14.2. MS (ESI) (*m*/*z*): [M + H]^+^ 331.1. HRMS (ESI) (*m*/*z*): [M + H]^+^ calcd for C_16_H_16_N_4_O_2_Cl^+^, 331.0957; found, 331.0955.

### Ethyl 4-((2-Amino-7-(benzo­[*d*]­[1,3]­dioxol-5-ylmethyl)-4-chloro-7*H*-pyrrolo­[2,3-*d*]­pyrimidin-5-yl)­methyl)­benzoate (**45**)

To a solution of **44** (0.15 g, 0.46 mmol, 1.0 equiv) and 5-(chloromethyl)­benzo­[*d*]­[1,3]­dioxole (0.118 g, 0.69 mmol, 1.5 equiv) in dry DMF (7 mL) was added K_2_CO_3_ (0.156 g, 1.15 mmol, 2.5 equiv), and the reaction mixture was stirred at r.t., overnight. The reaction mixture was extracted with ethyl acetate (3 × 10 mL), after being poured into water. The combined organic layers were washed with brine (3 × 10 mL), dried with anhydrous Na_2_SO_4_, and filtered, and the solvent was evaporated under vacuum. The crude mixture was then purified by silica gel column chromatography using *n*-hexane/ethyl acetate (7:3, v/v) as an eluent to afford the pure **45** (0.15 g, 70% yield) as an amorphous white solid. ^1^H NMR (400 MHz, DMSO-*d*_6_, δ): 7.87 (d, ^3^ *J*_H,H_ = 8.2 Hz, 2H, Ph H-2,6), 7.32 (d, ^3^ *J*_H,H_ = 8.2 Hz, 2H, Ph H-3,5), 6.92 (s, 1H, Pyr H-6), 6.85­(d, ^3^ *J*_H,H_ = 7.2 Hz, 1H, Ar H), 6.83 (s, 1H, Ar H), 6.66 (d, ^3^ *J*_H,H_ = 7.2 Hz, 1H, Ar H), 6.67 (br s, 2H, -N*H*_2_), 5.97 (s, 2H, -OC*H*_2_O), 5.09 (s, 2H, −C*H*_2_N), 4.29 (q, ^3^ *J*_H,H_ = 7.2 Hz, 2H, -OC*H*_2_), 4.12 (s, 2H, −C*H*_2_Ar), 1.30 (t, ^3^ *J*_H,H_ = 7.2 Hz, 3H, CH_2_–C*H*_3_). ^13^C NMR (100 MHz, DMSO-*d*_6_, δ): 165.7, 159.3, 154.0, 151.1, 147.4, 146.6, 131.5, 129.2 (2C), 128.6 (2C), 127.7 (2C), 120.7 (2C), 112.1, 108.3, 107.9, 107.2, 101.0, 60.5, 46.5, 31.2, 14.2. MS (ESI) (*m*/*z*): [M + H]^+^ 465.21. HRMS (ESI) (*m*/*z*): [M + H]^+^ calcd for C_24_H_22_ClN_4_O_4_^+^, 465.1325; found, 465.1321.

### Ethyl 4-((2-Amino-7-((6-bromobenzo­[*d*]­[1,3]­dioxol-5-yl)­methyl)-4-chloro-7*H*-pyrrolo­[2,3-*d*]­pyrimidin-5-yl)­methyl)­benzoate (**46**)

To a solution of **44** (57 mg, 0.17 mmol, 1.0 equiv) and 5-bromo-6-(bromomethyl)­benzo­[*d*]­[1,3]­dioxole (81 mg, 0.27 mmol, 1.6 equiv) in dry DMF (5 mL) was added K_2_CO_3_ (71 mg, 0.52 mmol, 3.0 equiv), and the reaction mixture was stirred at r.t., overnight. The reaction mixture was extracted with ethyl acetate (3 × 10 mL), after being poured into water. The combined organic layers were washed with brine (3 × 10 mL), dried with anhydrous Na_2_SO_4_, and filtered, and the solvent was evaporated under vacuum. The crude mixture was then purified by column chromatography using *n*-hexane/ethyl acetate (7:3, v/v) as an eluent to afford the pure **46** (80 mg, 87% yield) as an amorphous white solid. ^1^H NMR (400 MHz, DMSO-*d*_6_, δ): 7.86 (d, ^3^ *J*_H,H_ = 8.3 Hz, 2H, Ph H-2,6), 7.33 (d, ^3^ *J*_H,H_ = 8.3 Hz, 2H, Ph H-3,5), 7.26 (s, 1H, Ar–H), 6.89 (s, 1H, Pyr H-6), 6.69 (br s, 2H, -N*H*_2_), 6.39 (s, 1H, Ar–H), 6.04 (s, 2H, -OC*H*_2_O), 5.15 (s, 2H, Ar–C*H*_2_N), 4.29 (q, *J* = 7.1 Hz, 2H, −C*H*_2_CH_3_), 4.15 (s, 2H, −C*H*_2_Ar), 1.30 (t, *J* = 7.1 Hz, 3H, −CH_2_C*H*_3_). ^13^C NMR (100 MHz, DMSO-*d*_6_, δ): 165.6, 159.3, 154.2, 151.2, 147.8, 147.4, 146.6, 129.3 (2C), 129.1, 128.6 (2C), 127.6, 124.5, 112.6, 112.5, 112.3, 108.5, 107.1, 102.1, 60.5, 47.1, 31.1, 14.1. MS (ESI) (*m*/*z*): [M + H]^+^ 544.90. HRMS (ESI) (*m*/*z*): [M + H]^+^ calcd for C_24_H_21_BrClN_4_O_4_^+^, 543.0430; found, 543.0424.

### Ethyl 4-((2-Amino-4-chloro-7-((4-methoxy-3,5-dimethylpyridin-2-yl)­methyl)-7H-pyrrolo­[2,3-*d*]­pyrimidin-5-yl)­methyl)­benzoate (**47**)

To a solution of **44** (0.26 g, 0.8 mmol, 1.0 equiv) and 2-(chloromethyl)-4-methoxy-3,5-dimethylpyridine (0.22 g, 1.2 mmol, 1.5 equiv) in dry DMF (10 mL) was added K_2_CO_3_ (0.28 g, 2.0 mmol, 2.5 equiv), and the reaction mixture was stirred at r.t., overnight. The reaction mixture was extracted with ethyl acetate (3 × 10 mL), after being poured into water. The combined organic layers were washed with brine (3 × 10 mL), dried with anhydrous Na_2_SO_4_, and filtered, and the solvent was evaporated under vacuum. The crude mixture was then purified by column chromatography using *n*-hexane/ethyl acetate (3:7, v/v) as an eluent to afford the pure **47** (0.2 g, 52% yield) as an amorphous white solid. ^1^H NMR (400 MHz, CDCl_3_, δ): 8.17 (s, 1H, Pyridine), 7.91 (d, ^3^ *J*_H,H_ = 7.9 Hz, 2H, Ph H-2,6), 7.23 (d, ^3^ *J*_H,H_ = 7.9 Hz, 2H, Ph H-3,5), 6.54 (s, 1H, Pyrrole H-6), 5.26 (s, 2H, −C*H*_2_N), 5.12 (br s, 2H, -N*H*_2_), 4.34 (q, ^3^ *J*_H,H_ = 7.2 Hz, 2H, -OC*H*_2_), 4.14 (s, 2H, −C*H*_2_Ar), 3.71 (s, 3H, OCH_3_), 2.22 (s, 3H, Pyridine–C*H*_3_), 2.17 (s, 3H, Pyridine–C*H*_3_), 1.36 (t, ^3^ *J*_H,H_ = 7.2 Hz, 3H, CH_2_–C*H*_3_). ^13^C NMR (100 MHz, CDCl_3_, δ): 166.7, 164.4, 158.6, 154.2, 153.9, 152.4, 149.5, 146.0, 129.7 (2C), 129.4 (2C), 128.7, 128.6, 128.5, 126.1, 125.2, 113.6, 109.2, 60.8, 47.4, 32.1, 14.4, 13.4, 11.0. MS (ESI) (*m*/*z*): [M + H]^+^ 465.21. HRMS (ESI) (*m*/*z*): [M + H]^+^ calcd for C_25_H_27_ClN_5_O_3_^+^, 480.1797; found, 480.1798.

### Biology

#### In Vitro Assays on Hsp90

IC_50_ evaluations against Hsp90 were carried out as described in our previous studies.^55,58^ Briefly, the compounds were initially dissolved at a concentration of 10 mM in 100% DMSO and then serially diluted in a 1:3 ratio across ten concentrations, starting from 100 μM. The resulting solutions were then added to a reaction mixture containing human recombinant Hsp90α at a 30 nM concentration with His-tag expressed in and a buffer containing 10 mM MgCl_2_, 20 mM HEPES-pH 7.5, 50 mM NaCl, 0.02% Brij 35, 0.02 mg/mL BSA and 2 mM freshly added DTT, and 1% DMSO. The resulting mixtures were incubated for 30 min, after which a 5 nM concentration of FITC-labeled geldanamycin was added to initiate the reaction, followed by incubation at room temperature for an additional 3 h. After incubation, fluorescence polarization measurements were taken, and IC_50_ values were calculated applying a sigmoidal dose–response equation. Titration curves were generated using GraphPad Prism 8.0 software.

#### In Vitro Assays on HDACs

IC_50_ evaluations against recombinant HDACs were carried out as described in our previous studies.^29,54,55^ Briefly, the histone deacetylase recombinant enzymes, the compounds, and the appropriate fluorogenic substrates ((i) the RHKK-Ac-AMC peptide for the assays on HDAC1, HDAC2, HDAC3, HDAC6, and HDAC10; (ii) the trifluoroacetyl lysine for the assays on HDAC4, HDAC5, HDAC7, HDAC9, and HDAC11; and (iii) the RHK–Ac–K-Ac-AMC peptide for the assays on HDAC8) were first incubated in a buffer containing 137 mM NaCl, 1 mM MgCl_2_, 1 mg/mL BSA, 50 mM Tris–HCl-pH 8.0, and 2.7 mM KCl. Fluorescence measurements were taken at 360 nm (excitation) and 460 nm (emission) wavelengths after the completion of the reactions. For fluorescence calibration, 2 μM of a reference compound was added to a solution of 16 mg/mL trypsin in the same buffer; the blank for curve fitting was prepared using a buffer containing 1 × 10^–12^ M DMSO. IC_50_ values of the compounds were determined by performing a 3-fold serial dilution in DMSO, starting at 100 μM. The reference compounds used in these assays were trichostatin A for HDAC1, HDAC2, HDAC3, HDAC6, HDAC8, and HDAC11; TMP269 for HDAC4, HDAC5, HDAC7, and HDAC9; and quisinostat for HDAC10. A sigmoidal dose–response equation was then applied to analyze the data with the GraphPad Prism 8.0 software.

#### Cell Lines

The human PC cell line PC3 (ATCC # CRL-1435) was grown in Ham’s F12 (Biowest, Nuaillé, France) or in RPMI 1640 (Euroclone, ECB9006L) culture media, the human PC cell line LNCaP (ATCC # CRL-1740) in RPMI 1640 medium, and the human PC cell line DU145 (ATCC #HTB-81) in RPMI 1640 medium. All media were supplemented with 10% heat-inactivated Fetal Bovine Serum (FBS, Gibco, Fisher Scientific Italia, Segrate, Italia), 2 mM glutamine, 100U/ml penicillin, and 100 μg/mL streptomycin. All cells were maintained at 37 °C in a humidified 5% CO_2_ atmosphere and checked for mycoplasma contamination using the EZ-PCR Mycoplasma Test Kit (Biological Industries, Connecticut, USA; #20-700-20) or the MycoAlert Mycoplasma Detection Kit (Lonza, #LT07-318).

#### Cell Viability Assays

PC3, LNCaP, and DU145 cells were seeded at a density of 4000, 6000, and 2500 cells/well into a 96-well plate, respectively, along with a calibration curve with known cell numbers. The following day, the cells were treated with the synthesized candidate compounds at different concentrations, with serial dilutions as indicated in figure legends. Cell viability in 2D assays was evaluated after 72 h by the PrestoBlue (LNCaP and PC3) cell viability reagent. PrestoBlue reagent (#A13261, Thermo Fisher Scientific, MA, USA) was added to the medium (1:10 v/v) and incubated for 1 h at 37 °C. Cell viability was calculated by quantifying PrestoBlue reduction through absorbance readings at 570 and 620 nm. The viability of untreated cells was arbitrarily set to 100%, and the concentration at which cellular growth is inhibited by 50% (GI_50_) was determined. Three independent experiments were performed.

Cell viability in 3D cultures was evaluated by MTT assay. 0.5 mg/mL of thiazolyl blue tetrazolium bromide (MTT) (Sigma-Aldrich, St. Louis, MO, USA) in culture medium was added to the wells, and cells were incubated at 37 °C for 2h. The medium containing unconverted MTT was removed, and 100 μL of MTT solvent (4 mM HCl, 0.1% NP40, in isopropyl alcohol) was added to the wells to solubilize MTT crystals. After 15 min of incubation at room temperature, the absorbance values at 570 nm were recorded using a Multiskan FC Microplate Photometer (Thermo Fisher Scientific, MA, USA).

#### Protein Extraction and Immunoblotting

Drug-treated PC3 cells were harvested by trypsinization and washed with phosphate-buffered saline 1X (PBS), and whole-cell protein extracts were prepared by lysis into 1X SDS sample buffer (25 mM Tris–HCl pH 6.8, 1.5 mM EDTA, 20% glycerol, 2% SDS, and 5% b-mercaptoethanol). Protein quantitation was performed with the Pierce Detergent Compatible Bradford Assay (#23246, Thermo Fisher Scientific, MA, USA); an equivalent amount of extracts from cells was resolved by SDS-PAGE, transferred the Nitrocellulose membrane with the Trans-Blot Turbo Transfer System (Bio-Rad), and immunoblotted with the primary antibodies listed below, diluted 1:1000 in 1X TBS with 1 mg/mL BSA: Anti-Tubulin (#E-AB-20073, Elabscience), Antiacetylated α-tubulin (#sc-23950, Santa Cruz), Anti-HSP70 (#4872S, Cell Signaling), Anti-HDAC6 (#7558S, Cell Signaling), Antiacetylated HSP90 (#ABP50105, Abbkine), Anti-Akt (#9272S, Cell Signaling), Anti-Acetylated Histone H3 (#F1121, Santa Cruz), and Anti-Histone H3 (#PA5–16183, Invitrogen). After incubation with secondary HRP-conjugated antibodies, Antimouse (#A16017, Invitrogen) and Antirabbit (# A16023, Invitrogen) membranes were imaged using Supernova HRP substrates (Cyanagen) and Westar ηC detection reagents, with an Amersham Imager AI680 RGB (GE Healthcare). After imaging the initial immunoblotting procedure, some membranes required stripping and reprobing. To achieve this, membranes were first washed for 5 min in TBS, followed by two 5 min washes in dH_2_O. They were then stripped using 0.2 M NaOH for 20 min. Subsequently, the membranes underwent two additional 5 min washes in dH_2_O before being reblocked and probed with fresh antibodies, following the previously described protocol.

#### Flow Cytometric Analysis of Cell Cycle

Quantitative measurements of the cell cycle phase distribution were performed by flow cytometry. Cells were suspended in 0.5 mL of Propidium Iodide solution (50 μg/mL propidium iodide, 3.4 mM sodium citrate, and 0.1% Triton X-100 in PBS) and analyzed by the Attune NxT Flow Cytometer equipped with a Blue Excitation Laser (488 nm), Red Excitation Laser (638 nm), Violet Excitation Laser (405 nm), and Yellow Laser (561 nm).

#### Drug Combination Assays

PC3 cells were seeded at a density of 4000 cells/well into a 96-wells tissue-culture plate and incubated for 72 h with compound **17**, tubastatin A, geldanamycin, and doxorubicin as single agents or in different combinations. Treatments were performed as dose–response experiments, starting from 50 μM with 3-fold serial dilutions. Taking into account the different GI_50_ values, drugs were combined at a constant molar ratio of 1:1, except for geldanamycin that was used at 1:50 ratio. The percentage of viable cells was calculated using the Presto-Blue cell viability assay, and the Compusyn software (version 1.0) was used to calculate the CI to determine synergism, additivity, or antagonism at 50% cell viability (Fa = 0.5).

#### 3D Tumor Spheroids Generation and Treatment

For the generation of spheroids from multicellular cell aggregates (Multicellular Tumor Spheroid, MTS), 5000 PC3 cells were seeded into 96-well Round Bottom ultra-low attachment plates (Corning #7007 and #4515) using 150 μL of ice-cold complete medium supplemented with 0.25 mg/mL of Matrigel Growth Factor Reduced Basement Membrane Matrix (Corning #354230). The plates were centrifuged at 1000*g* for 10 min at 4 °C. Subsequently, the plates were incubated at 37 °C with 5% CO_2_ in a humidified environment. Afterward, the medium was replaced with 200 μL of a 10 μM solution of the test compound **17** or DMSO (CTR) in cell medium. The MTSs were maintained for 7 days at 37 °C, 5% CO_2_ in a humidified incubator. Images of the MTSs were captured daily from day 0 (immediately post-treatment) to day 7 using the EVOS M5000 imaging system (Thermo Fisher Scientific). The acquired images were analyzed with ImageJ software to calculate the spheroid area, and the Relative Spheroid Area (μm^2^), normalized to day 0, was determined for each time point using the following formula: Relative Spheroid Area (μm^2^) = (Spheroid Area (μm^2^) at day n/Spheroid Area (μm^2^) at day 0) × 100. To assess cell viability, an MTT assay was performed on the MTSs as described above.

For the generation of spheroids from single cells (sc-TSs), PC3 cells were grown in complete RPMI 1640 medium (Euroclone, ECB9006L), with addition of 1% essential amino acids solution (MEM; Euroclone, ECB3054D). Matrigel (Corning, #354254) was briefly mixed at a concentration of 3.5% with RPMI complete medium. This mixture was then added to a prechilled low attachment 6-well plate (Corning, #3471) in 100 μL domes per well. Subsequently, a mix of 3 × 10^4^ cells with Matrigel (30 μL of cells +270 μL of Matrigel) was added to each dome. After 72 h, spheroids formation was observed using inverted light microscopy, and the spheroids generated were seeded in complete RPMI at 1 × 10^4^ cells/well in a 96-well flat bottom ultralow attachment plate (Corning, #3474), generating single cell-derived spheroids (sc-TS). sc-TSs were treated for 6 days as follows: compound **17**, compound **1**, and tubastatin A were used at a concentration of 10 μM. Geldanamycin was tested at final concentrations of 10 μM and 1 μM. Additionally, combination treatments of geldanamycin and tubastatin A were administered at concentrations of 1μM + 10 μM and 10μM + 10 μM, respectively. sc-TSs viability was assessed each day by using thiazolyl blue tetrazolium bromide (3-(4,5-dimethylthiazol-2-yl)-2,5-diphenyltetrazolium bromide) (MTT; Sigma-Aldrich, Schnelldorf, Germany, #57360-69-7) according to the manufacturer’s instructions. At the end of each treatment, absorbance values were read at a wavelength of 570 nm using a TECAN M200 reader (Tecan, Männedorf, Switzerland). Images were acquired over a 6 day treatment using a light microscope (Leica DMIL #520802) at 10× magnification.

### Drug-like Properties

#### Kinetic Solubility

Stock solutions of the test compounds were prepared at a concentration of 50 mM and 5 mM in 100% DMSO. To obtain 500 μM and 50 μM samples, the stock solution was directly diluted with 50 mM phosphate buffer (pH 7.4) and 50 mM citric buffer (pH 4.5), ensuring a final DMSO concentration of 1%. Reference solutions were prepared in 100% methanol. For the solubility assay, 200 μL of each buffer solution was incubated at 37 °C with shaking (300 rpm) for 2 h in a 96-well plate and then centrifuged for 15 min at 4600 rpm. Samples were transferred onto a Multiscreen plate (Millipore), and vacuum was applied. After filtration, samples were diluted 1:100 in ACN, and verapamil (0.1 μM) was added as the IS. The samples were analyzed by LC–MS/MS using the Acquity UPLC system (Waters), coupled with an API 3200 Triple Quadrupole (AB Sciex). Samples (5 μL) were injected onto a Kinetex C8 column (50 × 2.1 mm, 2.6 μm, 100A), maintained at 35 °C, with a flow rate of 0.4 mL/min using a mobile phase composed of A: 0.1% Formic acid (FA) in water and B: 0.1% FA in ACN. The concentrations of the compounds were calculated by comparing the area under the curve (AUC) of the reference solutions (500 μM dissolved in the organic solvent) with the AUC of the test compounds dissolved in the aqueous buffer, after filtration.

#### Permeability in Caco-2 Cells

Caco-2 cells (ECACC) were obtained from Readycells (Barcelona, Spain) after 21 days of culture. Three days prior to the experiment, the CacoReady plates were placed at 37 °C in a cell culture incubator, with a 95% air/5% CO_2_ atmosphere, for 4 h. The shipping medium was removed from the plate by aspiration and replaced with fresh culture medium consisting of DMEM (1g/L glucose, 10% FCS, 1% glutamine 200 mM, and 1% penicillin (10,000 U/mL)—streptomycin (10/mg/mL) in HBSS). The cells were maintained at 37 °C with 5% CO_2_ until the day of the experiment. Before starting the experiments, transendothelial electrical resistance was confirmed to be > 1000 Ω cm^2^, using a Millicell-ERS Millipore.

Metoprolol was used as a reference compound for high permeability. The test compounds were assessed at 2 μM and standards at 5 μM concentration. Test compound stock solutions (1 mM in DMSO) were diluted in HBSS buffer (1:500) to reach 2 μM concentration (0.1% DMSO). The transport across the Caco-2 monolayer was determined by adding 0.25 mL of the above solutions to the apical side with 0.75 mL of buffer in the basolateral side. The cells were incubated for 2 h at 37 °C and 5% CO_2_. After 2 h, 50 μL of the sample from the two compartments was collected, diluted with 150 μL of IS solution (Verapamil 0.1 μM in ACN), and kept at −20 °C until analysis. At the end of the experiment, the integrity of the cellular monolayer was assessed by measuring the permeability of Lucifer yellow, as a low permeability compound. The plate was analyzed using a fluorimeter at λ: 430–538 nm. For the analysis, the solution from the apical side (100 μL + 100 μL of buffer) and from the basolateral side (200 μL) was transferred to a 96-black plate. The samples were analyzed using the UPLC Acquity (Waters) coupled with API 3200 Triple Quadrupole (AB Sciex). Samples (5 μL) were injected into a Kinetex C8 column (50 × 2.1 mm, 2.6 μm, 100A), maintained at 35 °C, with a flow rate of 0.3 mL/min and a mobile phase composed of A: 0.1% Formic acid (FA) in water and B: 0.1% FA in Acetonitrile.

The apparent permeability (*P*_app_) was calculated according to the following equation:

$$P_{app}=J/C_{o}$$

where *J* = flux (d*X*/d*t* × *A*) and *C*_o_ = donor concentration (uM) at *t* = 0; d*X*/d*t* = change in mass (*X*, n mol) per time (*t*, sec); and *A* = filter surface area (cm^2^). Data were processed with Microsoft Excel (https://office.microsoft.com/excel) and are expressed as mean of *n* = 2 (±St.Dev.).

#### Metabolic Stability in Liver Microsomes

The test compound and reference standard Verapamil were solubilized in DMSO to obtain a 200 μM solution. Samples were prepared in duplicate at 1 μM concentration in potassium phosphate buffer 50 mM pH 7.4, 3 mM MgCl_2_, and preincubated with mouse and human liver microsomes (Sigma) at the final concentration of 0.5 mg/mL at 37 °C for 10 min. Then, the mixture of cofactors (NADP, Glc6P, and Glc6P-DH in 2% Sodium bicarbonate) was added; a control sample without cofactors was prepared to check the stability of test compounds in the matrix after 60 min. At time points 0, 10, 30, 45, and 60 min, 20 μL samples were collected and mixed with 150 μL of acetonitrile containing 250 ng/mL of 7-Ethoxy coumarin as IS to stop the reaction. The samples were centrifuged, and the supernatant was collected and analyzed by LC–MS/MS. Acquity UPLC (Waters) coupled with an API 3200 Triple Quadrupole ABSciex was employed. The samples (5 μL) were injected onto a Kinetex C8 column (50 × 2.1 mm, 2.6 μm, 100A) maintained at 35 °C with a flow rate of 0.4 mL/min. The mobile phase consisted of A: 0.1% Formic acid (FA) in water and B: 0.1% FA in ACN.

The percentage of the test compound’s area remaining at each incubation time point was calculated by comparing its area to the compound’s area at 0 min time point.

The rate constant, *k* (min^–1^) obtained from for the exponential decay equation (peak area/IS vs time) was used to calculate the intrinsic clearance rate (*Cl*_*i*_) of the compound using the following equation:

$${Cl}_{i}\left(\;\mu L/\min/mg\right)=k/\left[microsomal\;conc.\right]\times 1000$$

where *k* is the rate constant (min^–1^) and the microsomal protein concentration is 0.5 mg protein/mL.

## Supplementary Material





## Notes

Dose–response curves of the synthesized compounds for the inhibition of recombinant HDAC6; dose–response curves of the synthesized compounds for the inhibition of recombinant Hsp90; dose–response curves of compounds **7** and **17** for the inhibition of all HDAC isoforms (selectivity profiling); dose–response curves of the antiproliferative effects of the synthesized compounds on LNCaP cells; dose–response curves of the antiproliferative effects of the synthesized compounds on PC3 cells; dose–response curves of the antiproliferative effects of the synthesized compounds on DU145 cells; Western blot analyses for testing nonspecific targeting of nuclear HDACs and efficacy of the dual targeting compound **17**; combination studies of tubastatin A, geldanamycin, compound **17**, and doxorubicin in PC3 cells; and NMR spectra and HPLC chromatograms (PDF)
Molecular formula of the investigated compounds in SMILES format (CSV)

## #

University of Cagliari, Complesso Universitario di Monserrato, Monserrato (Cagliari), Italy

## Abbreviations

Ac-TUB: acetylated-tubulin
ADT: androgen deprivation therapy
AR: androgen receptor
CI: combination index
CRPC: castration-resistant prostate cancer
Fa: fraction affected
GELDA: geldanamycin
HDAC6: histone deacetylase 6
HDACs: histone deacetylases
HPLC: high-performance liquid chromatography
HRMS: high-resolution mass spectra
Hsp90: heat shock protein 90
PI: propidium iodide
MTSs: multicellular tumor spheroids
PC: prostate cancer
rt: room temperature
TLC: thin layer chromatography
TUB: tubulin
TUB-A: tubastatin A
WB: Western blot
ZBG: zinc binding group.
