# Supplementary material for: Structure-Based Discovery of Hsp90/HDAC6 Dual Inhibitors Targeting Aggressive Prostate Cancer
Source: J Med Chem. 2025 Jul 23;68(15):15738–65. doi: 10.1021/acs.jmedchem.5c00717 (PMC12362625; doi:10.1021/acs.jmedchem.5c00717)

SUPPORTING INFORMATION

Structure-Based Discovery of Hsp90/HDAC6 Dual Inhibitors Targeting Aggressive Prostate Cancer

Andrea Citarella,[1,2] Silvia Belluti,[1] Davide Bonanni,[1] Davide Moi,[1,2] Isabella Piccinini,[1] Arianna Rinaldi, [1] Chiara Papulino,[3] Rosaria Benedetti,[3,4] Laura Cuoghi,[1] Stefano Di Ciolo,[2] Alessandra Silvani,[2] Lucia Altucci,[3,4,5] Luca Pinzi,[1] Silvia Franchini,[1] Daniele Passarella,[2] Claudia Sorbi,[1] Clelia Giannini,[2] Carol Imbriano,[1] Giulio Rastelli[1*]

[1] Department of Life Sciences, University of Modena and Reggio Emilia, Via Campi 103, 41125 Modena, Italy
[2] Department of Chemistry, University of Milan, Via Golgi 19, 20133 Milano, Italy
[3] Department of Precision Medicine, University of Campania "Luigi Vanvitelli", 80138 Naples, Italy.
[4] Program of Medical Epigenetics, Vanvitelli Hospital, 80138, Naples, Italy.
[5] Biogem Institute of Molecular and Genetic Biology, 83031, Ariano Irpino, Italy.

Corresponding Author
Prof. Giulio Rastelli
Department of Life Sciences
University of Modena and Reggio Emilia
Via Campi 103, 41125 Modena, Italy
Email: giulio.rastelli@unimore.it

TABLE OF CONTENTS

Figures

Figure S1: Dose-response curves of the synthesized compounds for the inhibition of recombinant HDAC6.

Figure S2: Dose-response curves of the synthesized compounds for the inhibition of recombinant Hsp90.

Figure S3: Dose-response curves of compounds **7** and **17** for the inhibition of all HDAC isoforms (selectivity profiling).

Figure S4: Dose-response curves of the anti-proliferative effects of the synthesized compounds on LNCaP cells.

Figure S5: Dose-response curves of the anti-proliferative effects of the synthesized compounds on PC3 cells.

Figure S6: Dose-response curves of the anti-proliferative effects of the synthesized compounds on DU145 cells.

Figure S7: Western blot analyses for testing nonspecific targeting of nuclear HDACs, and efficacy of the dual-targeting compound **17**.

Figure S8: Combination studies of tubastatin A, geldanamycin, compound **17**, and doxorubicin in PC3 cells.

NMR spectra, LCMS and HPLC chromatograms

Figure S1: Dose-response curves of the synthesized compounds for the inhibition of recombinant HDAC6.

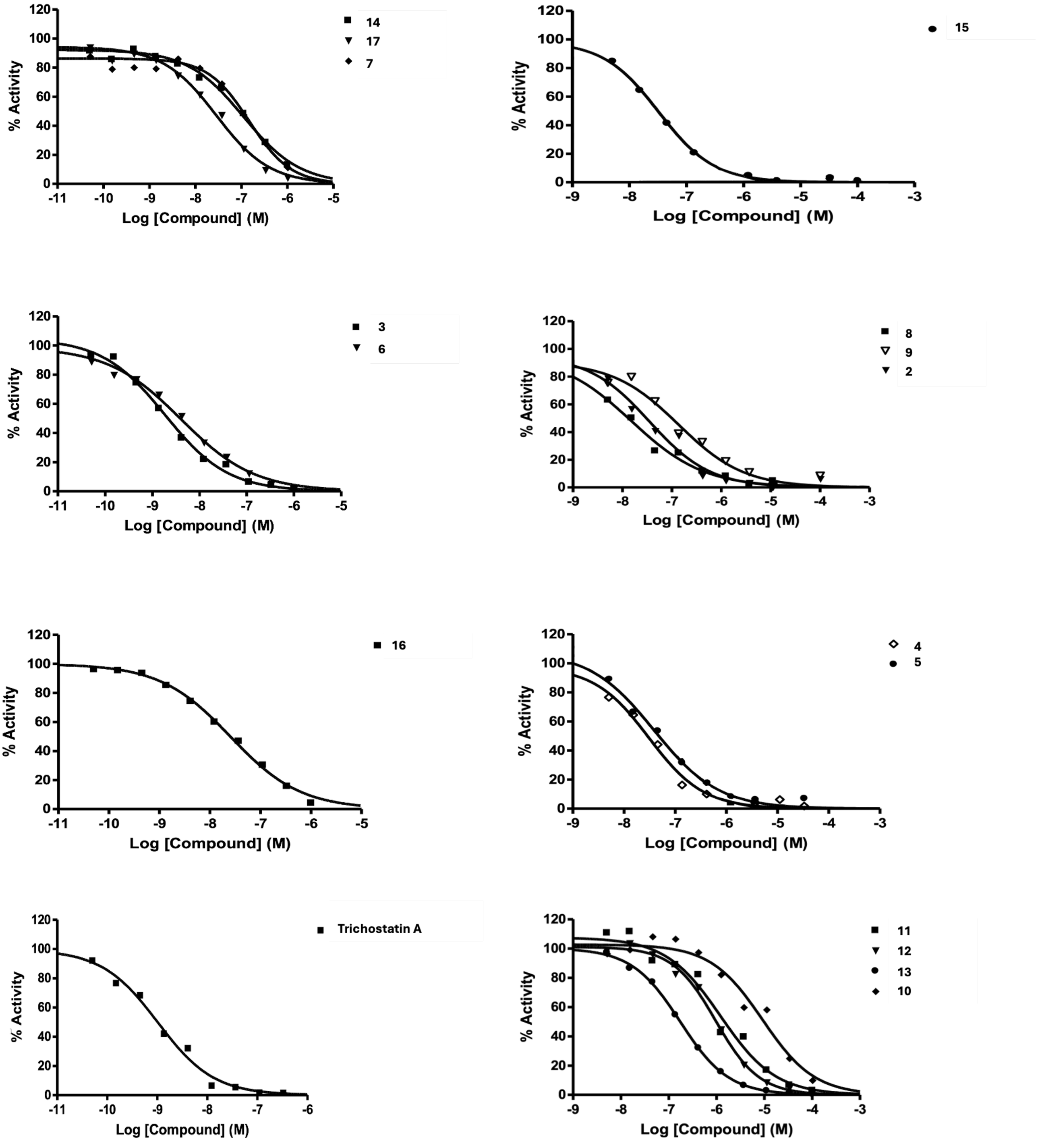

Figure S2: Dose-response curves of the synthesized compounds for the inhibition of recombinant Hsp90.

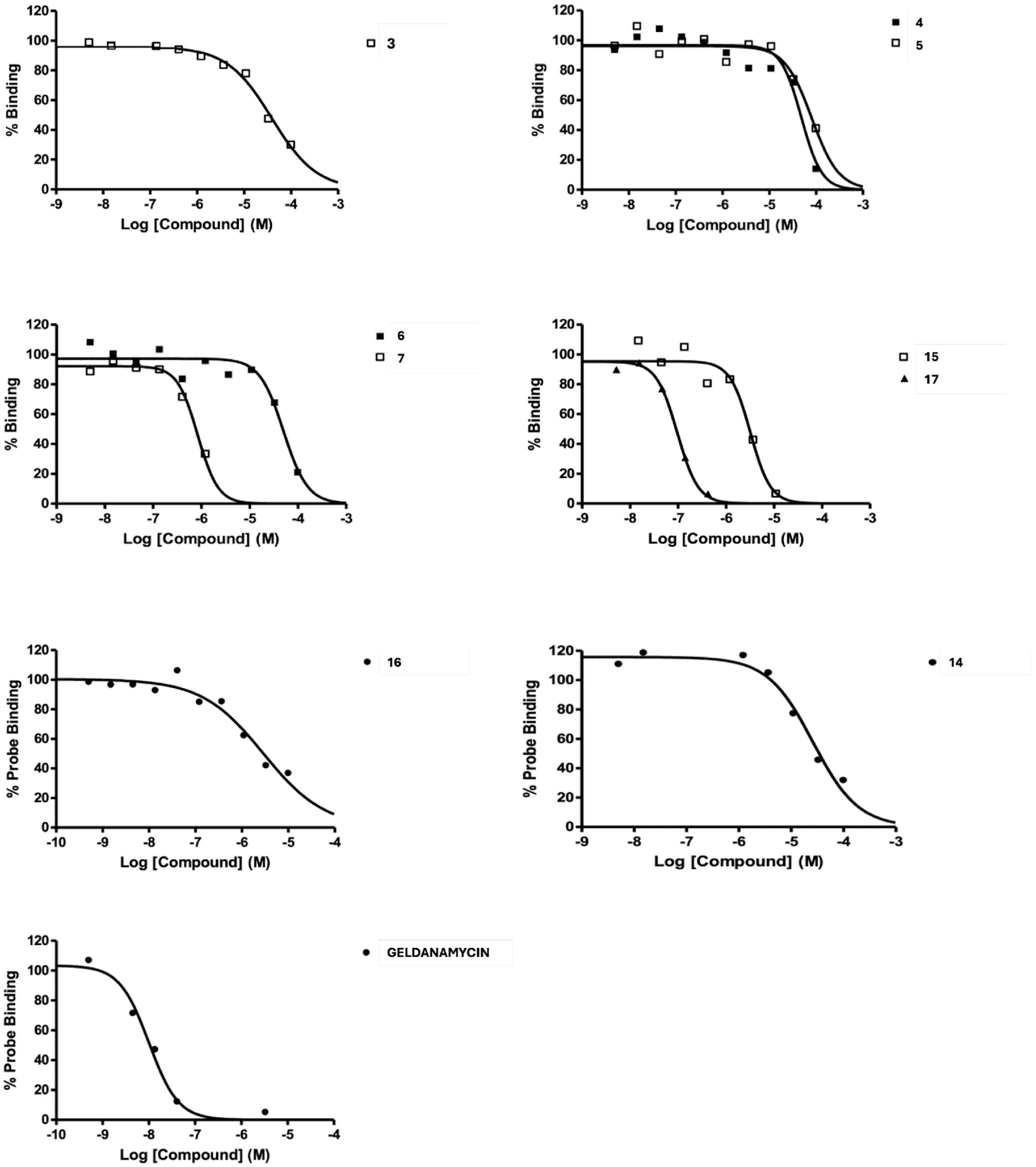

Figure S3: Dose-response curves of compounds **7** and **17** for the inhibition of all HDAC enzymes (selectivity profiling).

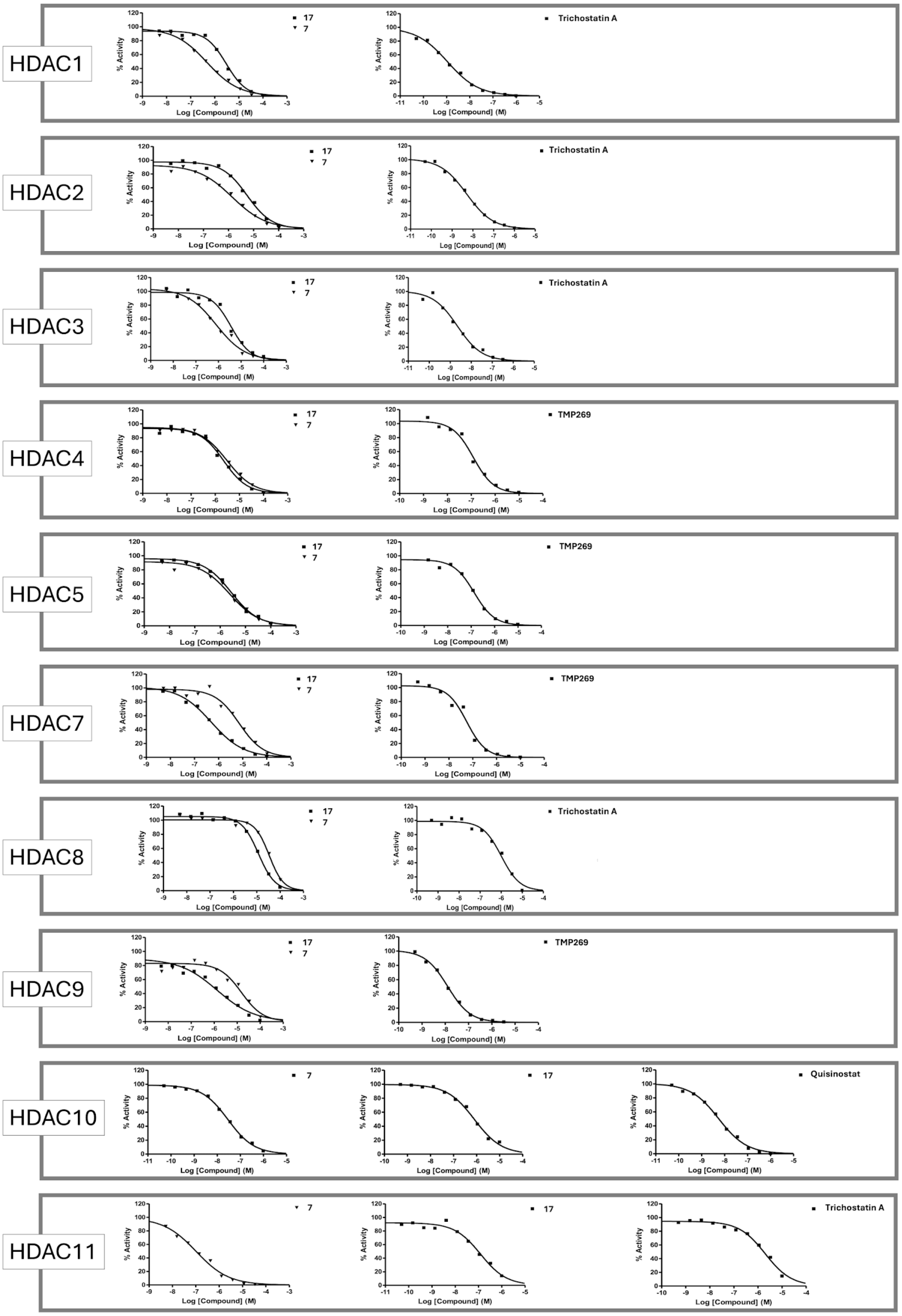

Figure S4. Dose-response curves of the anti-proliferative effects determined by Presto-Blue assay on LNCaP cells treated for 72h with different doses of the synthesized compounds and reference standards *(n=3)*, starting from 100 μM with 2-fold serial dilutions.

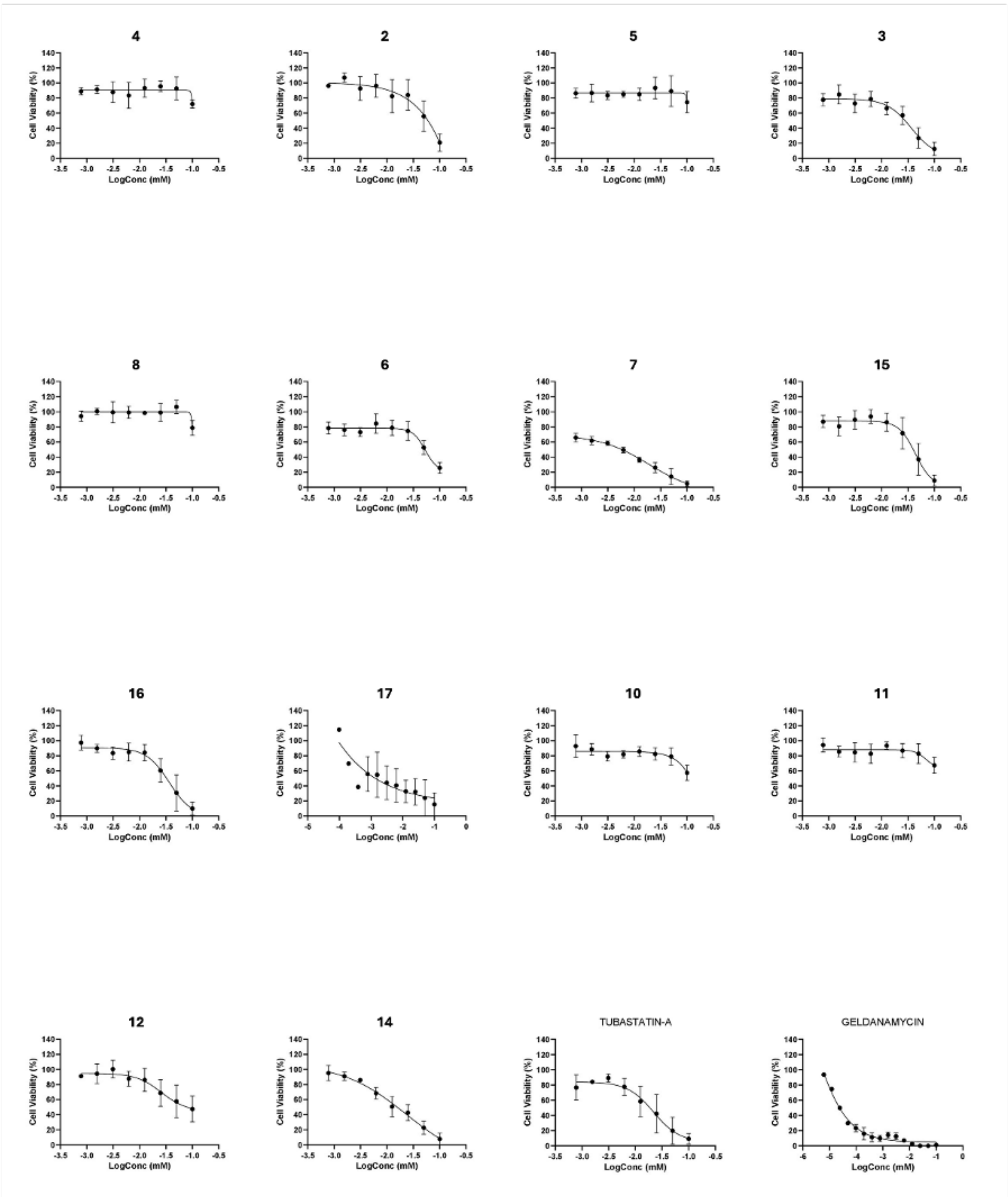

Figure S5. Dose-response curves of the anti-proliferative effects determined by Presto-Blue assay on PC3 cells treated for 72h with different doses of the synthesized compounds and reference standards *(n=3)*, starting from 300 μM with 3-fold serial dilutions.

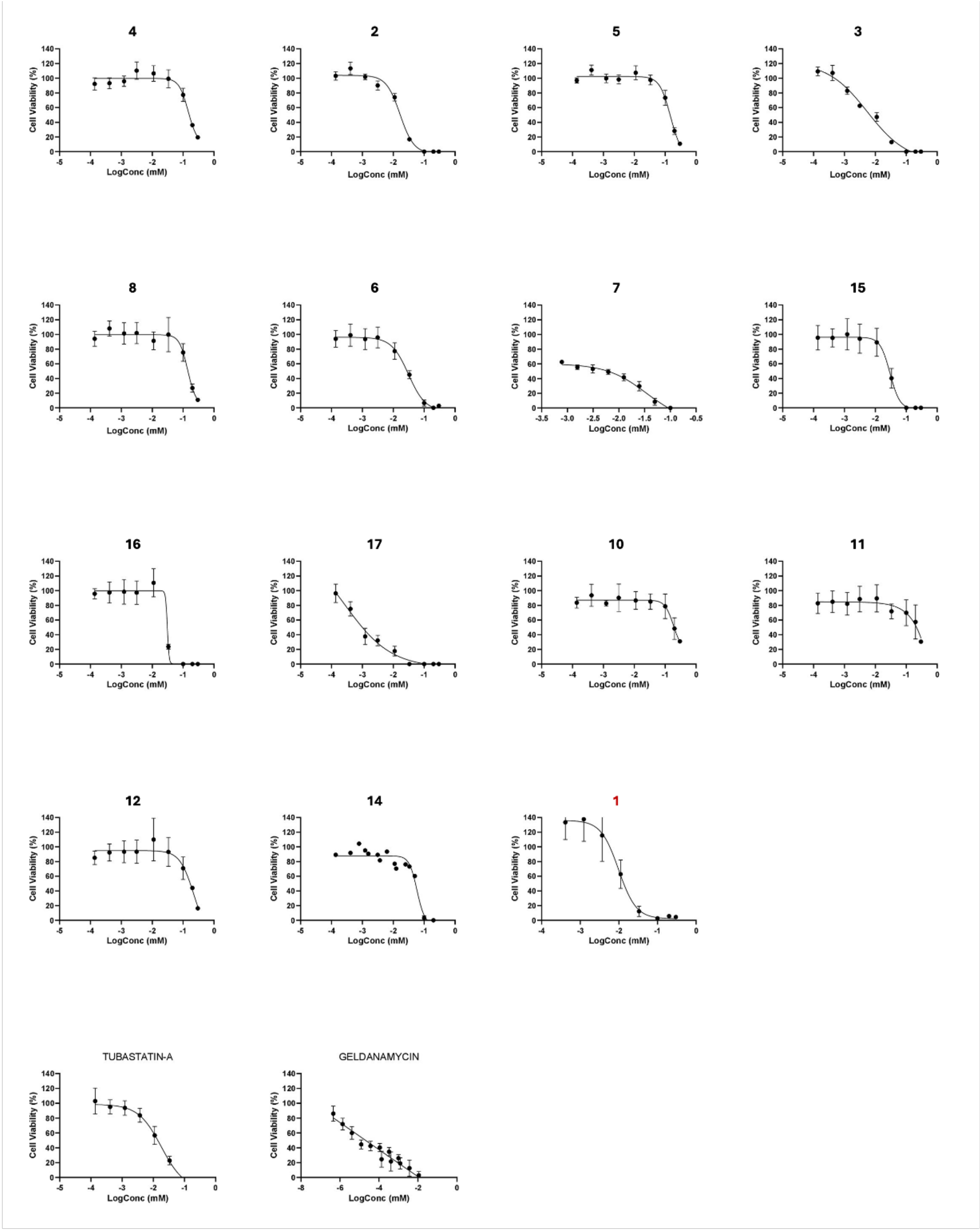

Figure S6. Dose-response curves of the anti-proliferative effects determined by MTT assay on DU145 cells treated for 72h with different doses of the synthesized compounds and reference standards *(n=3)*, starting from 200 µM with 3-fold serial dilutions.

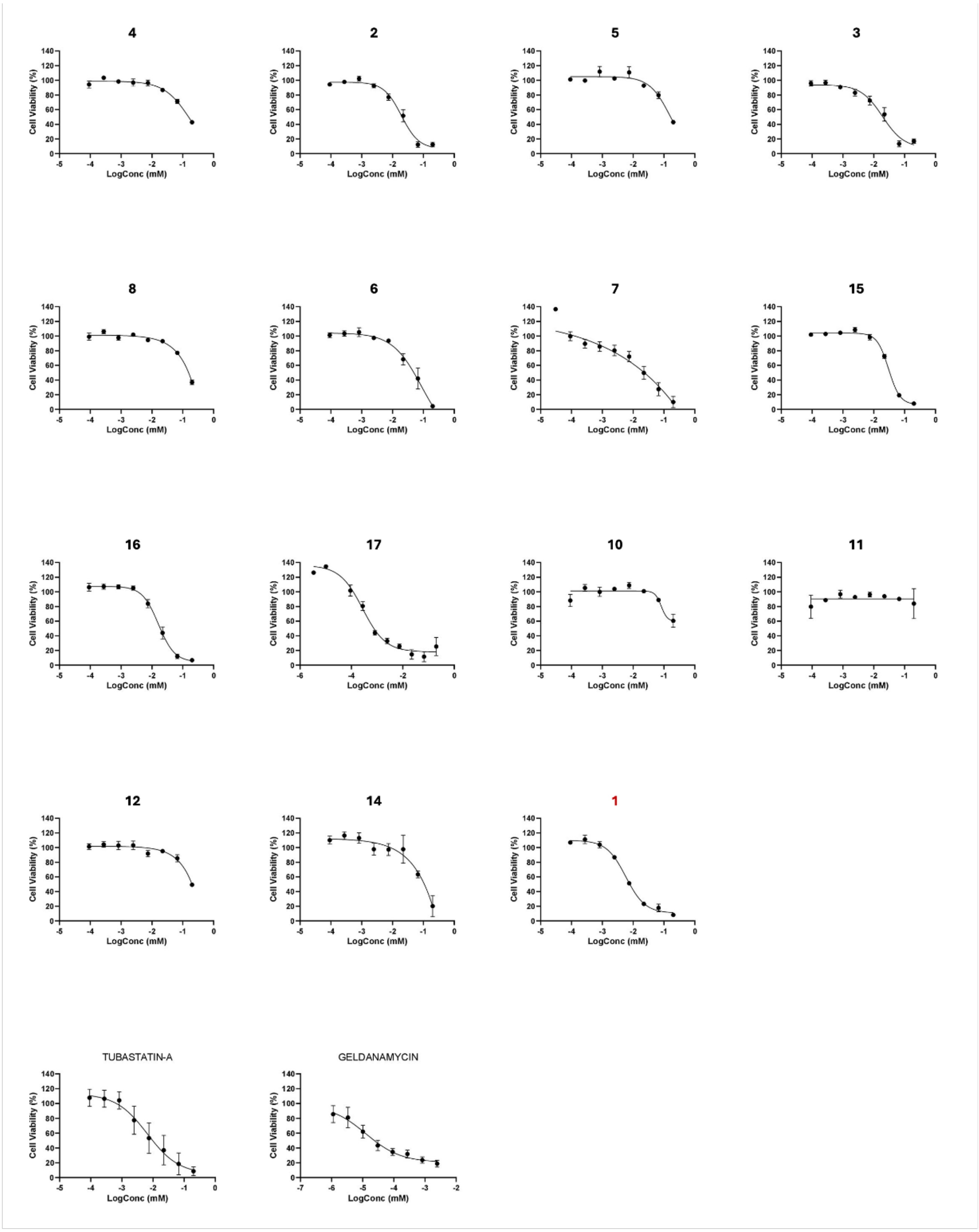

Figure S7. Expression levels of acetylated-H3 and total H3 in PC3 cells treated for 24 h at 1µM dose (panel A) and 10µM dose (panel B), as evaluated by Western blot analysis. Tubastatin A and geldamamycin were used as HDAC6- and Hsp90-specific positive controls, respectively. SAHA was used as positive control for the inhibition of nuclear HDACs. H3 was used as loading control. (Panel C): The viability of PC3 cells was evaluated after 72 h of treatment with tubastatin A and geldanamycin at their GI_{50} concentrations. Cell viability was also evaluated after co-administration of the two compounds at their respective GI_{50}, 1/4 of GI_{50}, and 1/10 of GI_{50} concentrations. Cell viability vas evaluated by PrestoBlue™ reagent. (Panel D): Expression levels of acetylated-tubulin, Hsp70, and Tubulin in PC3 cells treated for 24 hours at the GI_{50} dose of compound 17, or after co-treatment with tubastatin A and geldamamycin at doses corresponding to 1/4 and 1/10 of their respective GI_{50} concentrations. Tubulin was used as loading control.

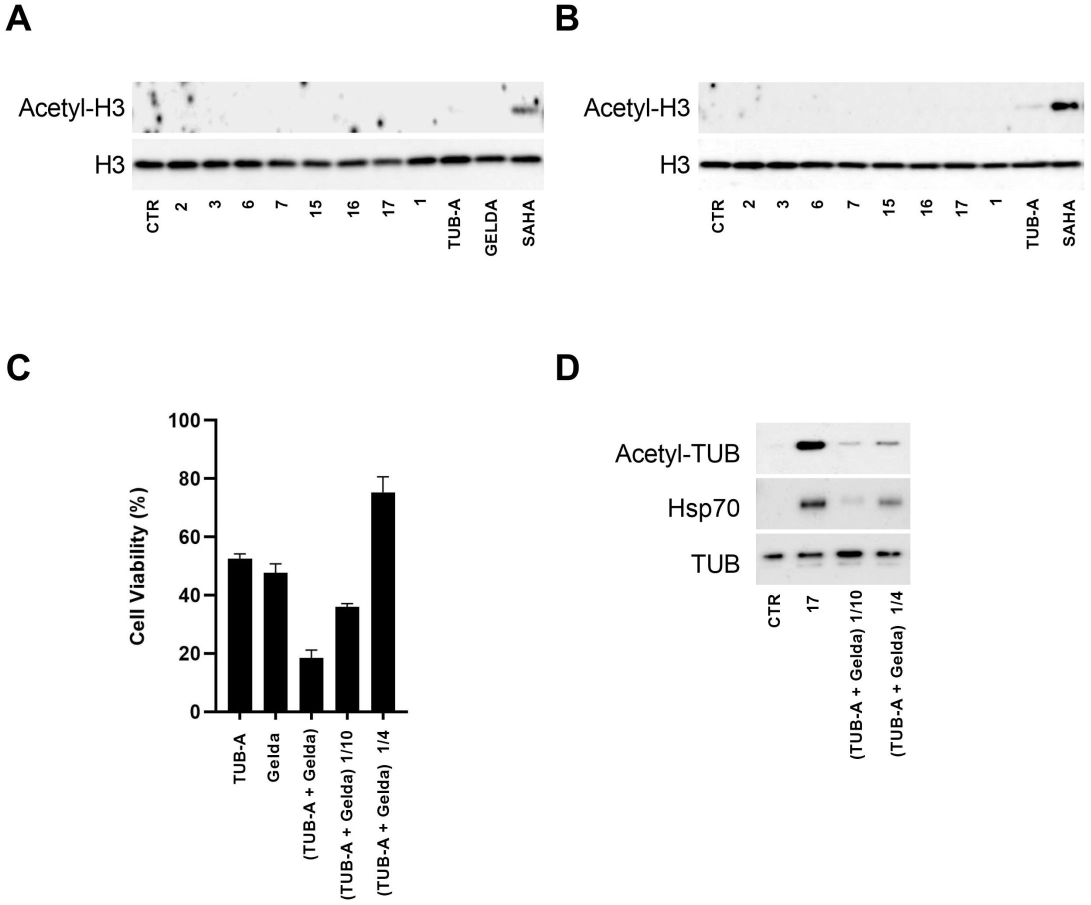

Figure S8. Combination studies of tubastatin A, geldanamycin, compound **17**, and doxorubicin in PC3 cells. Cells were incubated for 72 hours with the indicated combinations of compounds and Doxorubicin, starting from 50 µM with 3-fold serial dilutions, at a constant ratio of 1:1, except for geldanamycin, with constant ratio of 1:50 (geldanamycin:compound). The percentage of viable cells was calculated by Presto Blue cell viability assay. The Chou-Talalay method was used. Graphs represent the Fa-log(CI) plot obtained with CompuSyn software. Fa indicates the fractional inhibition for each combinational index (i.e. Fa = 0.5 corresponds to 50% of inhibition of cell proliferation). The baseline at log(CI)=0 indicates an additive effect, while log(CI) values below, equal to, or above 0 indicate synergy, additivity, or antagonism, respectively.

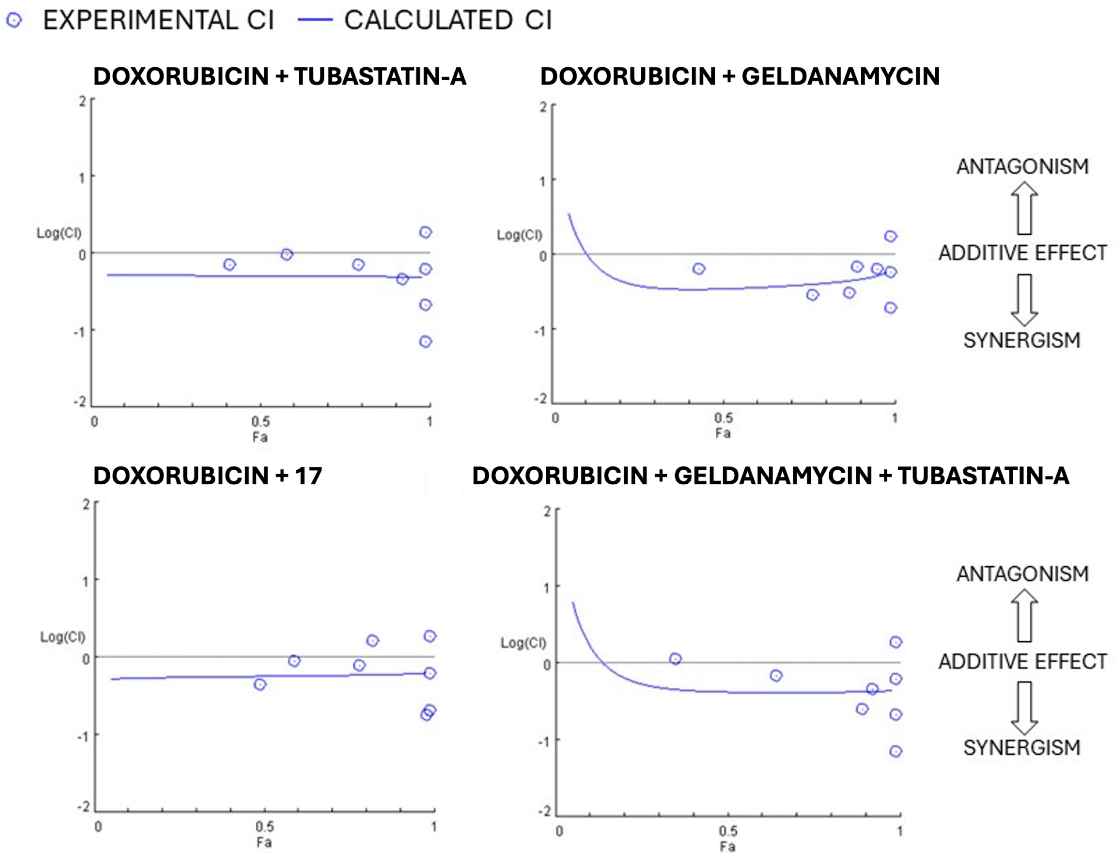

NMR spectra, LCMS and HPLC chromatograms

1H NMR and 13C NMR spectra of compound 2.

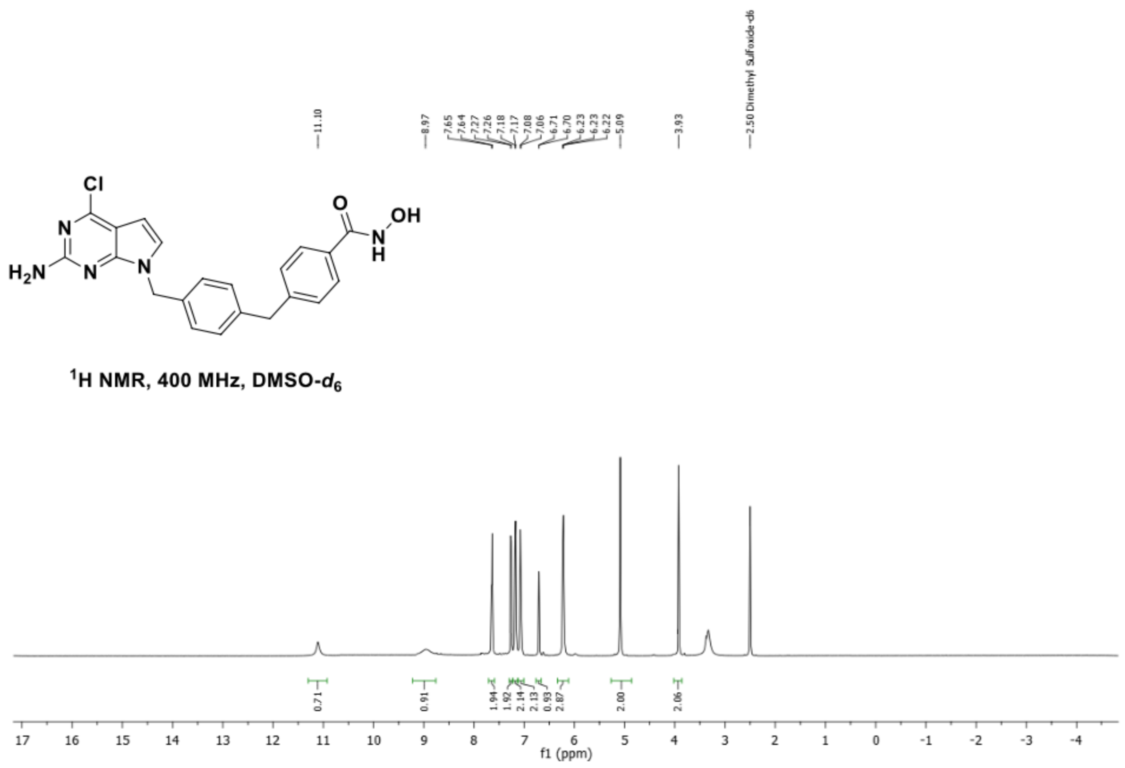

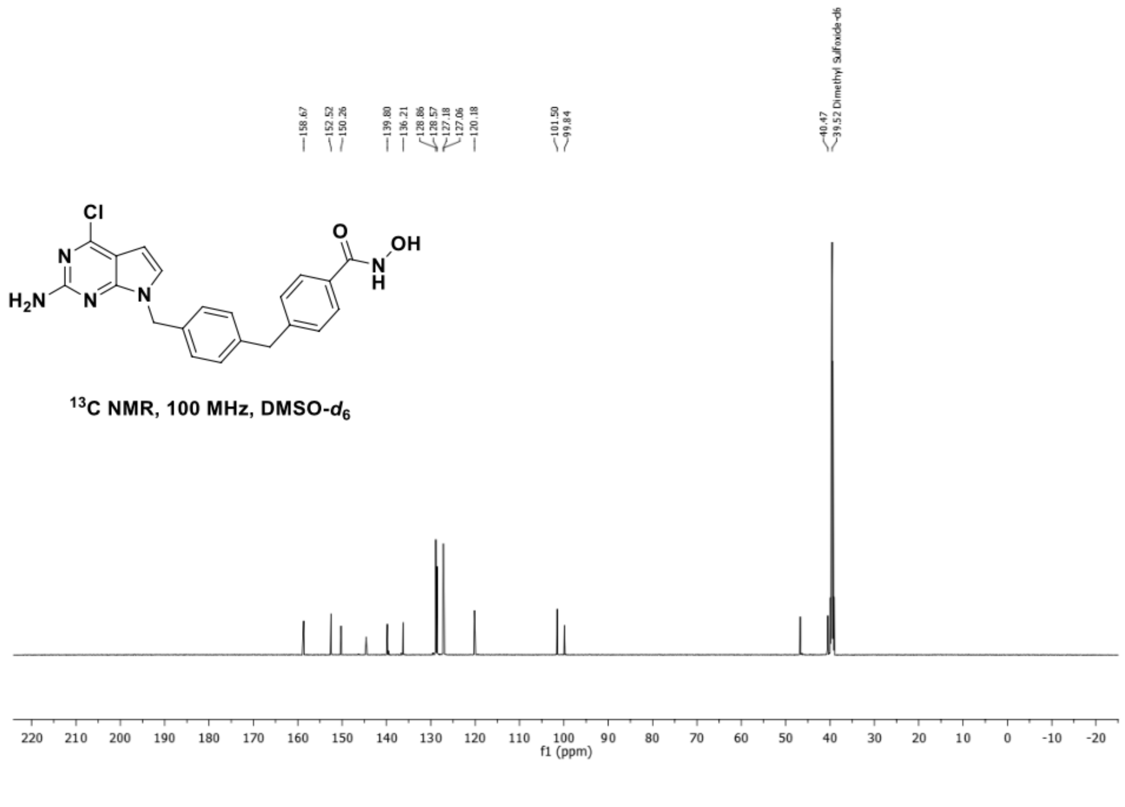

1H NMR and 13C NMR spectra of compound 3.

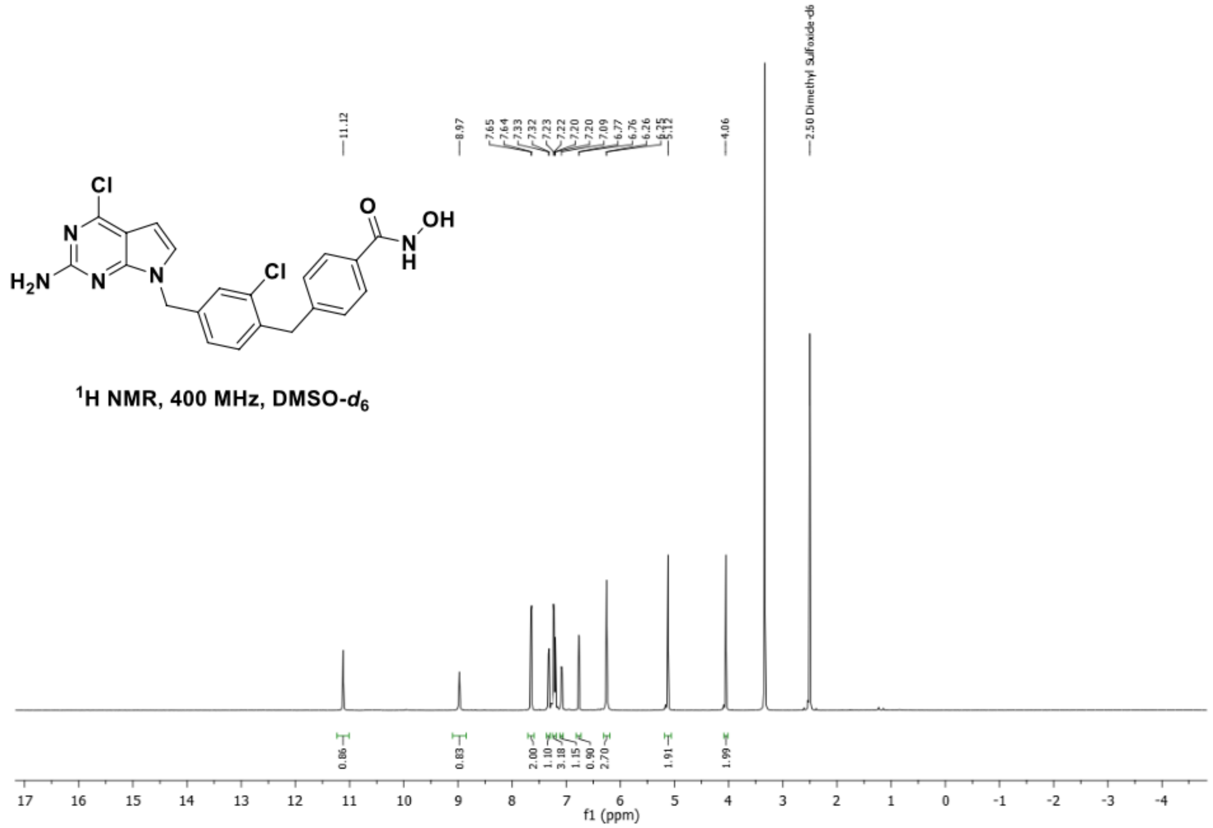

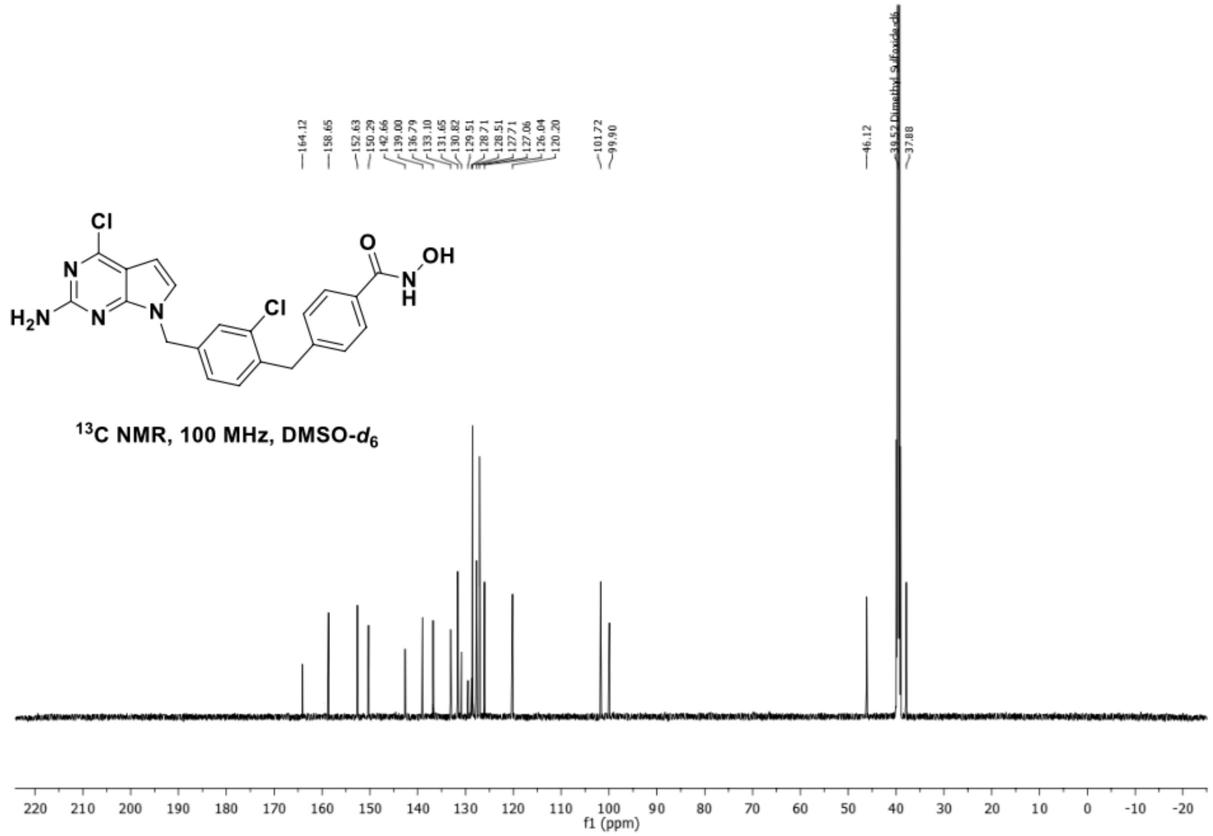

1H NMR and 13C NMR spectra of compound 4.

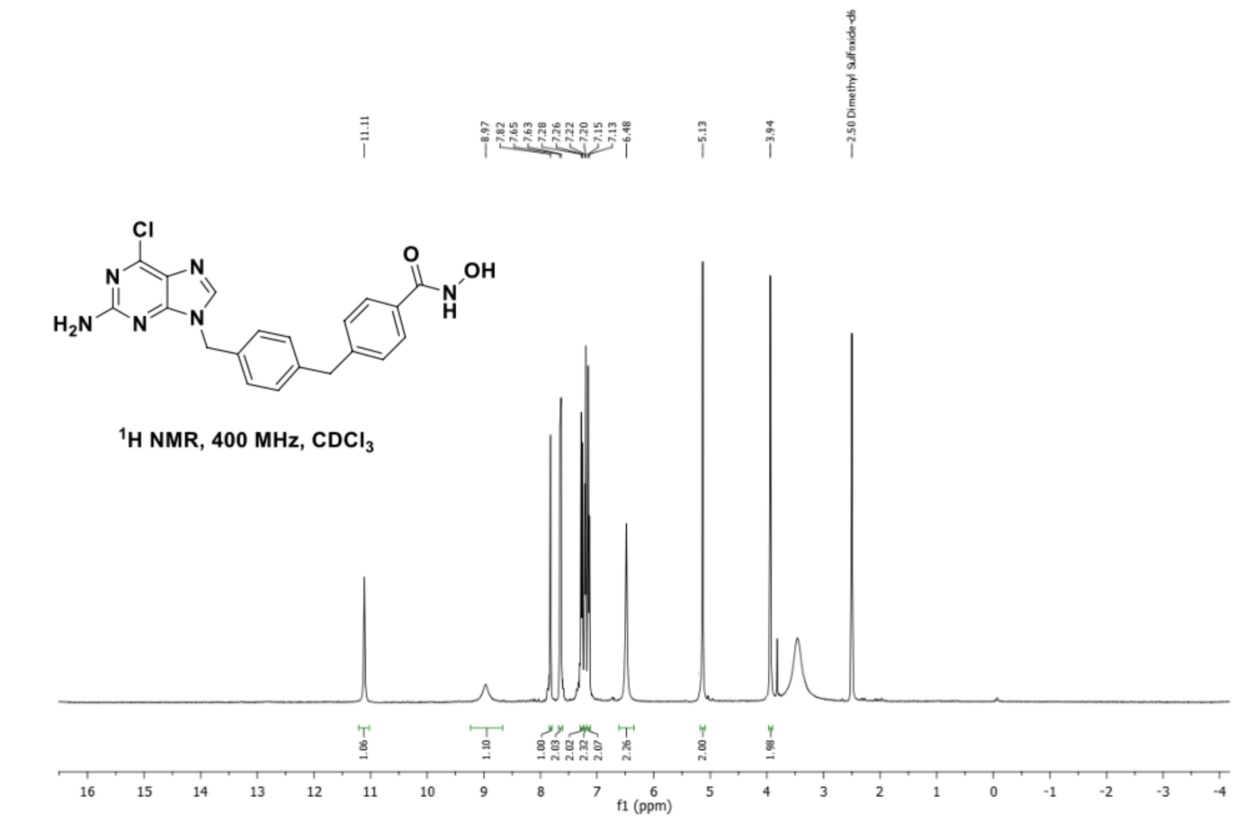

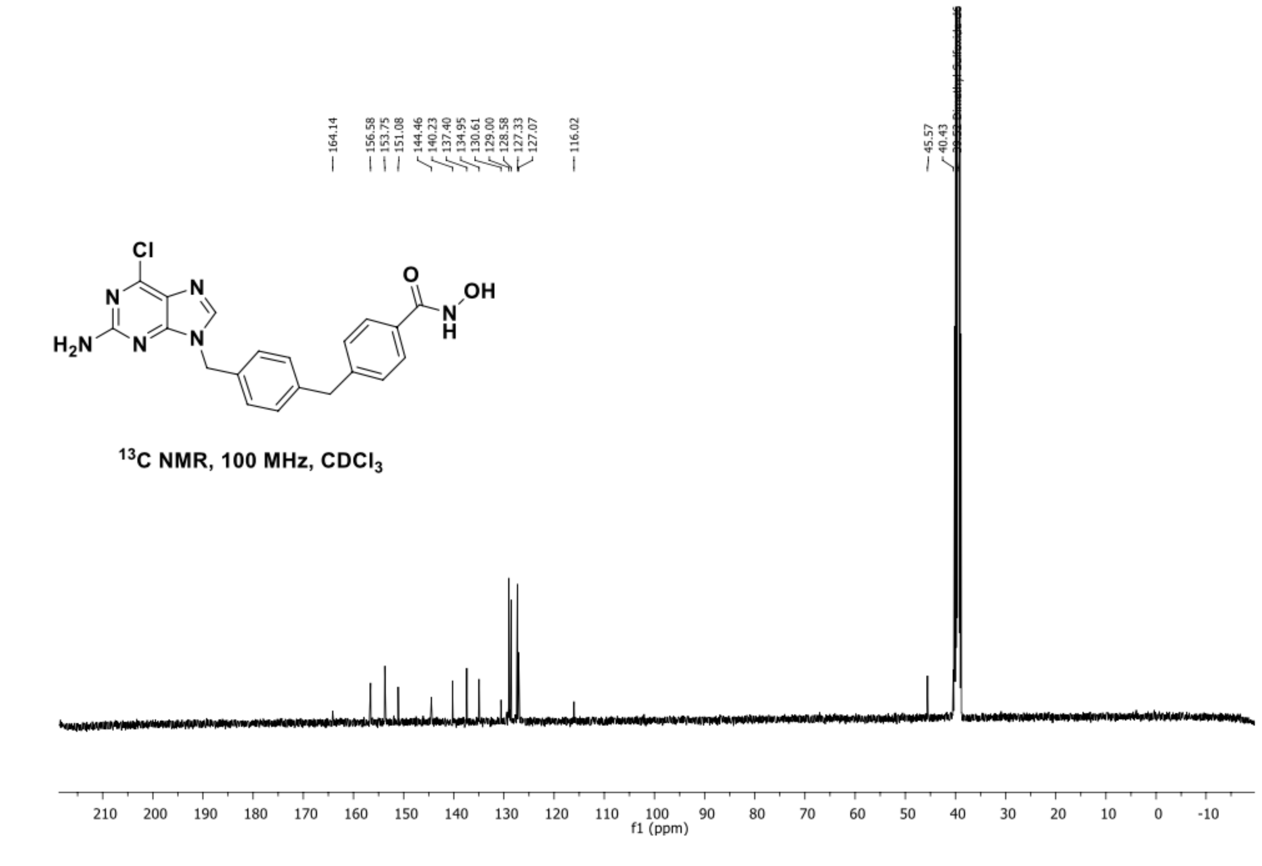

1H NMR and 13C NMR spectra of compound 5.

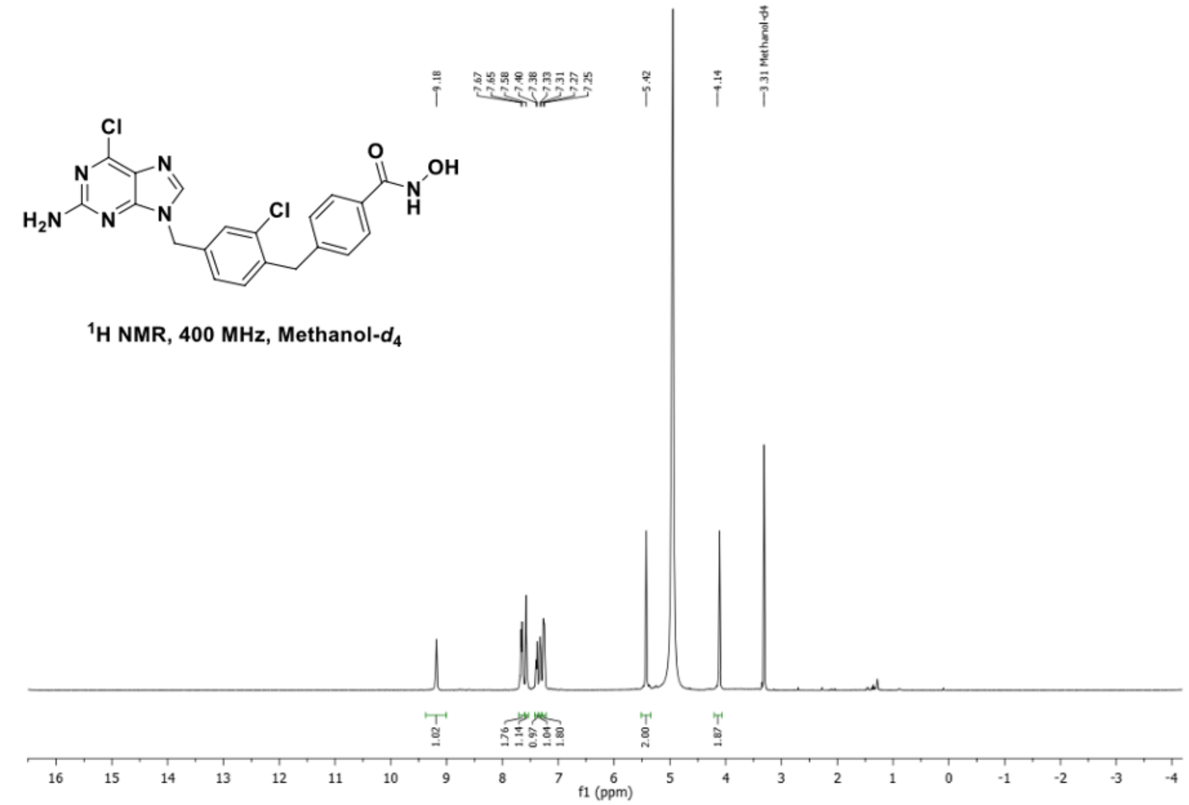

^{1}H NMR, 400 MHz, Methanol-d_4

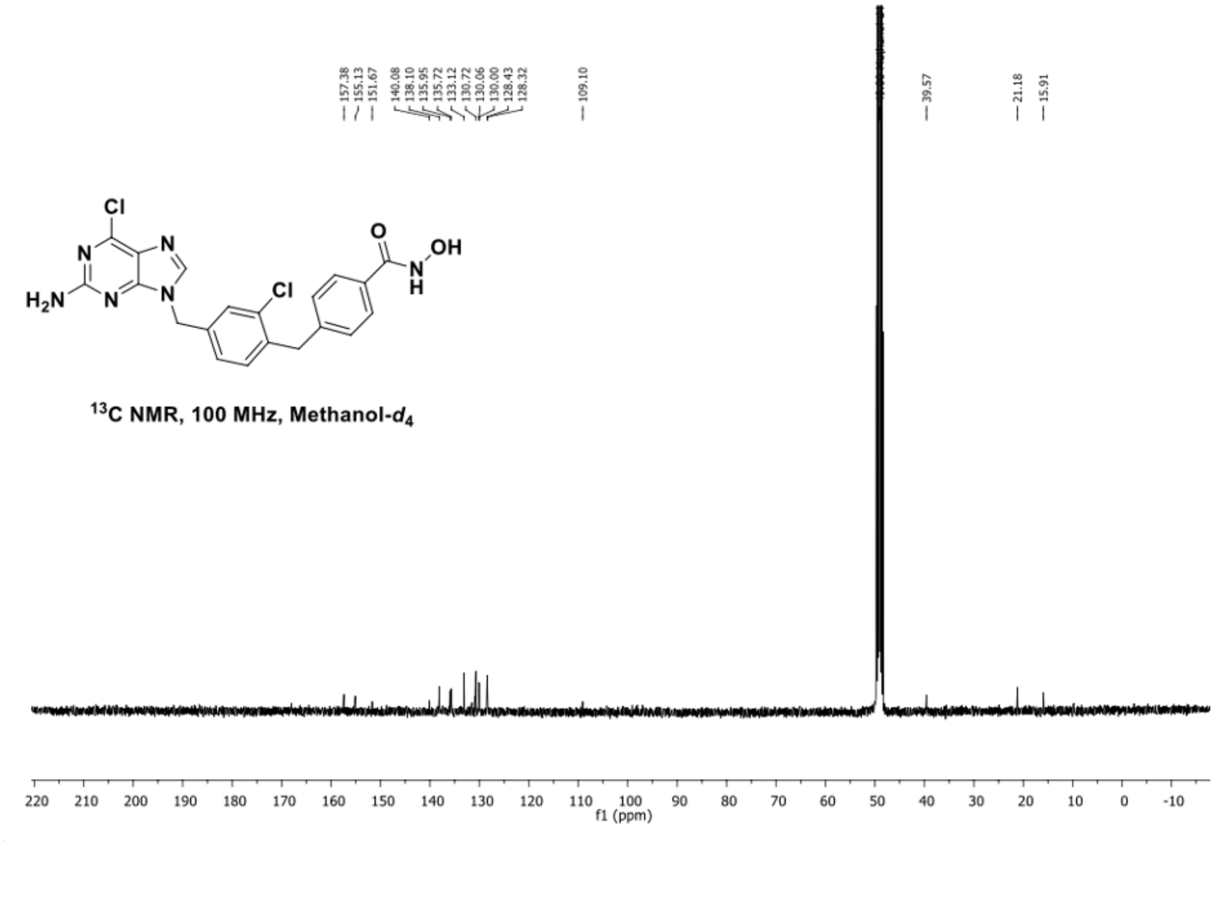

^{13}C NMR, 100 MHz, Methanol-d_4

1H NMR and 13C NMR spectra of compound 6.

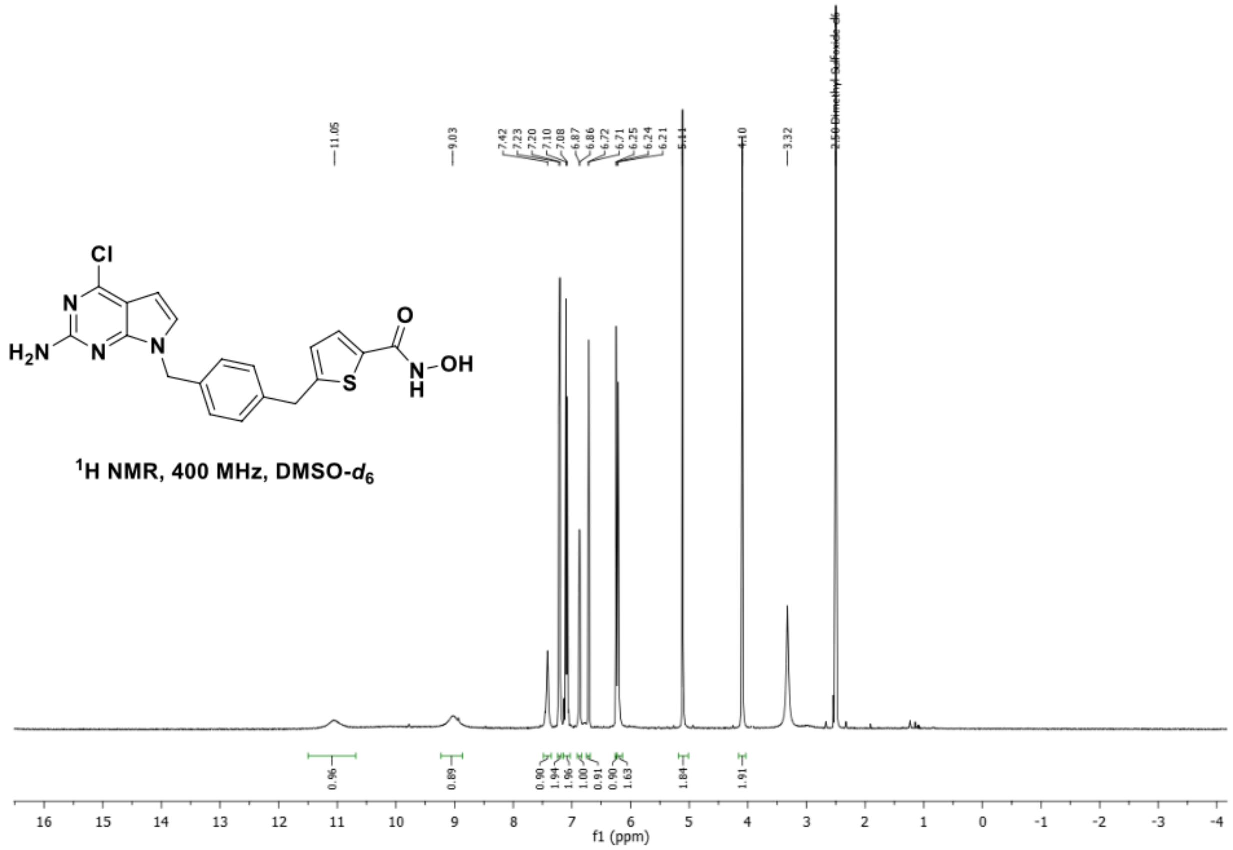

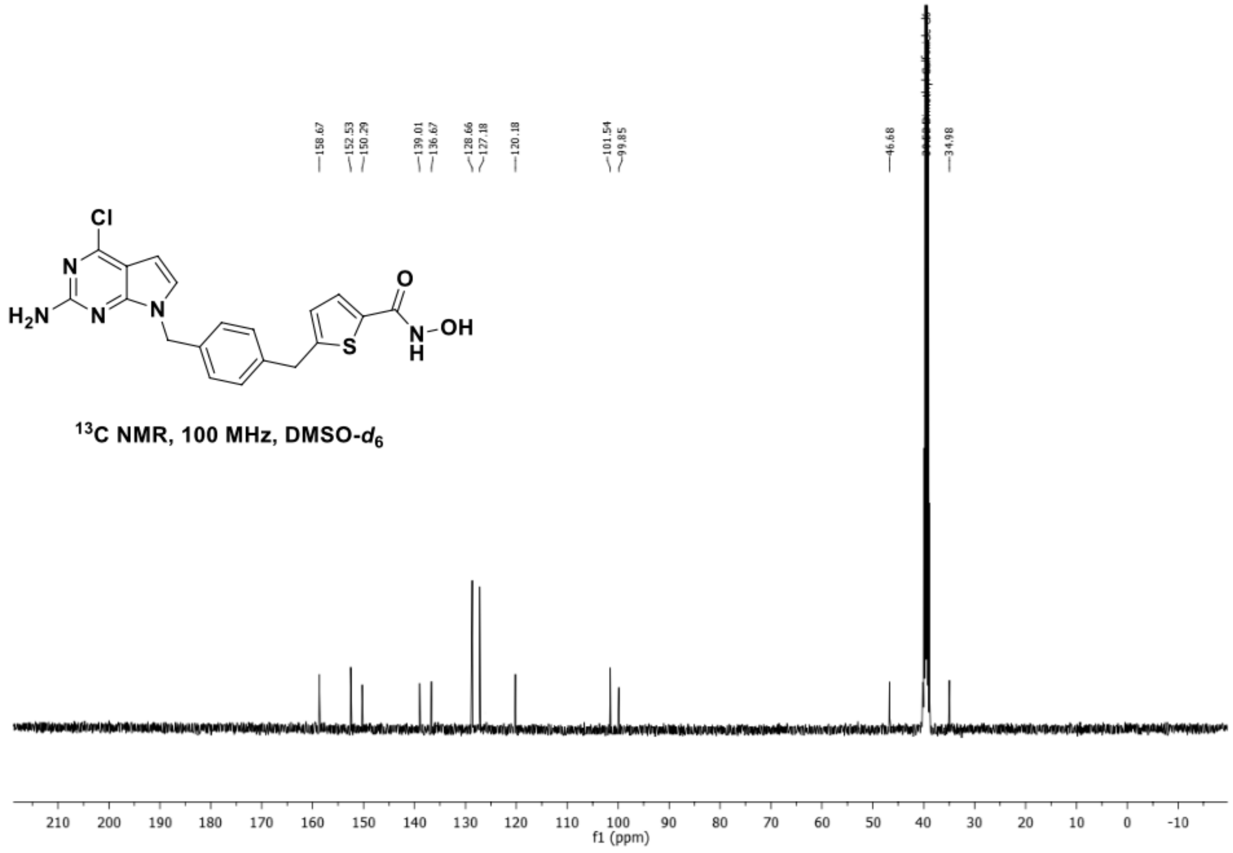

1H NMR and 13C NMR spectra of compound 7.

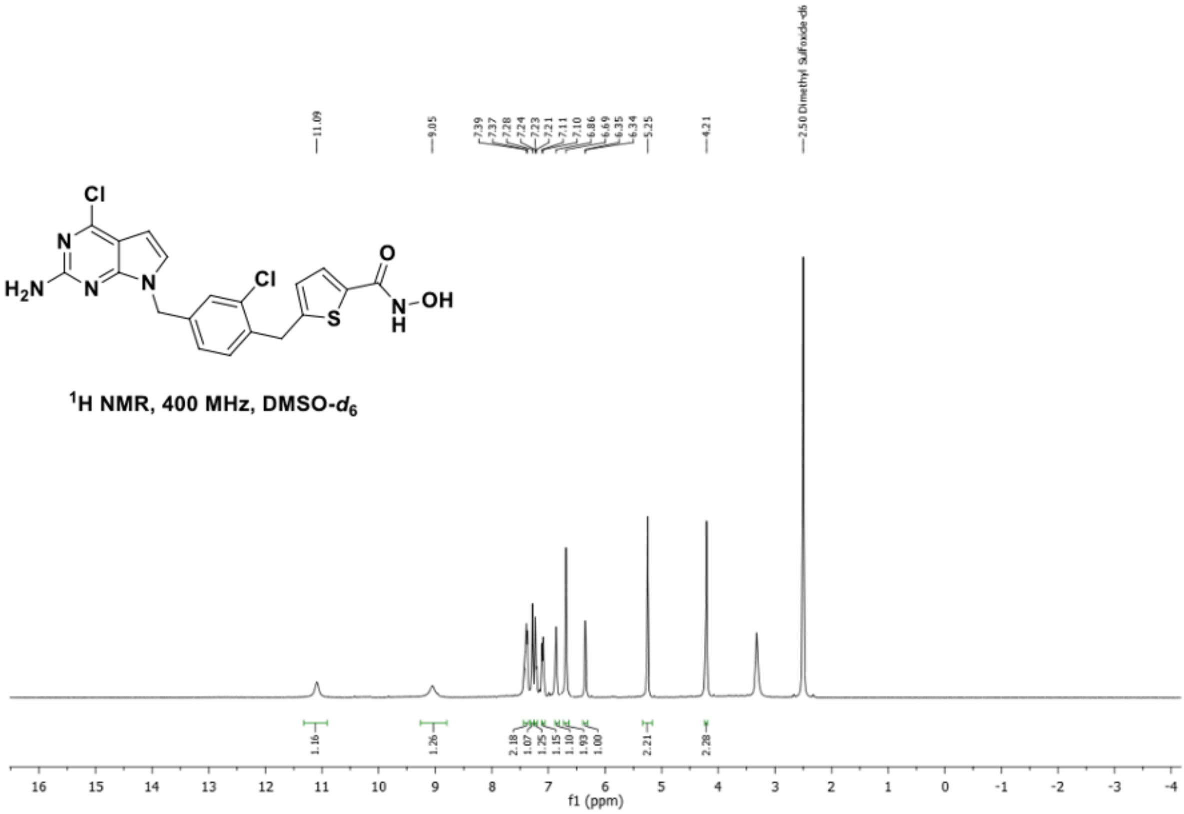

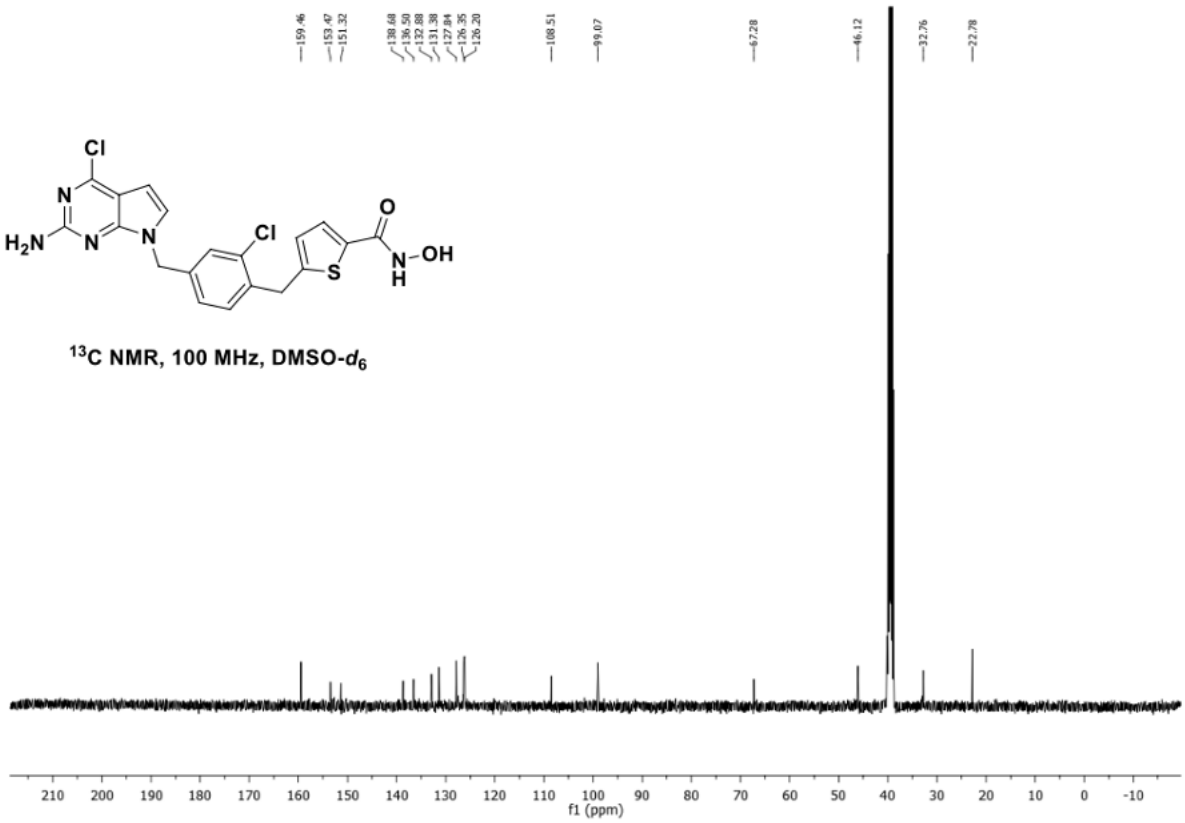

1H NMR and 13C NMR spectra of compound 8.

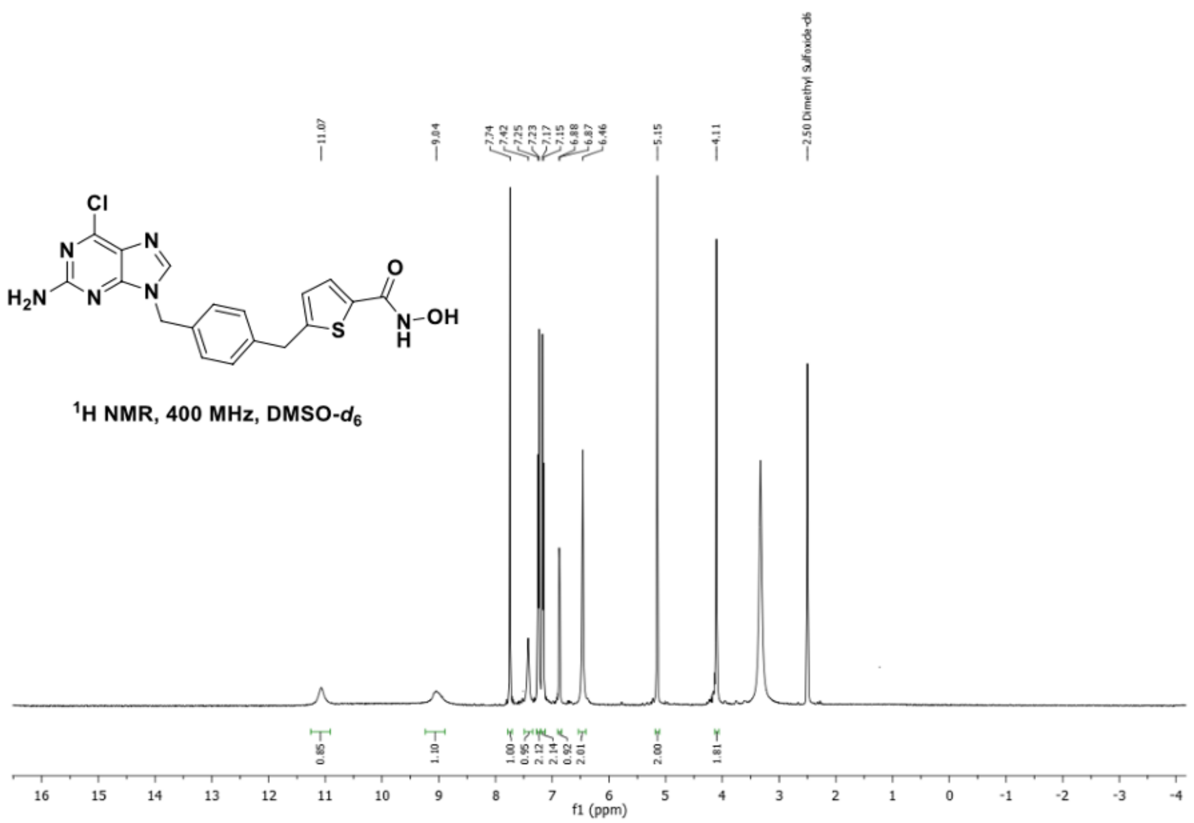

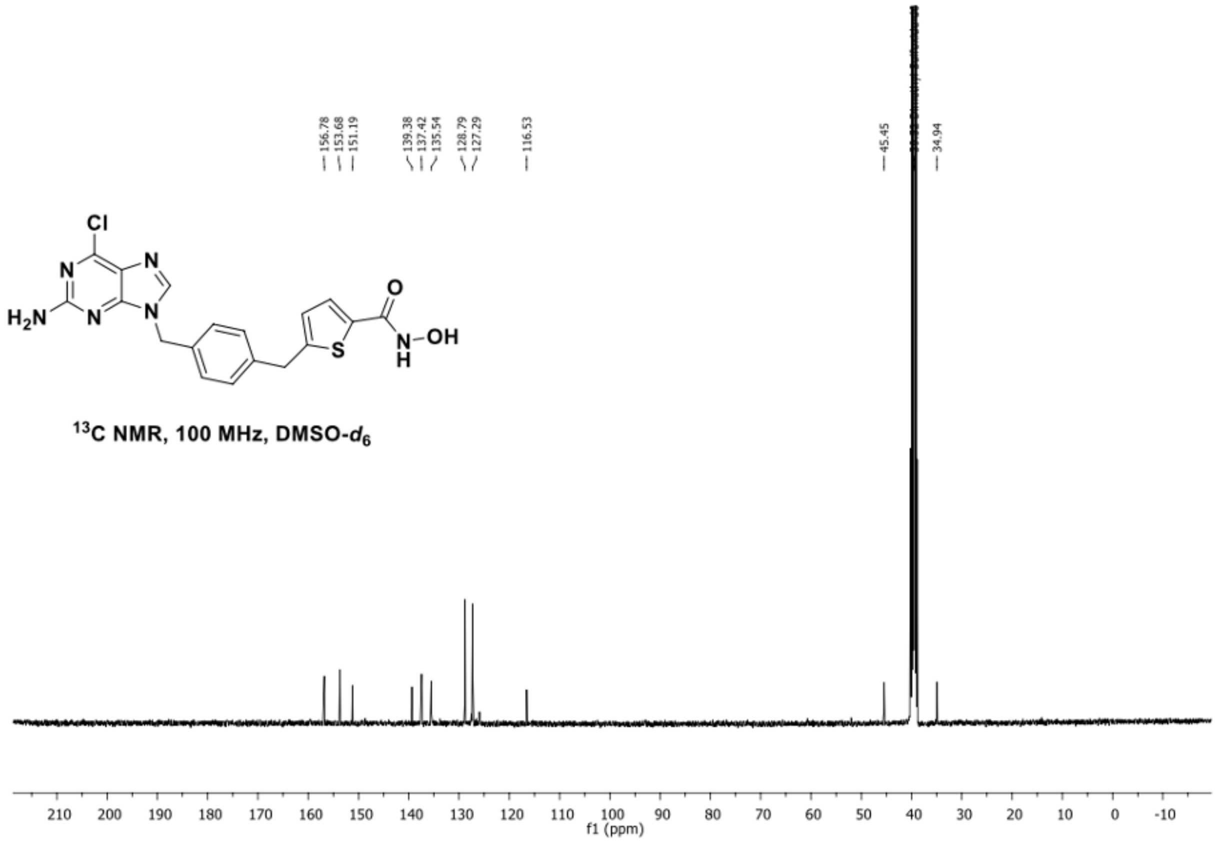

1H NMR and 13C NMR spectra of compound 9.

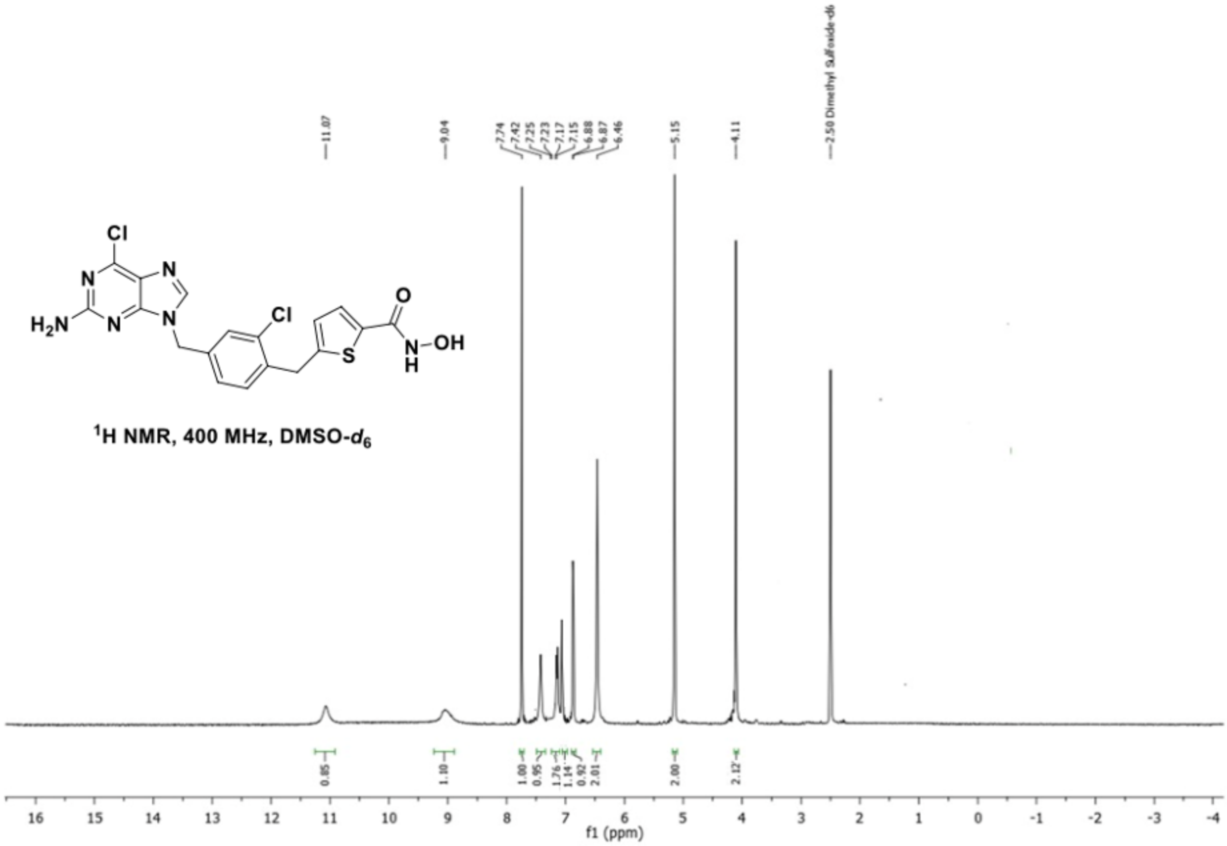

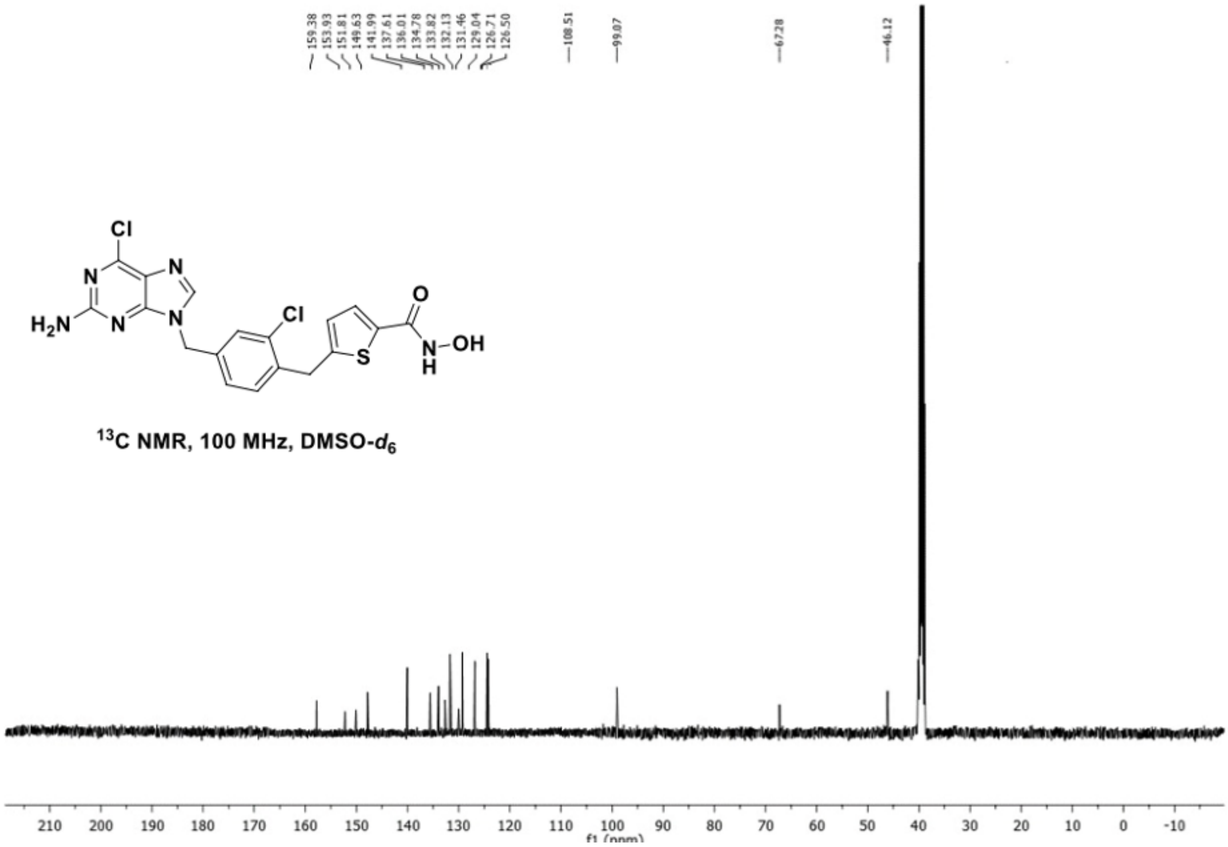

1H NMR and 13C NMR spectra of compound 10.

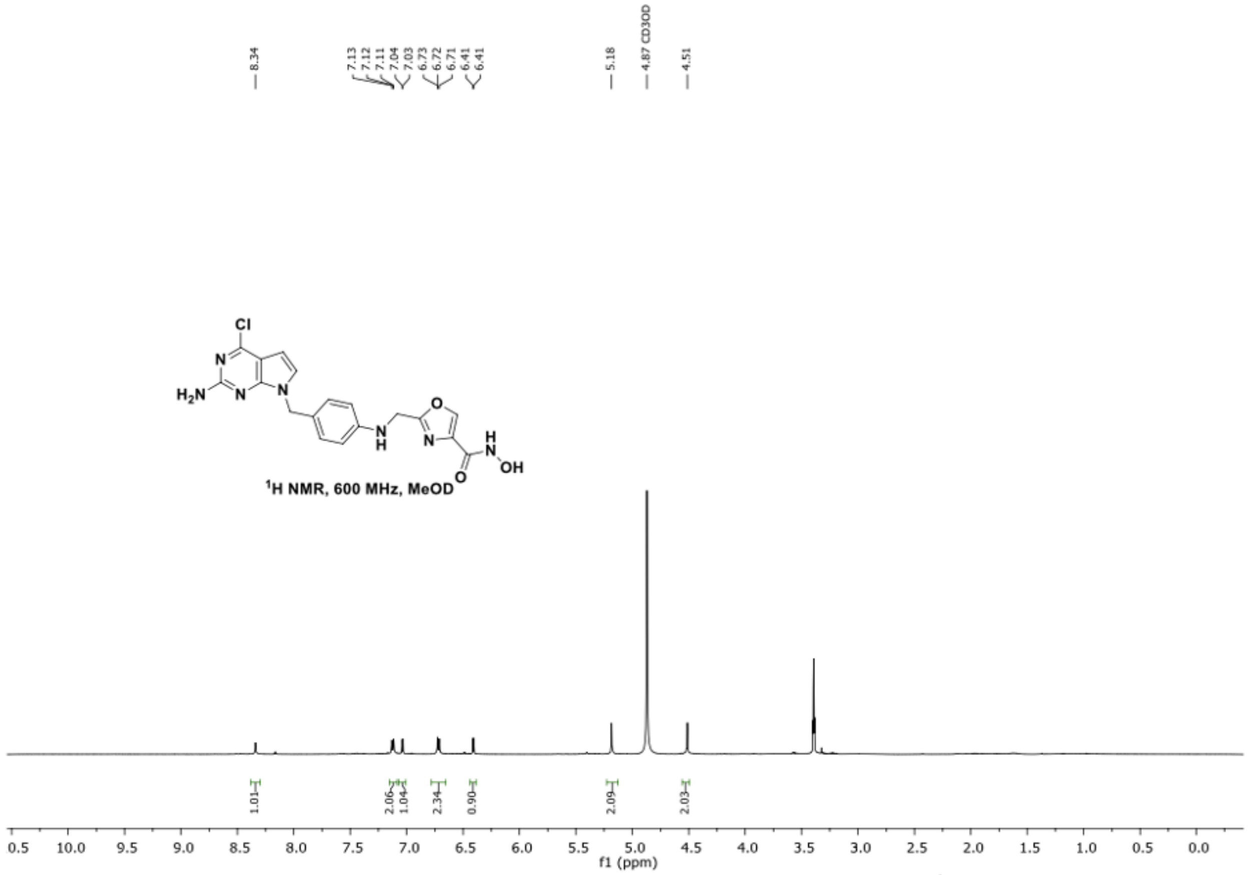

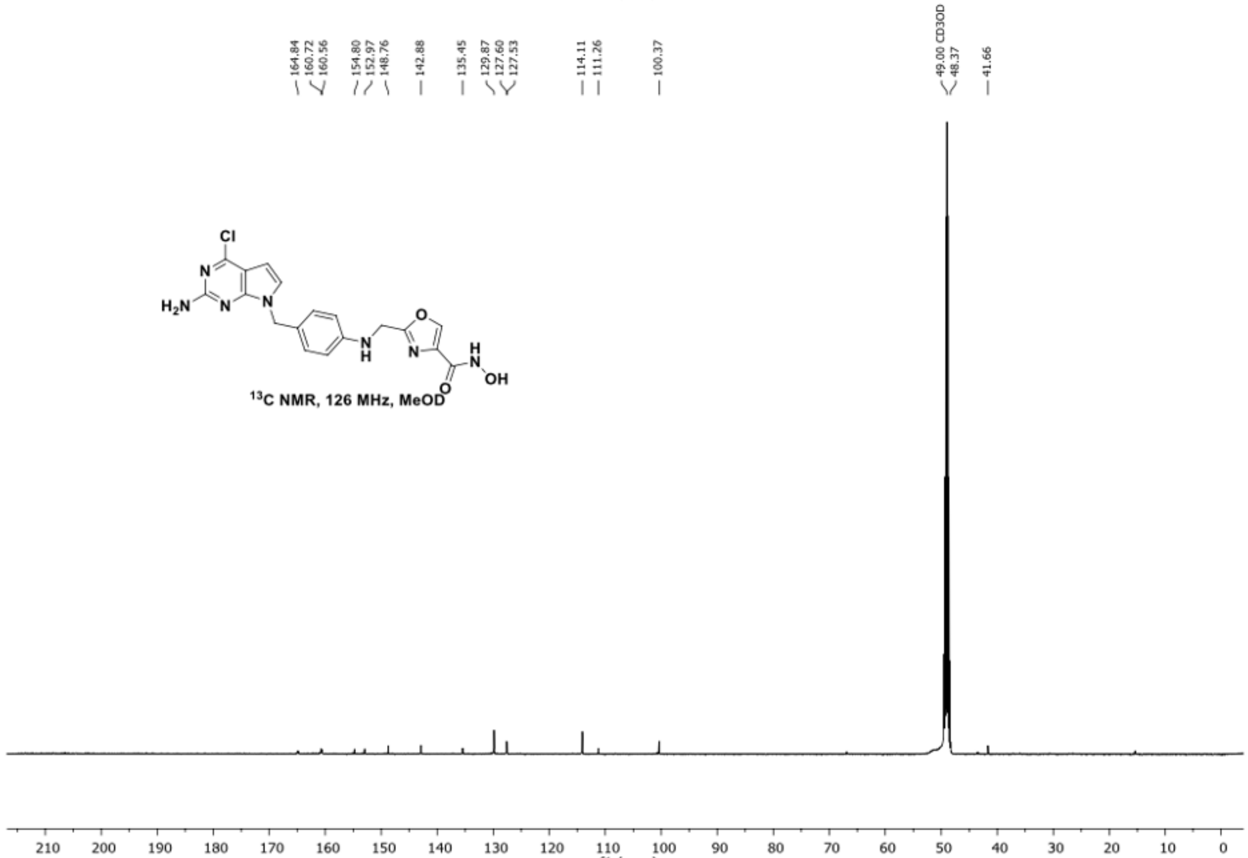

1H NMR and 13C NMR spectra of compound 11.

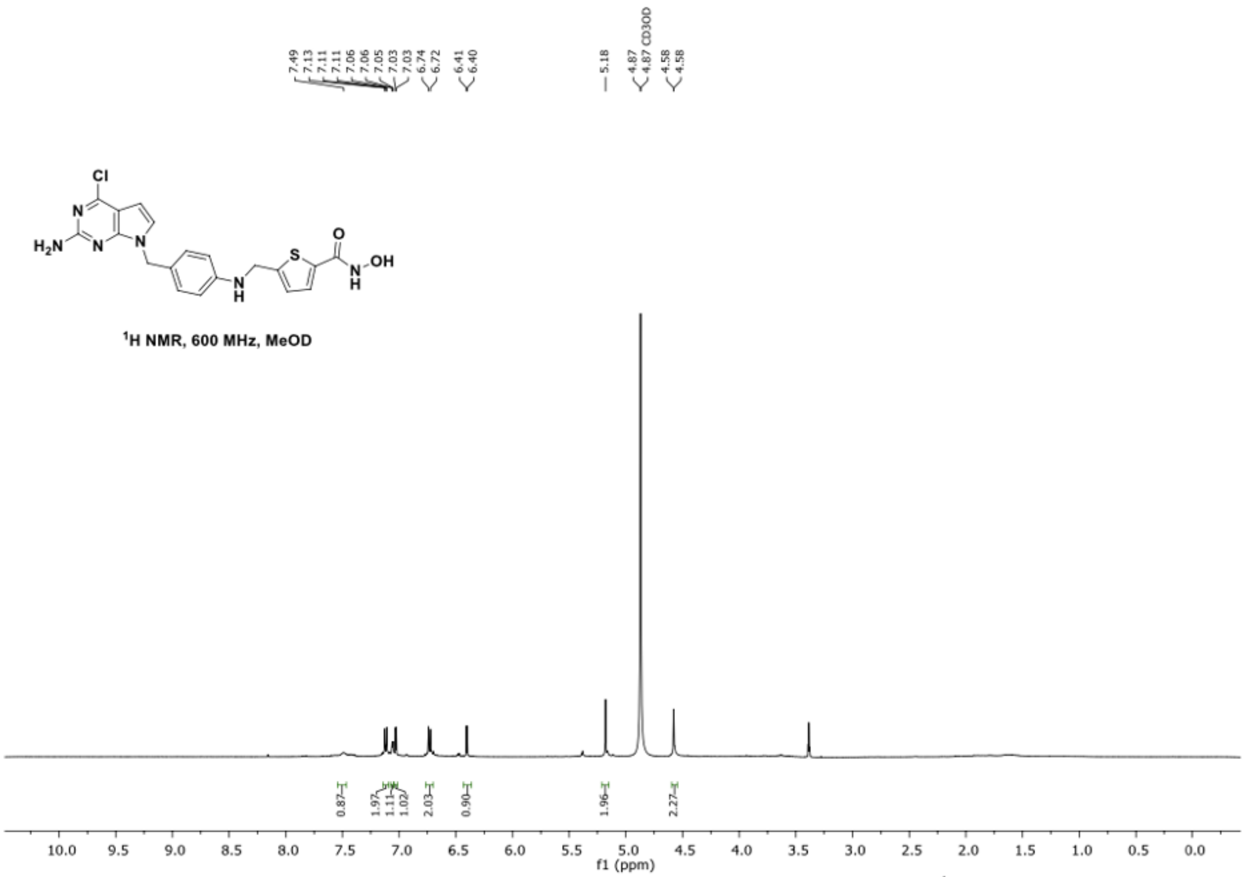

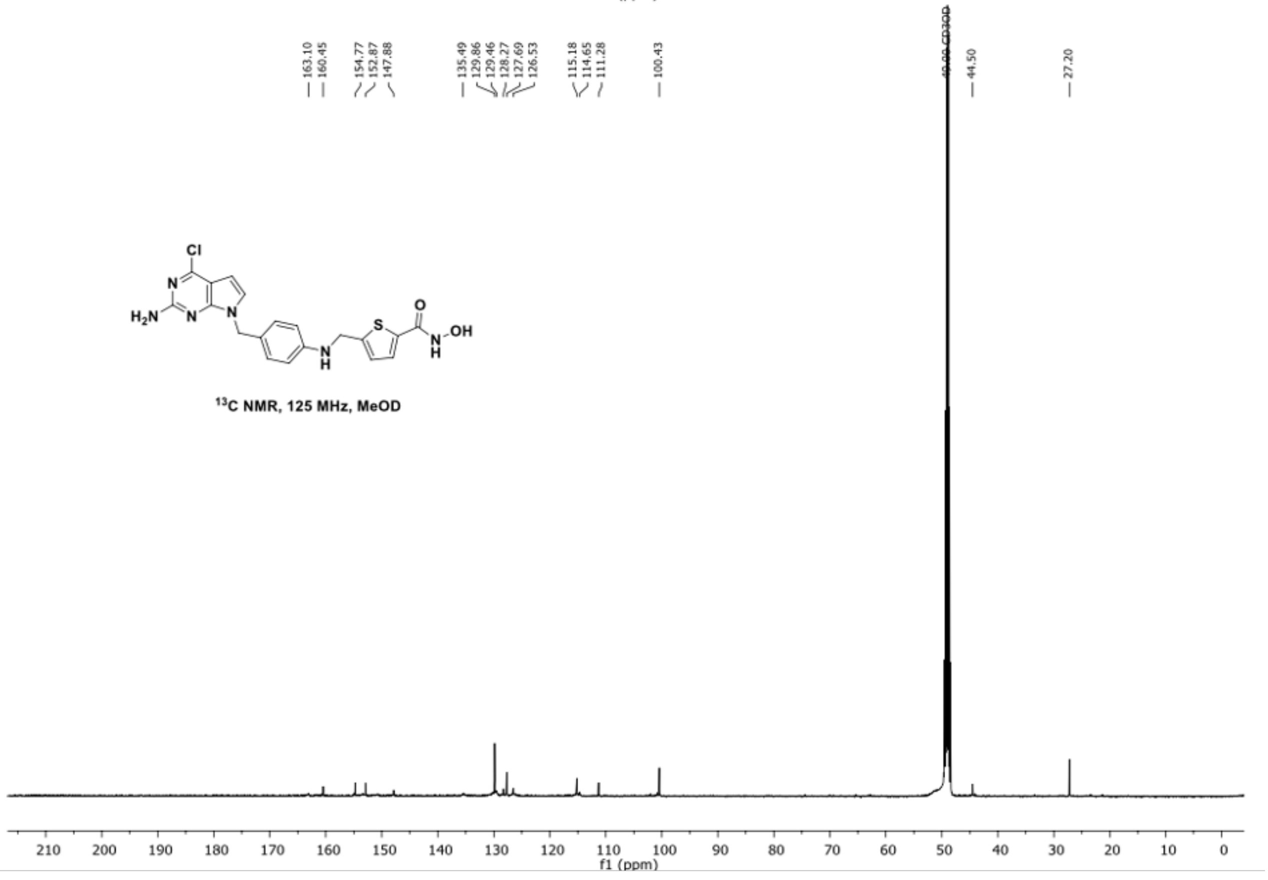

1H NMR and 13C NMR (APT) spectra of compound 12.

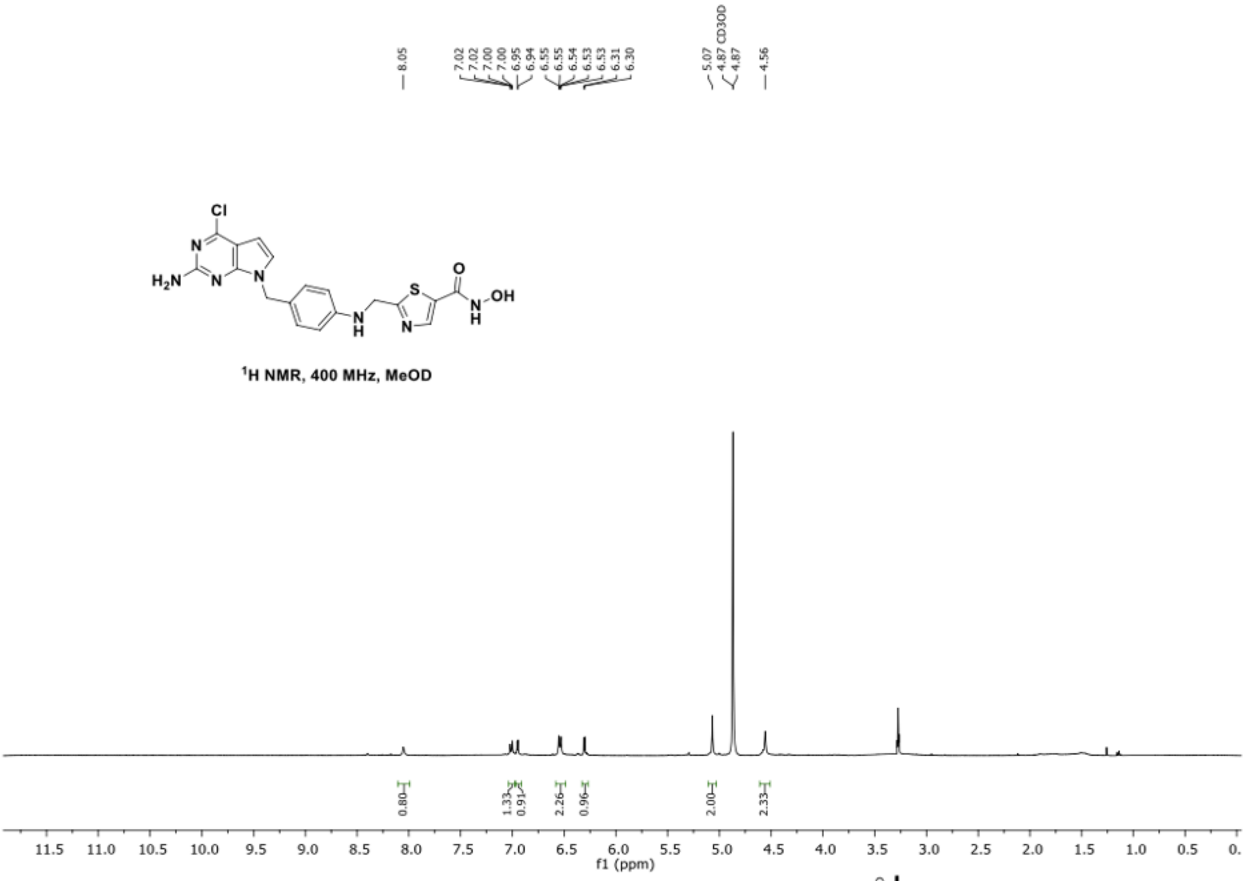

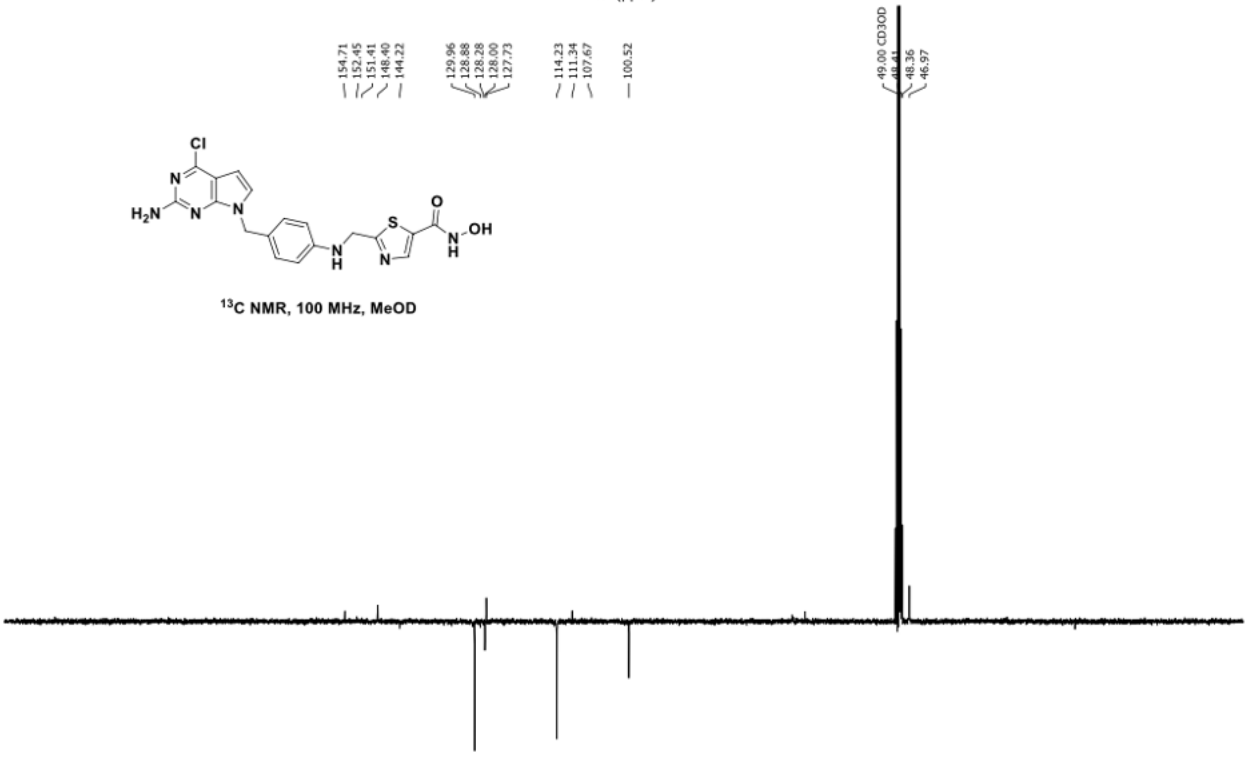

1H NMR and 13C NMR spectra of compound 13.

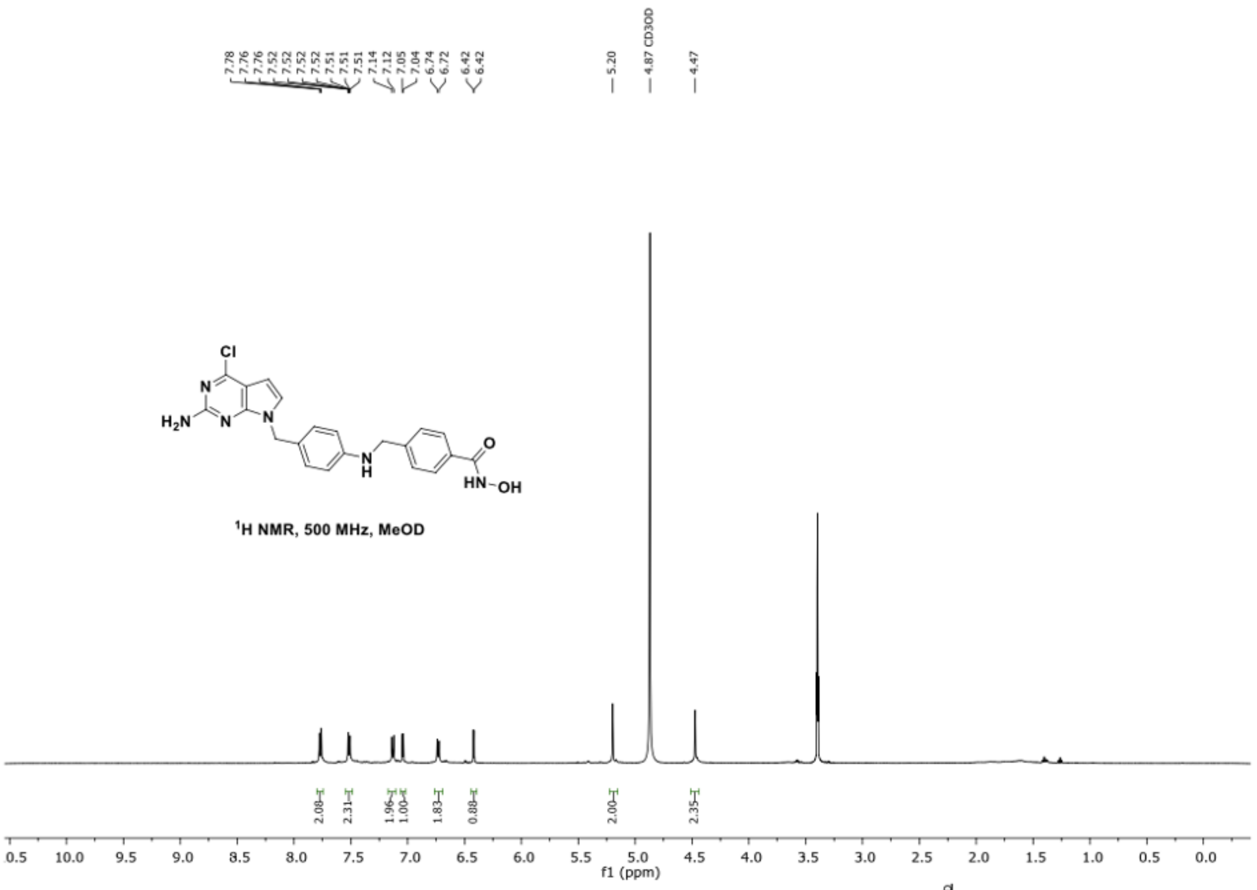

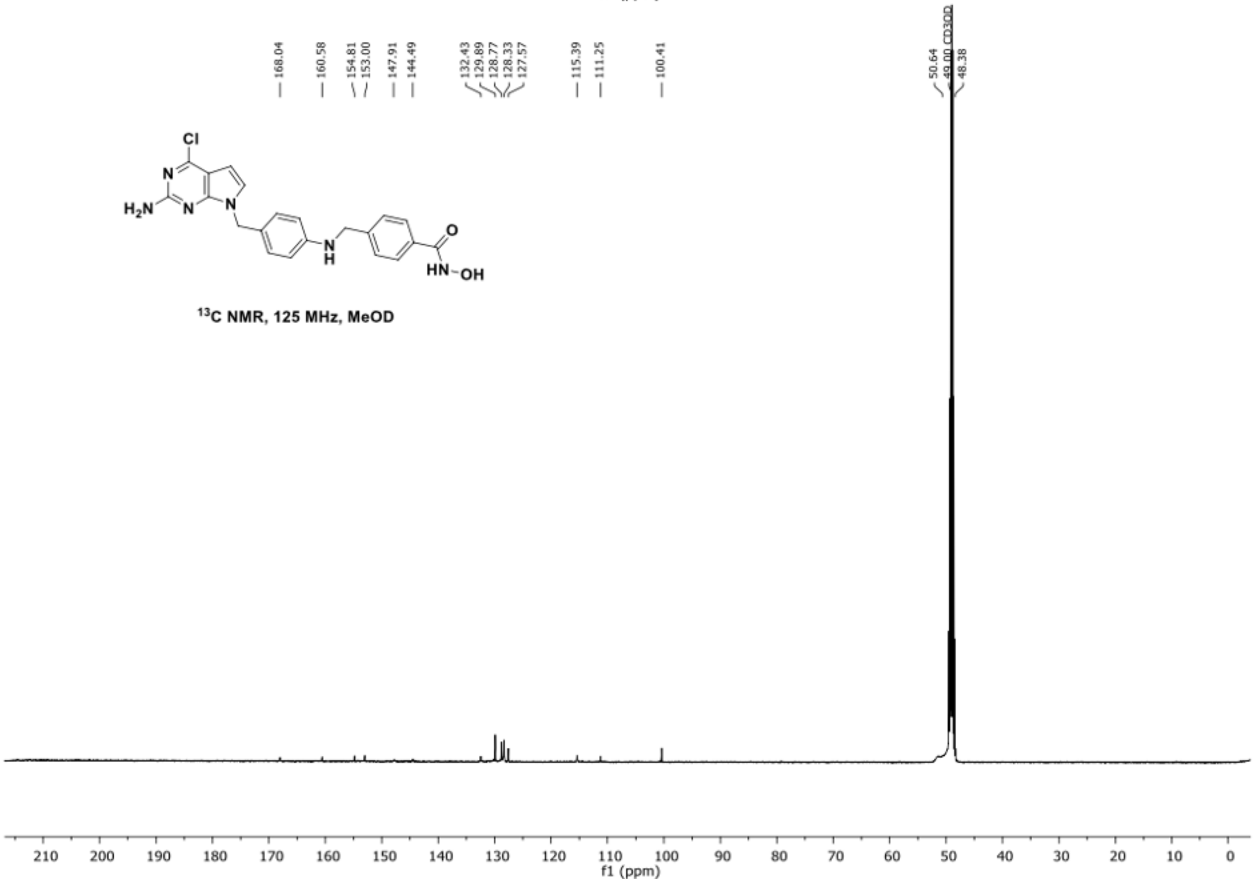

1H NMR and 13C NMR spectra of compound 14.

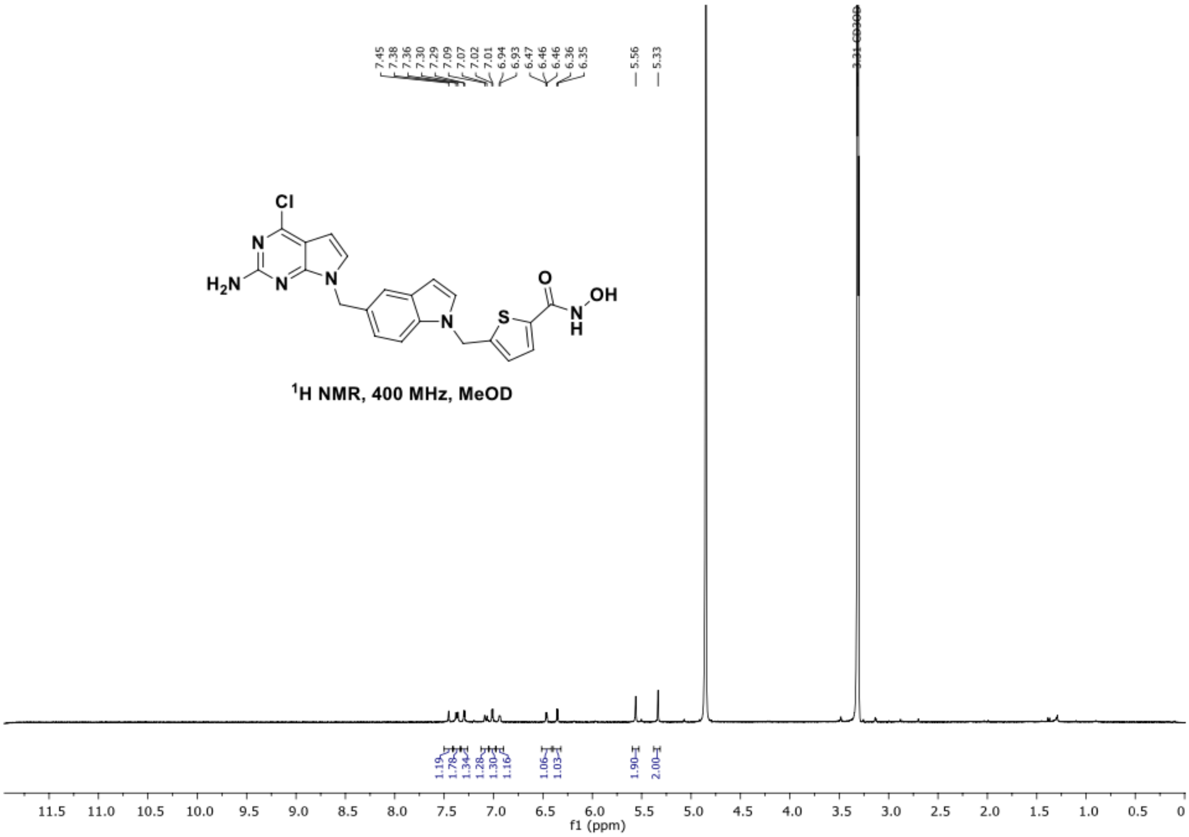

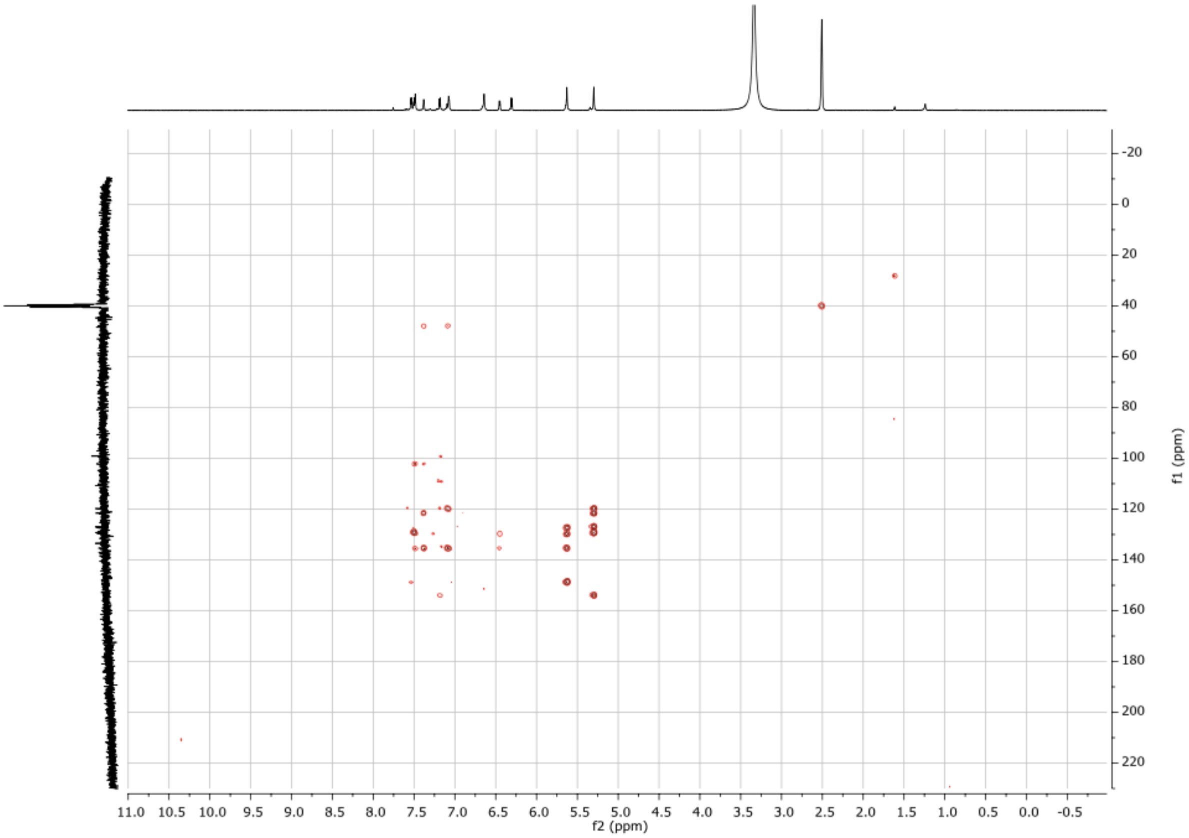

1H NMR and 13C NMR (APT) spectra of compound 15.

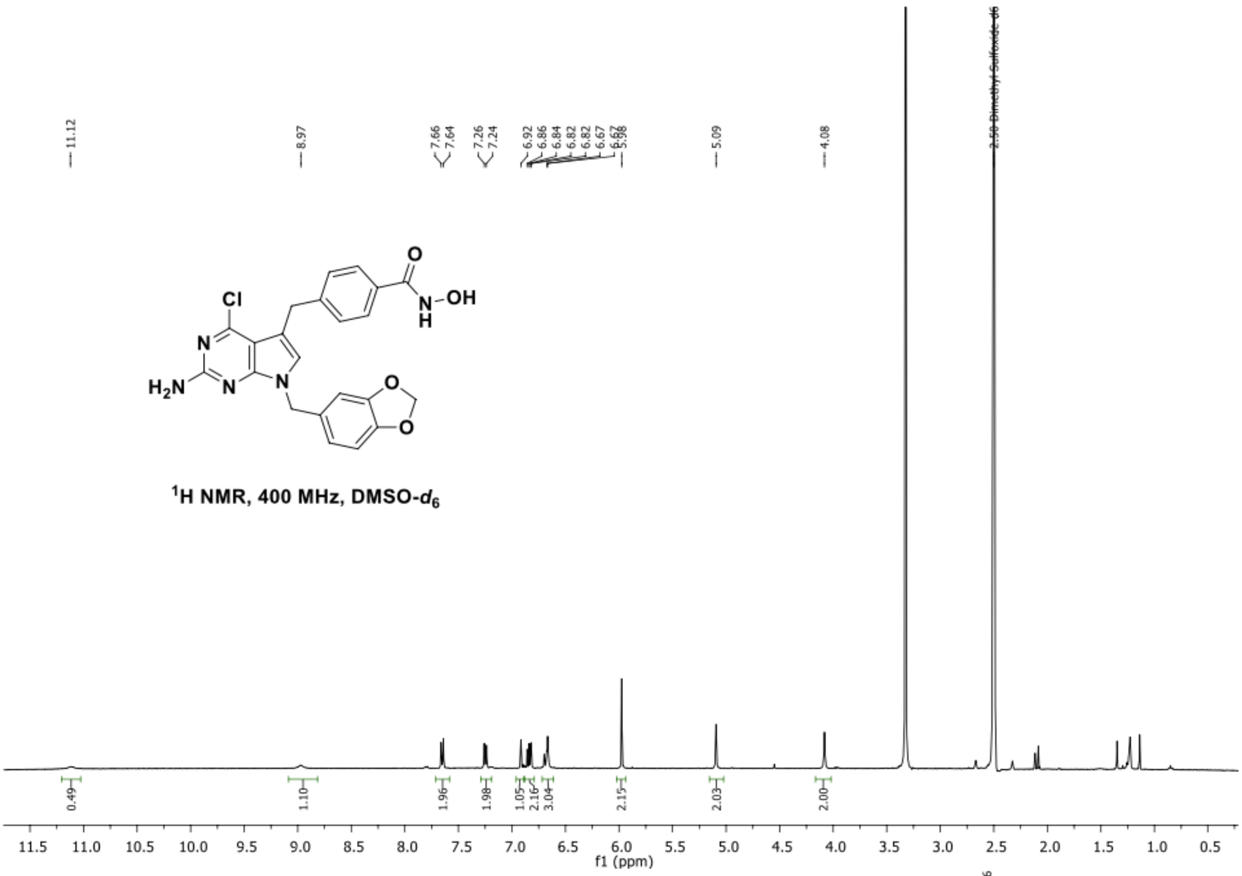

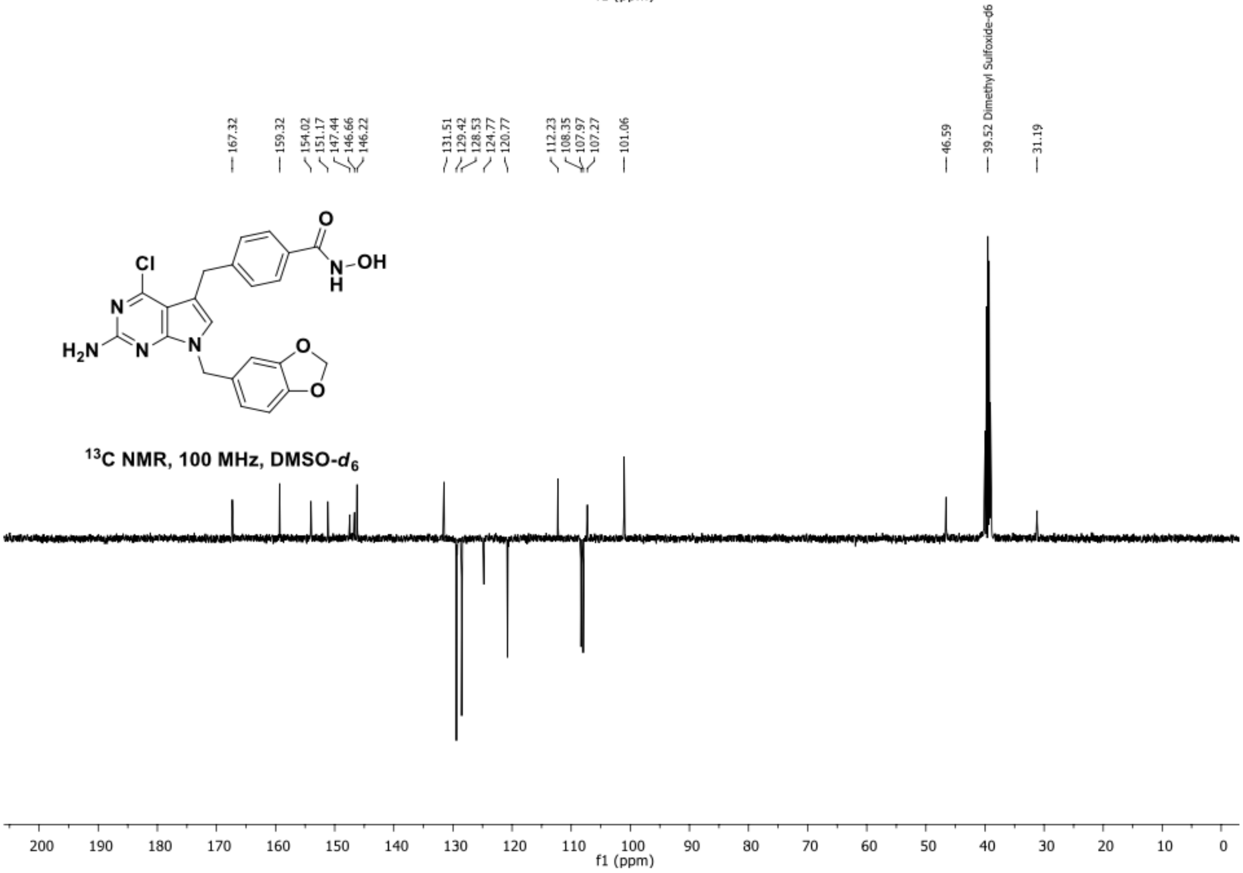

1H NMR and 13C NMR (APT) spectra of compound 16.

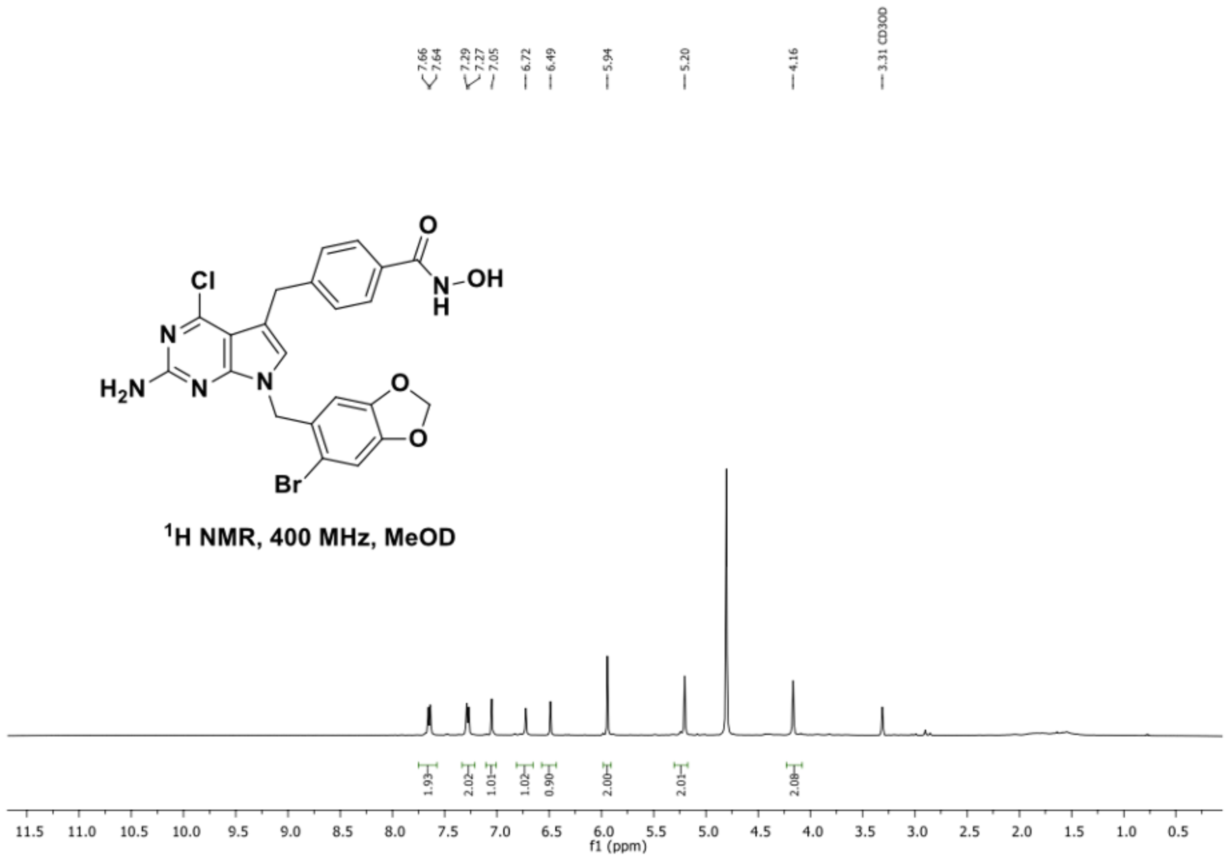

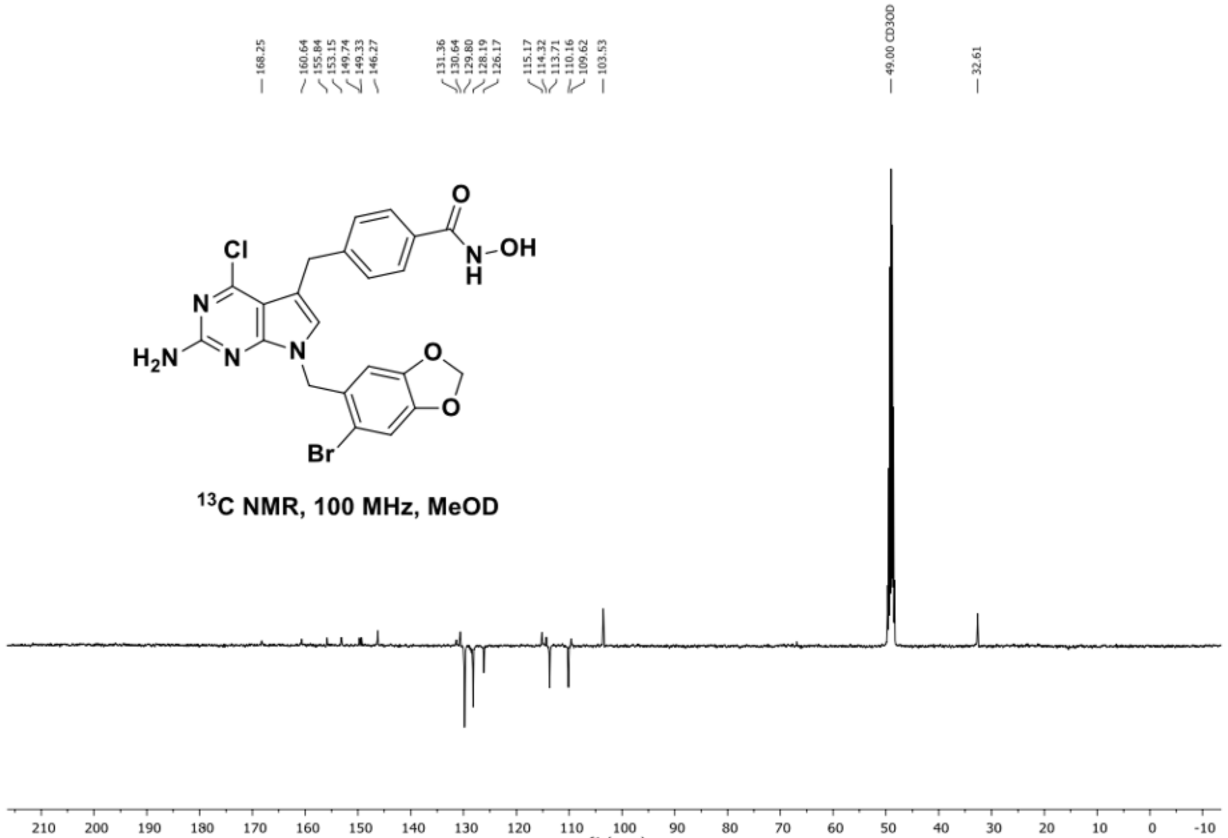

1H NMR and 13C NMR (APT) spectra of compound 17.

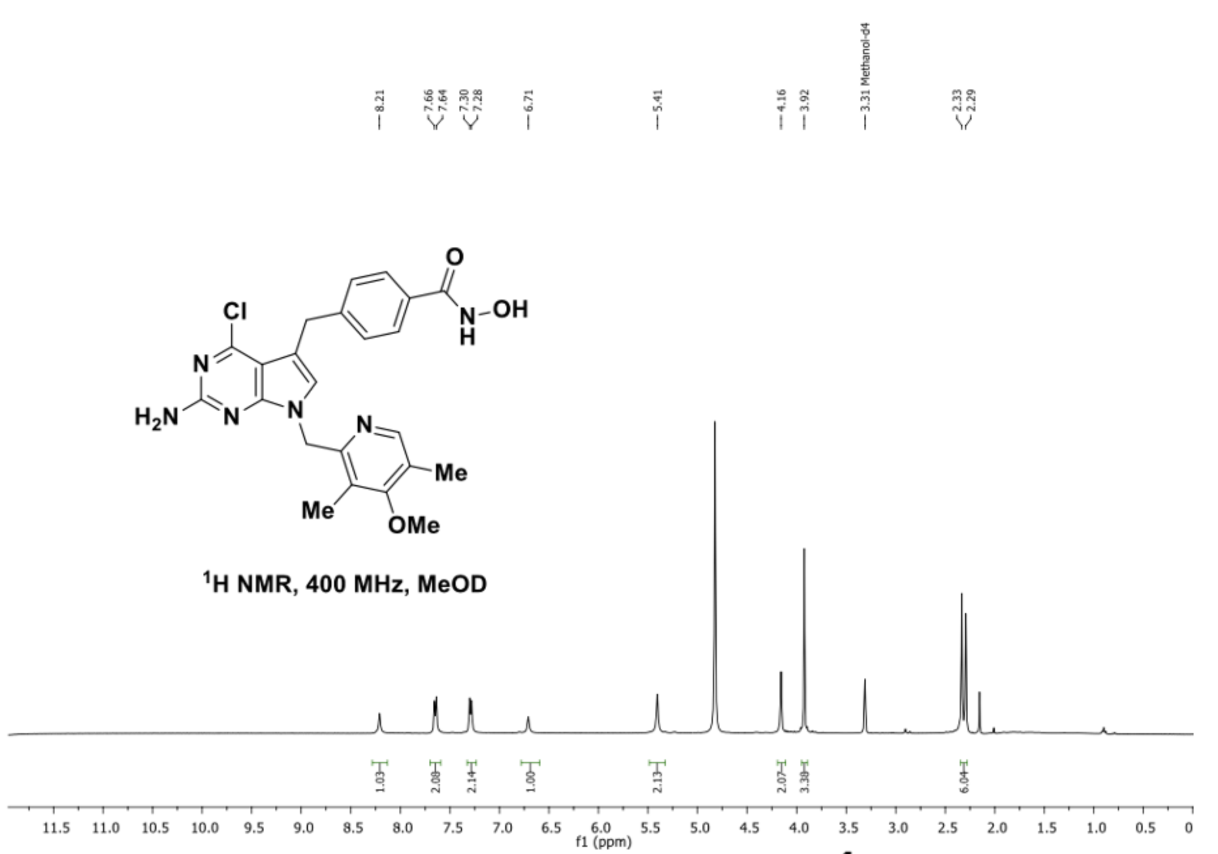

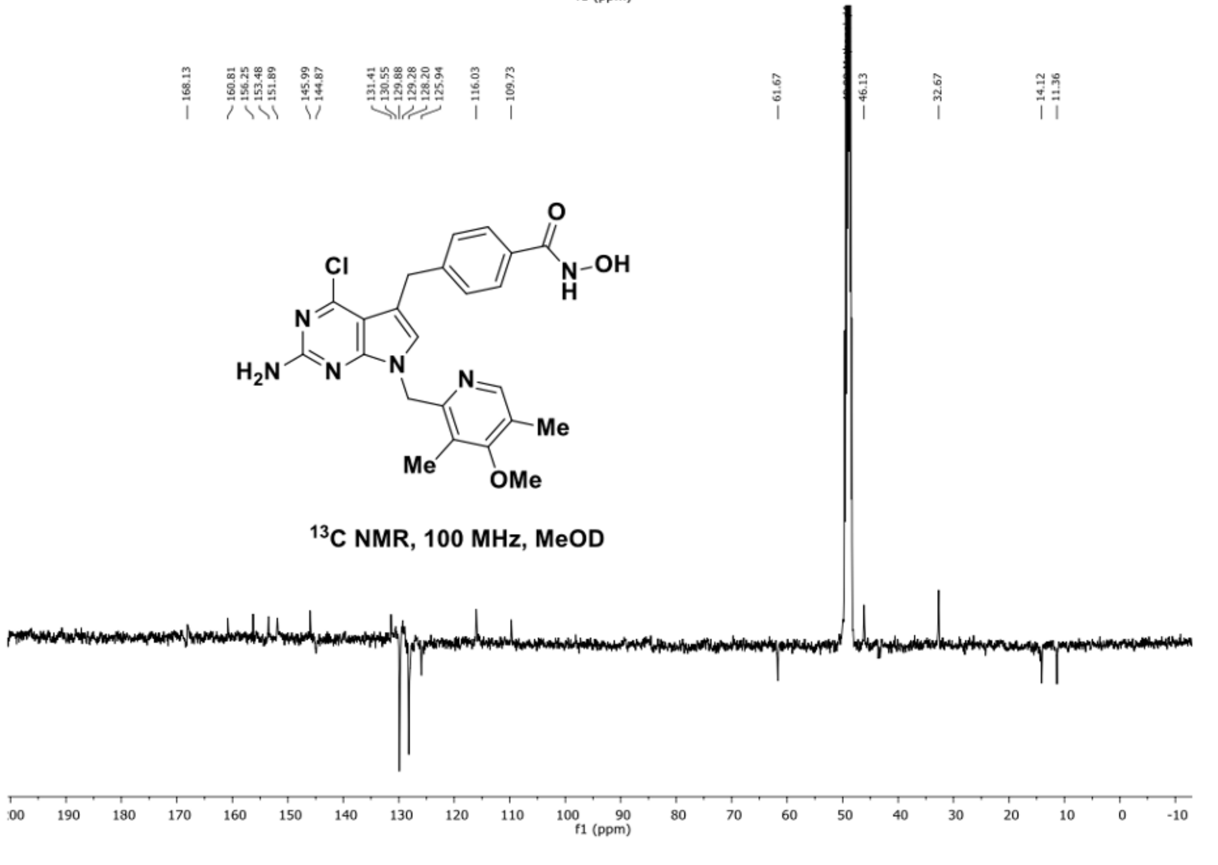

1H NMR and 13C NMR spectra of compound 18.

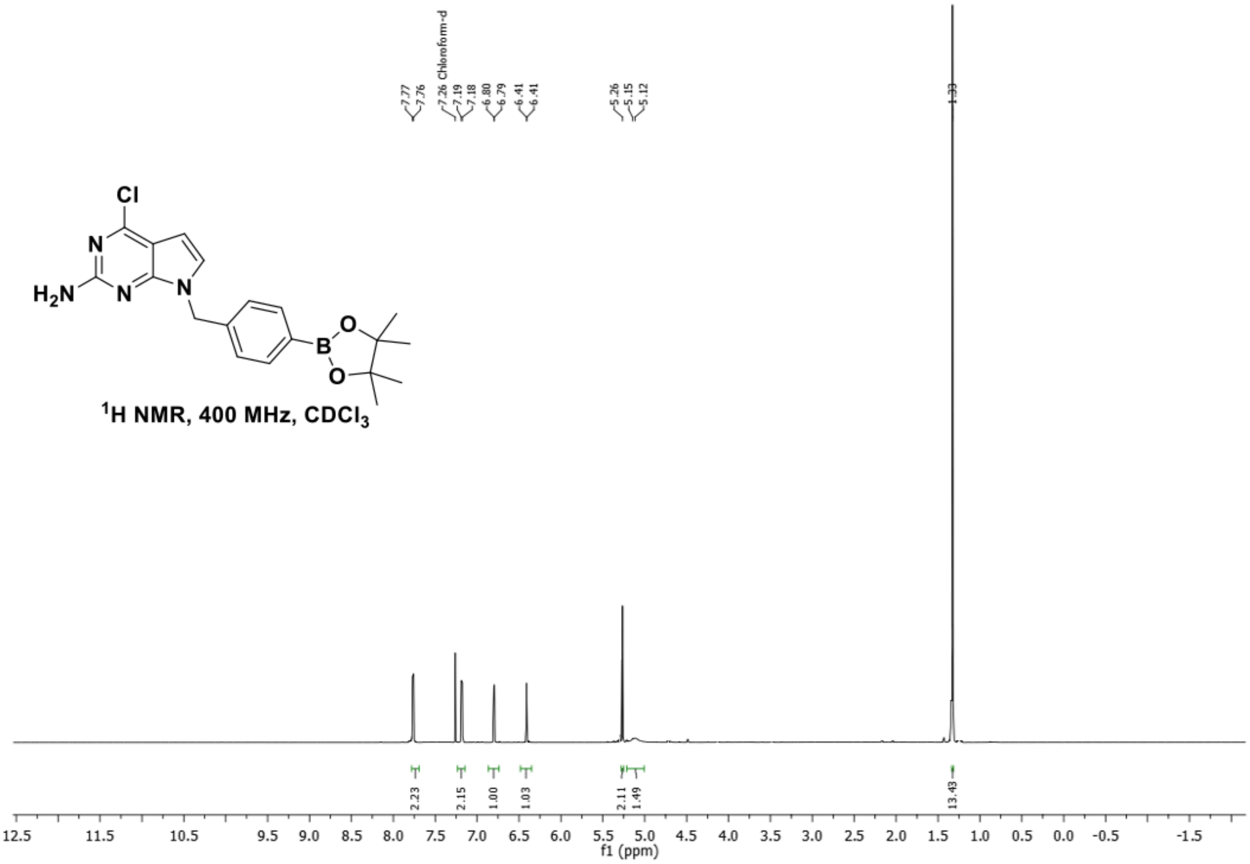

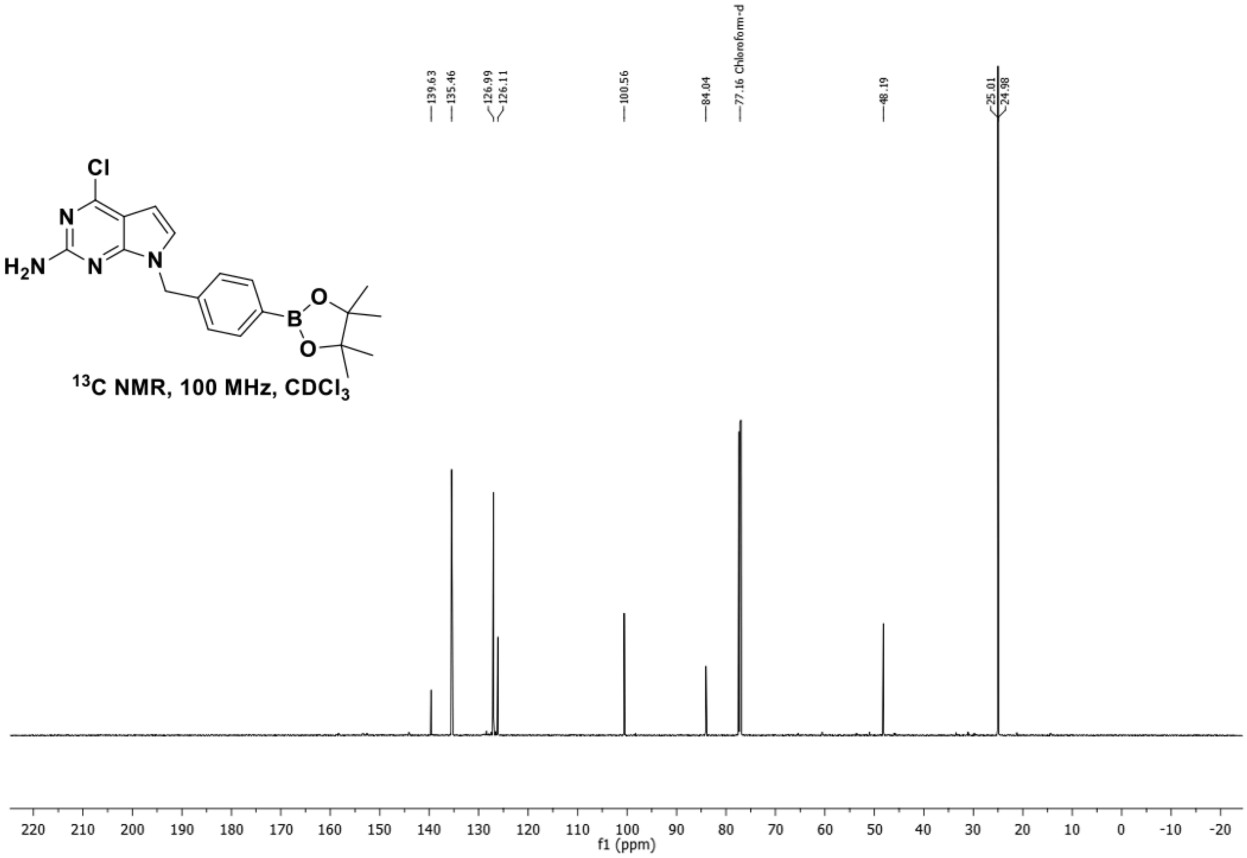

1H NMR and 13C NMR spectra of compound 19.

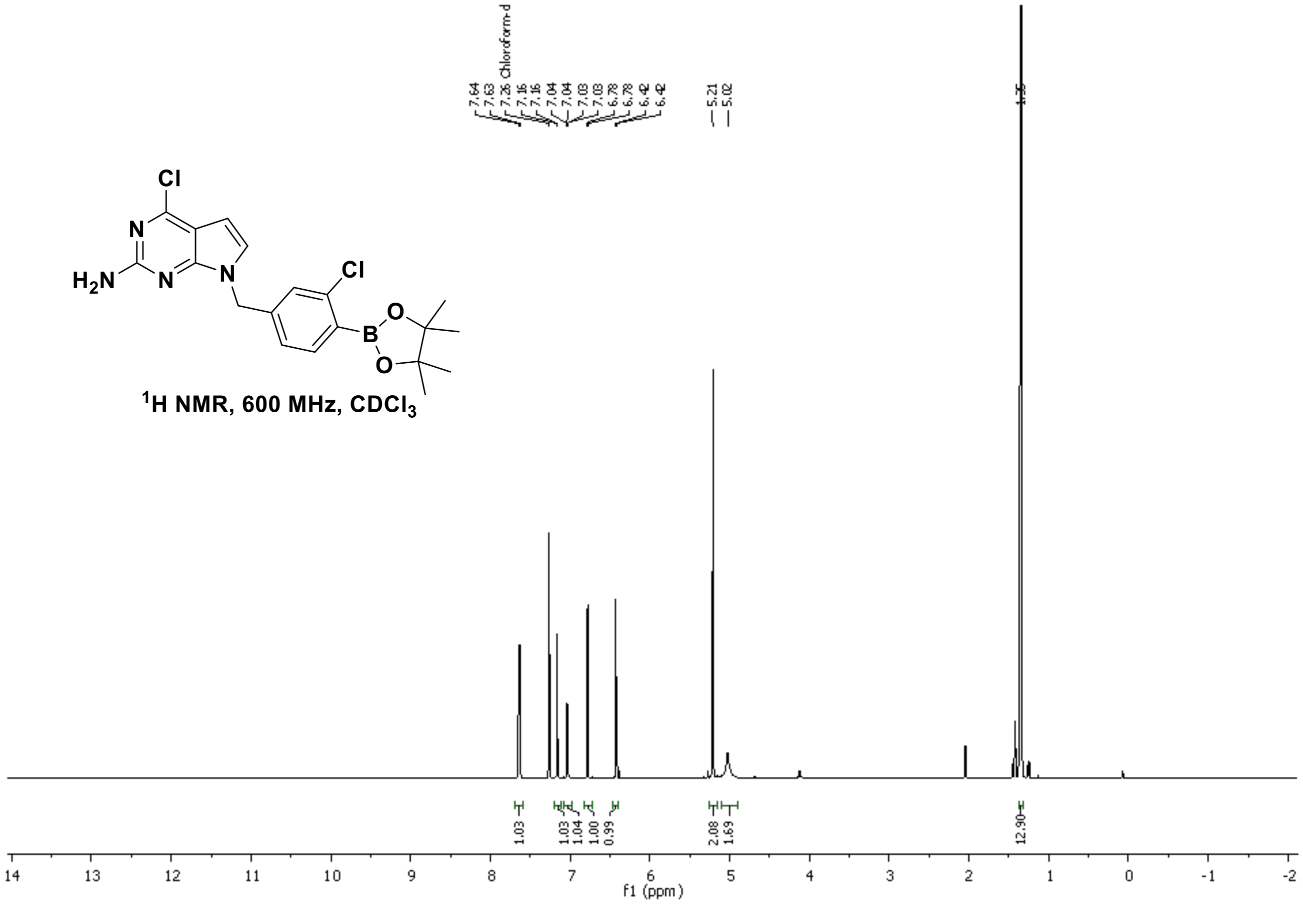

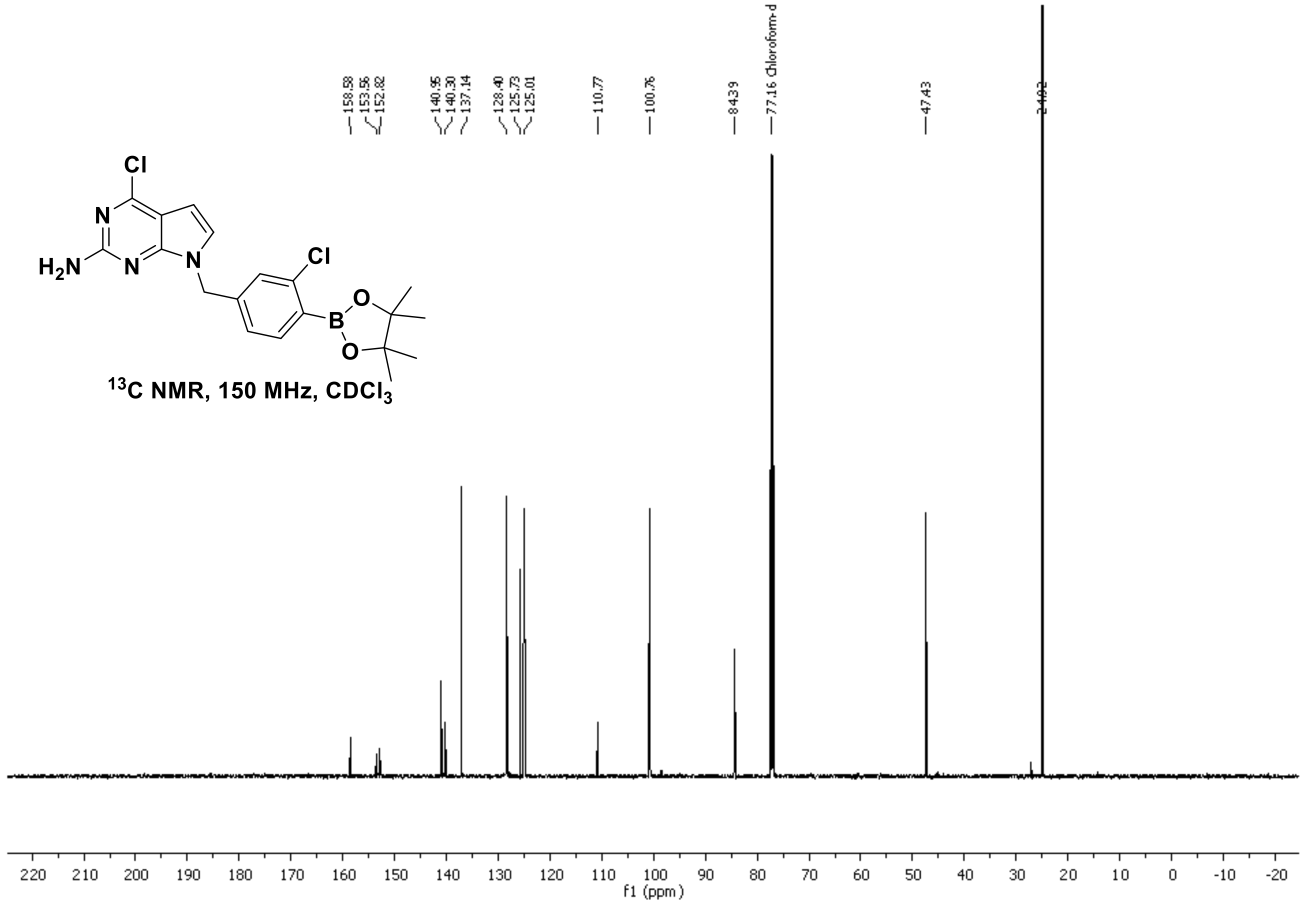

1H NMR and 13C NMR spectra of compound 20.

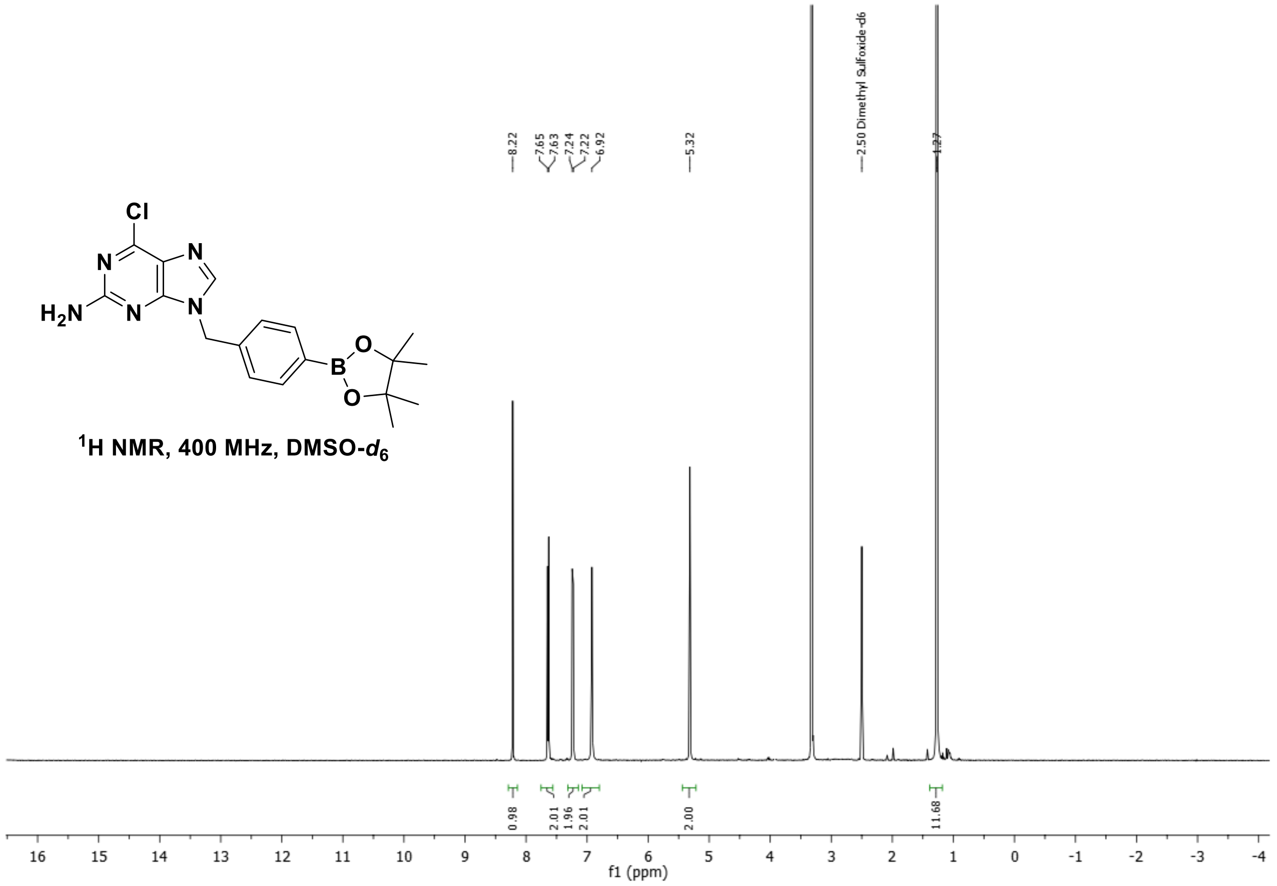

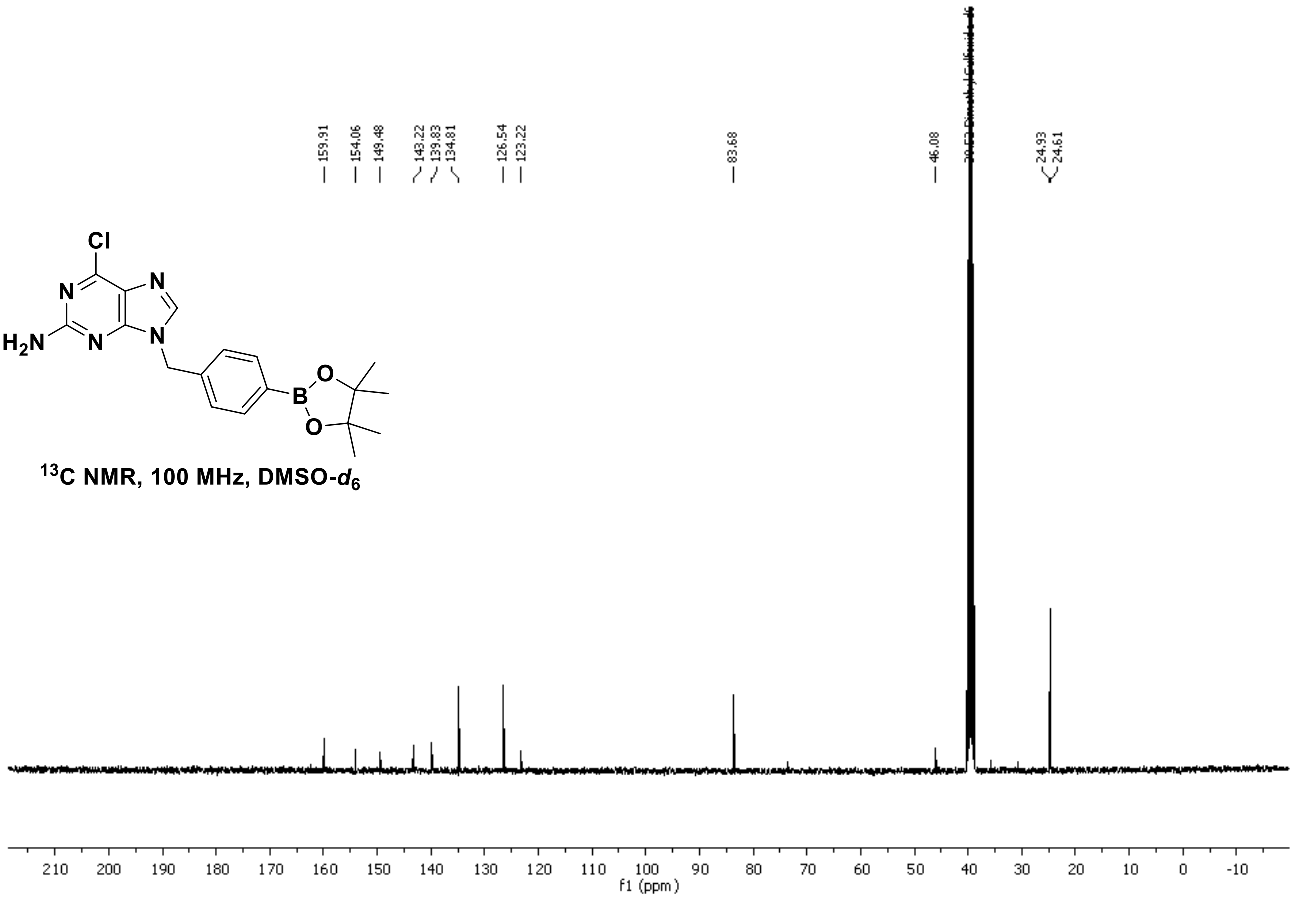

1H NMR and 13C NMR spectra of compound 21.

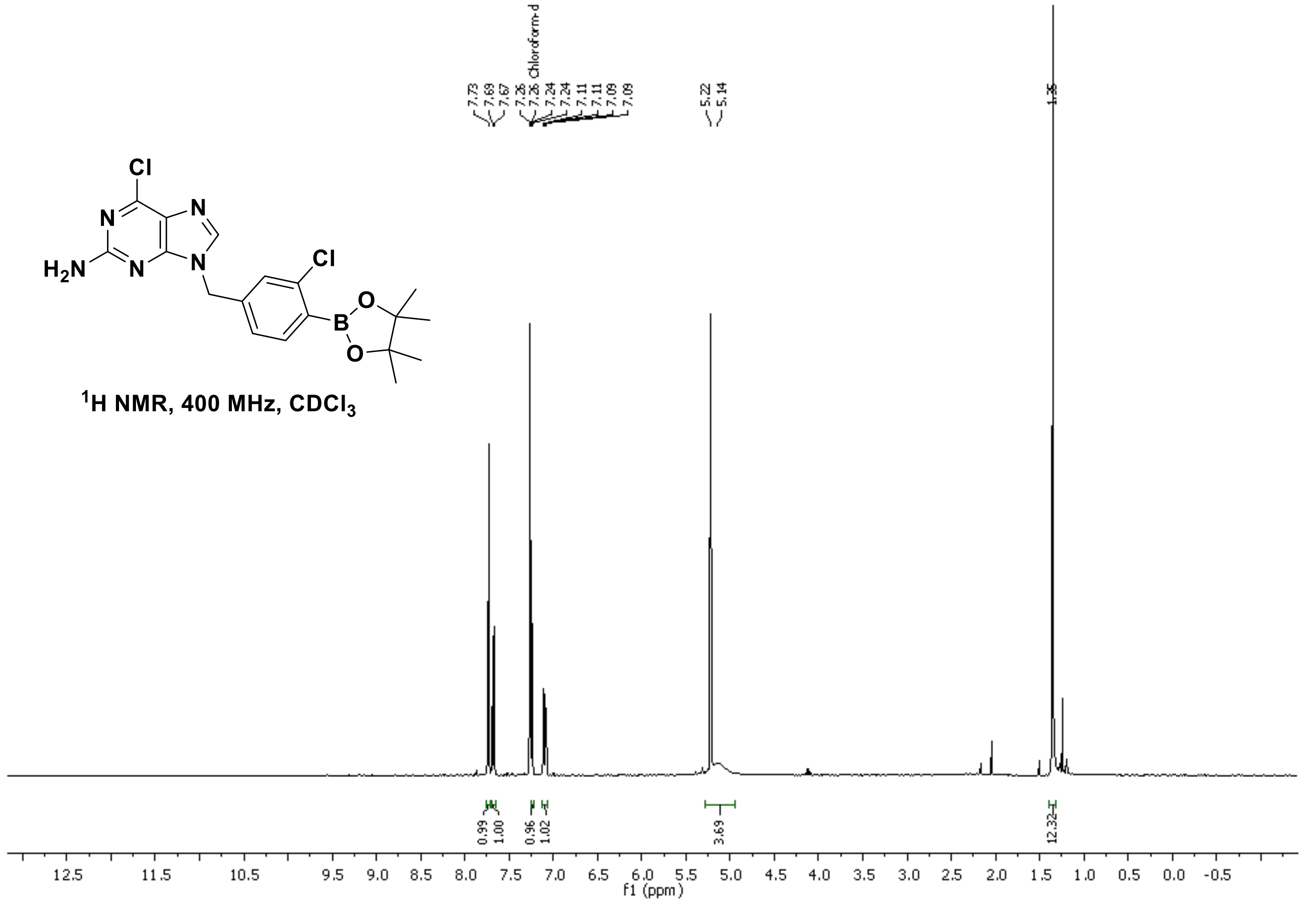

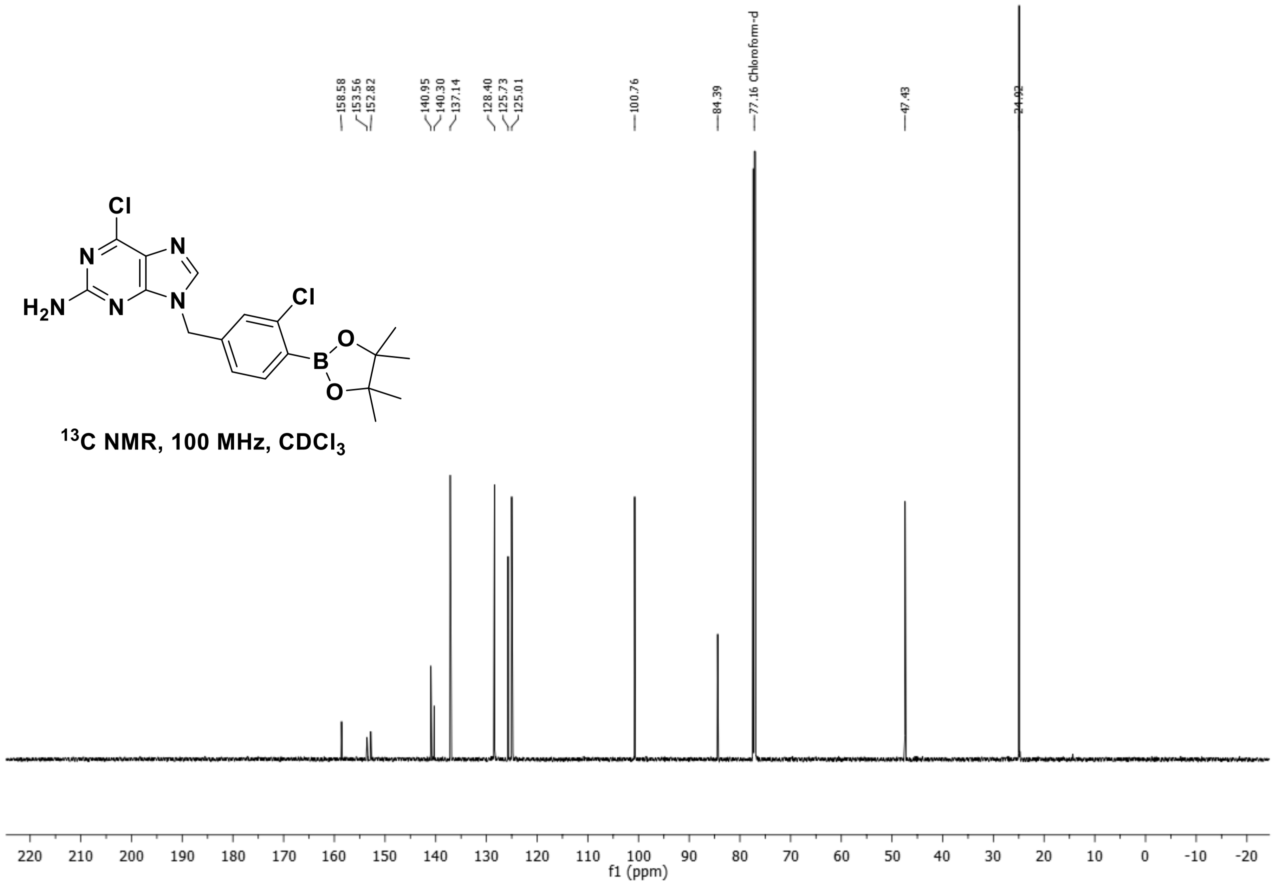

1H NMR and 13C NMR spectra of compound 22.

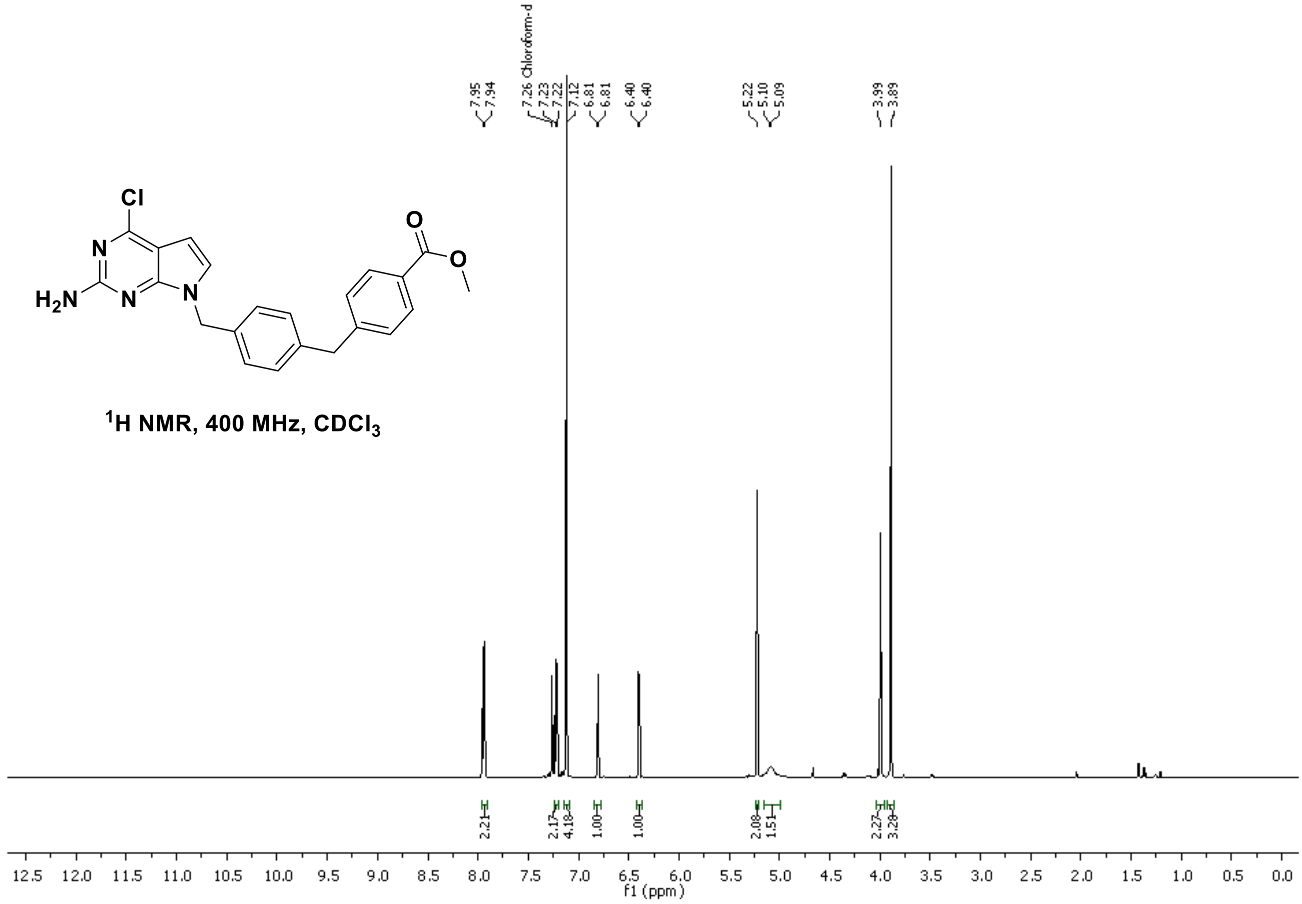

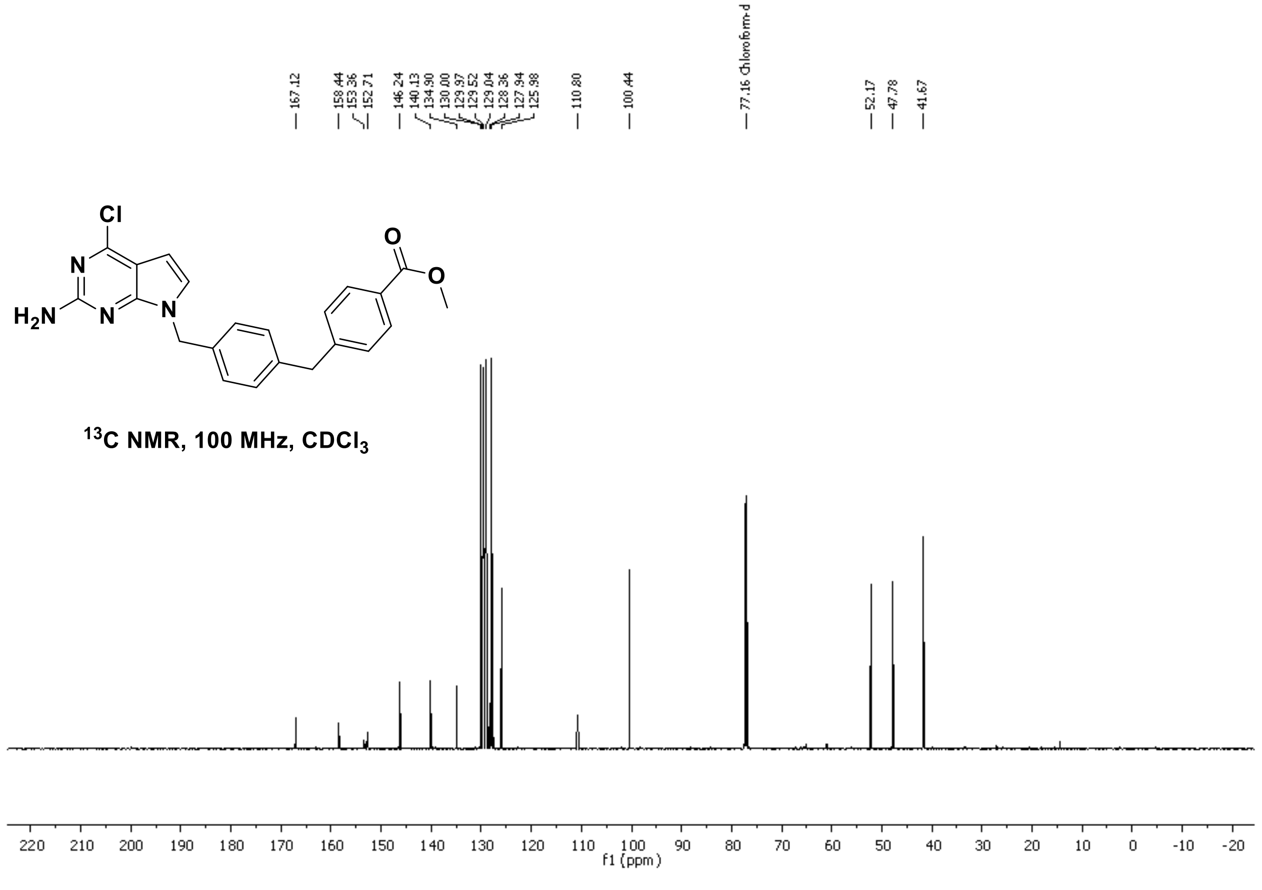

1H NMR and 13C NMR spectra of compound 23.

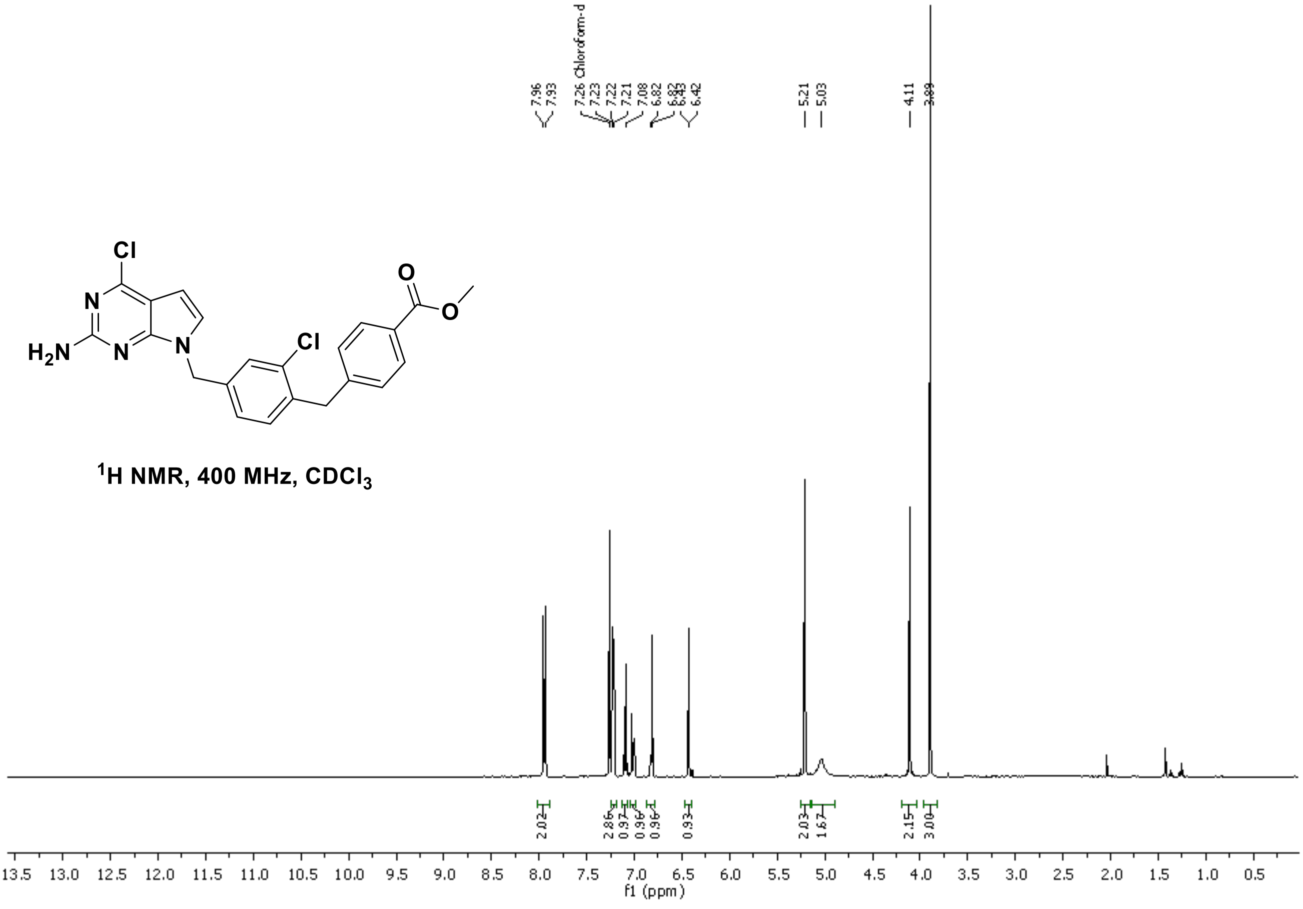

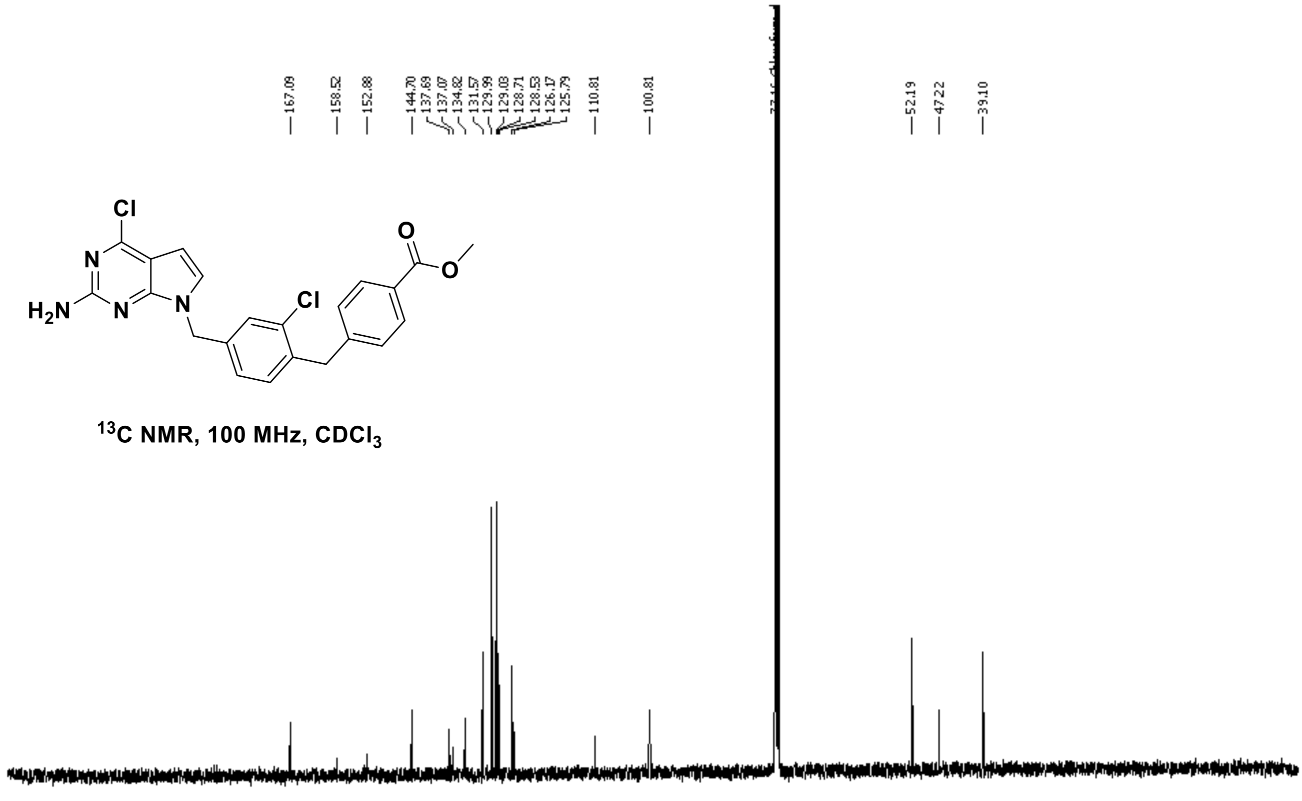

1H NMR and 13C NMR spectra of compound 24.

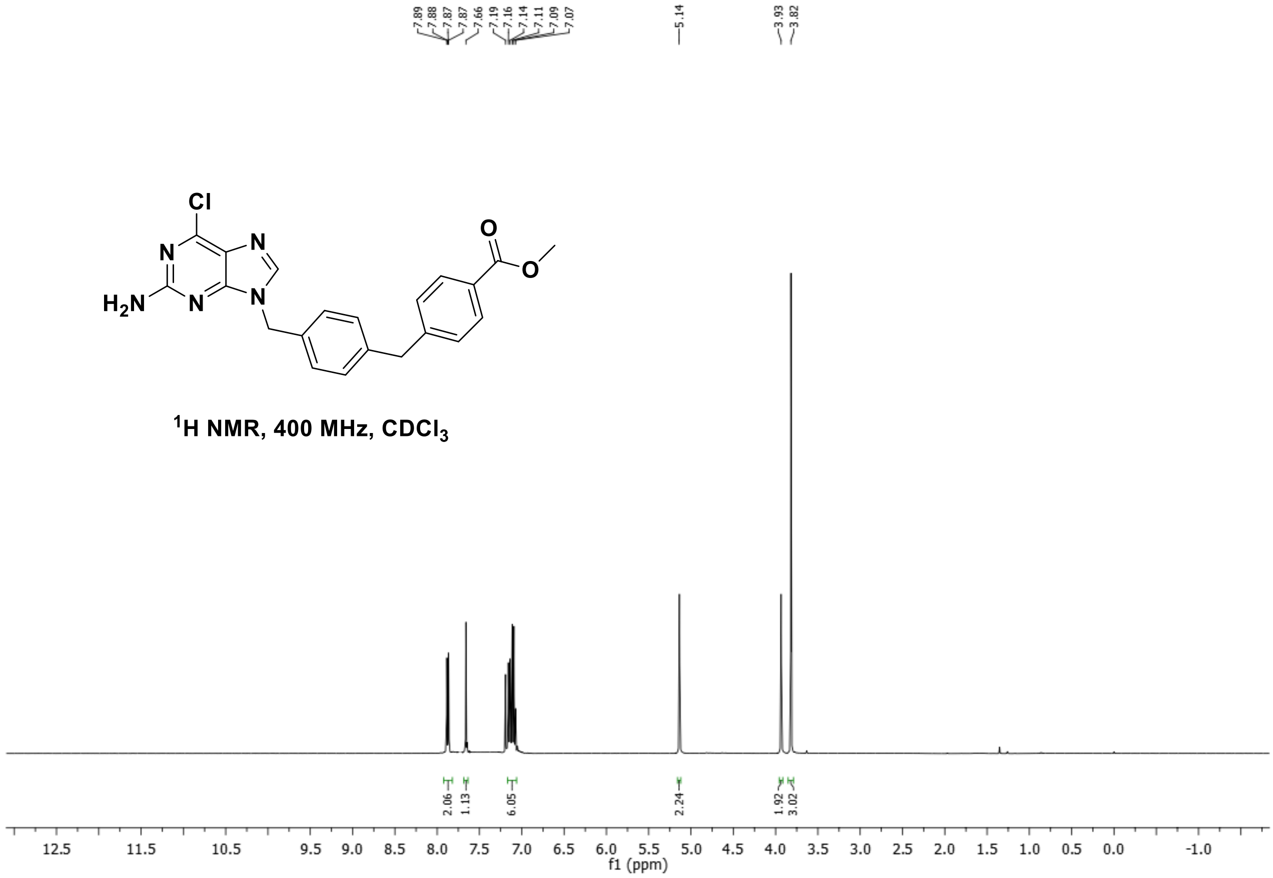

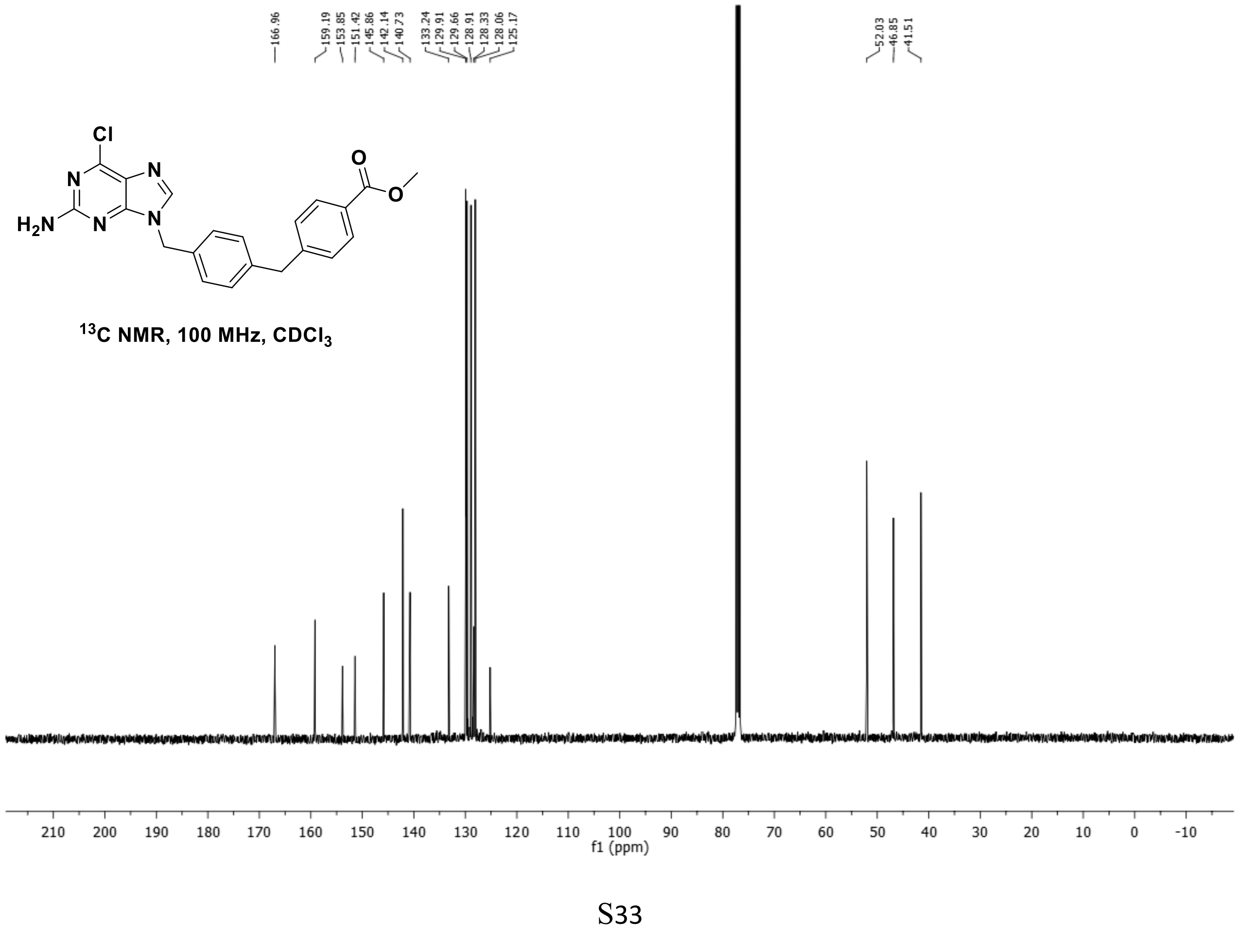

1H NMR spectra of compound 25.

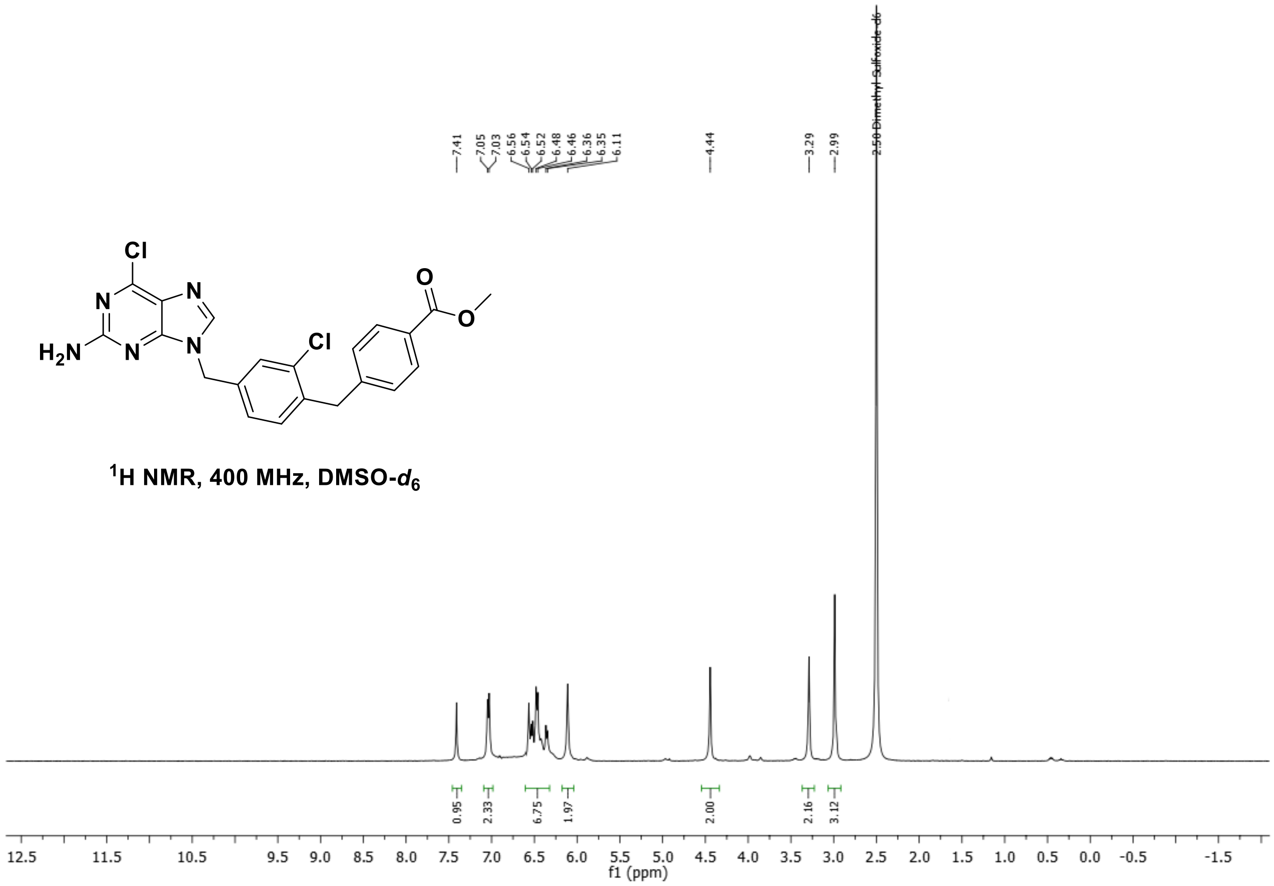

1H NMR and 13C NMR spectra of compound 26.

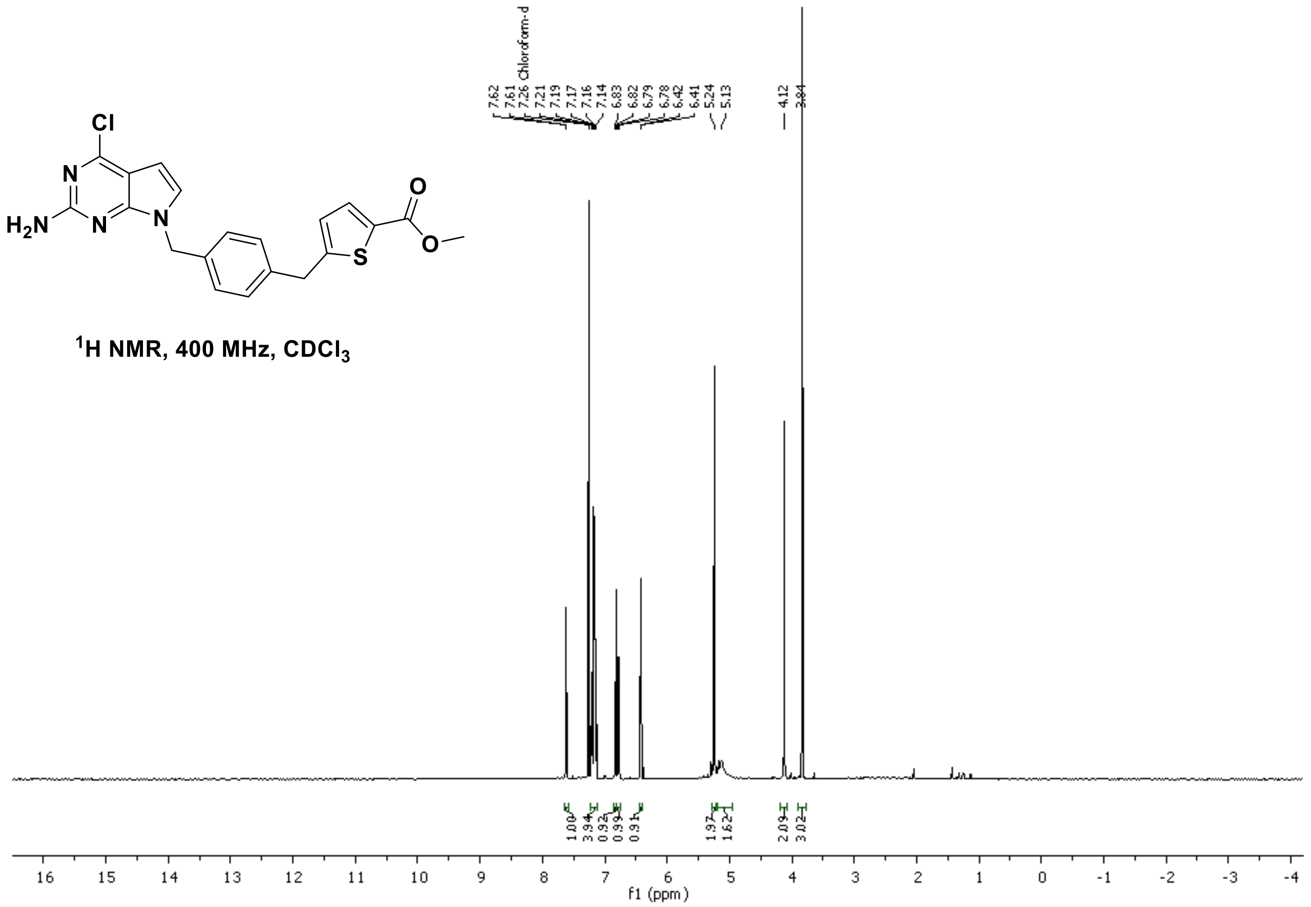

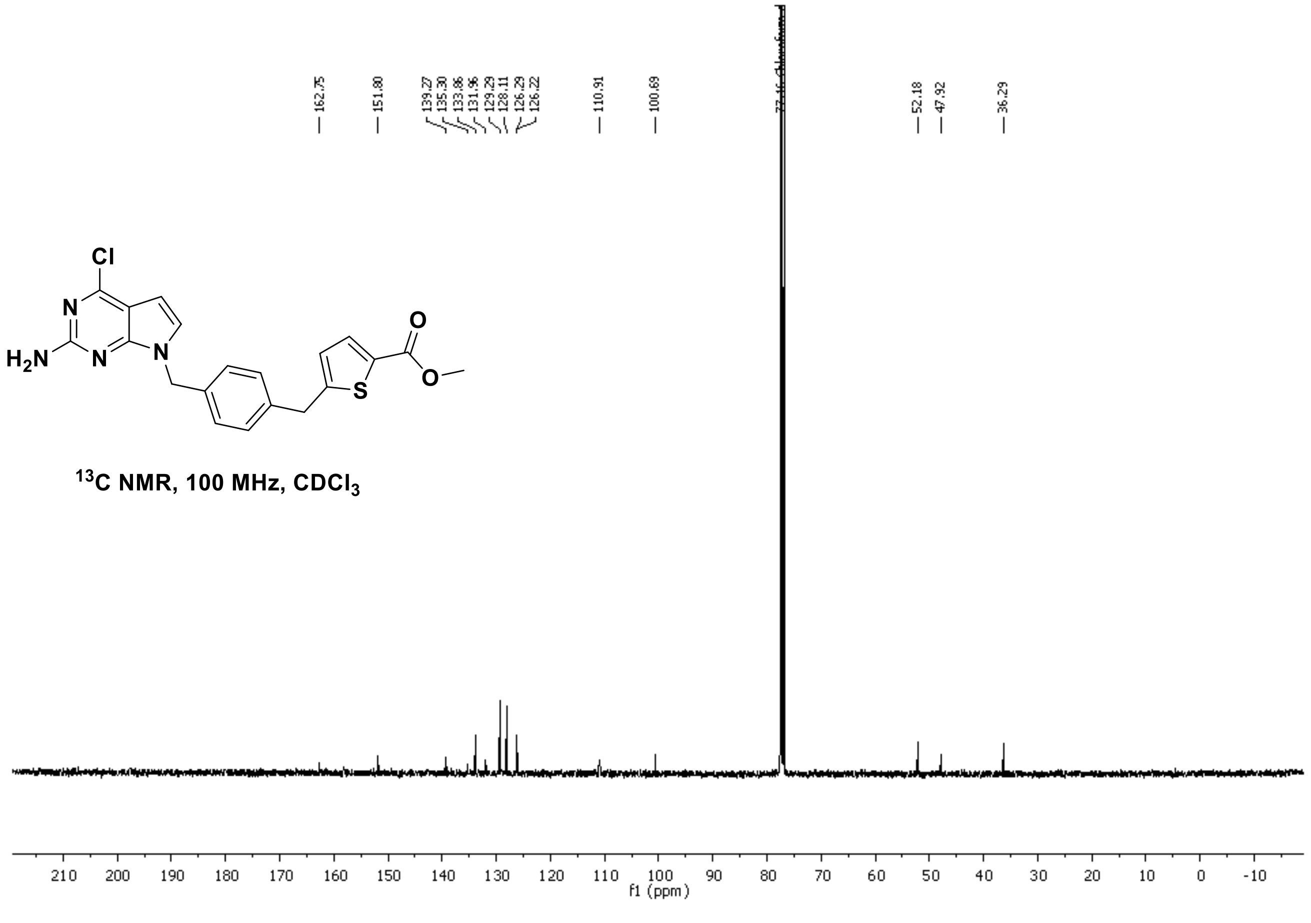

1H NMR and 13C NMR spectra of compound 27.

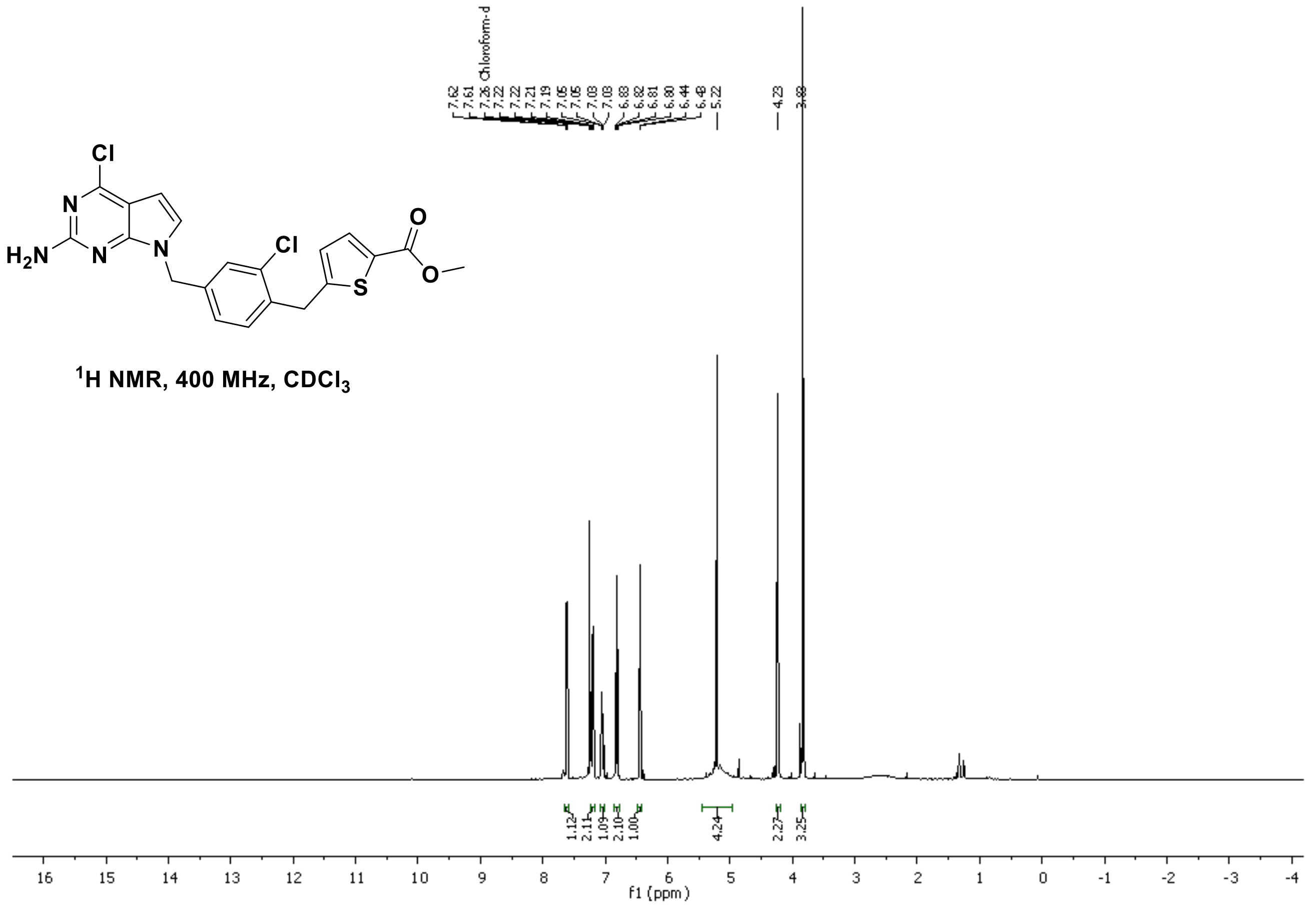

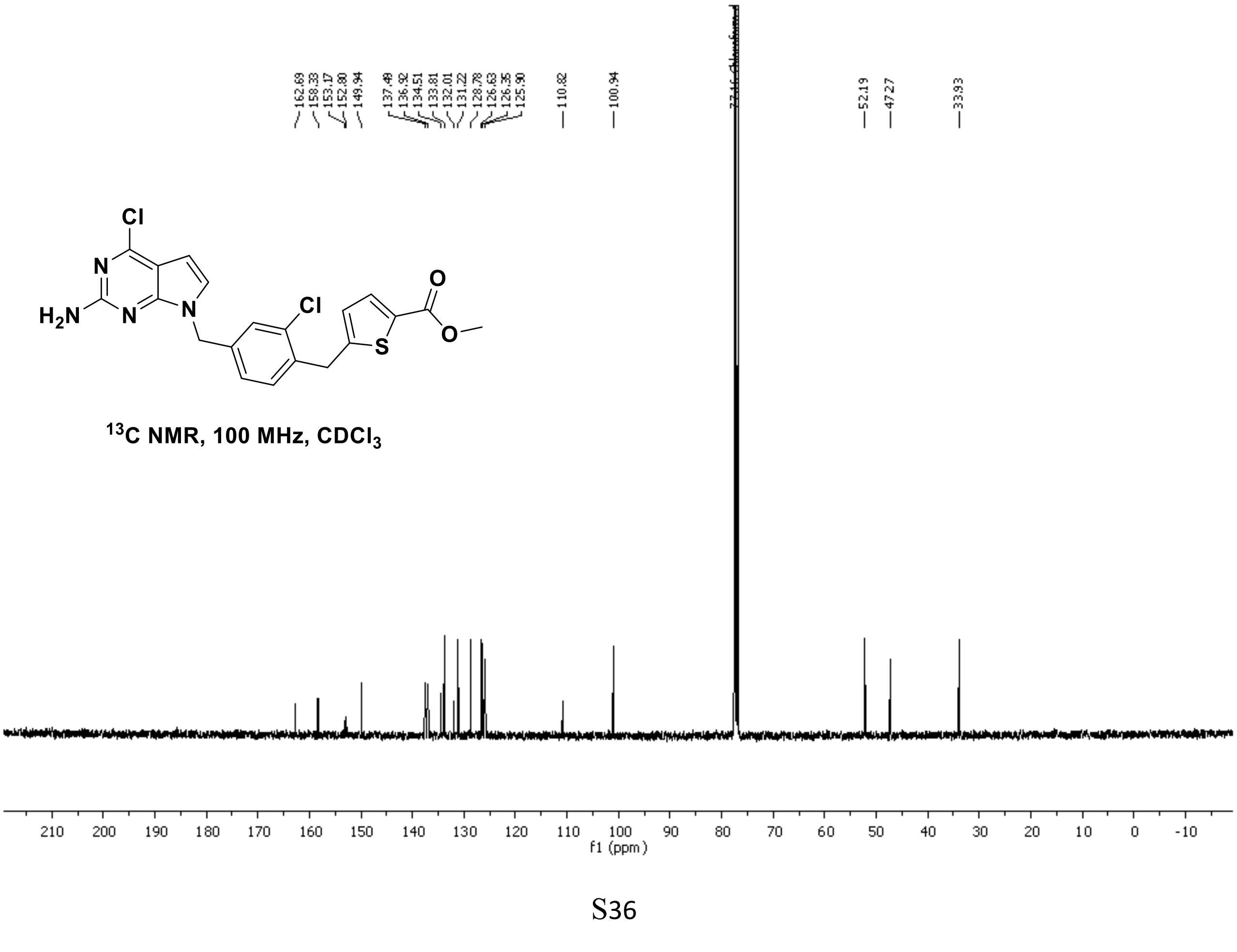

1H NMR and 13C NMR spectra of compound 28.

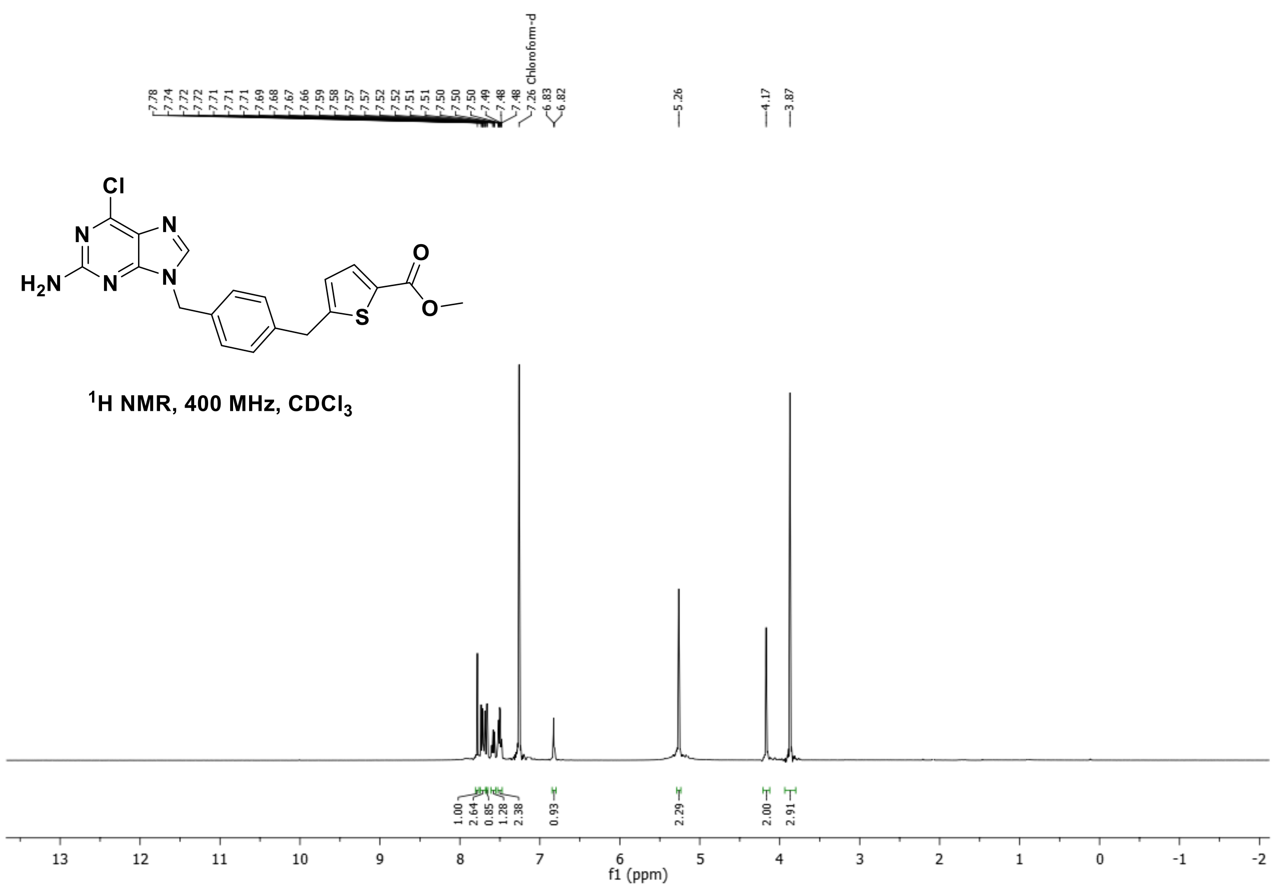

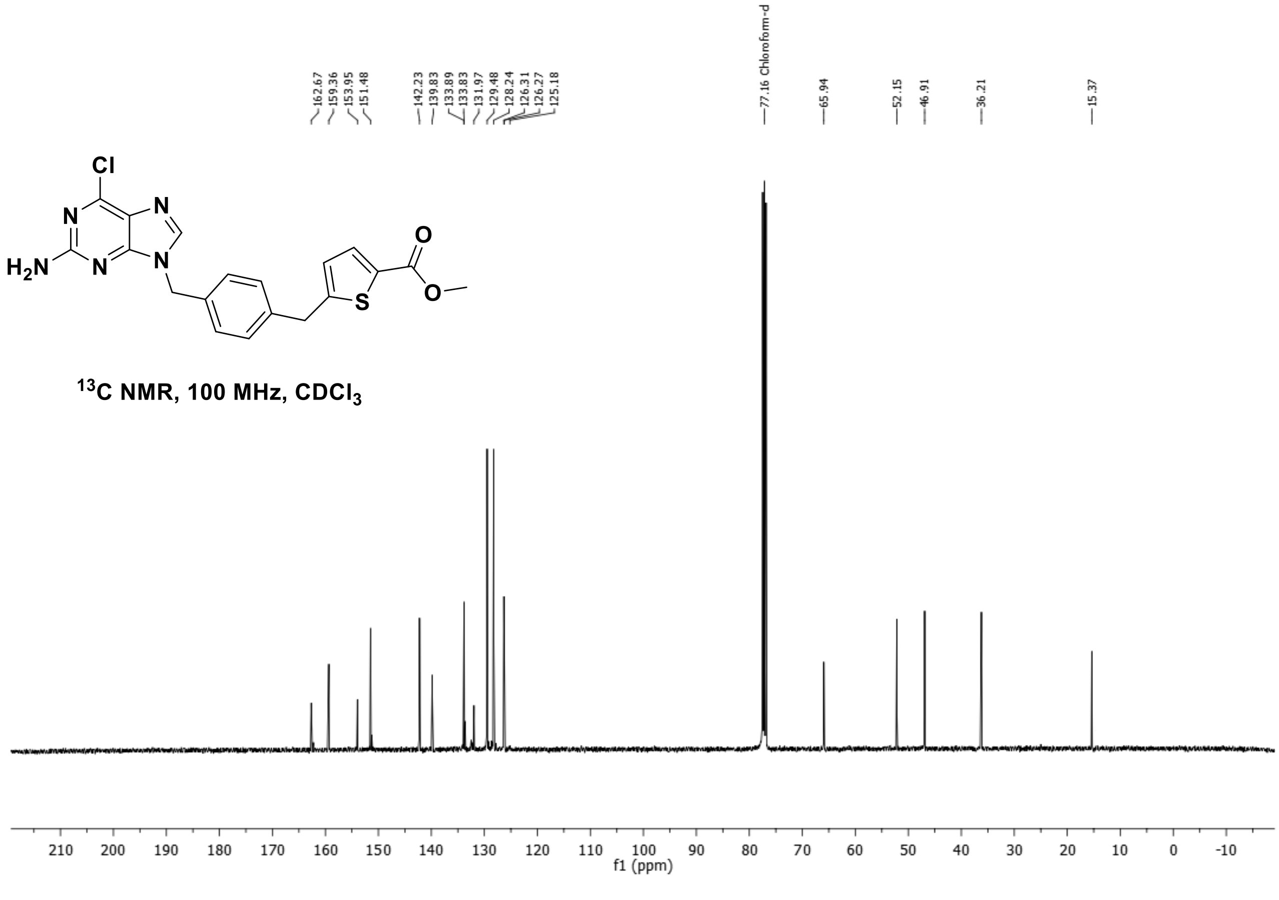

1H NMR and 13C NMR spectra of compound 29.

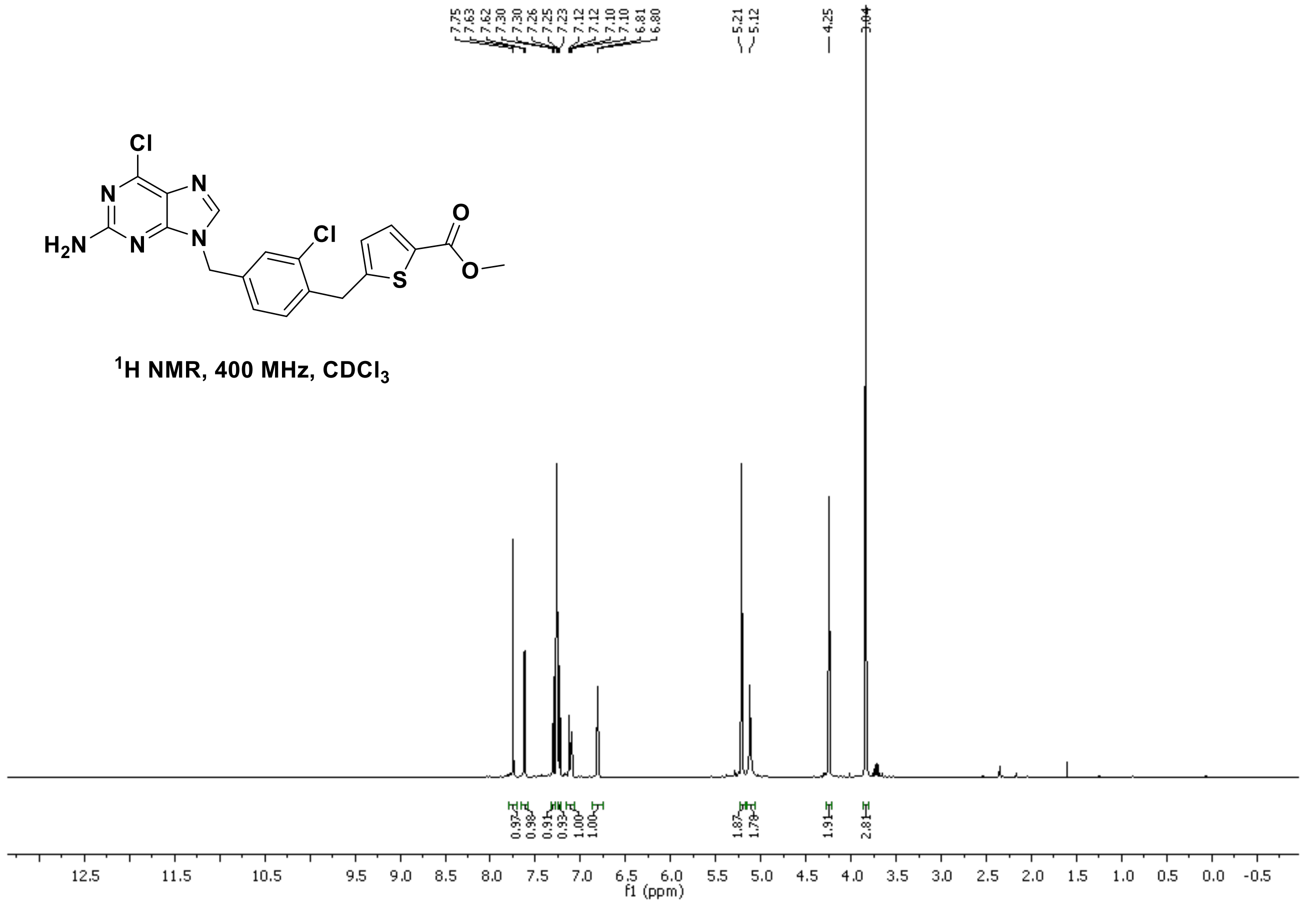

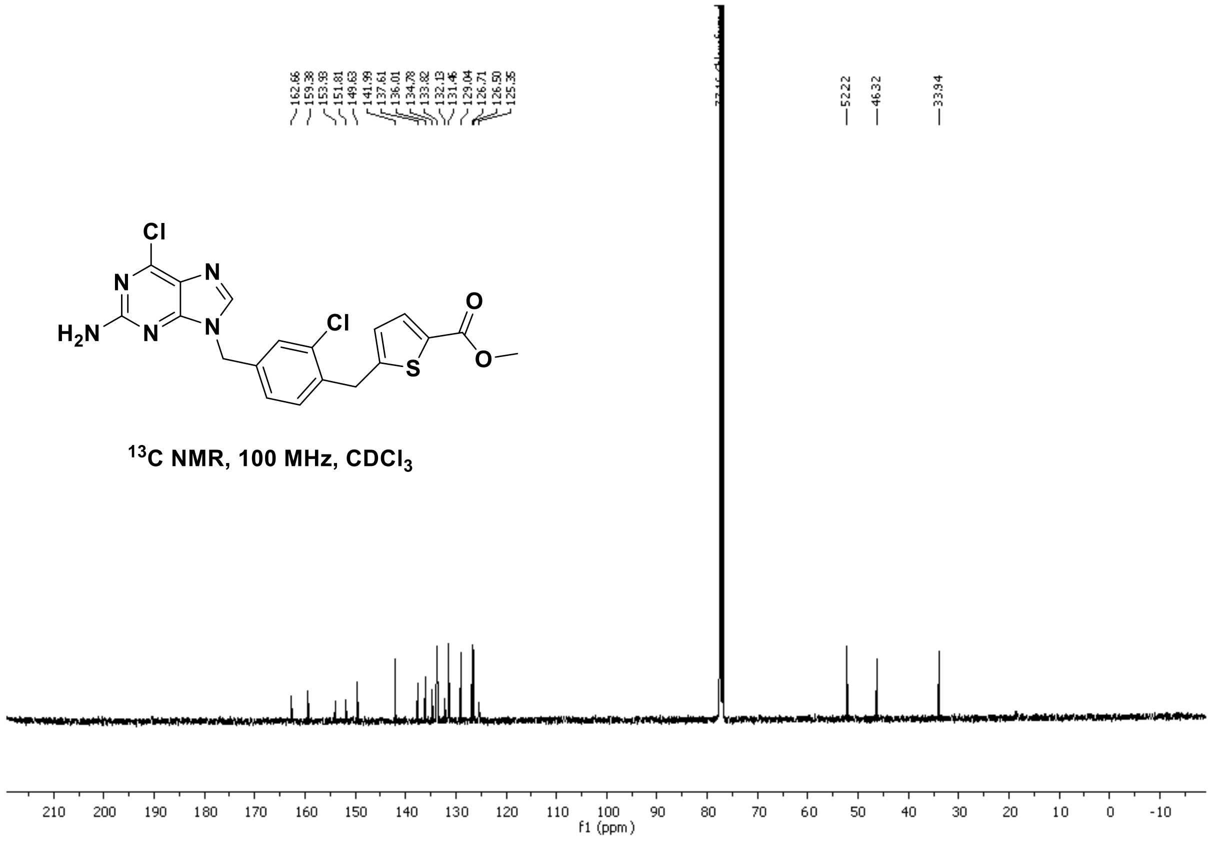

1H NMR and 13C NMR spectra of compound 30.

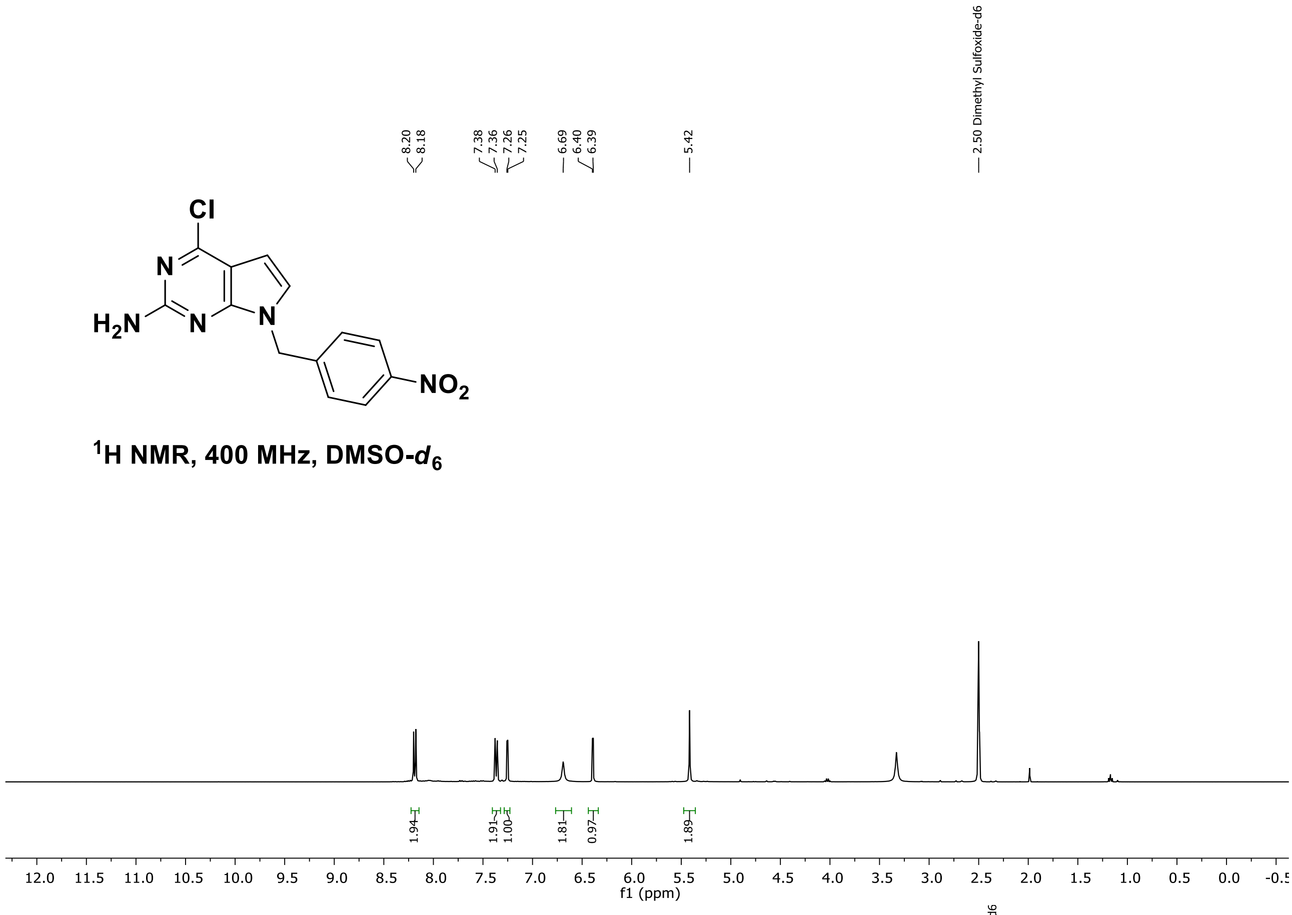

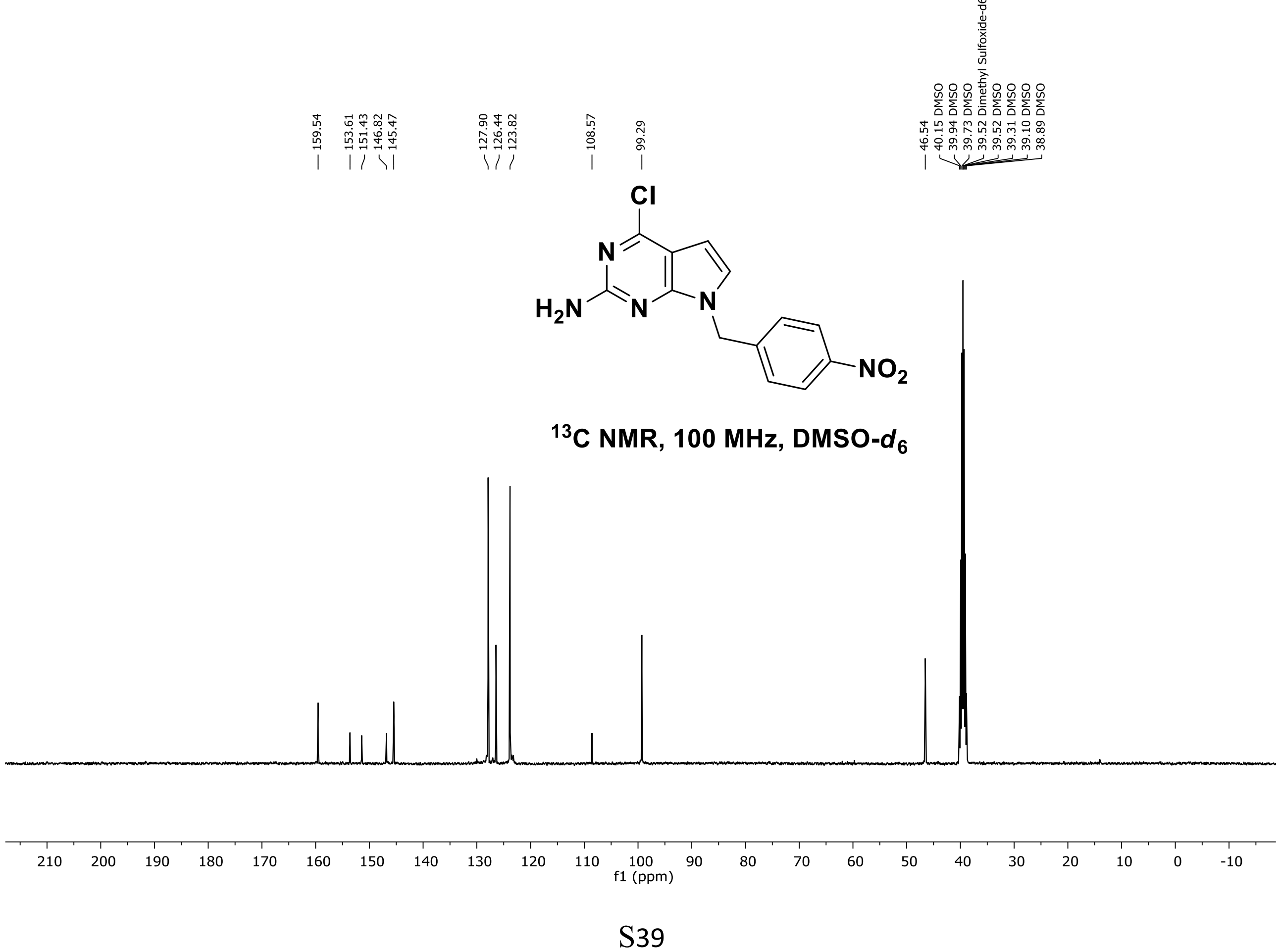

1H NMR and 13C NMR spectra of compound 31.

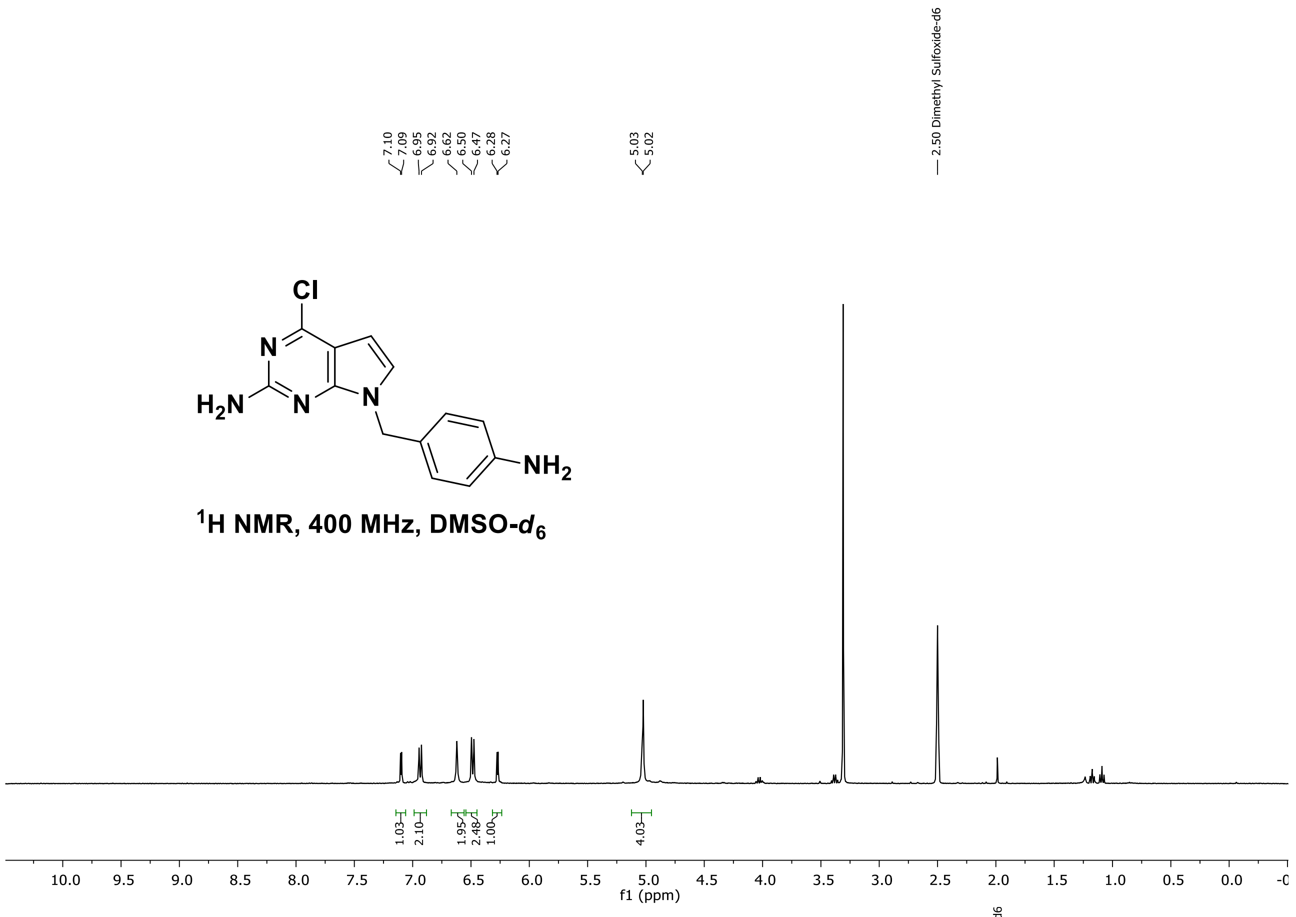

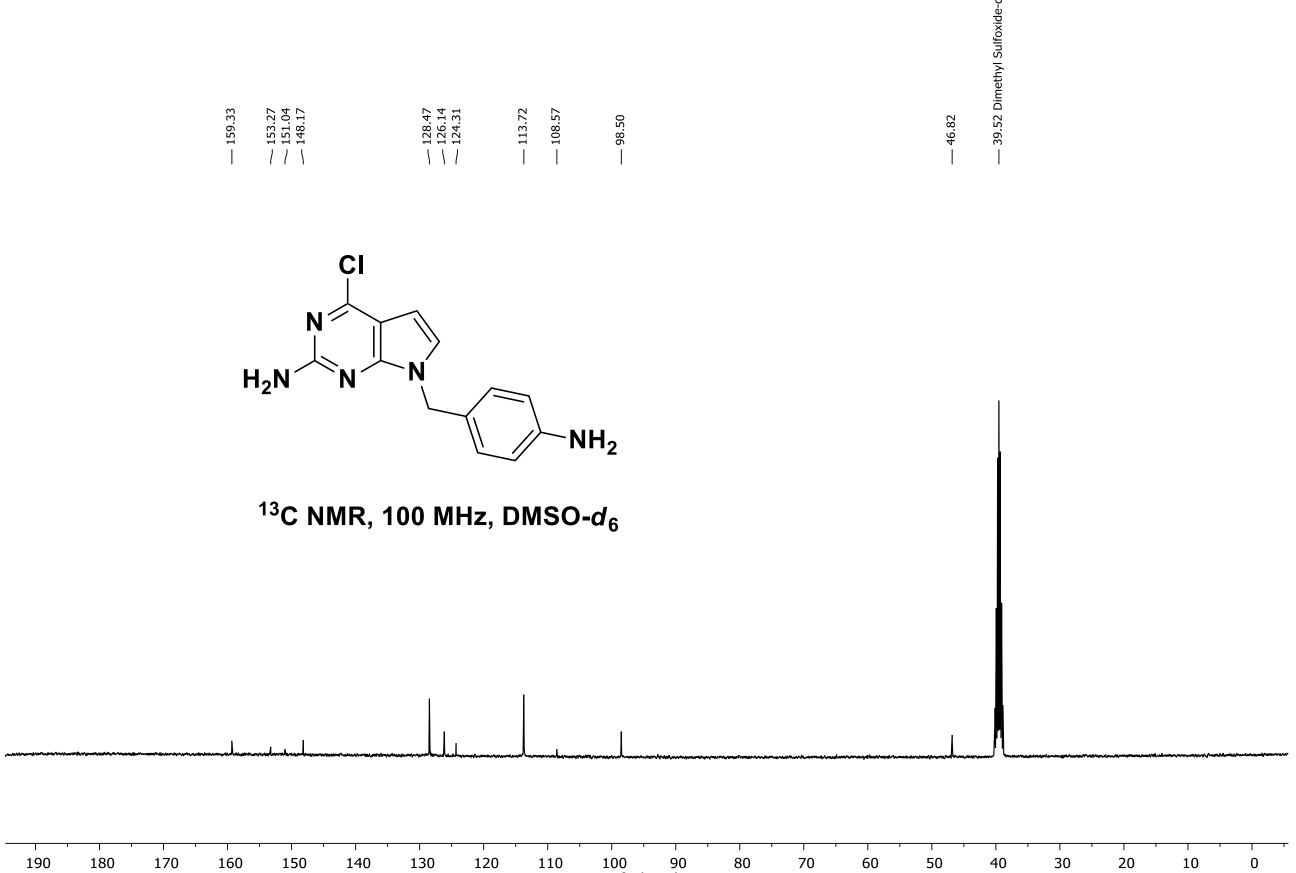

1H NMR spectra of compound 32.

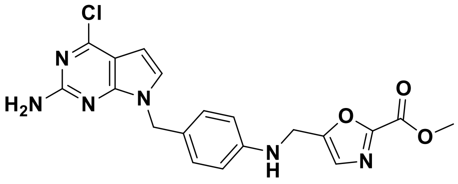

^{1}H NMR, 400 MHz, DMSO-d_6

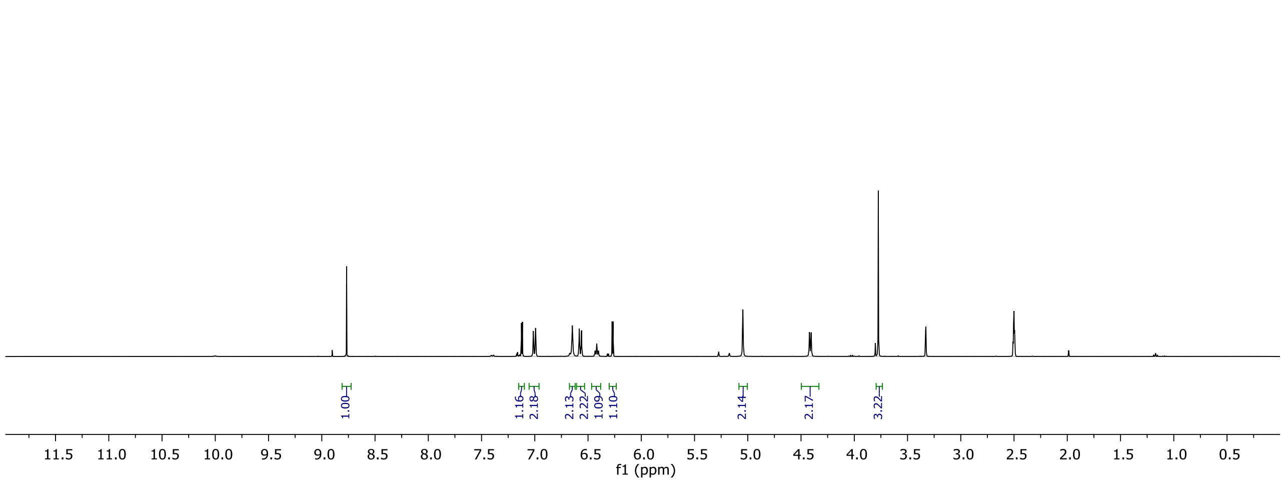

1H NMR spectra of compound 33.

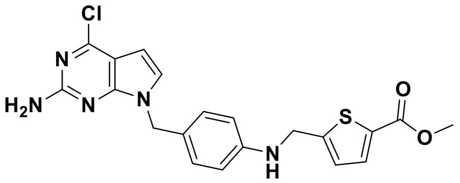

^{1}H NMR, 400 MHz, CD_2Cl_2

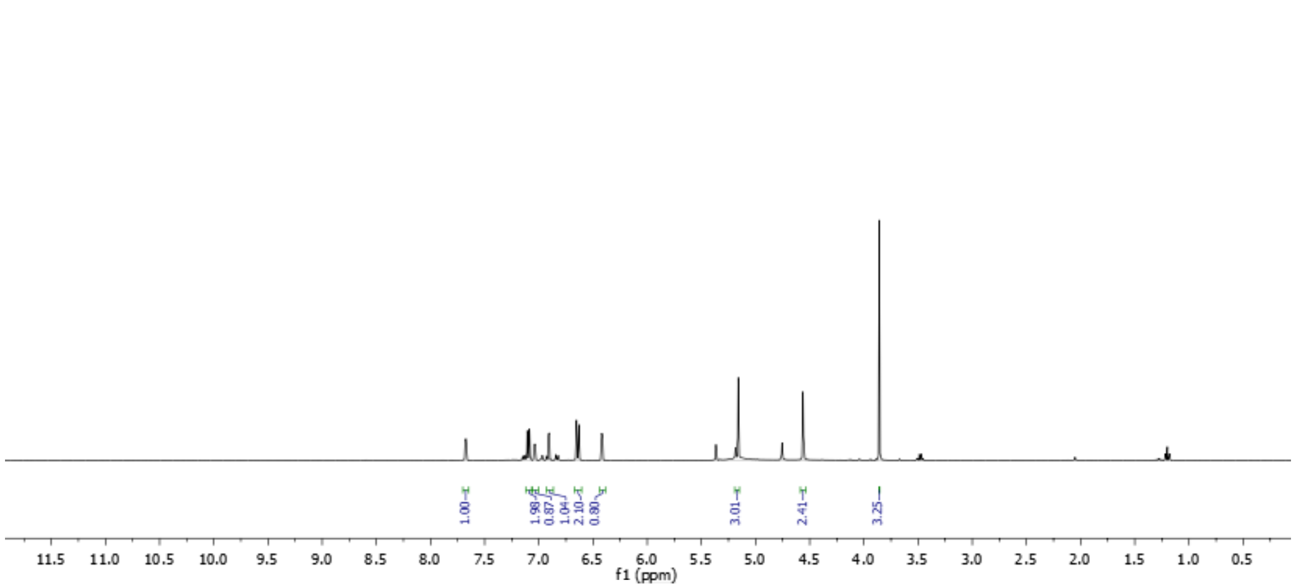

1H NMR and 13C NMR spectra of compound 34.

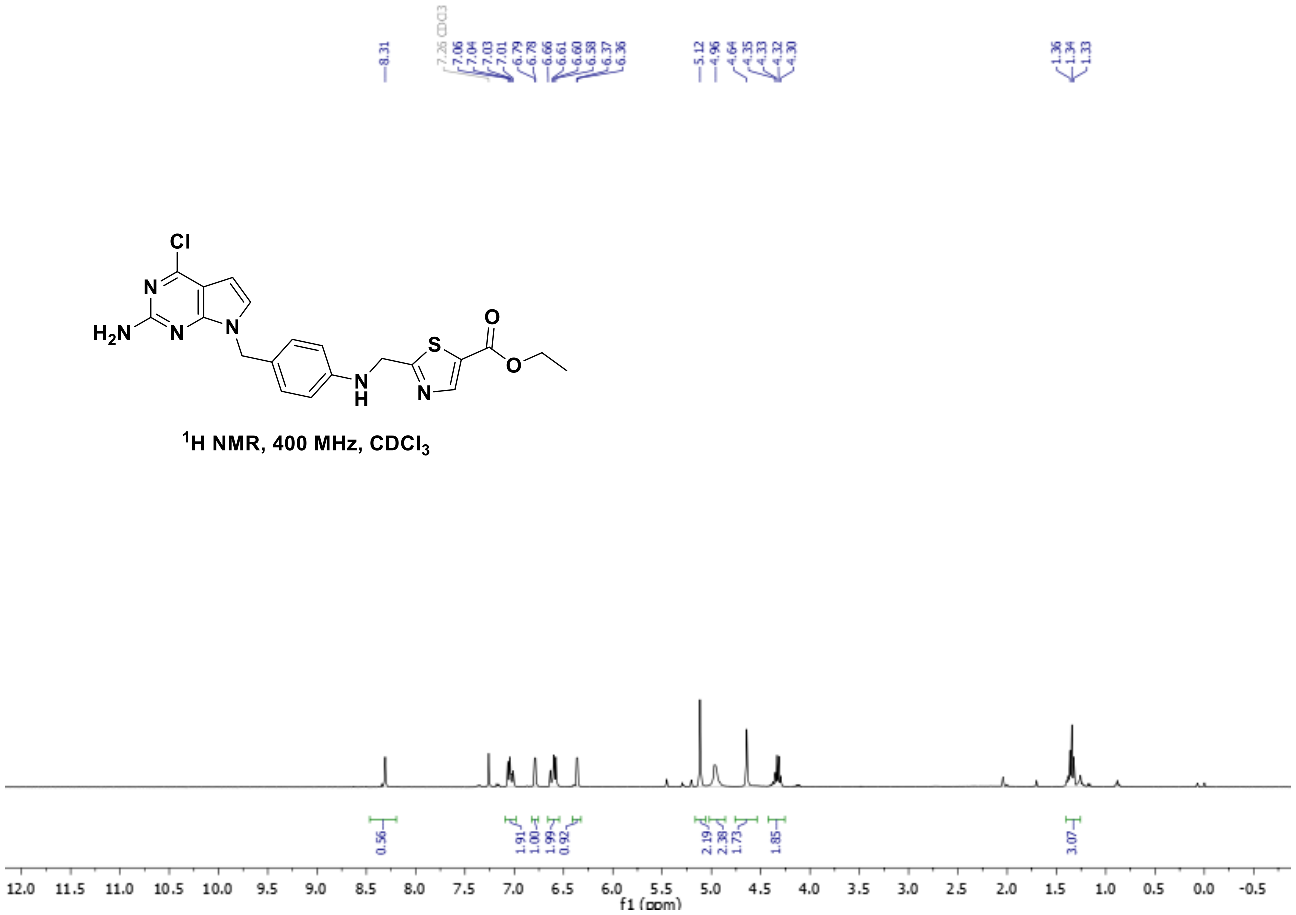

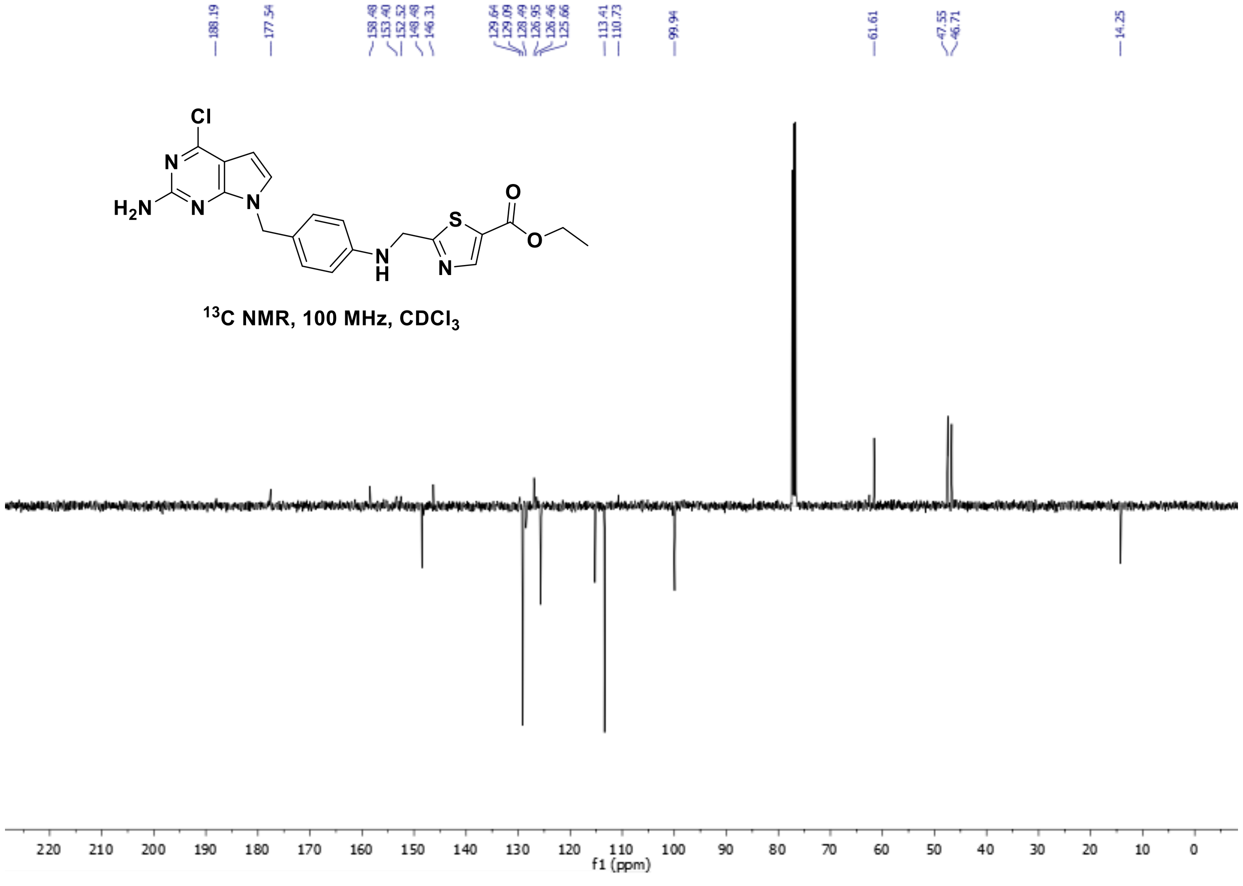

1H NMR spectra of compound 35.

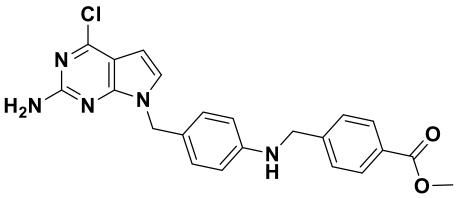

^{1}H NMR, 400 MHz, $CDCl_3$

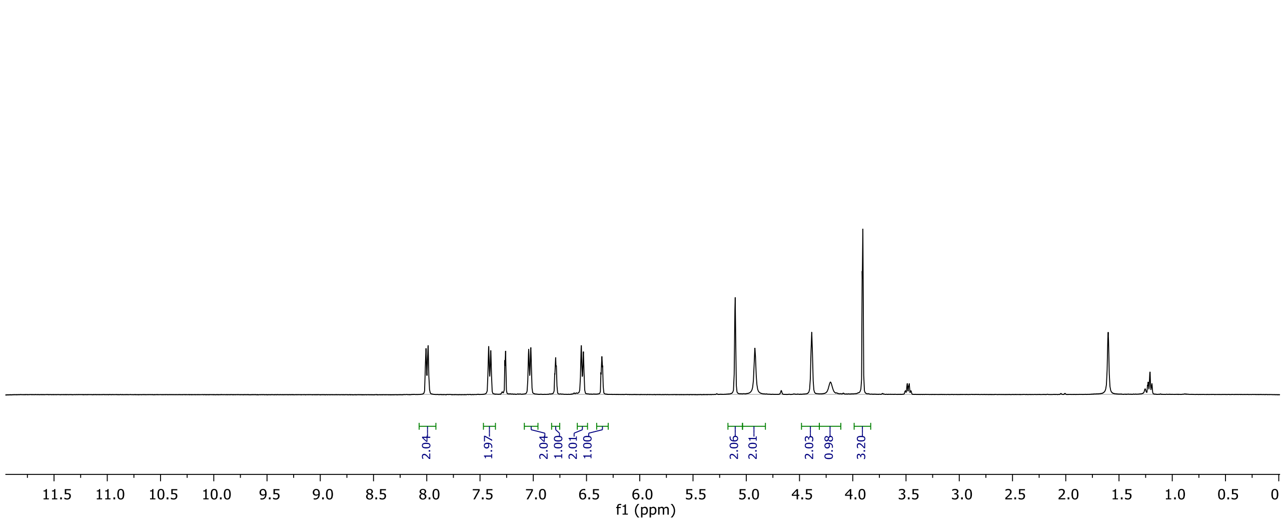

1H NMR spectra of compound 36.

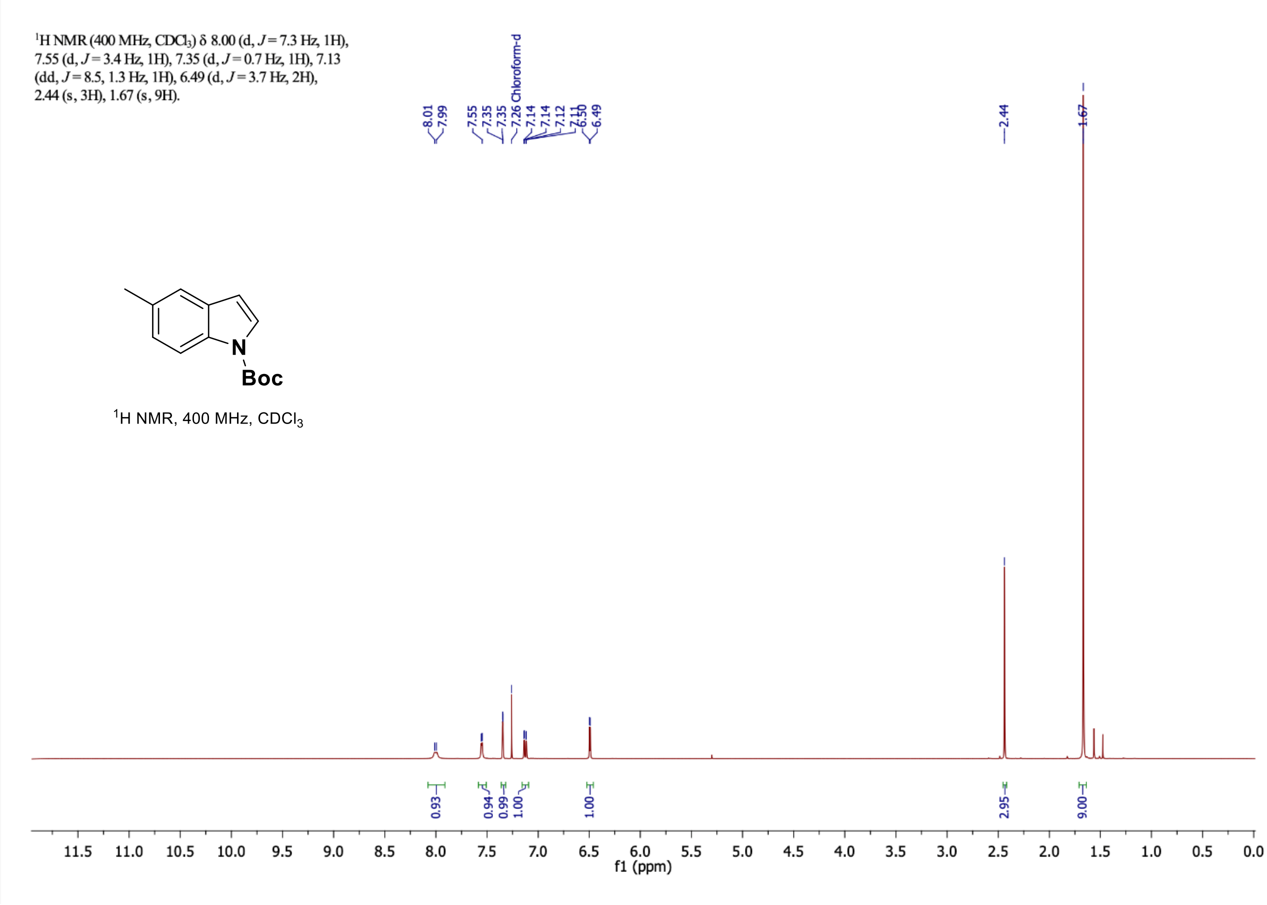

1H NMR spectra of compound 37.

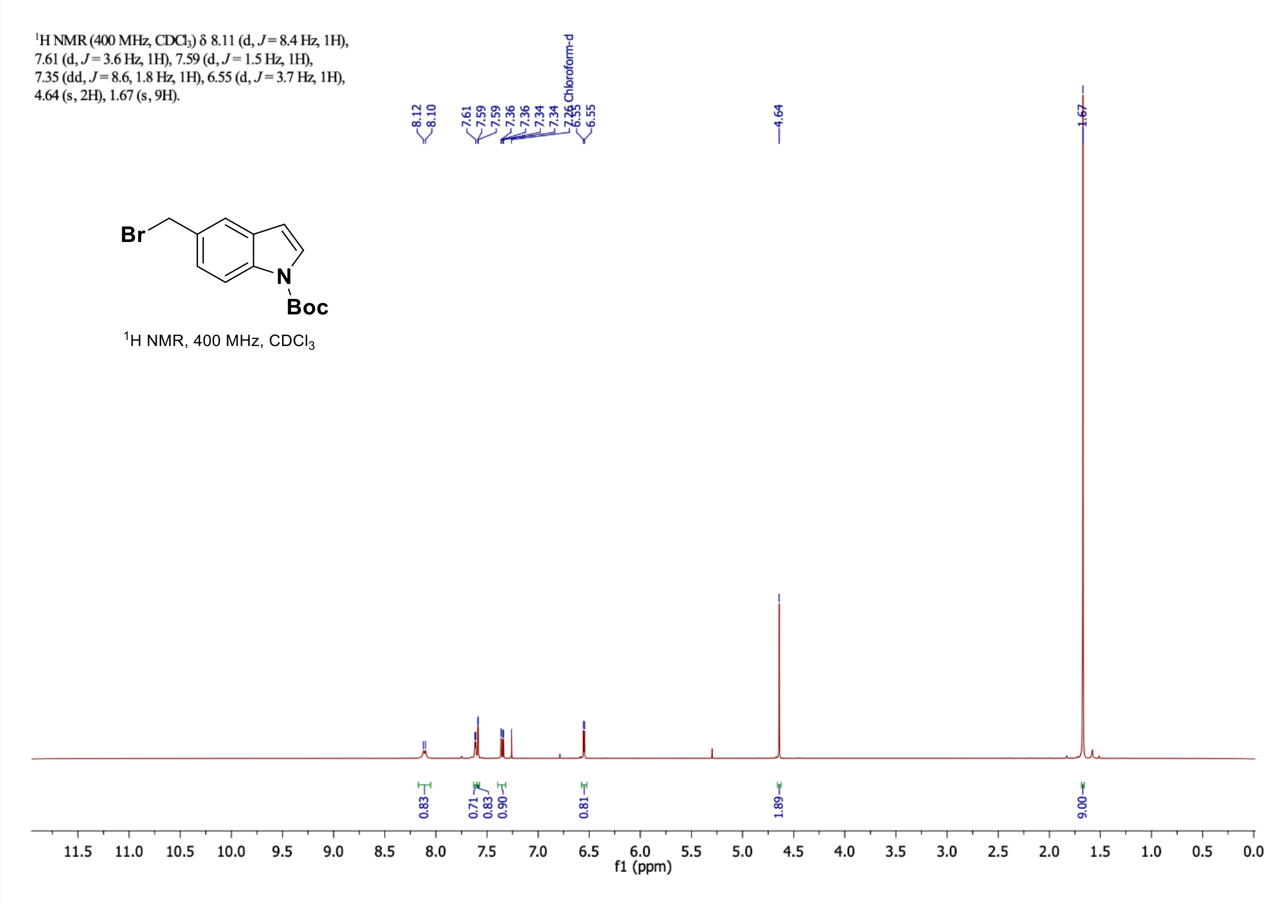

1H NMR spectra of compound 38.

^{1}H NMR (400 MHz, $CDCl_3$) δ 8.08 (d, J = 8.3 Hz, 1H), 7.59 (d, J = 3.5 Hz, 1H), 7.37 (s, 1H), 7.17 (d, J = 8.6 Hz, 1H), 6.81 (d, J = 3.7 Hz, 1H), 6.51 (d, J = 3.7 Hz, 1H), 6.38 (d, J = 3.7 Hz, 1H), 5.32 (s, 2H), 1.65 (s, 9H).

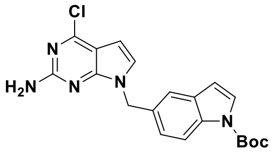

^{1}H NMR, 400 MHz, $CDCl_3$

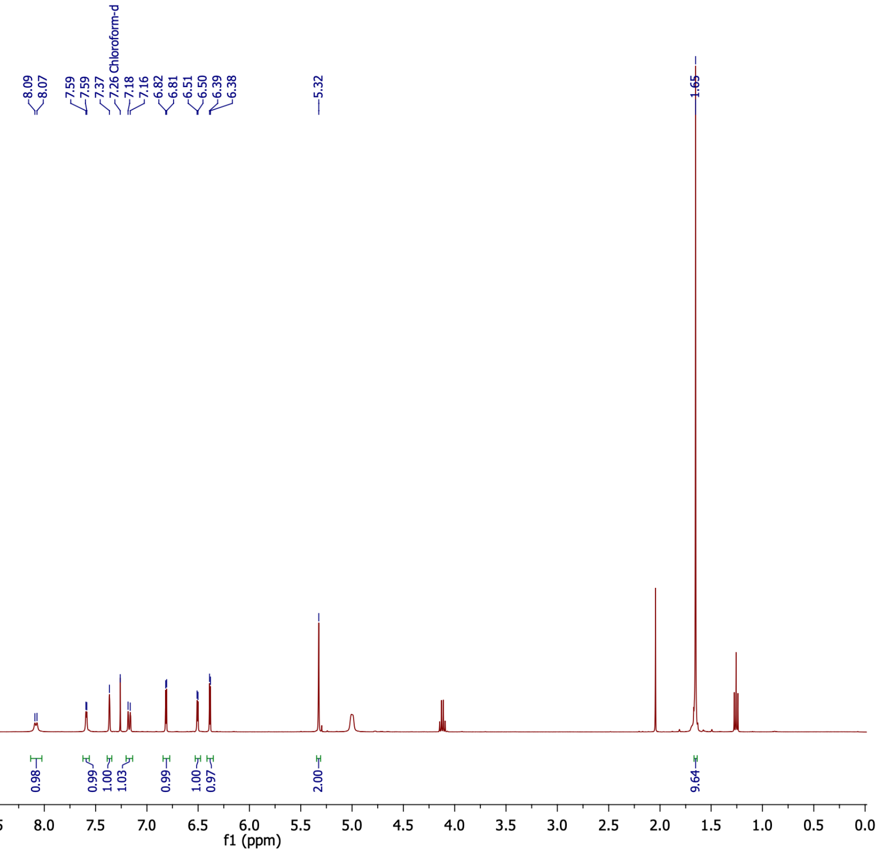

LCMS of compound 38.

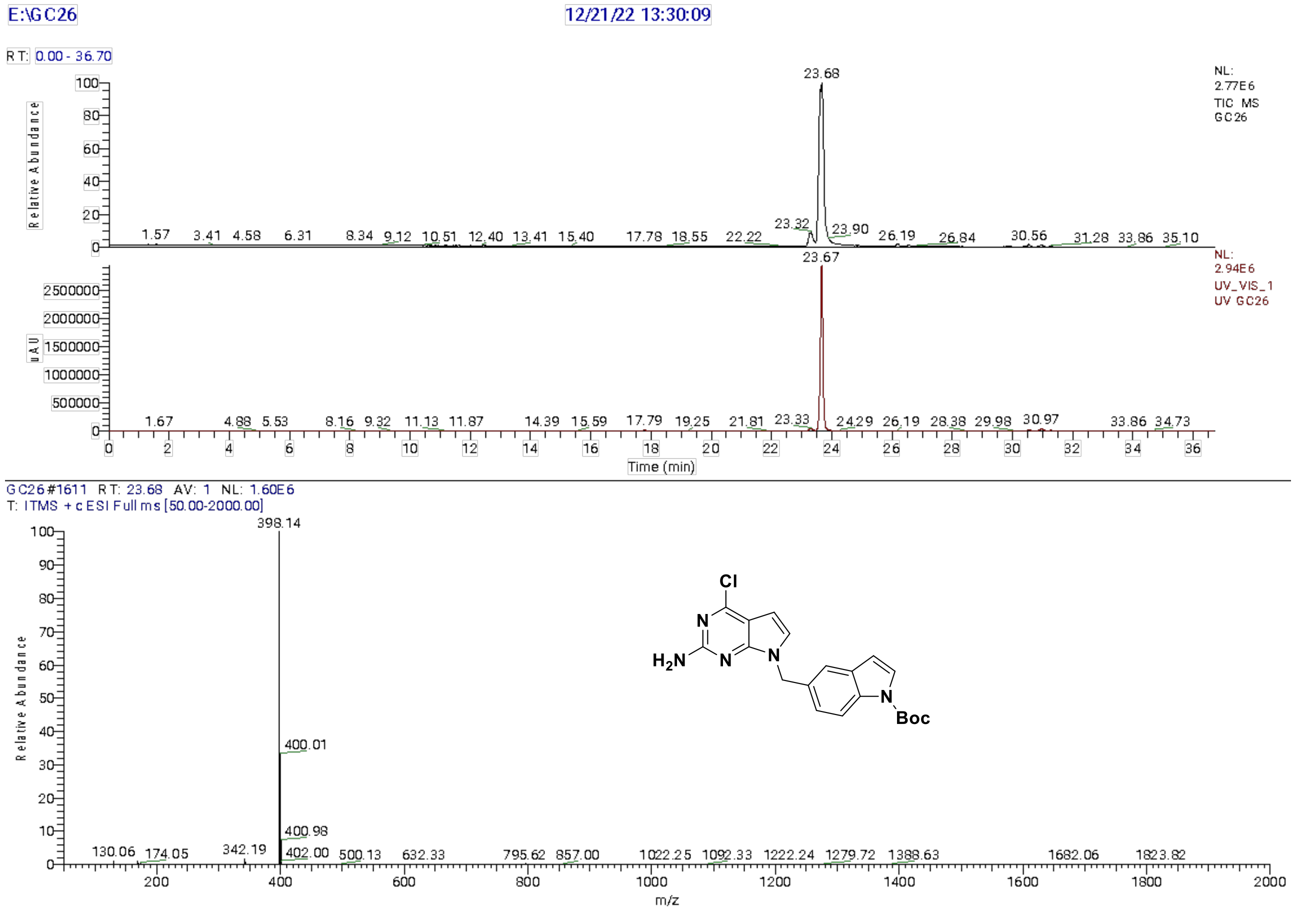

1H NMR spectra of compound 39.

^{1}H NMR (400 MHz, DMSO) δ 11.09 (s, 1H), 7.39 (s, 1H), 7.33 (d, J = 8.7 Hz, 1H), 7.33 (d, J = 3.1 Hz, 1H), 7.17 (d, J = 3.6 Hz, 1H), 7.01 (dd, J = 8.4, 1.3 Hz, 1H), 6.68 (s, 2H), 6.37 (s, 1H), 6.30 (d, J = 3.6 Hz, 1H), 5.28 (s, 2H).

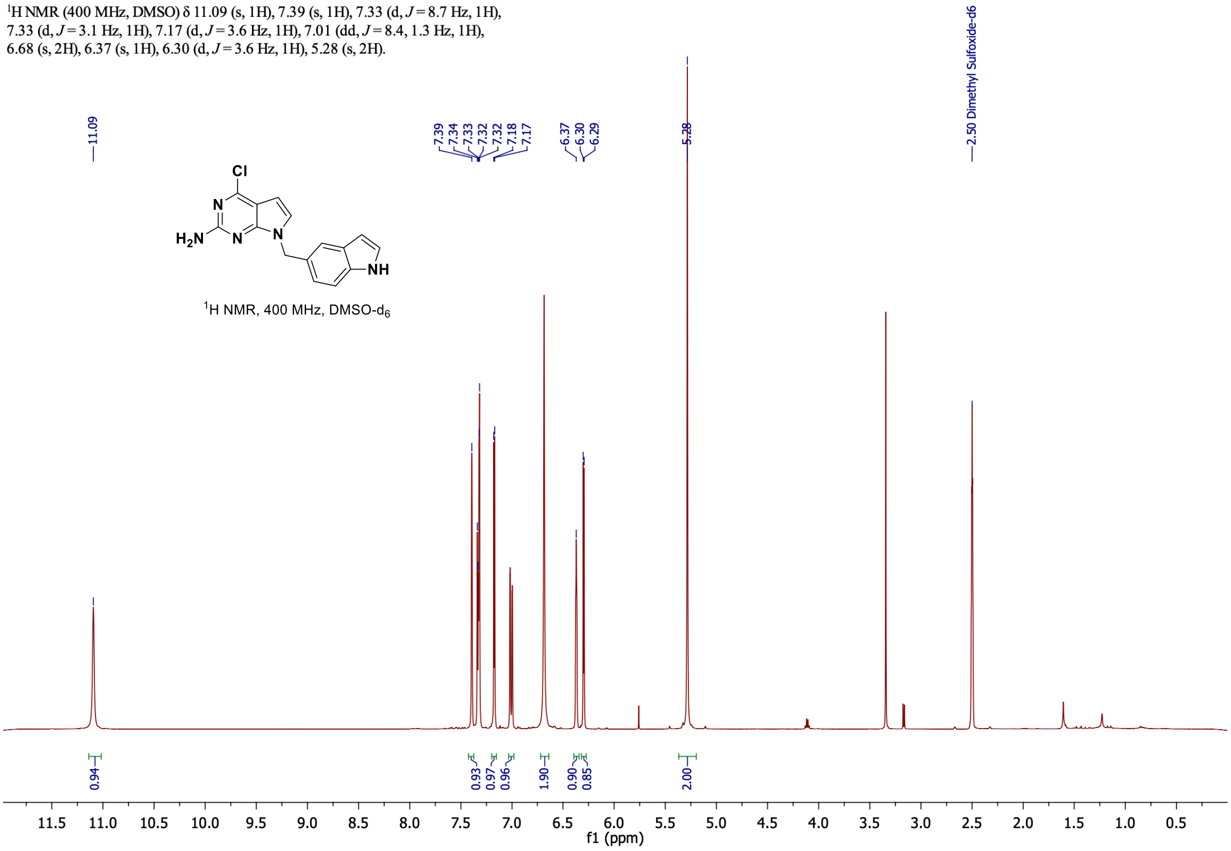

LCMS of compound 39.

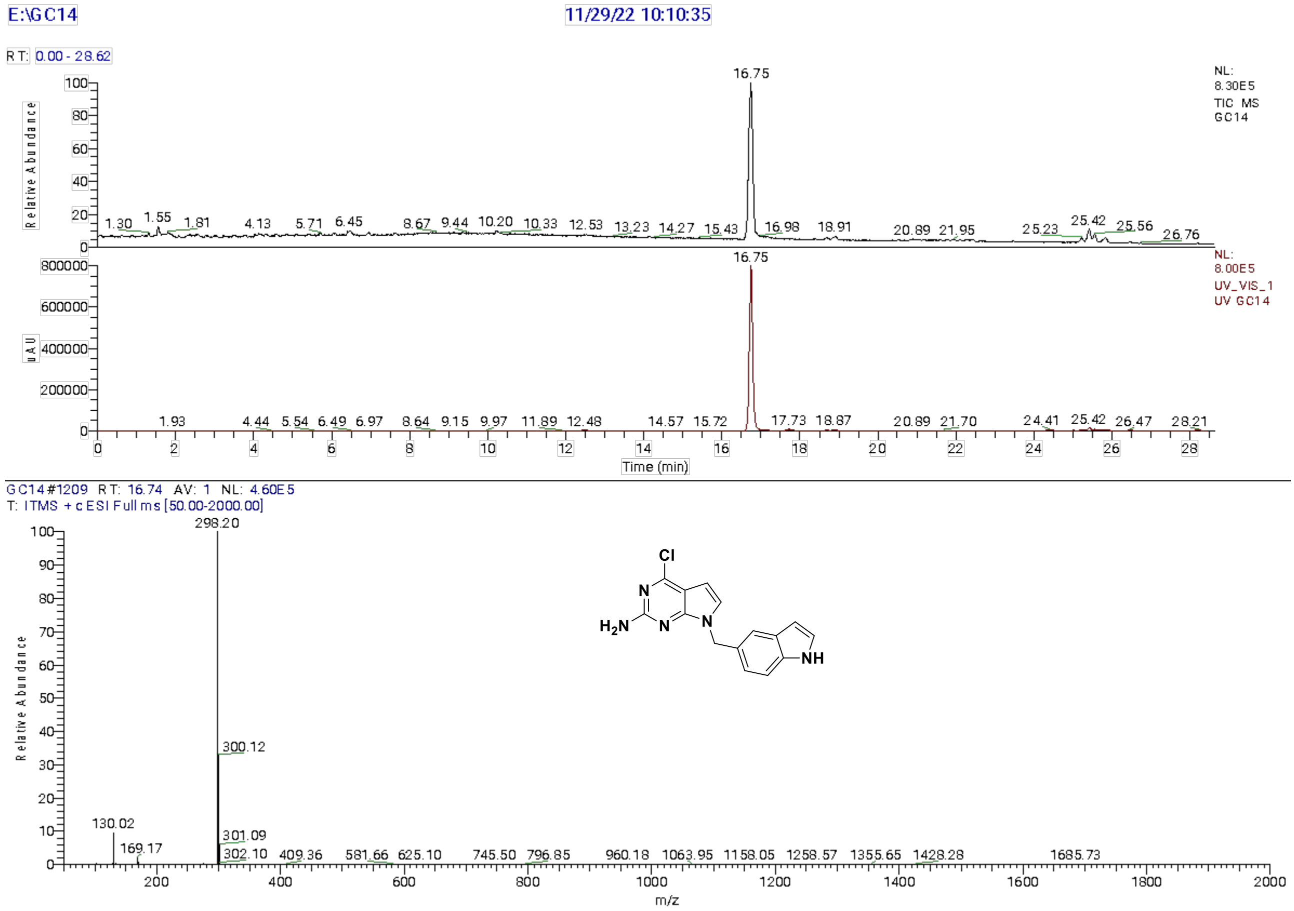

1H NMR spectra of compound 40.

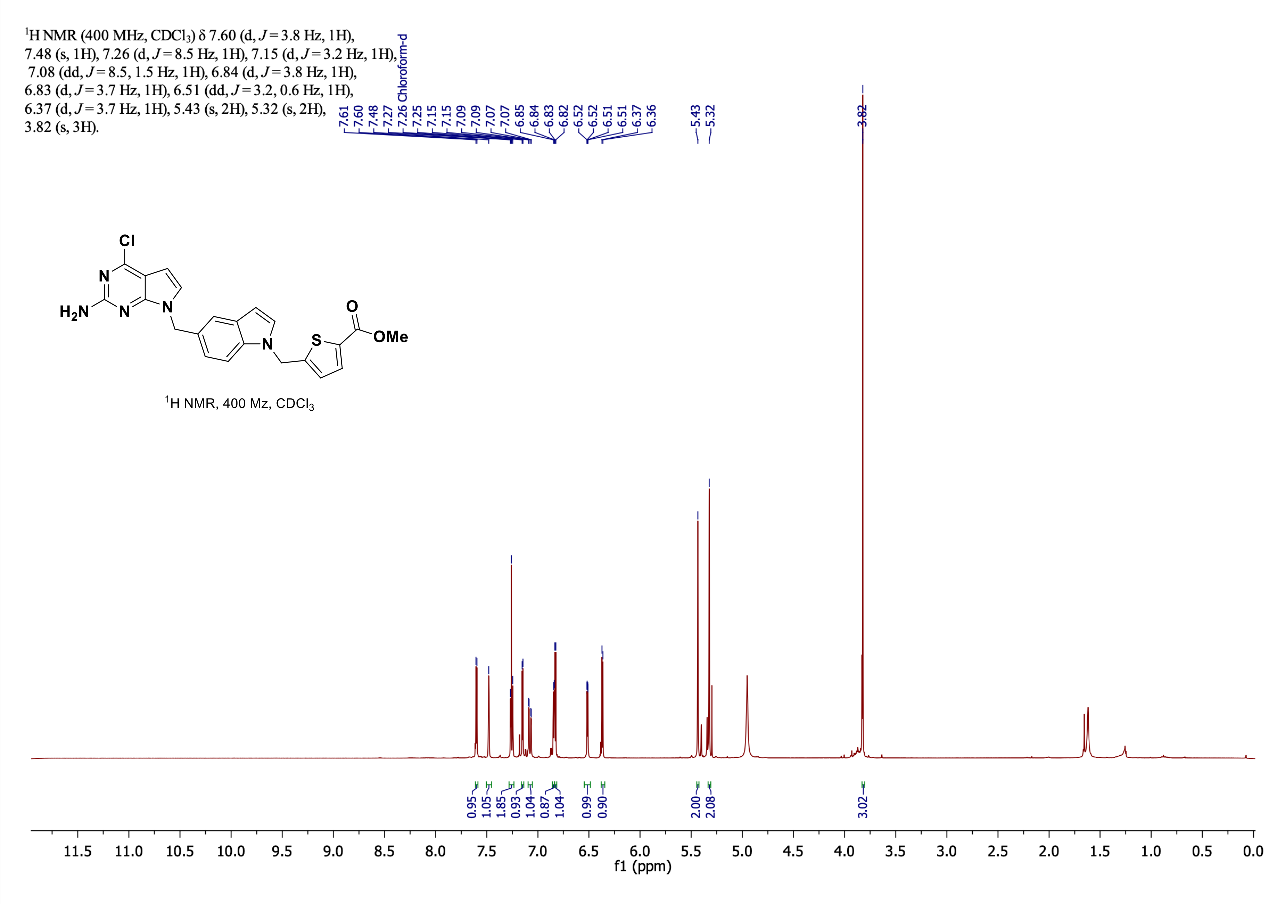

LCMS of compound 40.

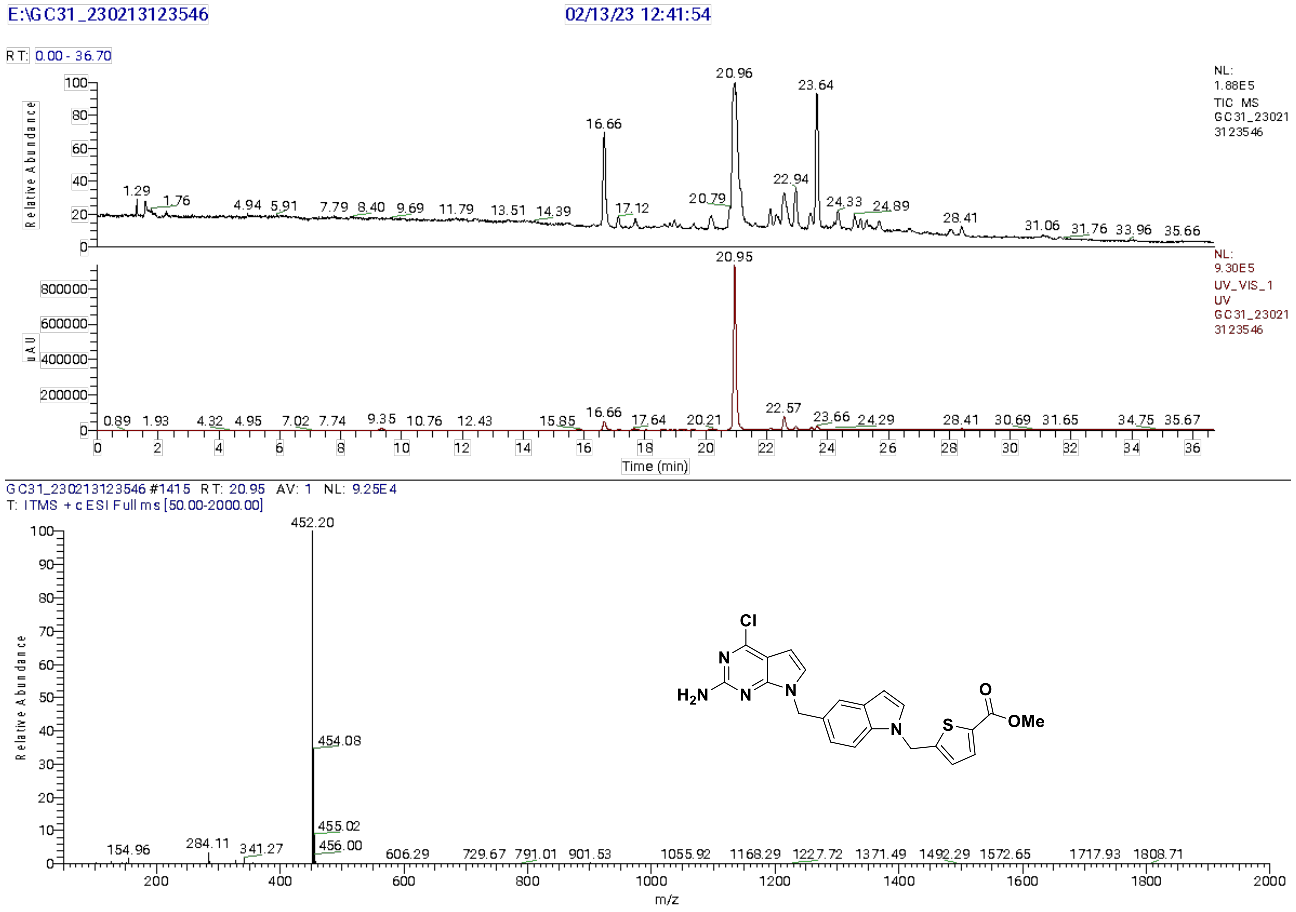

1H NMR spectra of compound 41

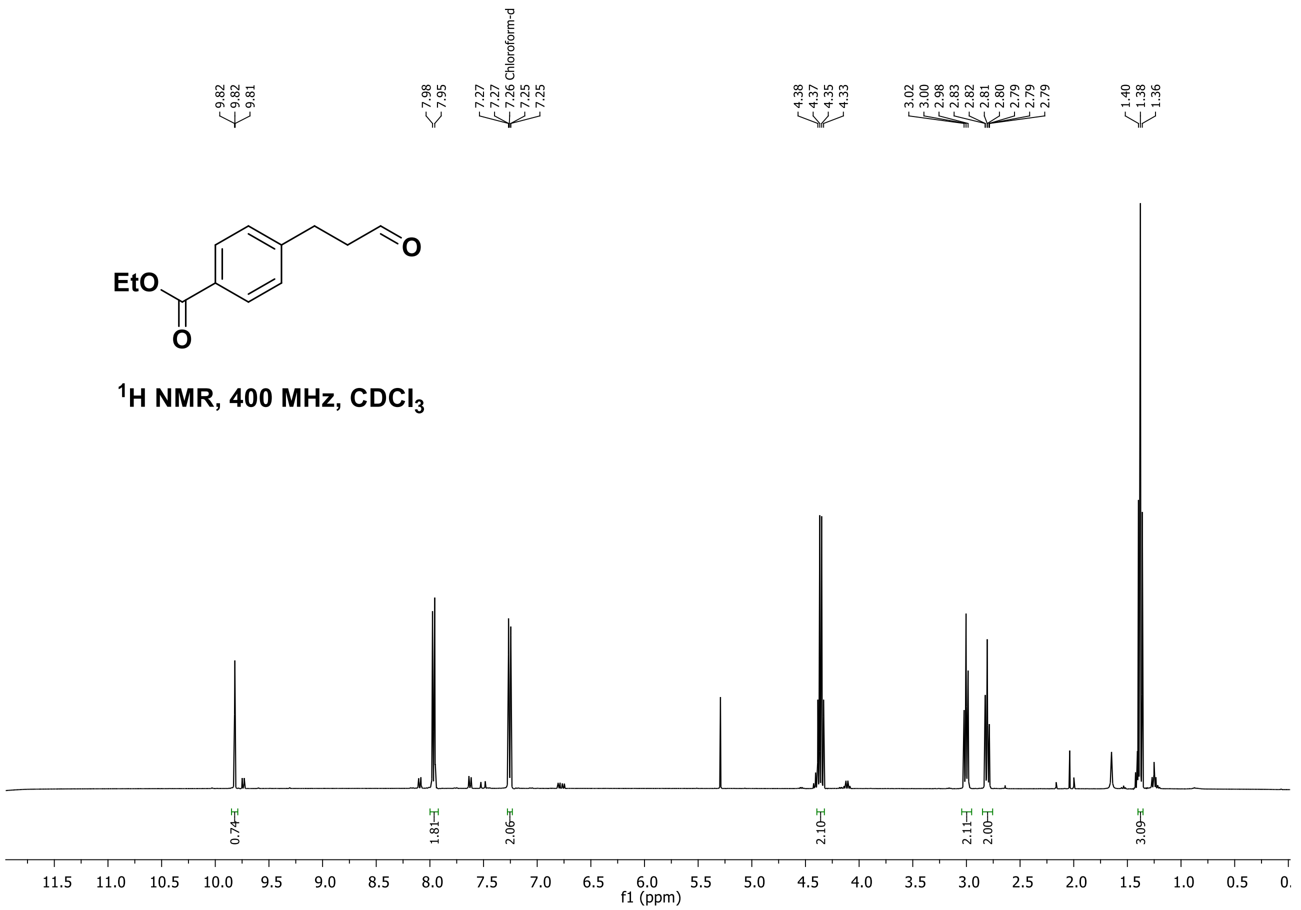

1H NMR and 13C NMR spectra (APT) of compound 42.

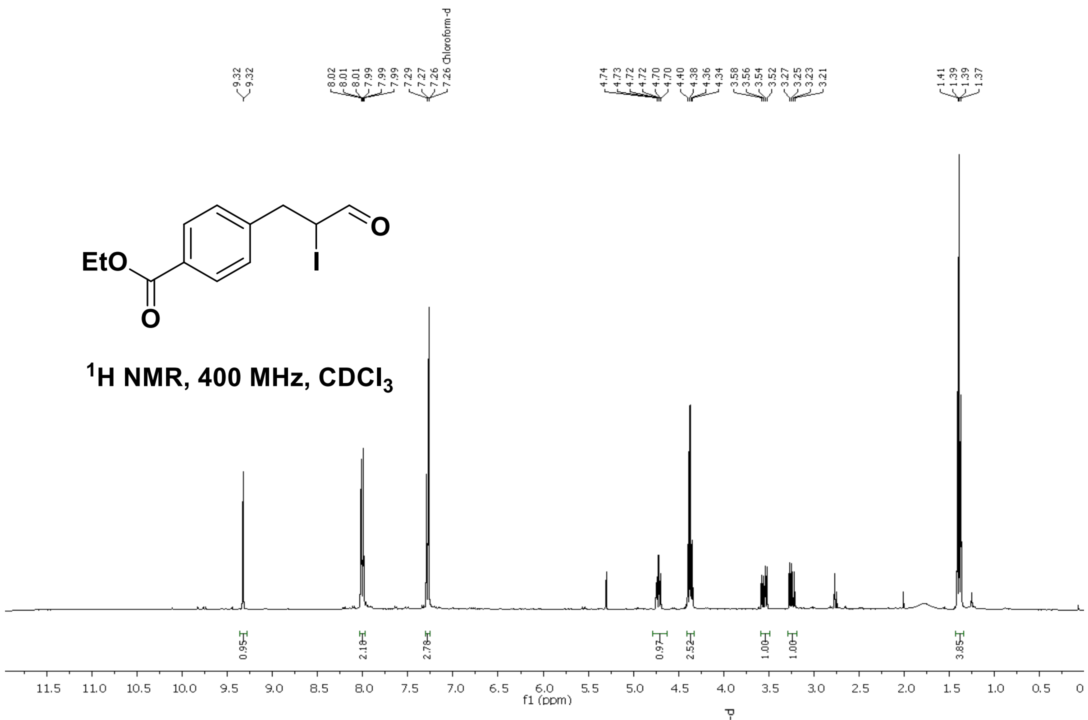

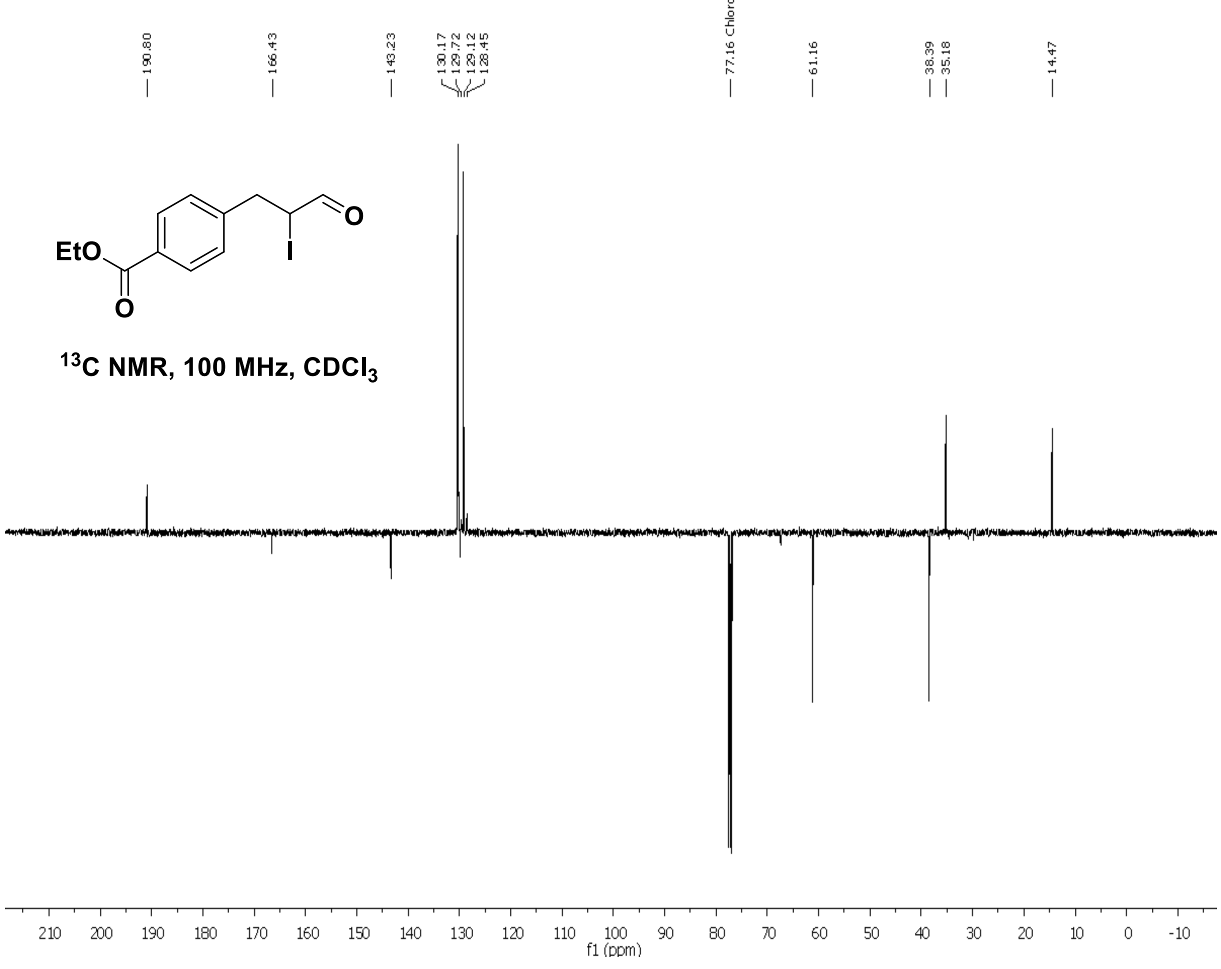

1H NMR and 13C NMR spectra (APT) of compound 43.

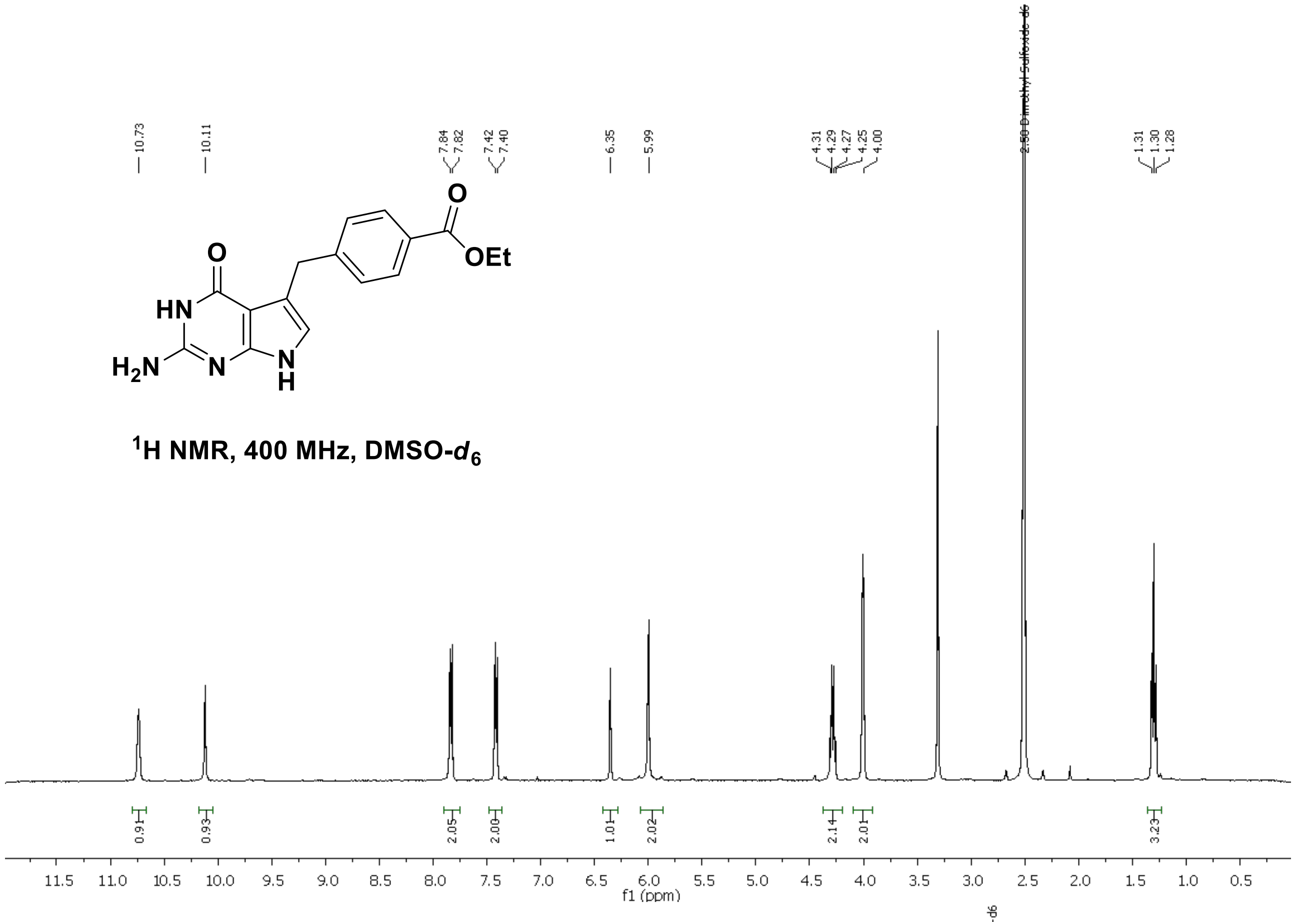

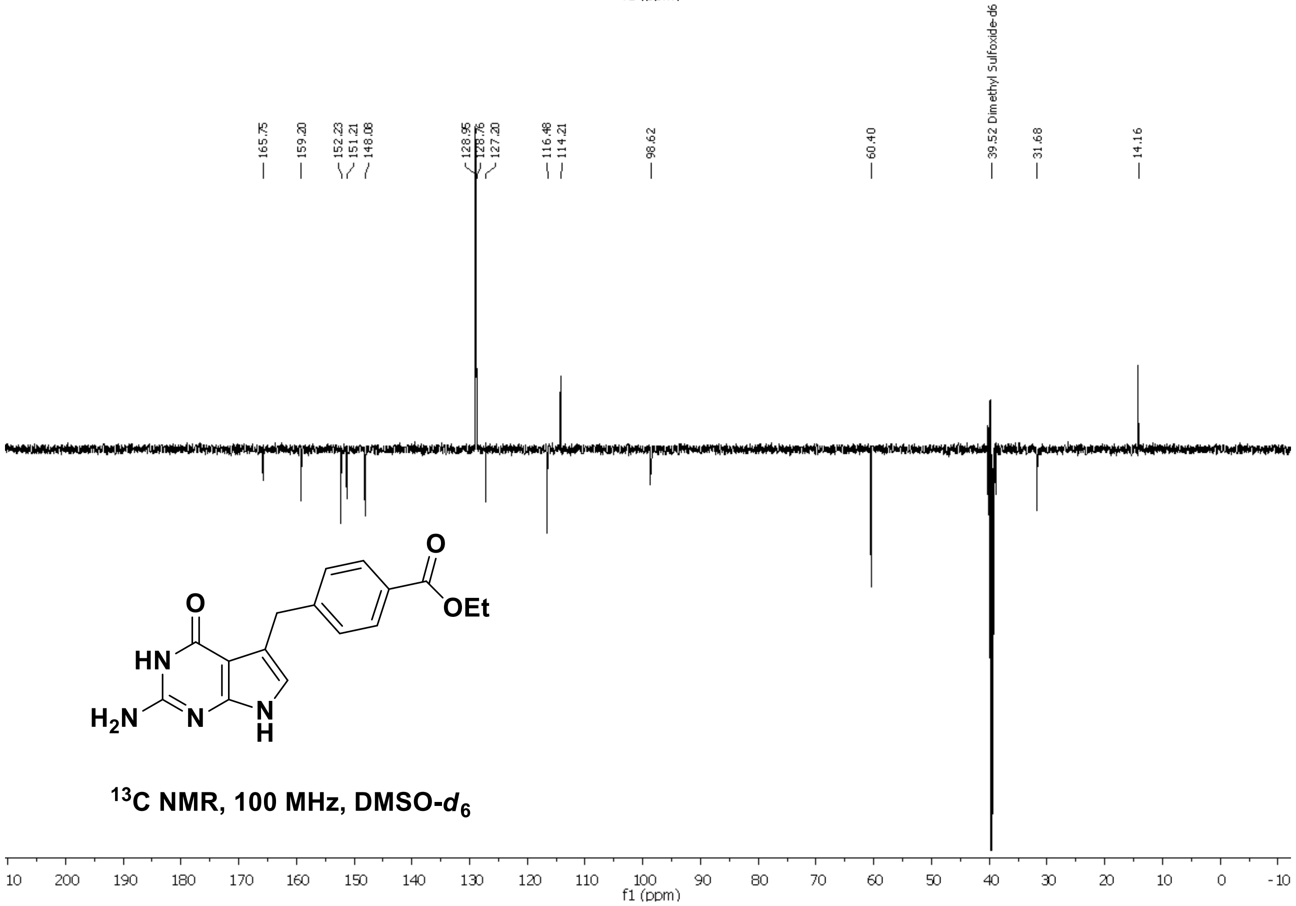

1H NMR and 13C NMR spectra (APT) of compound 44.

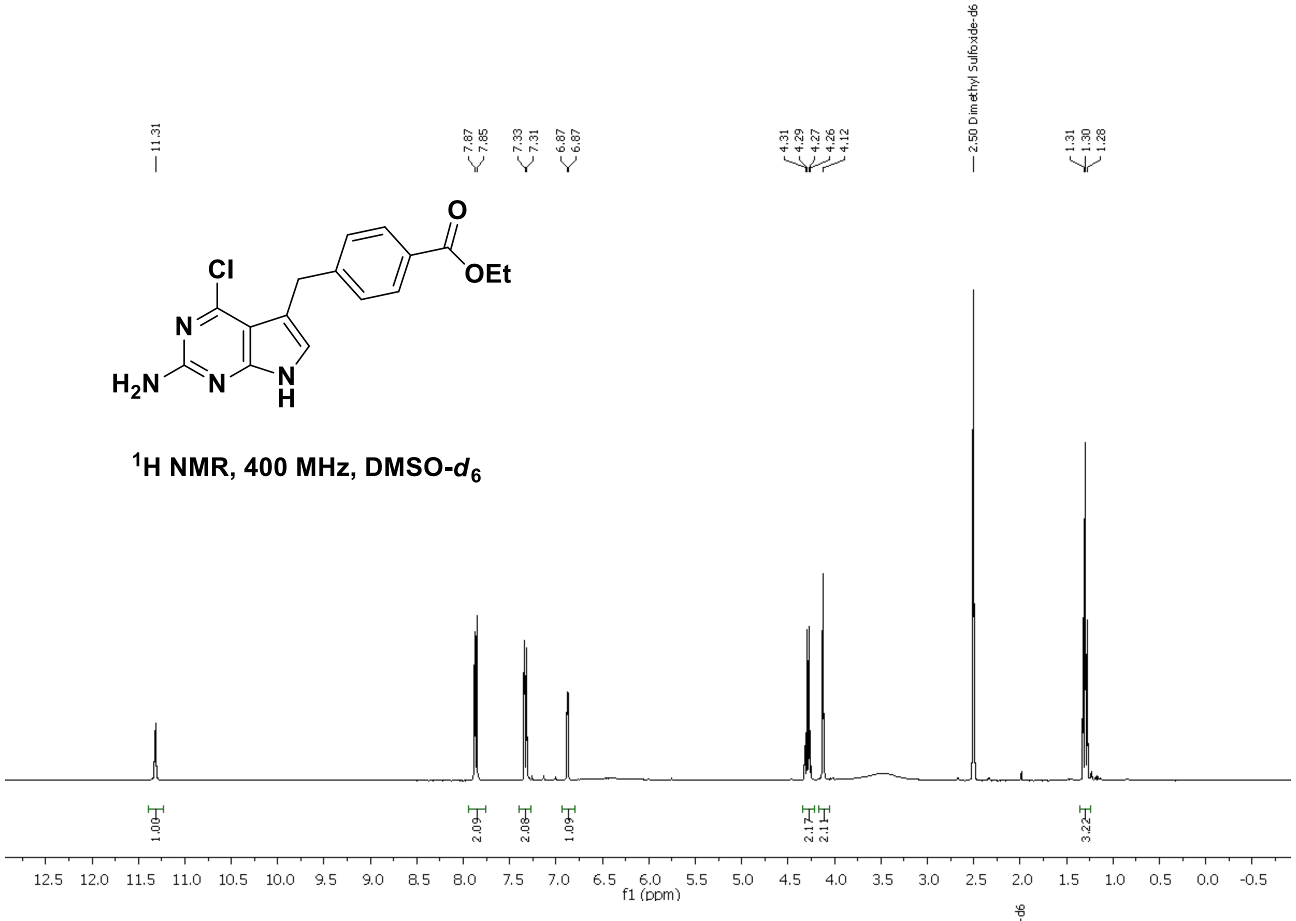

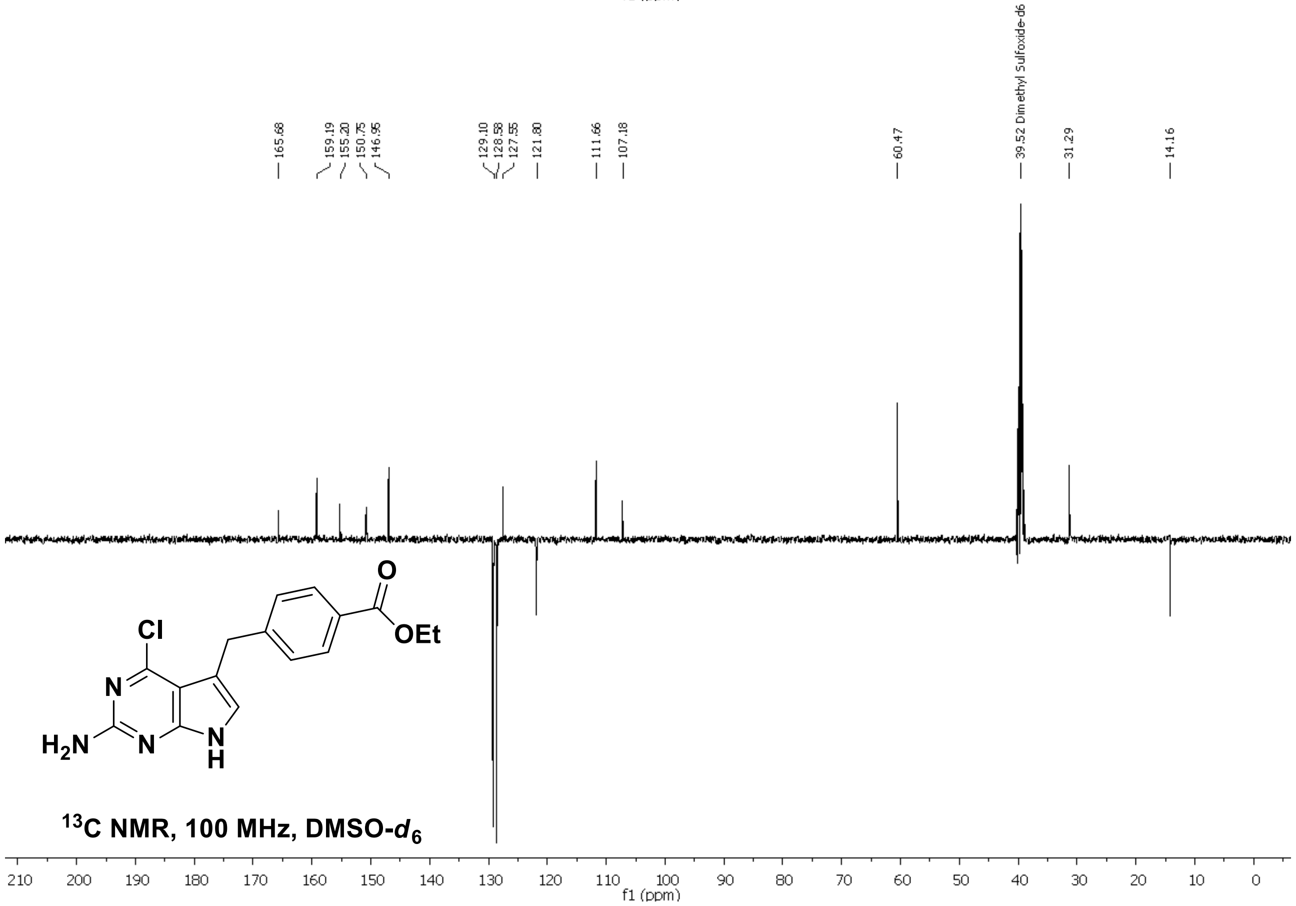

1H NMR and 13C NMR spectra (APT) of compound 45.

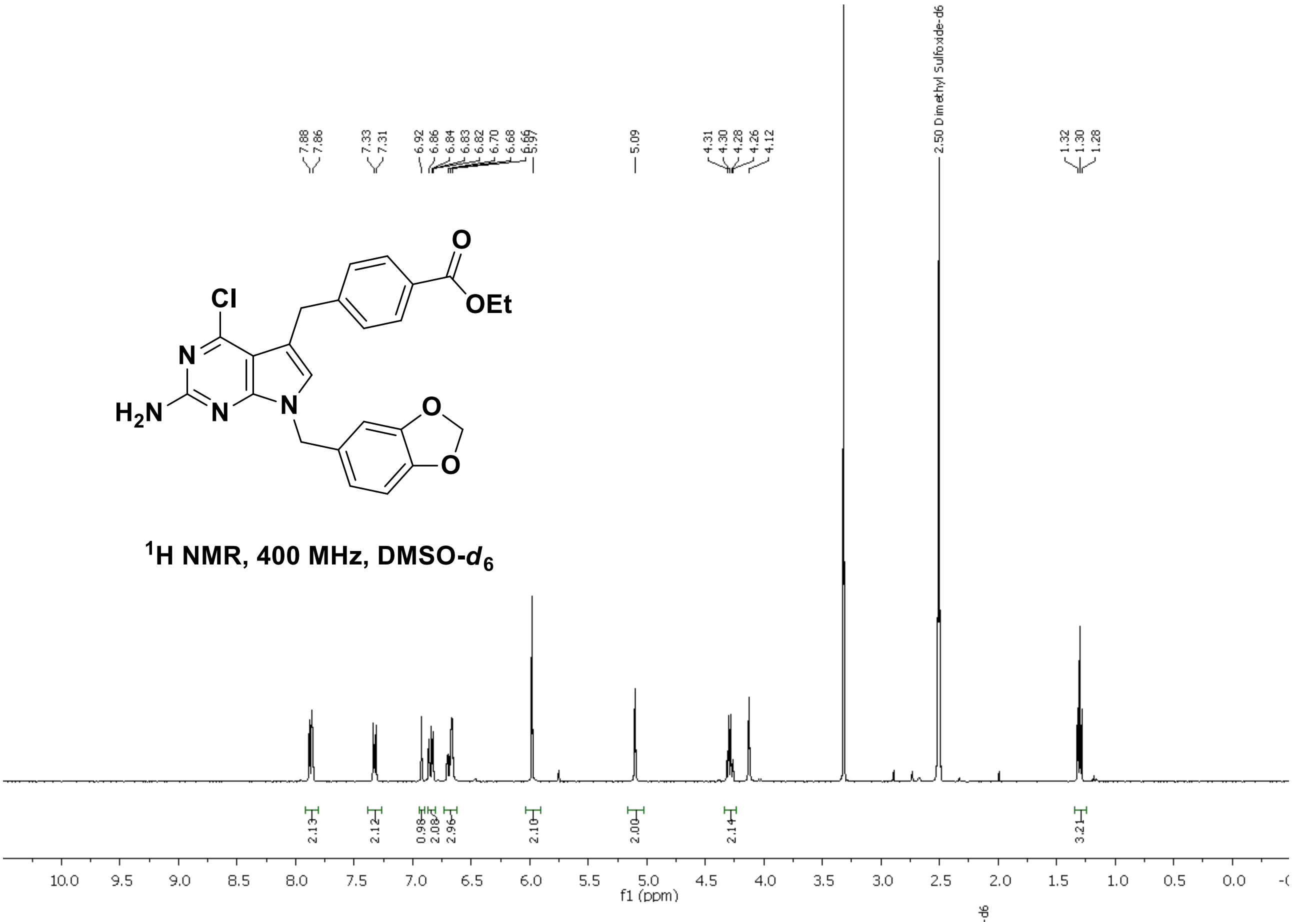

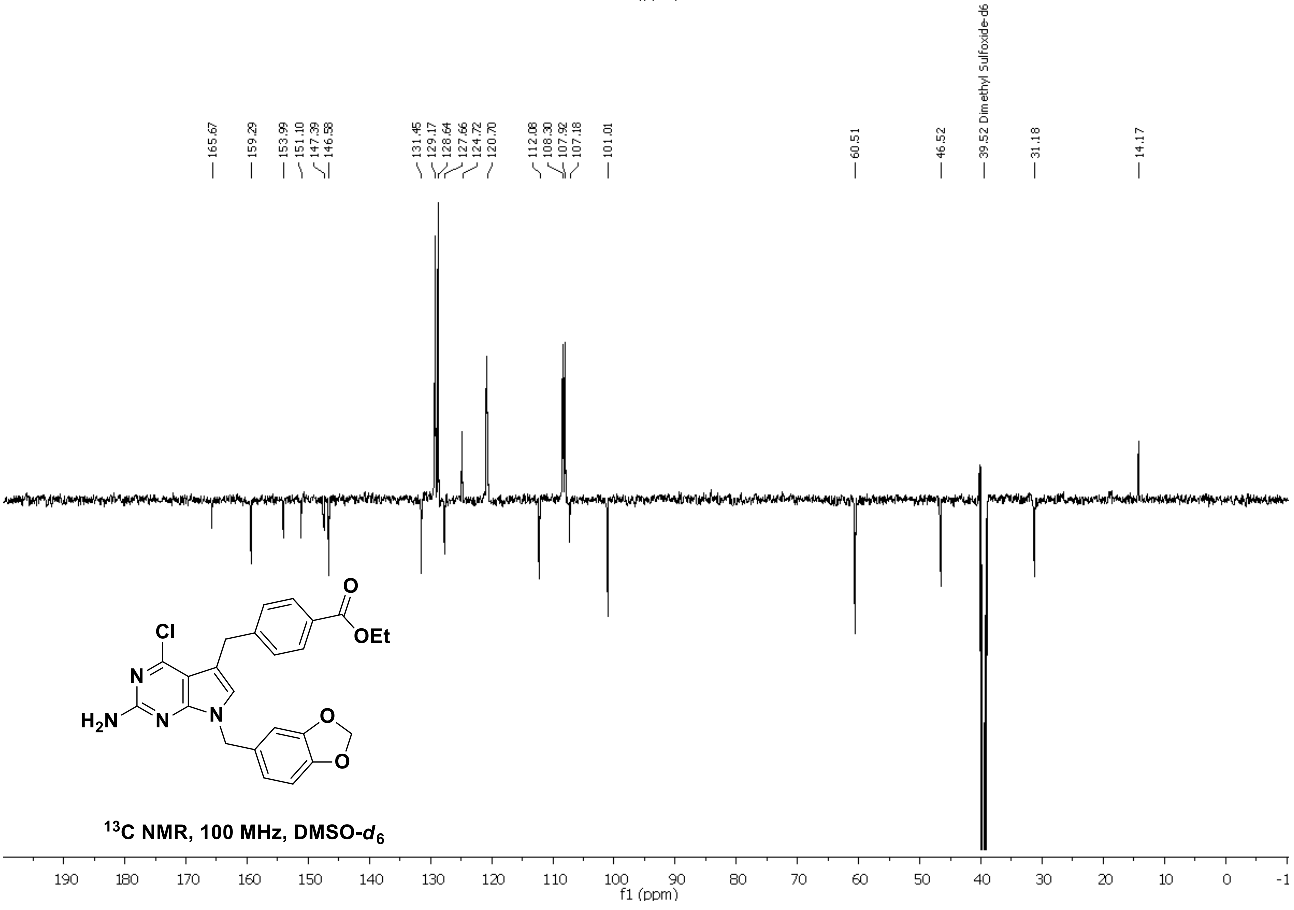

1H NMR and 13C NMR spectra (APT) of compound 46.

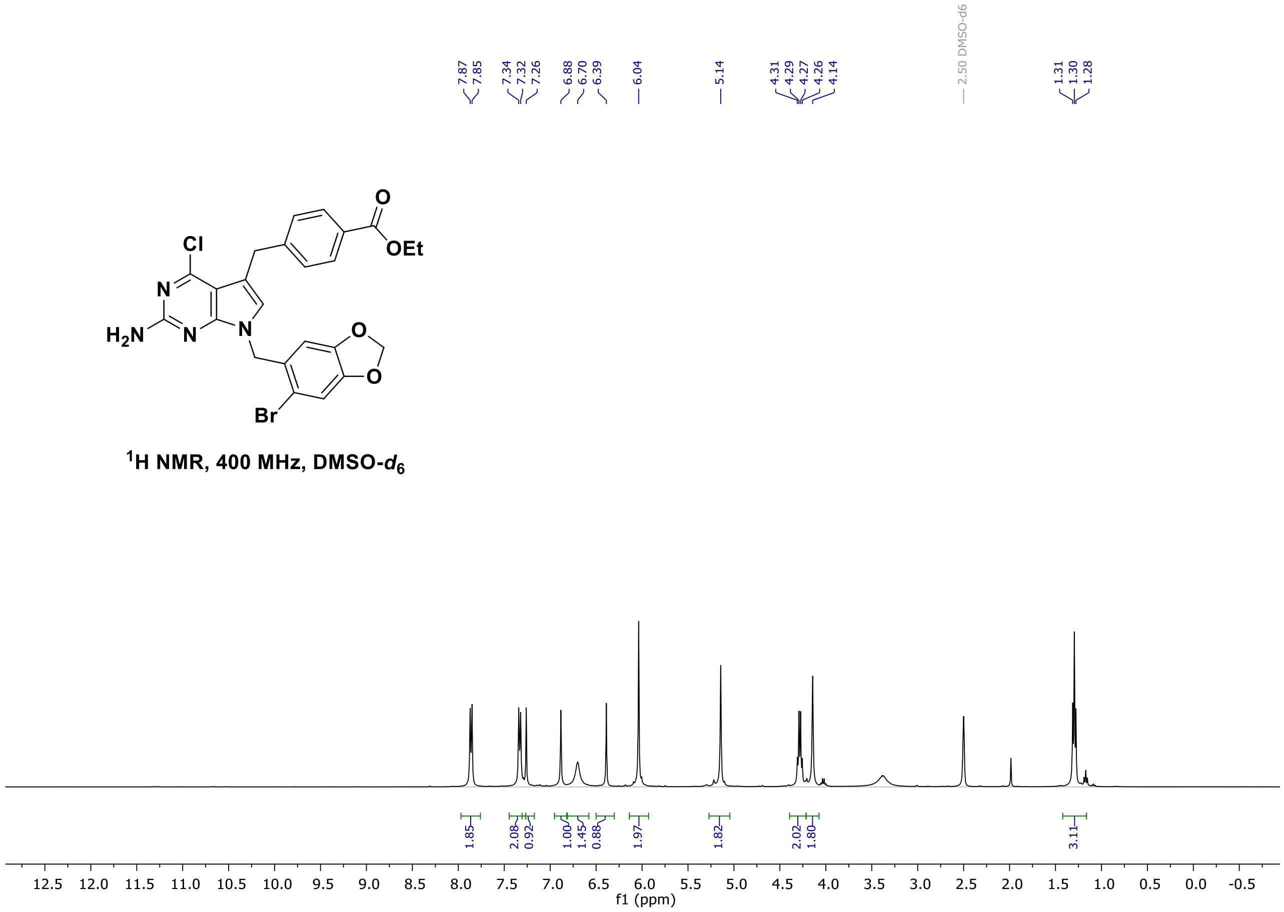

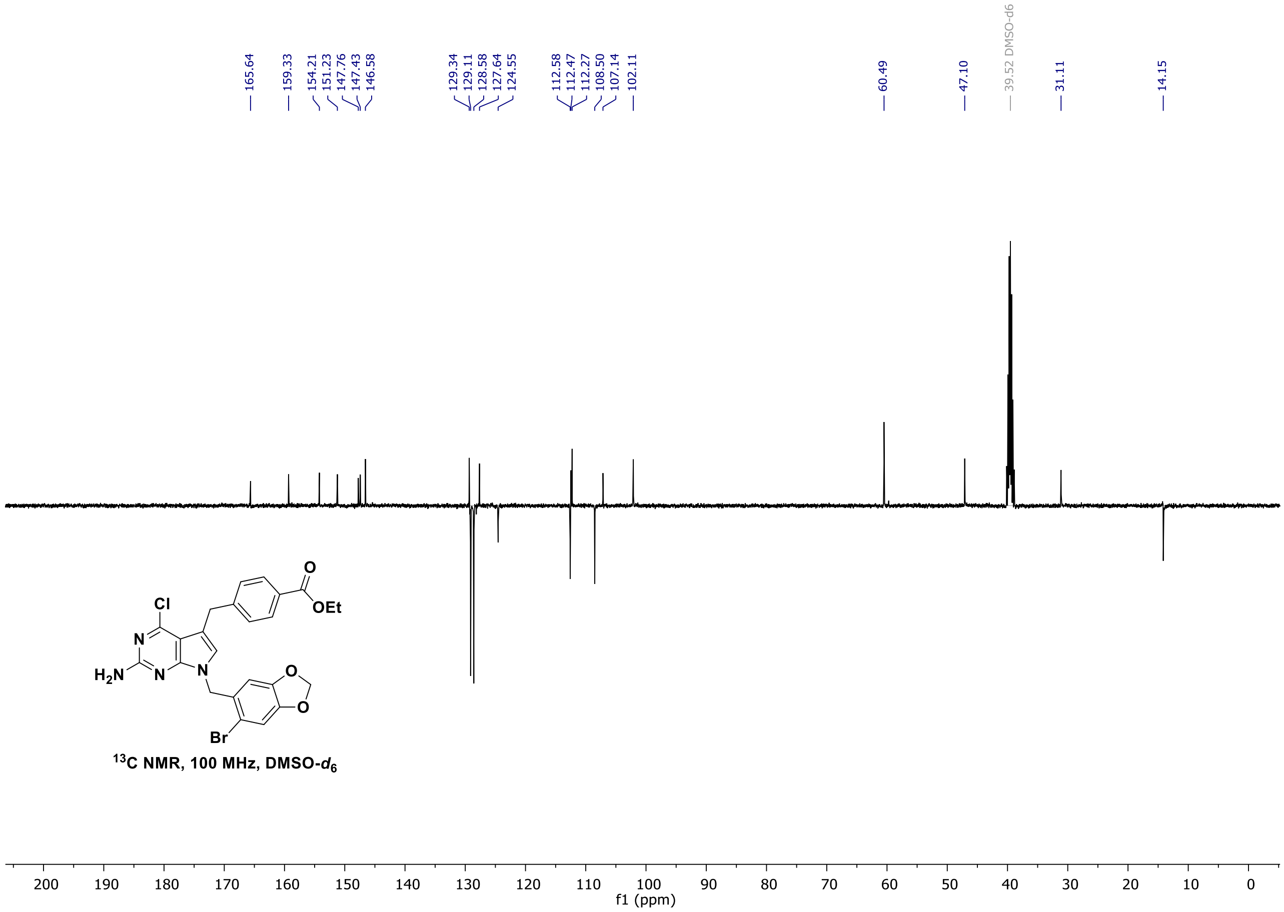

1H NMR and 13C NMR spectra (APT) of compound 47.

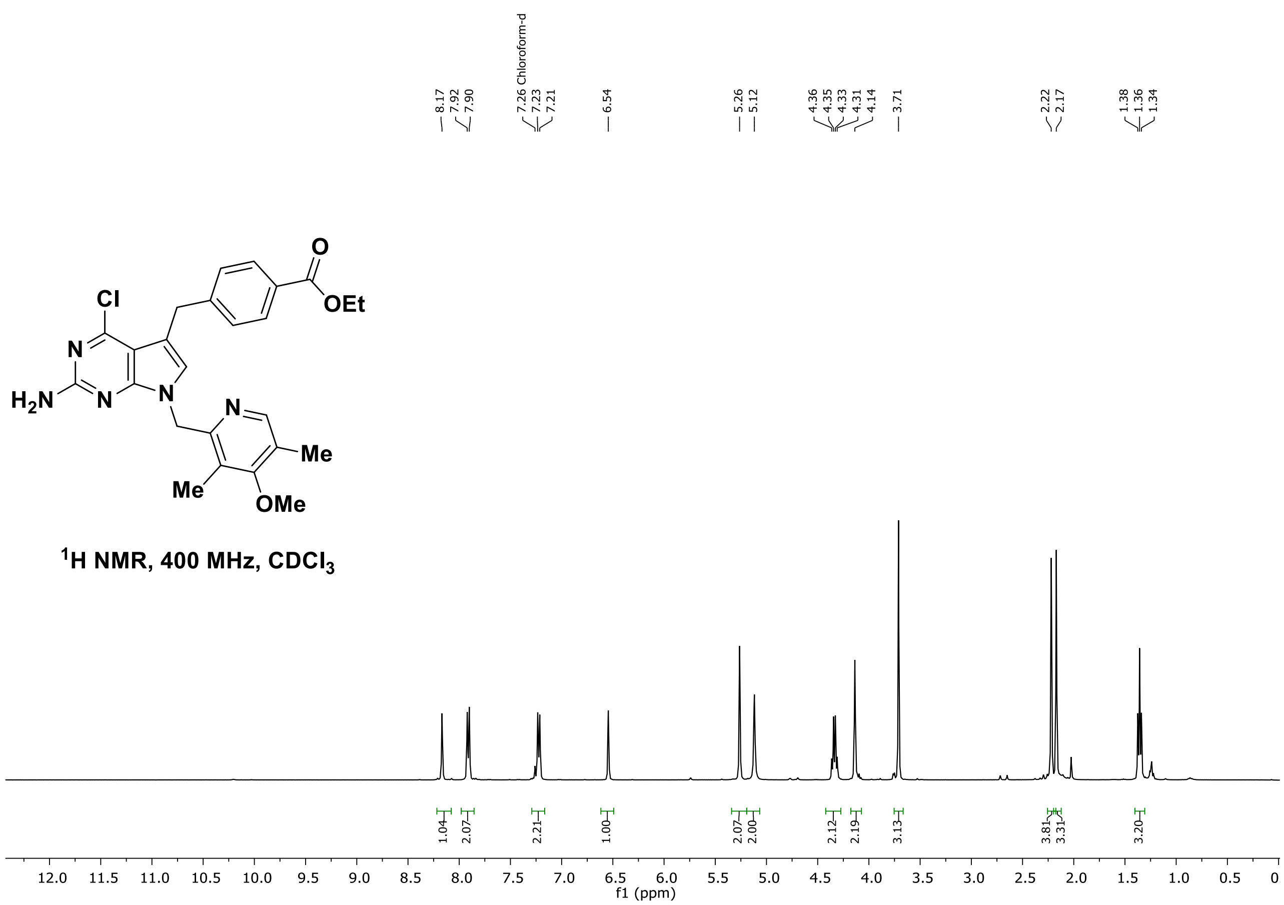

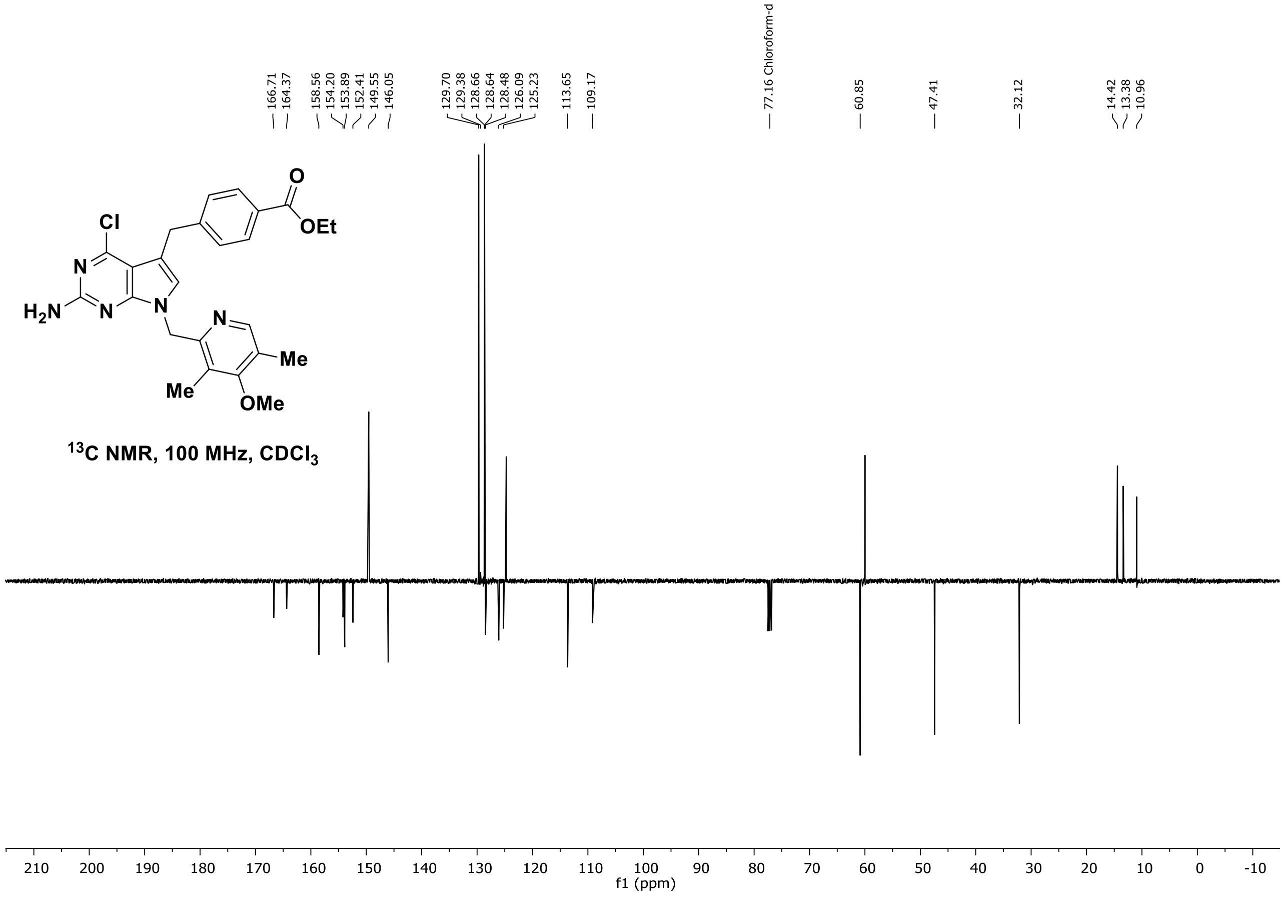

HPLC chromatogram of compound 2.

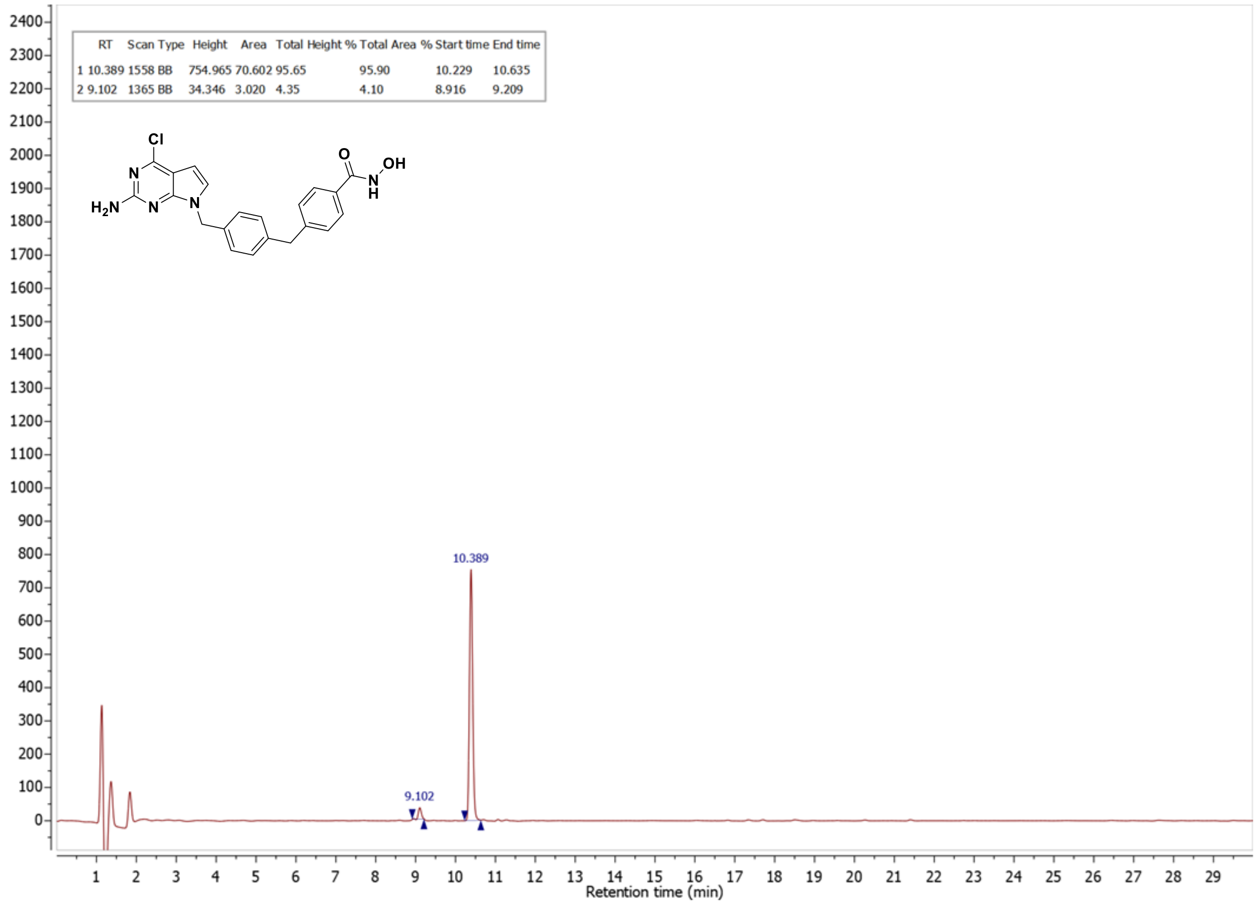

HPLC chromatogram of compound 3.

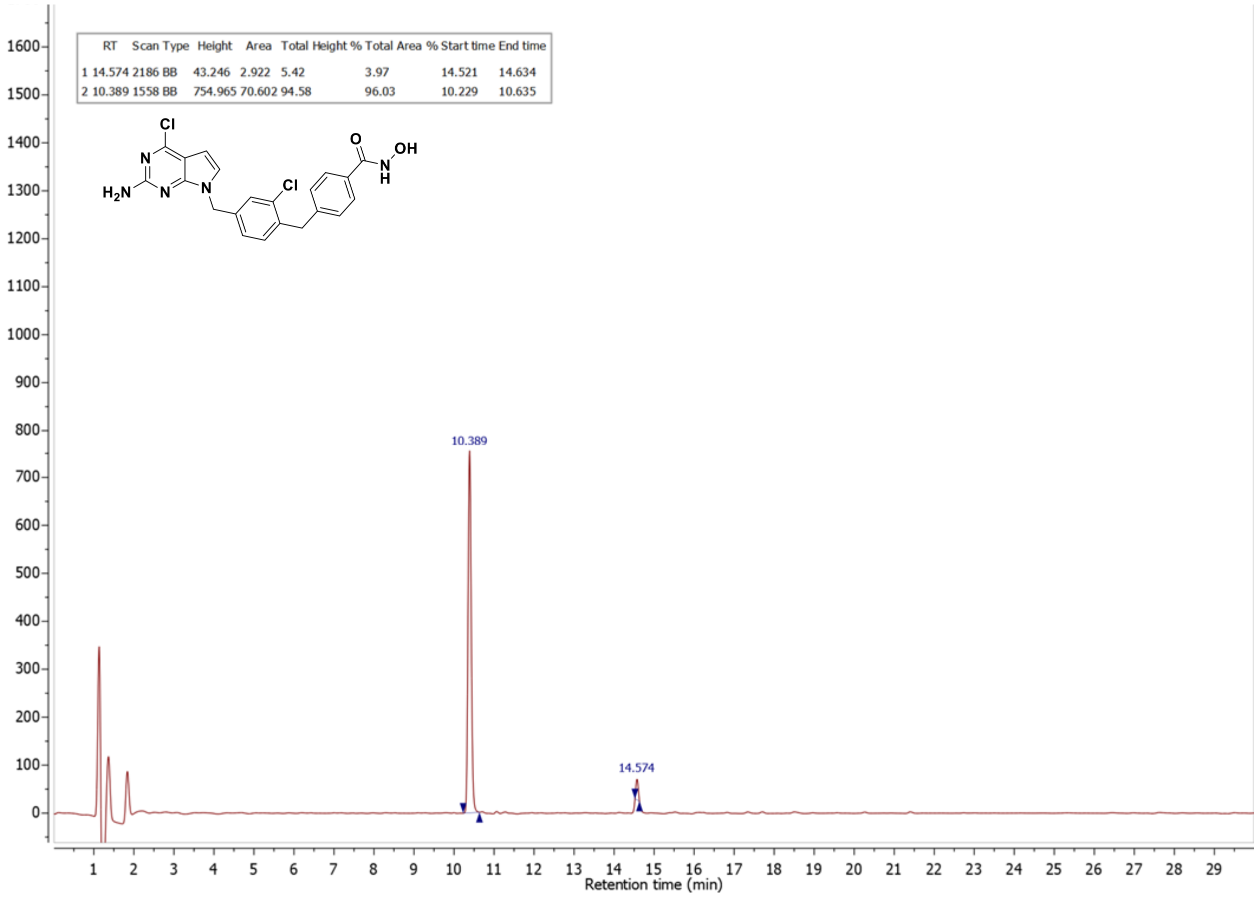

HPLC chromatogram of compound 4.

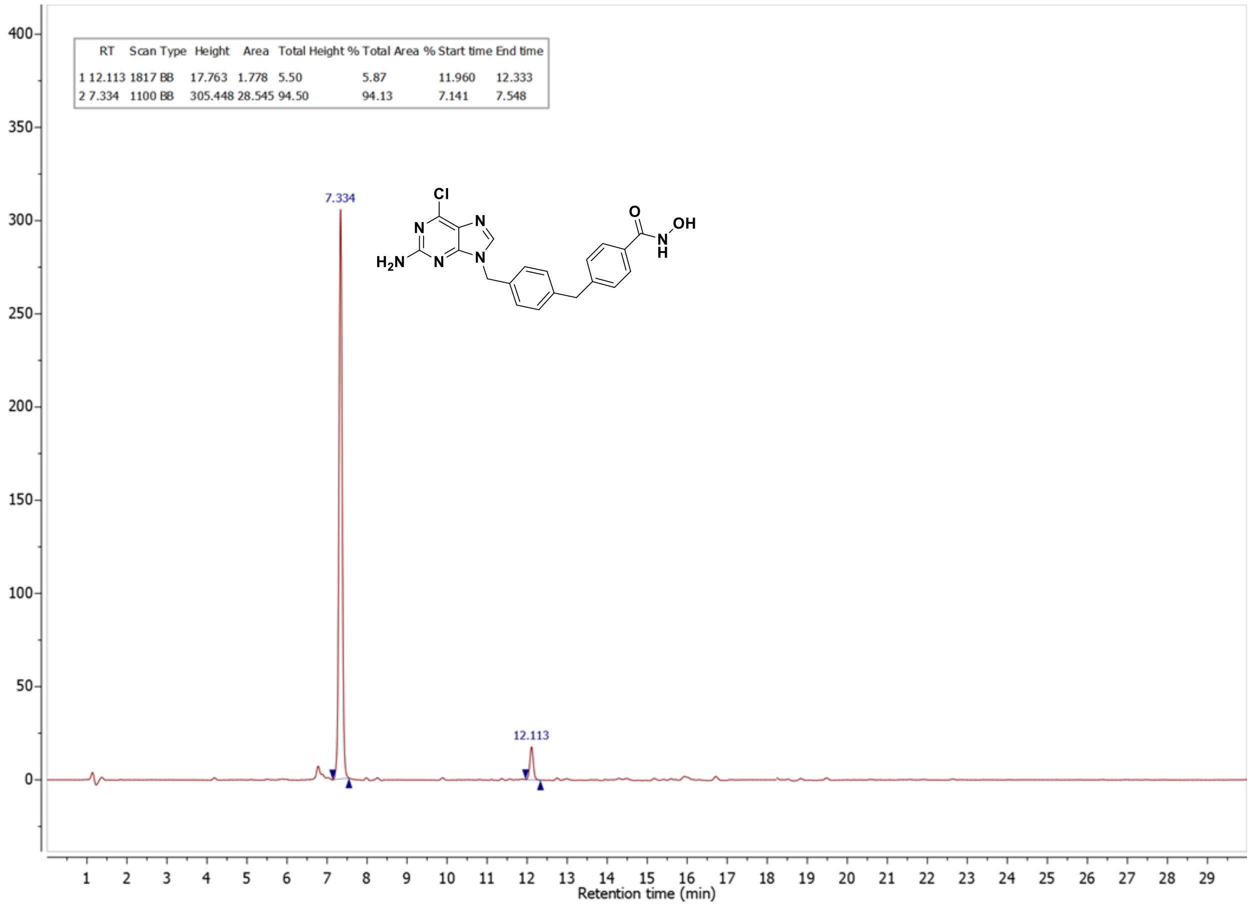

HPLC chromatogram of compound 5.

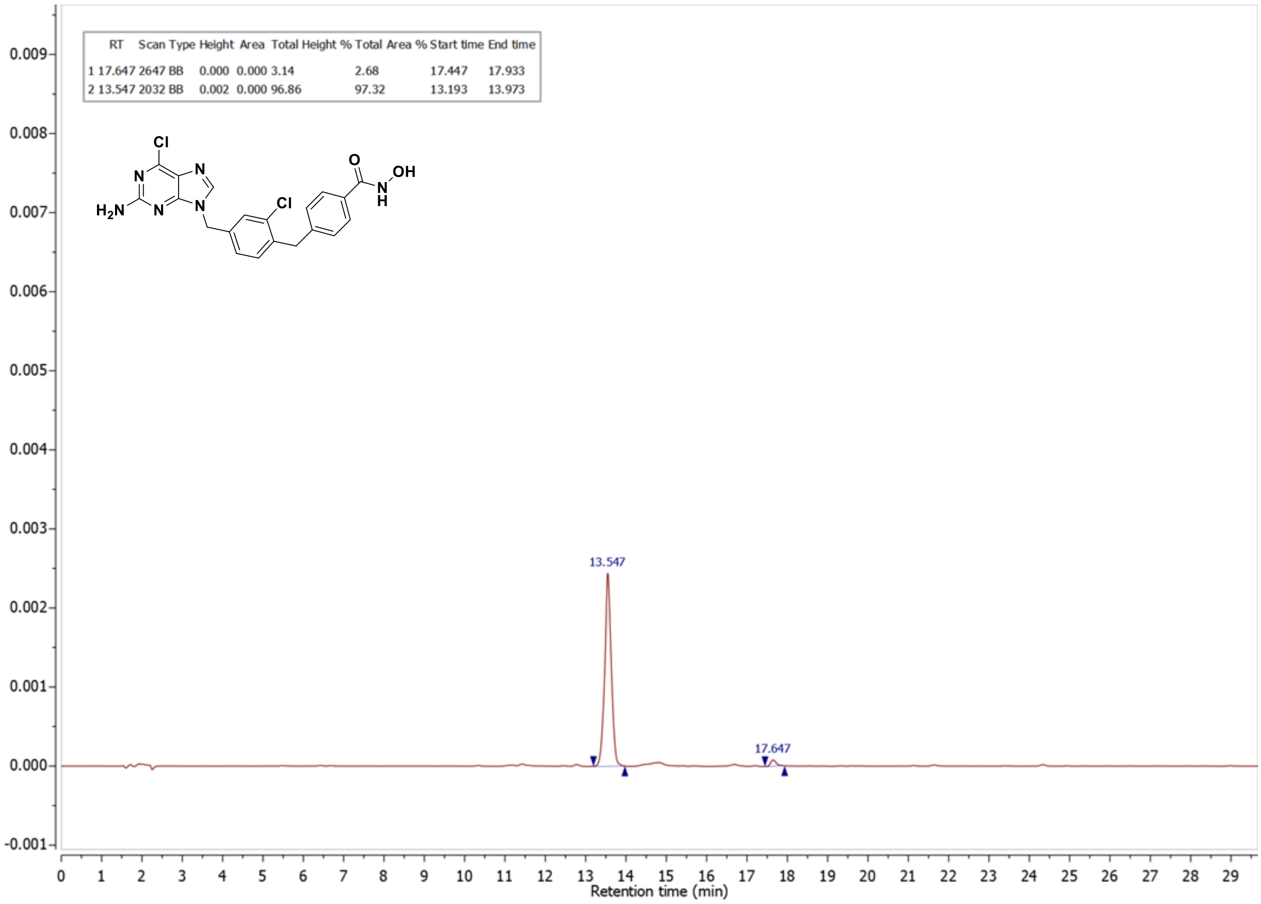

HPLC chromatogram of compound 6.

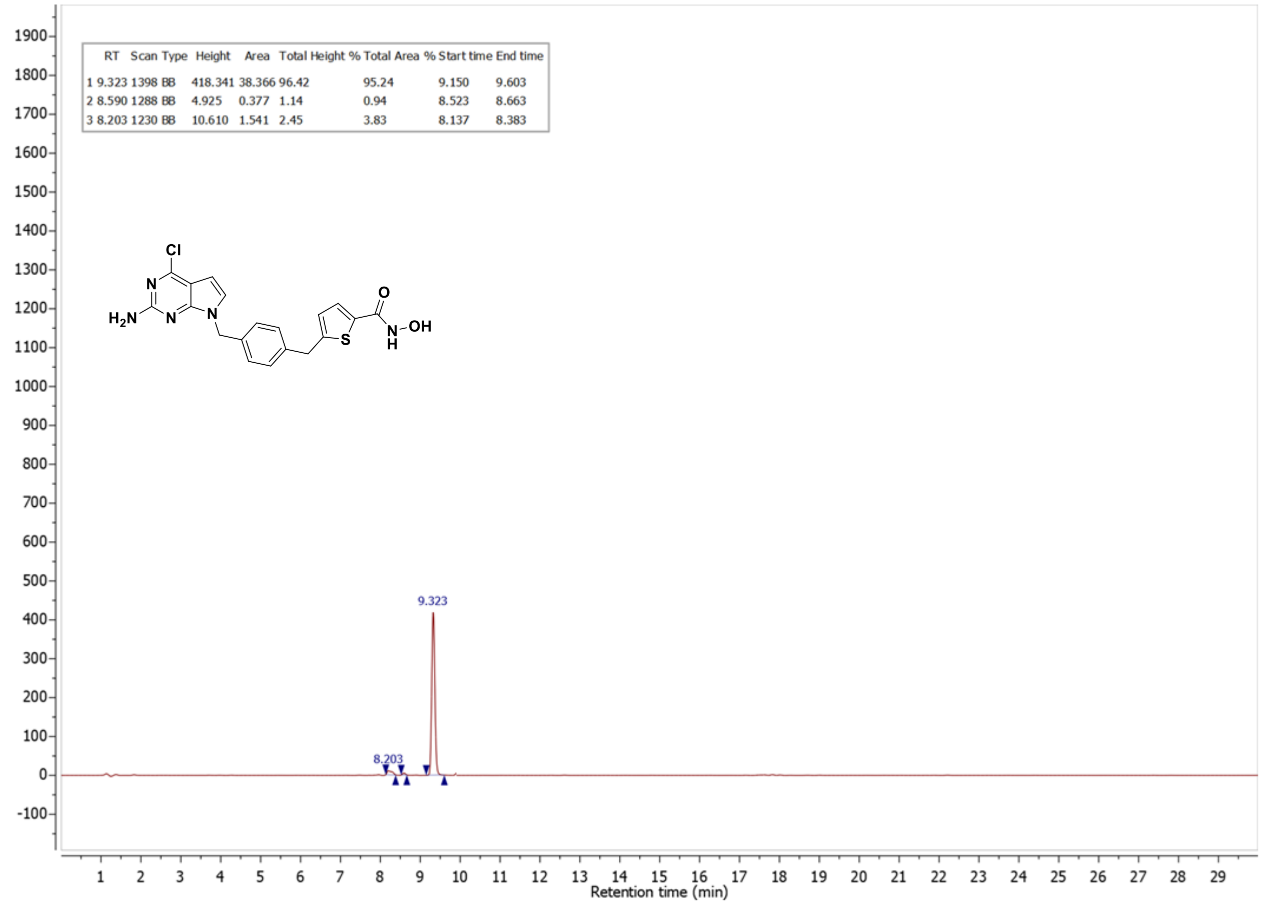

HPLC chromatogram of compound 7.

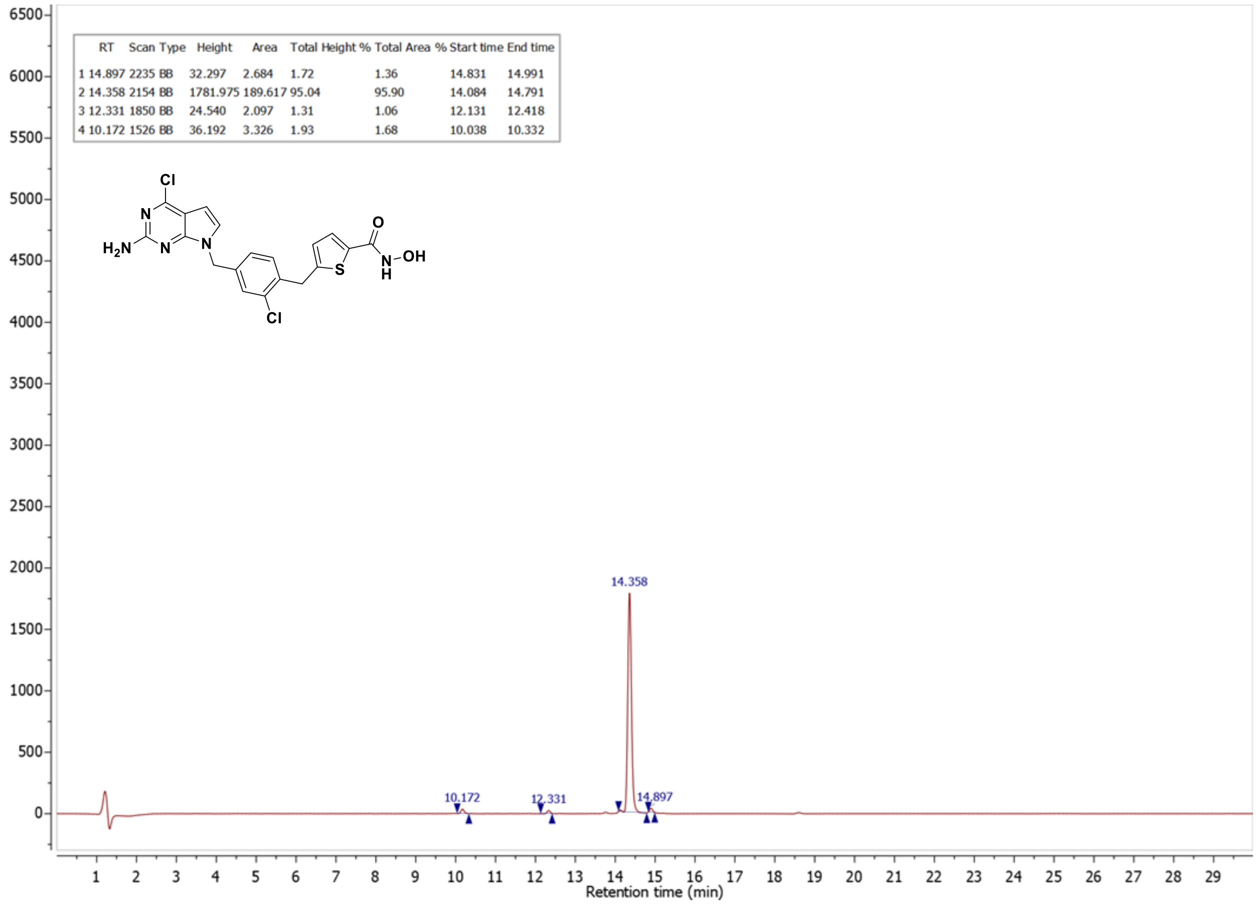

	RT	Scan	Type	Height	Area	Total Height %	Total Area %	Start time	End time
1	14.897	2235	BB	32.297	2.684	1.72	1.36	14.831	14.991
2	14.358	2154	BB	1781.975	189.617	95.04	95.90	14.084	14.791
3	12.331	1850	BB	24.540	2.097	1.31	1.06	12.131	12.418
4	10.172	1526	BB	36.192	3.326	1.93	1.68	10.038	10.332

HPLC chromatogram of compound 8.

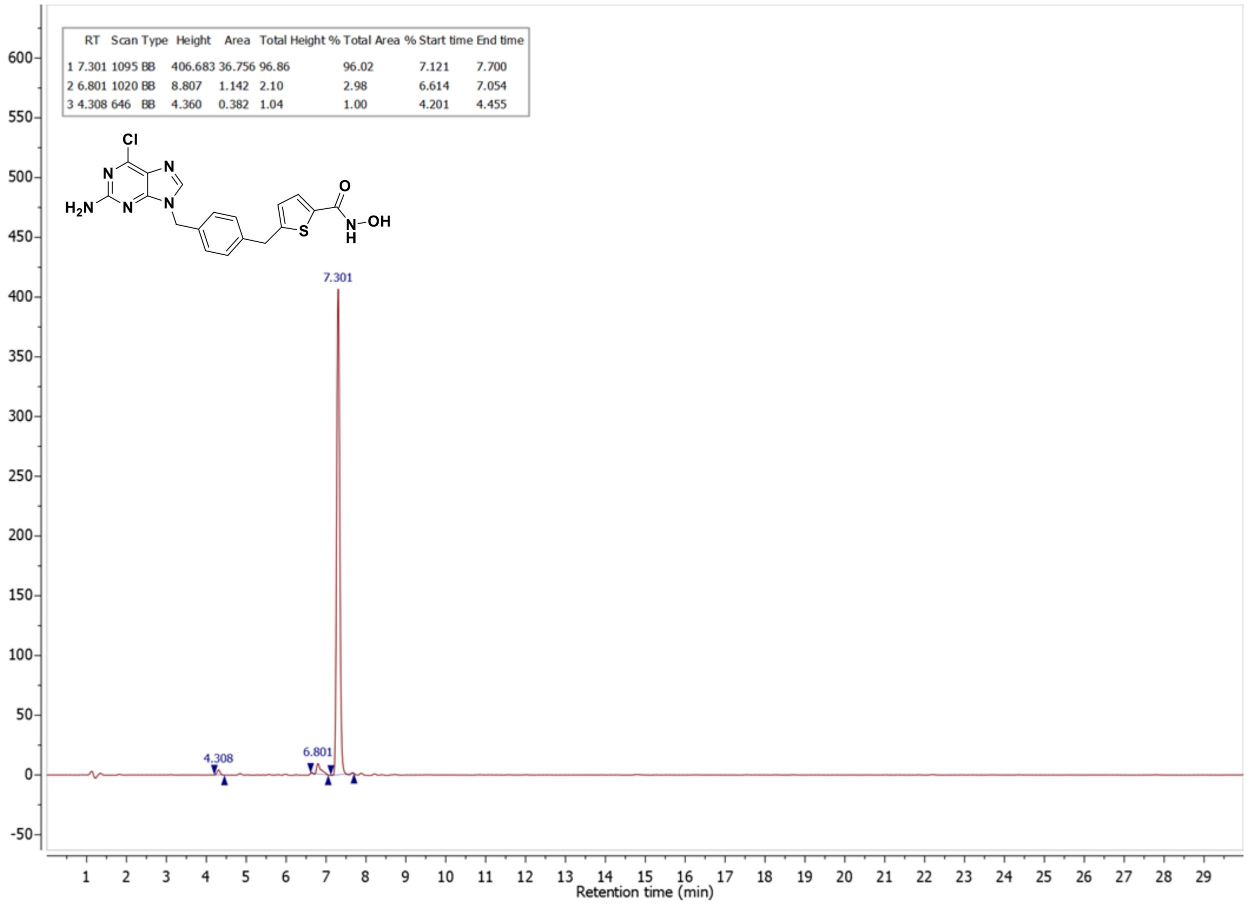

HPLC chromatogram of compound 9.

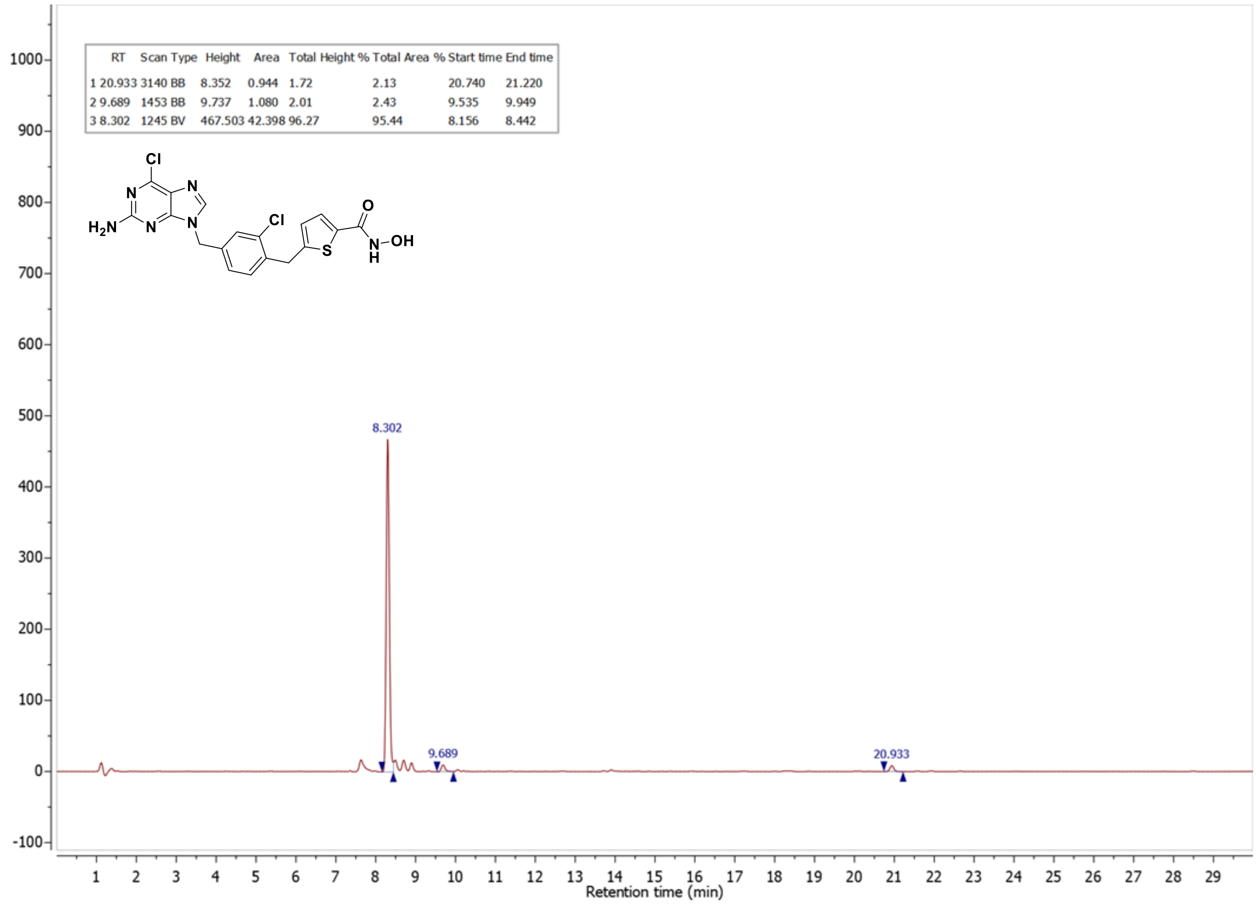

HPLC chromatogram of compound 10.

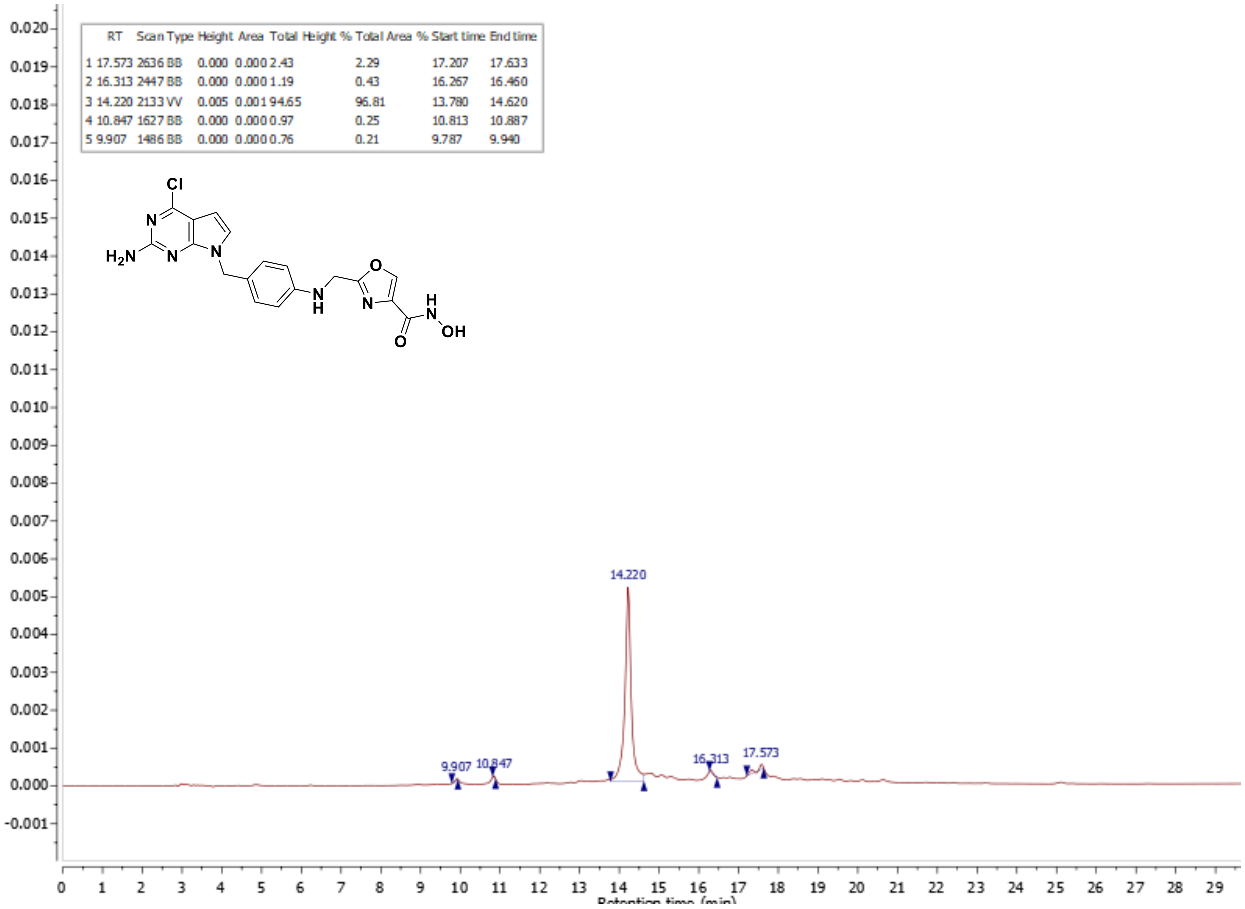

	RT	Scan	Type	Height	Area	Total Height %	Total Area %	Start time	End time
1	17.573	2636	BB	0.000	0.000	2.43	2.29	17.207	17.633
2	16.313	2447	BB	0.000	0.000	1.19	0.43	16.267	16.460
3	14.220	2133	VV	0.005	0.001	94.65	96.81	13.780	14.620
4	10.847	1627	BB	0.000	0.000	0.97	0.25	10.813	10.887
5	9.907	1486	BB	0.000	0.000	0.76	0.21	9.787	9.940

HPLC chromatogram of compound 11.

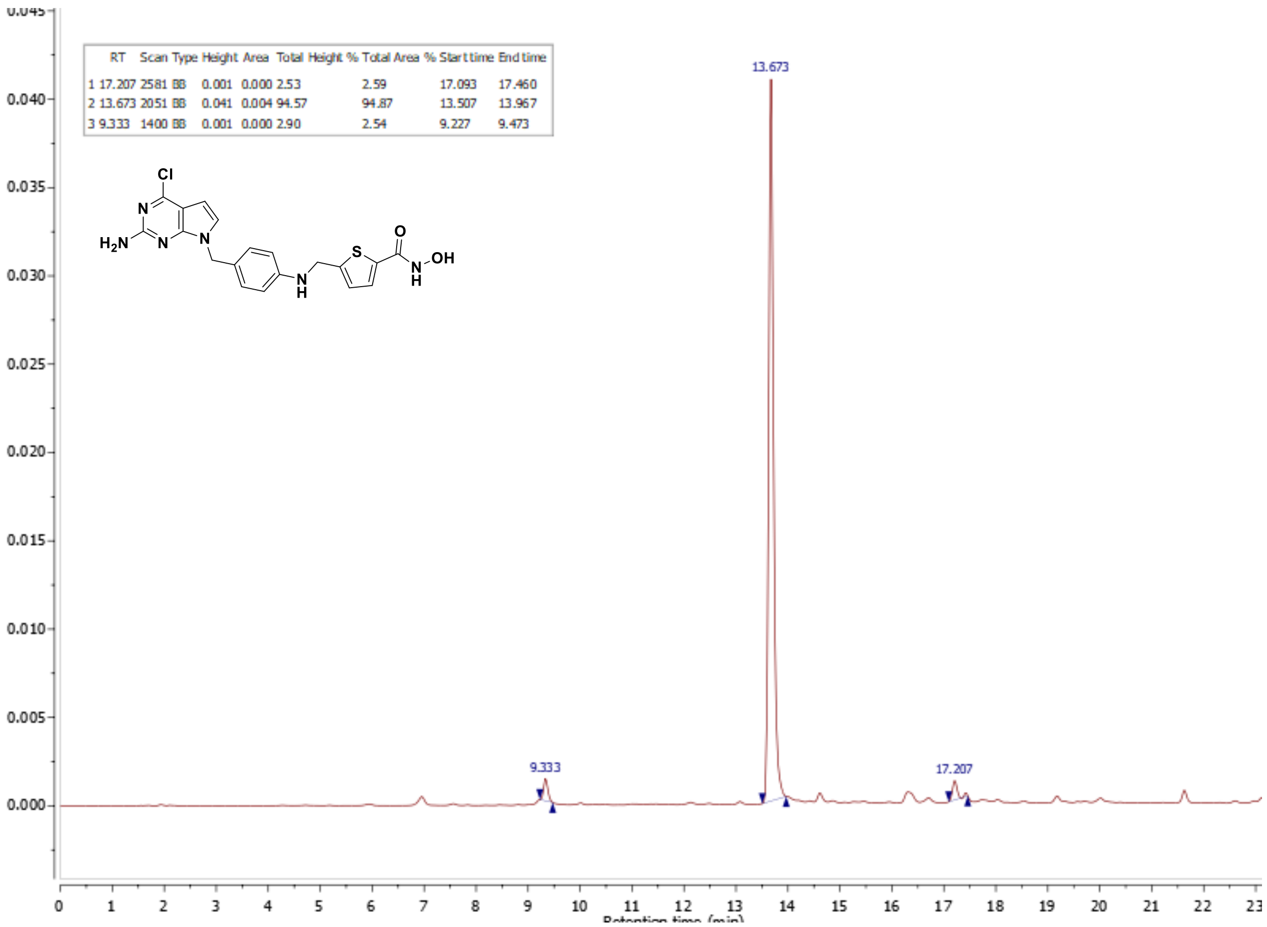

	RT	Scan	Type	Height	Area	Total Height %	Total Area %	Start time	End time
1	17.207	2581	BB	0.001	0.000	2.53	2.59	17.093	17.460
2	13.673	2051	BB	0.041	0.004	94.57	94.87	13.507	13.967
3	9.333	1400	BB	0.001	0.000	2.90	2.54	9.227	9.473

HPLC chromatogram of compound 12.

	RT	Scan	Type	Height	Area	Total Height %	Total Area %	Start time	End time
1	12.227	1834	BB	0.057	0.006	95.97	97.19	11.953	12.500
2	10.033	1505	BB	0.002	0.000	4.03	2.81	9.953	10.087

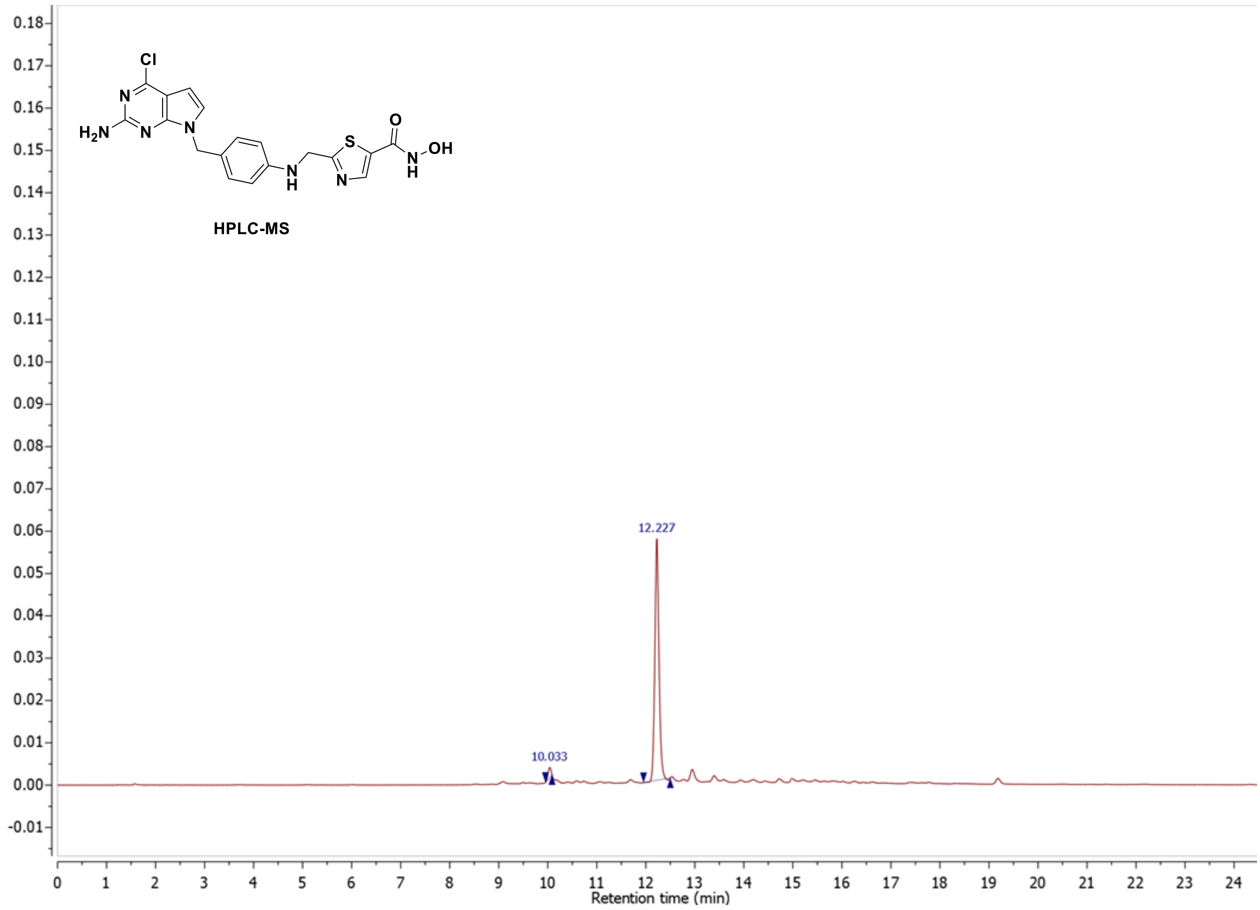

HPLC chromatogram of compound 13.

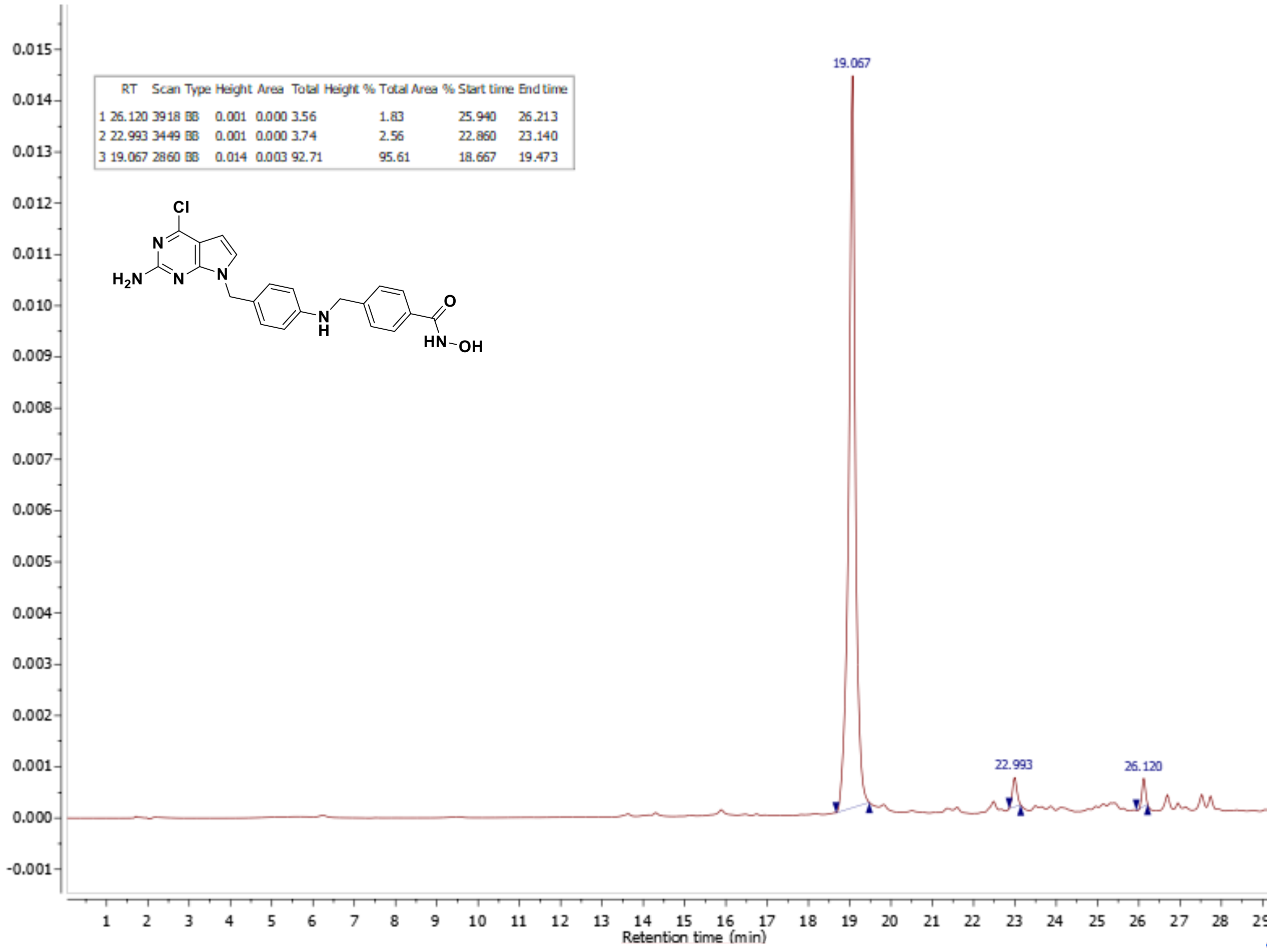

	RT	Scan	Type	Height	Area	Total Height %	Total Area %	Start time	End time
1	26.120	3918	BB	0.001	0.000	3.56	1.83	25.940	26.213
2	22.993	3449	BB	0.001	0.000	3.74	2.56	22.860	23.140
3	19.067	2860	BB	0.014	0.003	92.71	95.61	18.667	19.473

LCMS of compound 14.

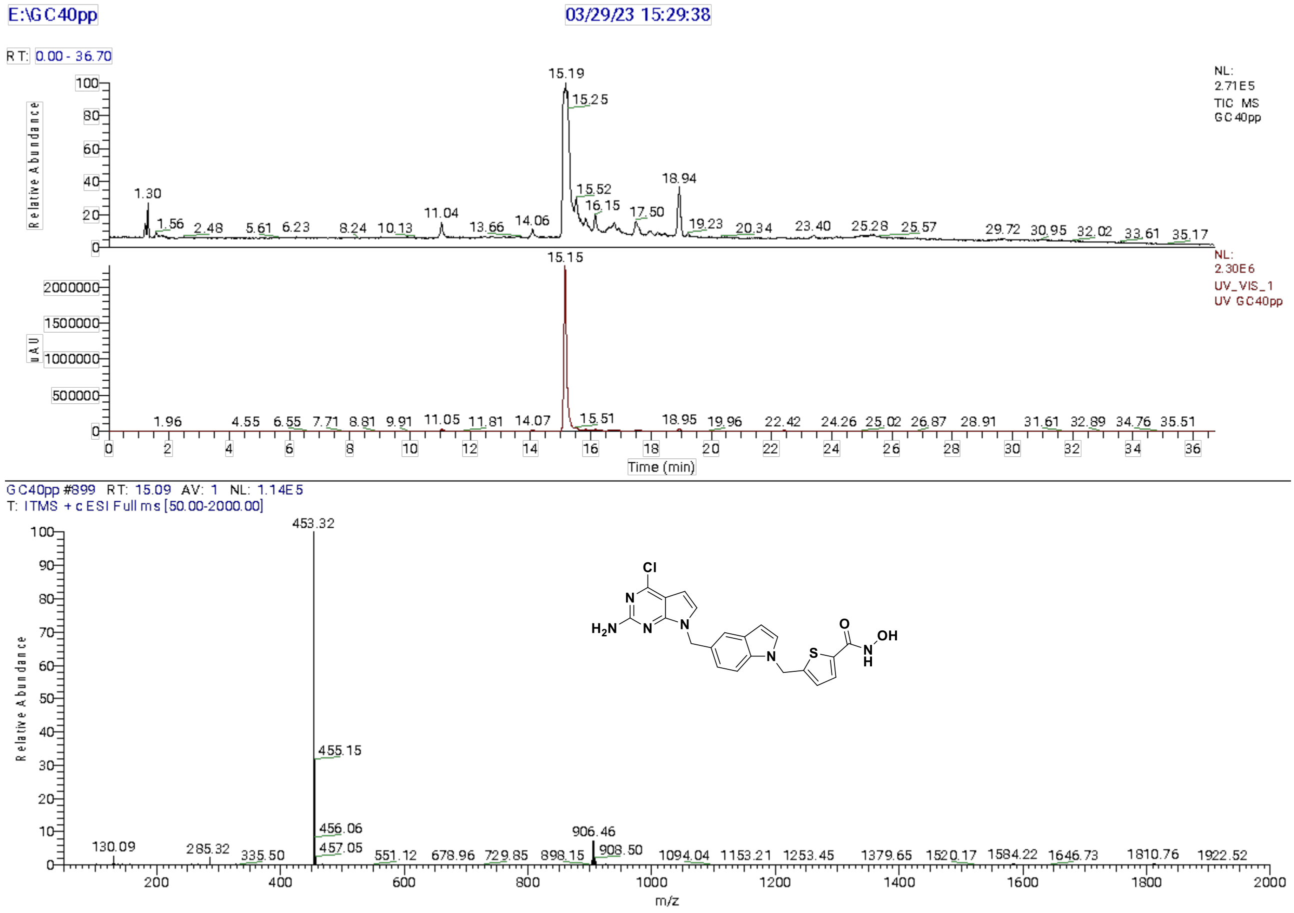

HPLC chromatogram of compound 15.

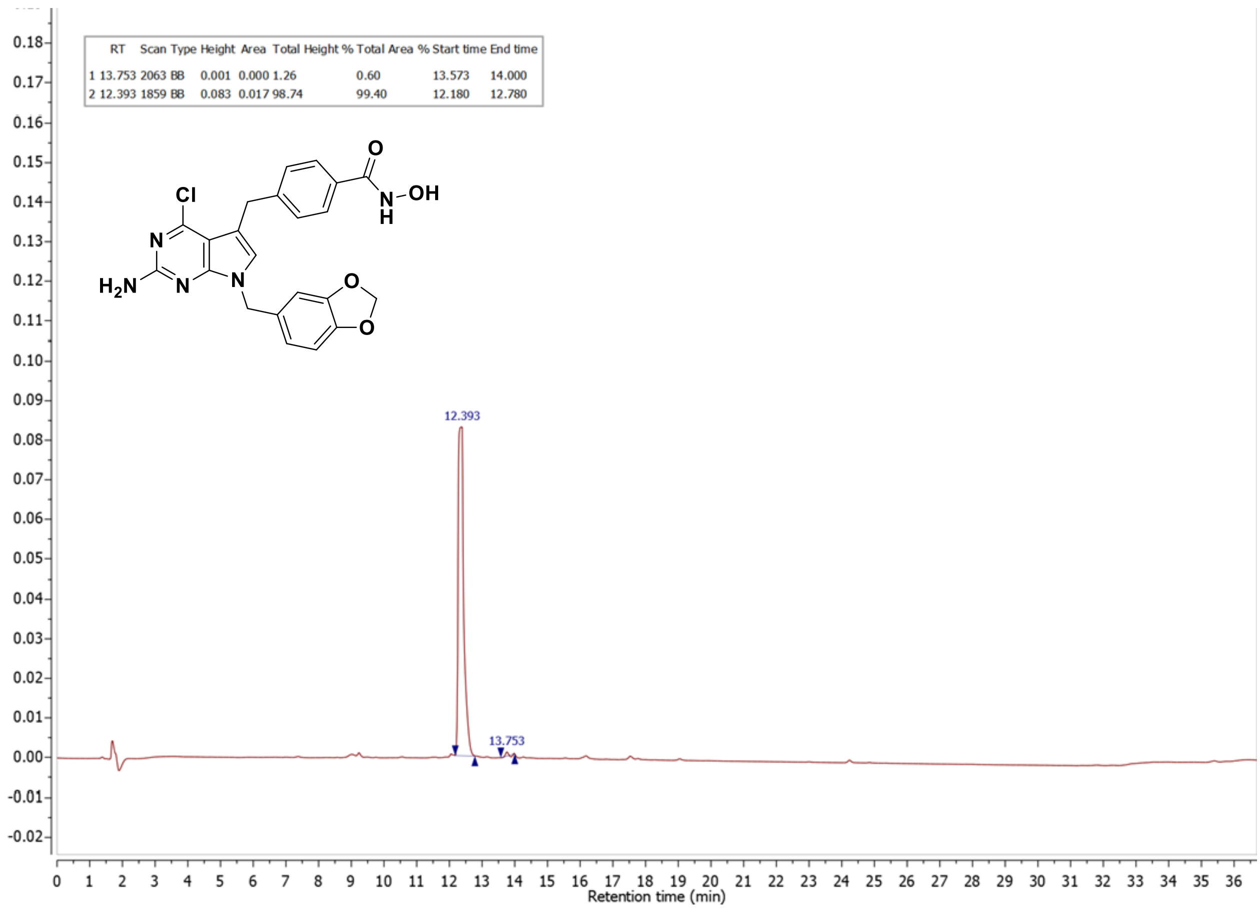

HPLC chromatogram of compound 16.

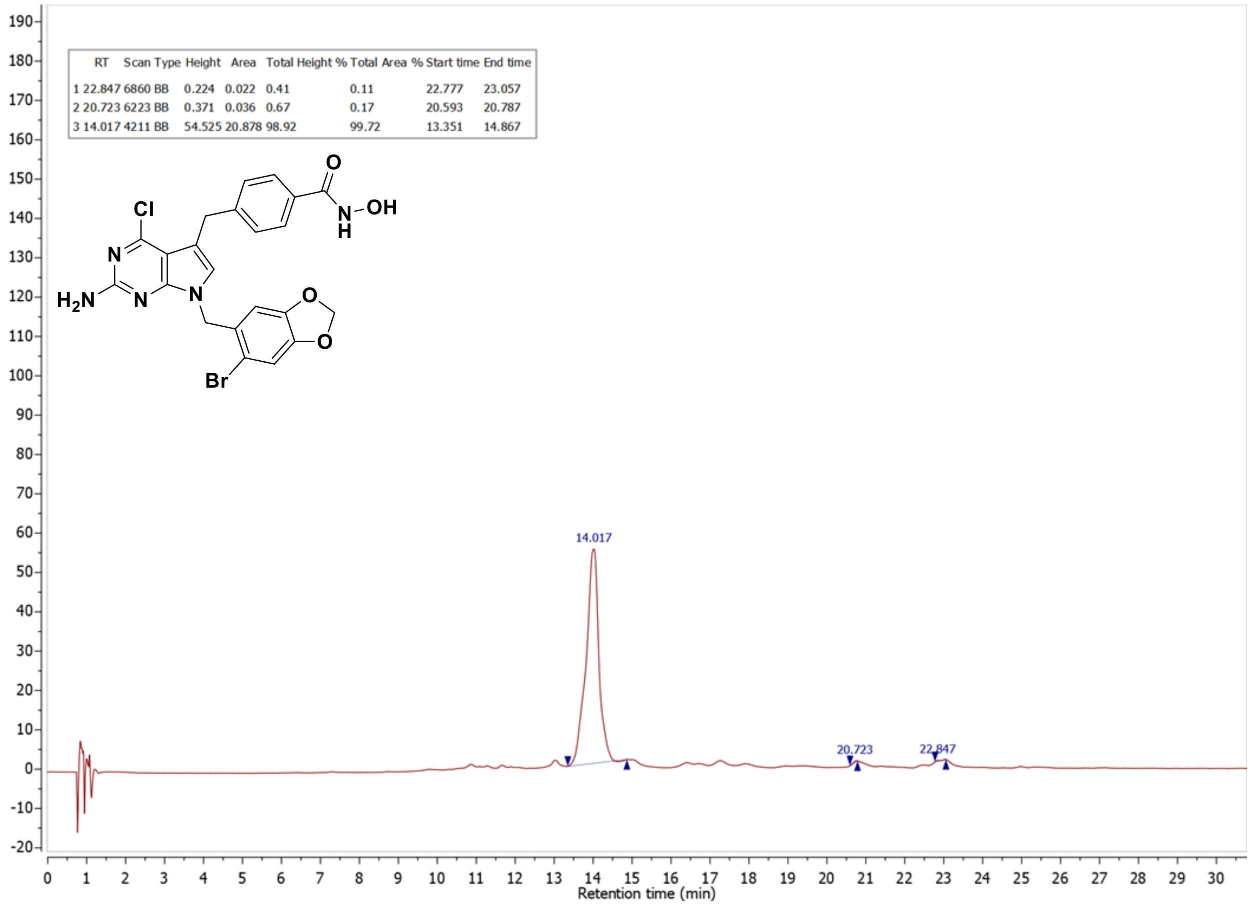

HPLC chromatogram of compound 17.

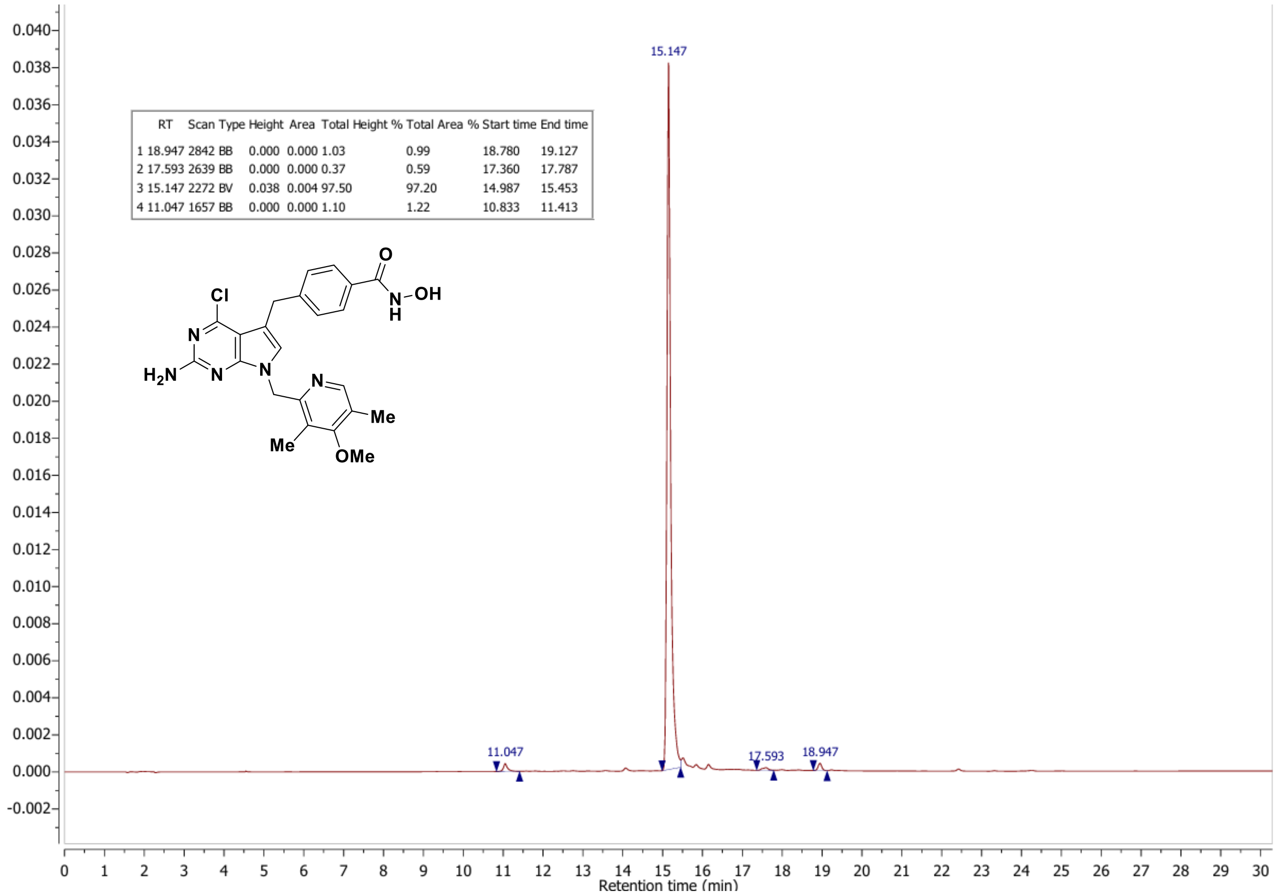

Supplement: Supplementary file 1 [file jm5c00717_si_001.pdf]
